# Birds of the Tolima Department of Colombia’s central Andean Region

**DOI:** 10.3897/BDJ.12.e68286

**Published:** 2025-11-27

**Authors:** Ronald M Parra-Hernández, Yair G Molina-Martínez

**Affiliations:** 1 Asociación Tolimense de Ornitología – Anthocephala, Calle 45 # 9ª – 19, Limonar 5to Sector, Ibagué, Colombia Asociación Tolimense de Ornitología – Anthocephala Calle 45 # 9ª – 19, Limonar 5to Sector, Ibagué Colombia; 2 Universidad de Ibagué, Carrera 22 calle 67, Ibagué, Colombia Universidad de Ibagué Carrera 22 calle 67, Ibagué Colombia

**Keywords:** bird diversity, distribution, habitat, species check-list

## Abstract

**Background:**

The information in this article corresponds to a compilation of data from scientific journals, ornithological collections and virtual libraries (xeno-song, eBird and Biomap), as well as our contributions during more than 15 years of studies in Tolima. Together, this information allows us to understand the diversity of birds in the region. In addition, this article includes data on localities and the altitudinal and geographical distribution of each species. The results indicate the presence of 800 bird species in Tolima, of which 24 are endemic, 69 are near-endemic and 82 are migratory. Furthermore, 42 species face some degree of threat, two are classified as having deficient data and four have been introduced.

**New information:**

This list constitutes a reference for studies of birds of the Upper Magdalena Valley. It is a necessary tool for conservation plans in this region of the country.

## Introduction

Birds are one of our most well-studied animal groups (Hochkirch et al. 2022); however, at present, there is still a lack of information on the composition of birds in many regions of Colombia and in the face of alarming landscape transformation, pollution and other processes that affect bird communities, it is necessary to develop inventories, complete and accurate lists of avifauna (e.g. Acevedo-Charry 2017, Avendaño et al. 2018, Cordoba and Sierra 2018, Gómez et al. 2022). This information will improve the understanding of the ecology of the communities and their biogeography (e.g. Cohn-Haft et al. 1997, Acevedo-Charry 2017, Avendaño et al. 2018, Palacio et al. 2019, Gómez et al. 2022, Instituto de Investigación de Recursos Biológicos Alexander von Humboldt Programa de Evaluación 2022). In addition, it generates information on the species composition of a specific area, highlighting data on the presence of species of conservation concern (e.g. endemic and threatened), data necessary for the declaration of conservation areas, the development of local management plans for focal species, emblematic species declarations and environmental marketing (Marinho and Beech 2020, Gómez et al. 2022). Furthermore, the filtered, verified and technically supported species lists contribute to reinforce community-based tourism processes and their avitourism routes (Gómez et al. 2022).

Many lists of birds from different regions of Colombia have already been published (e.g. Álvarez 2000, Álvarez et al. 2003, Losada-Prado et al. 2005a, Krabbe et al. 2006, Parra-Hernández et al. 2007, Donegan et al. 2010, Verhelst et al. 2011). However, only a few publications have been compiled for Colombia’s 32 Departments, with lists of birds available for only six Departments (Ayerbe et al. 2008,Calderón et al. 2011, Arbeláez-Cortés et al. 2011, Carrillo-Chica et al. 2018, Chaparro et al. 2018, Cárdenas et al. 2020). The Tolima Department is located on the eastern slopes of the Colombian Central Andes and has been a site of interest for researchers since the beginning of organised ornithology in the region. Notable events date back to the Royal Botanical Expedition led by José Celestino Mutis in the 18^th^ century (1732), which contributed toward recognising and highlighting the great richness of species in this region of Colombia (Córdoba 2009). This research drew the attention of prestigious foreign ornithologists, tempting them to explore Colombia to identify new species. For example, Goudot (French) and Wirt Robinson (US Navy) studied birds in the north of Tolima, as well as in Ibagué (Wirt 1895).

Important studies carried out in the Department include the expedition of the American Museum of Natural History led by Frank Chapman (Chapman 1912, Chapman 1917), the work carried out by Alden Miller in the Upper Magdalena Valley (Miller 1947, Miller 1952), publications by Meyer de Schauensee between 1948 and 1951 (de-Schauensee 1948, de-Schauensee 1949, de-Schauensee 1950, de-Schauensee 1951) and work by the brothers Nicéforo María and Olivares (Olivares 1960, Nicéforo and Olivares 1965, Nicéforo and Olivares 1975, Nicéforo and Olivares 1976a, Nicéforo and Olivares 1976b), all of which provide valuable early records. Since the 2000s, the Zoology Research Group (Grupo de Investigación en Zoología - GIZ) at Tolima University, in association with the Corporación Autónoma del Tolima (CORTOLIMA), have undertaken rapid assessment surveys of local fauna as part of the on-going watershed land-use planning, resulting in many updates to the lists of birds of the Department. As a result of these accumulated efforts, 527 bird species are known from the city of Ibagué and its immediate surroundings (Parra-Hernández et al. 2007). Reinoso et al. (2009) compiled the first regional list of birds, which includes 671 species. However, the latter list lacks references to specimens and other important information, such as localities.

Although Tolima has a lengthy ornithological history stretching back more than 100 years, no complete compendium of its regional avifauna has ever been published. This has hampered efforts to prioritise important species and sub-regions in Tolima that are worth conserving. The present work, thus, aimed to establish an annotated list of bird species found in the Department of Tolima, providing information about the habitat where they are found, altitudinal range, locality, bibliographical source, recording method, and catalogue number for specimens registered in national and foreign museums or significant collections. The voucher and museum number where the specimens are found are referenced in the observation section below.

## Materials and methods

### Study site

The Department of Tolima is located in the centre of Colombia (2°52'59"N; 75°19'59"W and 4°24'18"N; 76°06'23"W) in an altitudinal range between 183 m a.s.l. to a zone of perpetual snow at 5220 m a.s.l. It is mainly located on the eastern slopes of the Central mountain range and a small portion of the western slopes of the Eastern mountain range, being part of the Magdalena River Valley. It covers 23,580 km^2^ that can be divided into three large areas: (i) a mountainous area formed by part of Central Andes with peaks over 5,000 m a.s.l.; (ii) a small portion of the Cordillera Oriental and an area covering the Cordillera Central’s piedmont (premontane area) where the majority of the Department’s human population live and carry out production activities; and (iii) an area covered by the plains of the Upper Magdalena River Valley, rising from 230 to approximately 1,000 m a.s.l. (Instituto Geográfico Agustín Codazzi - IGAC 1996, Fig. 1).

It has differing ecological zones, according to the Holdridge life zone classification scheme: tropical rainforest, tropical dry forest, premontane wet forest, premontane rainforest, lower montane wet forest, lower montane rainforest, montane rainforest, montane rainforest and subalpine desert (paramo) life zones (Andean snowy) (Pomar and Vargas 1985). The coordinates of the sites that have been sampled in Tolima can be seen in Table 1.

The landscape of Tolima has different types of human (anthropogenic) intervention: the lower lying areas with dry forest and tropical wet forest are widely suited for rice and sorghum growing and, to a lesser extent, corn and livestock, while the mountainous areas ranging from 1,000 to 2,000 m a.s.l. are used for growing fruit, coffee and yucca. Extensive areas of the higher sub-paramo and paramo areas are used for livestock grazing and potato growing (Instituto Geográfico Agustín Codazzi - IGAC 2004, Reinoso et al. 2009, Lozano et al. 2011).

### Data collection

In line with the taxonomy and sequence established by Remsen et al. (2020), this work compiles the majority of information available to date concerning the lists of avifauna for the basins of the Toche, Coello, Prado and Amoya Rivers (López et al. 2000, Losada-Prado et al. 2003, Losada-Prado et al. 2005b, Losada-Prado et al. 2005c, Parra-Hernández 2006), the Totare, Anamichu, Lagunillas, Guanabanos and Mendarco Rivers (e.g. Molina-Martinez and Rodríguez (2007), Molina-Martinez et al. (2008), Molina-Martínez et al. (2008), Molina-Martínez (2014), Molina-Martínez et al. (2015)); and partial or total publications regarding the avifauna of several municipalities in Tolima, mainly Ibagué (Corporación Autónoma Regional del Tolima (CORTOLIMA) 1996, Quevedo and Lopéz 2002, Bejarano et al. 2005, Parra-Hernández et al. 2007, Losada-Prado and Molina-Martínez 2011).

This list includes historical bibliographical records for Tolima (Stone 1899, Chapman 1912, Chapman 1917, Miller 1947, Miller 1952), as well as data from several authors who provided records regarding the Department’s species (Dugand 1941, Dugand 1948, Nicéforo 1964, Nicéforo and Olivares 1965, Nicéforo and Olivares 1975, Nicéforo and Olivares 1976a, Nicéforo and Olivares 1976b, Olivares 1973, Hilty and Brown 1986, Quevedo and Lopéz 2002). We also incorporated records provided by the National Museum of Natural History (NMNH - USNM), the Museum of Comparative Zoology (MCZ), the Academy of Natural Sciences Philadelphia (ANSP), the Field Museum of Natural History Chicago (FMNH), the Museum of Vertebrate Zoology Berkeley (MVZ), the Instituto Alexander von Humboldt (SIB) and the Museo de La Salle (MSL). Museum collection databases were independently reviewed online or the information was requested directly from the museum. In addition, we made revisions and confirmations of some species at the Colección Zoológica de La Universidad del Tolima - Ornitología (CZUT-OR) and Instituto Nacional de Ciencias (ICN) at the Universidad Nacional de Colombia.

The BioMap Project / Darwin Database (2018), eBird (Cornell Lab of Ornithology and National Audubon Society 2023) and Xeno-canto (2020) databases were also consulted for every species and the catalogue numbers are included in this list. We also included our occasional records collected over more than 15 years. All bibliographic references and databases used are noted in Table 2. Abbreviations have been assigned and are used below in the species list.

For better understanding of the species spatial and altitudinal distributions, the municipalities where grouped into four areas or sub-regions, namely the south-eastern, northern, south-western and central areas of the Department (Table 3).

An indication is given of the altitudinal ranges of the species found in Tolima (minimum and maximum elevations), as well as the habitat and/or life zone where they was observed, captured, collected or sound-recorded. The bibliographical source and pertinent notes regarding the species’ regional status are also included. Relevant details, such as the type of record, endemic or near-endemic (Stiles 1998, Chaparro et al. 2013, Ayerbe 2018), migratory (Naranjo et al. 2012, Avendaño et al. 2017), extralimital (Hilty and Brown 1986), introduced (Restall et al. 2006), reintroduced or threatened species (International Union for Conservation of Nature - IUCN 2020) are also indicated.

The distribution section indicates the municipalities in Tolima where each species was registered. The municipalities are abbreviated as follows: (A-G) Armero-Guayabal, (Al) Alvarado, (Am) Ambalema, (An) Anzoátegui, (At) Ataco, (Cb) Casabianca, (Cha) Chaparral, (Cj) Cajamarca, (Cm) Carmen de Apicala, (Co) Coyaima, (Cu) Cunday, (Do) Dolores, (Es) Espinal, (Fa) Falan, (Fl) Flandes, (Fr) Fresno, (Gu) Guamo, (Gy) Gualanday, (He) Herveo, (Ho) Honda, (Ib) Ibagué, (Ic) Icononzo, (Le) Lérida, (Li) Líbano, (Ma) Mariquita, (Me) Melgar, (Mu) Murillo, (Na) Natagaima, (Or) Ortega, (Pie) Piedras, (Pl) Planadas, (Pr) Prado, (Pu) Purificación, (Rb) Rio Blanco, (Rc) Roncesvalles, (Rv) Rovira, (Sa) Saldaña, (Si) Santa Isabel, (Sl) San Luis, (SAN) San Antonio (Su) Suarez, (Ve) Venadillo, (Vh) Villahermosa and (Vi) Villarrica. The notes section indicates the type of record, the voucher number in the case of museum specimens or collections, endemism, category of threat and whether the species is migratory or introduced. The institutions are abbreviated as follows: MNHNP = Museum National d’Histoire Naturelle Paris, DNHM = Delaware Natural History Museum, WFVZ = Western Foundation of Vertebrate Zoology, FMNH = The Field Museum of Natural History, CUMV = Cornell University Museum of Vertebrates, NHMA = Natural History Museum of Los Angeles County, NMNH = Smithsonian National Museum of Natural History, AMNH = American Museum of Natural History, MCZ = Harvard Museum of Comparative Zoology, MLS = Museo La Salle, SIB = Colección Humboldt IAvH, MPUJ = Museo Pontificie Universidad Javeriana, CZUTOR = Colección Zologica de La Universidad del Tolima - referencia Ornitología, MHN-ICN = Museo de Historia Natural, Instituto de Ciencias Naturales Universidad Nacional de Colombia, and IVI = Instituto Vallecaucano de Investigación. The type of record is indicated using the following conventions: (v) visual, (au) auditory, (ca) capture, (co) collection and (ph) photographic. The distributions are indicated using the following conventions (Chaparro et al. 2013): (E) endemic, (CE) almost endemic, (M) migratory, (EL) extralimital, (INT) introduced, (RINT) reintroduced and (F) probable translocation and/or flight from a nearby area. The threat category is indicated as follows: (CR) critically endangered, (EN) endangered, (VU) vulnerable, (NT) nearly threatened, (EX) considered to be extinct at the national or departmental level and (WD) widely distributed throughout the Department. These and other data, such as altitudinal range (m) and habitat, are provided in Suppl. material 1.

### Data Analysis

In order to determine the variation in species composition amongst municipalities, we calculated the Jaccard Similarity Index. Dimensionality was reduced using non-metric multidimensional scaling (NMDS), based on a matrix of presence-absence species data. Contrasts in species composition were displayed using two-dimensional images (Legendre and Legendre 1998), with points designating the localities where each species was found. We used the Jaccard Index and NMDS to reduce the problems generated by differences in sample efforts across localities (Ludwing and Reynolds 1998). The non-parametric species richness estimators Chao 2 and ICE were calculated using EstimateS 9.1 (Colwell 2013) to determine the data representativeness from the richness (for the data used, see Suppl. material 2).

## Checklists

### List of birds from Tolima department, Colombia

#### Nothocercus julius

(Bonaparte, 1854)

0B9DB04A-633A-53E3-B56B-78DE10BBF8C5

##### Distribution

Chaparral, Cajamarca, Ibagué, Planadas, Roncesvalles

##### Notes

(v; au XC127813, ML131110861; co ANSP 154806)

#### Nothocercus bonapartei

(Gray, 1867)

46501698-1F93-5EDE-A466-9A339626F1F2

##### Distribution

Ibagué, Libano, Melgar, Planadas

##### Notes

(au ML 376862821; co USNM 372295).

#### Tinamus major

(Gmelin, 1789)

90DE4272-24B6-5D9A-A6D5-DA417285A723

##### Distribution

Armero -Guayabal, Falan, Ibagué, Piedras.

##### Notes

(au) New record - on 11 August 2015, we casually listened to the singing of this species in a wooded area of the municipality of Falan, in the area known as Ciudad Perdida (900 m), which was identified during several field trips to this area. A specimen was heard vocalising at Quebrada La Honda (Ibagué), near Hacienda el Zorro, on 21 July 2017. On 7 September 2017, an individual was heard in the RN La Primavera, municipality of Ibagué (pers. commun., D. Carantón), at approximately 1,100 m a.s.l.; in the municipality of Piedras, in La Hacienda Gascoña (pers. commun. H. Cruz); and in Armero - Guayabal in the Granja de Armero (pers. commun. M. Moreno).

#### Crypturellus soui

(Hermann, 1783)

5FF3666C-C5CD-5BFC-9CE5-27820B5397F0

##### Distribution

Armero-Guayabal, Alvarado, Alpujarra, Cunday, Dolores, Ibagué, Falan, Fresno, Lerida, Mariquita, Melgar, Planadas, Prado, San Luis, Suárez, Villarrica

##### Notes

(v; au XC424036; co ICN MHN 33401; ph ML123194721).

#### Crypturellus erythropus

(von Pelzeln, 1863)

3D534ED1-240C-5668-88DA-AEE873CEDEA4

##### Distribution

Honda, Mariquita, Armero

##### Notes

(v; co; ph ML616044311; au ML616044279). There is a historical record of an specimen from *C. saltuarius* collected by Fray Diego García in Chiminá Feligresia (Mariquita) (López 2002).

#### Chauna chavaria

(Linnaeus, 1766)

9AE1BC85-82DF-500F-8642-8E627B678F54

##### Distribution

Armero-Guayabal, Honda, Melgar

##### Notes

(v; ph https://www.inaturalist.org/photos/64474746).

#### Dendrocygna viduata

(Linnaeus, 1766)

22DAD939-D692-5275-9220-5CEBB9936405

##### Distribution

Armero - Guayabal, Ambalema, Coyaima, Honda, Ibagué, Lerida, Mariquita, Melgar, Natagaima, Piedras, Prado, Purificación, Saldaña, Venadillo

##### Notes

(v; ph) EL. New report - ten individuals were observed and photographed during November 2004 in the lagoon of Coya between Purificación and Coyaima, registered by Parra and Rojas (2011). This record corresponds to the first verifiable record of the presence of this species in the valley Magdalena later in the municipality of Ibagué by W. Yate who made an observation in 2007. D. Juez made an observation on 21 January 2018 in Mariquita. On 15 November 2008, K. Certuche recorded 12 individuals in Venadillo; one individual was recorded in Hacienda Pajonales – Ambalema on 12 May 2016 (Y. Tolosa); and two individuals were recorded in Ibagué on 12 November 2017.

#### Dendrocygna autumnalis

(Linnaeus, 1758)

21B70B20-31B4-5189-99D3-34D1DF30B793

##### Distribution

Alvarado, Armero-Guayabal, Coyaima, Honda, Ibagué, Libano, Mariquita, Melgar, Prado, Purificación, Suárez, Venadillo

##### Notes

(v; au XC156277; ph ML118594121)

#### Oressochen jubatus

(Gmelin, 1789)

D0038642-E05F-591C-9090-D57F7300B6C8

##### Distribution

Ibagué

##### Notes

(v; ph ML118593401) F. New record - a couple of individuals were observed in Aparco, a peri-urban area of the municipality of Ibagué, during the months of September and October 2018. The birds were initially identified by H. Arias and later by us and other observers (Fig. 4c). The individuals did not belong to any resident of the area; however, we do not rule out that they are the product of an escape or that they are migrants.

#### Cairina moschata

(Linnaeus, 1758)

694880B8-93DC-50FF-9FD2-2F138ED00018

##### Distribution

Ibagué, Piedras, San Luis

##### Notes

(ph ML57130461) Rint. New record - on 8 July 2006, five individuals were observed by the Grupo de observación de Aves (GOAT) at Hacienda Gascoña (Piedras). On 11 July 2007, an individual was observed in the lagoon complex of Salado (Ibagué). On 7 June 2017, we photographed two individuals in Aparco, located towards the peri-urban area of the municipality of Ibagué. These records correspond to repopulation of this species, as its numbers had declined in past decades (Fig. 4b).

#### Merganetta armata

Gould, 1841

91C3B8C8-ECD0-549B-ABEA-7FE400DD0DA4

##### Distribution

Cajamarca, Fresno, Ibagué, Libano, Murillo, Planadas, Río Blanco, Roncesvalles, Villahermosa

##### Notes

(v; co AMHN 111392; ph ML79620011)

#### Spatula discors

(Linnaeus, 1766)

6A7893FB-9066-5F31-9F5B-05C9DA95F2B0

##### Distribution

Armero-Guayabal, Coyaima, Ibagué, Libano, Murillo, Piedras, Purificación

##### Notes

(v; ph ML24395711) Mb

#### Spatula clypeata

(Linnaeus, 1758)

51E3D261-8131-5D53-AA52-60B2C3F6A8A4

##### Distribution

Ibagué

##### Notes

(v, ph) Mb New record - an individual was registered and recorded in the lagoon complex of Picaleña (Ibagué) on 13 May 2017 by D. Carantón and team-mates during the Global Big Day (GBD). The record corresponds to a sporadic case of this migratory species being observed perched and flying over the lagoon.

#### Anas platyrhynchos

(Linnaeus, 1758)

EA0AC7ED-ACE0-50D8-B350-CA5FB136CF53

##### Distribution

Ibagué

##### Notes

(v; ph ML123176141) INT. New record - two individuals were photographed on 7 May 2017 in the lagoon of Aparco, on the outskirts of the Municipality of Ibagué. The individuals correspond to feral species of neighbouring farms observed around some lagoons in this zone.

#### Anas andium

(Sclater & Salvin, 1873)

B9BF7609-042A-5D9D-96BB-EC26FE89CFA9

##### Distribution

Anzoátegui, Cajamarca, Ibagué, Murillo, Planadas, Roncesvalles, Santa Isabel

##### Notes

(v; co SIB 6790; ph ML123178761)

#### Aythia affinis

(Eyton, 1838)

8261C6B8-59A8-550E-803B-C9AC403C4C4B

##### Distribution

Ibagué, Prado

##### Notes

(v; ph ML68427311) M New record. Initially, five individuals were observed on 11 February 2011 and then a group of eight was photographed on 10 January 2013, at Hacienda el Escobal (900 m a.s.l.). In the Municipality of Ibagué Initially, five individuals were observed on 11 February 2011. Later, a group of eight was photographed on 10 January 2013 at Hacienda El Escobal (900 m a.s.l.) in the Municipality of Ibagué (Fig. 4a). The individuals were observed on the water mirror in groups comprising females and non-reproductive males, located with native species of ducks (Anatidae) and cormorants. The records coincide with other observations made on those days in Bogotá (Colombia), suggesting an unusual migration route of this species during some seasons. These records are the first for this migratory species in the Upper Magdalena Valley. These observations, like those for other migratory ducks that arrive sporadically in the Municipality of Ibagué, suggest an alternative route for this species.

#### Nomonyx dominicus

(Linnaeus, 1766)

20A9FC4A-5564-56FE-98EE-82F91D591B96

##### Distribution

Ibagué, Coyaima, Armero - Guayabal, Murillo, Planadas, Purificación

##### Notes

(v; co MHN-ICN 562; ph)

#### Oxyura jamaicensis

(Gmelin, 1789)

671986C7-AB4D-59B1-BD32-B8C6B0567D02

##### Distribution

Anzoátegui, Cajamarca, Ibagué, Herveo, Murillo

##### Notes

(v; ph ML106506211). New record - different individuals of this species were recorded in Tolima, especially in the upper area of the Department. This species was registered regularly in Murillo (pers. commun., Y. Rincón). On 11 September 2016, D. Carantón registered a group of 10 individuals (pers. commun., D. Carantón) in Anzoátegui.

#### Chamaepetes goudotii

(Lesson, 1828)

955F80A8-5BF0-5A18-A4C4-A36CF0819771

##### Distribution

Anzoátegui, Cajamarca, Honda, Ibagué, Falan, Libano, Mariquita, Planadas, Rio Blanco, San Antonio, Santa Isabel, Villarrica

##### Notes

(v; au; co CZUTOR 0177; ph ML115212621)

#### Penelope montagnii

(Bonaparte, 1856)

1DB2D859-E89A-563A-9616-D4899EB58E47

##### Distribution

Cajamarca, Fresno, Ibagué, Murillo, Planadas, Roncesvalles, Santa Isabel

##### Notes

(v; co SIB 126311; ph ML45290981)

#### Aburria aburri

(Lesson, 1828)

E5FA486D-0281-514B-A43F-0D90E946DF79

##### Distribution

Anzoátegui, Ambalema, Ibagué, Murillo, Planadas, Roncesvalles

##### Notes

(v; au) NT. A few records of this species in Tolima were made by chance in Roncesvalles in 2005 by W. Yate. Similarly, it was observed in the Reserva Natural Bella vista (Ibagué) in May 2007 (Molina-Martínez 2014). M. Olaya registered this species in the municipality of Planadas on 5 May 2018 (pers. commun., M. Olaya). Although this species was observed in high areas of the Department, it may go unnoticed and the few records correspond to casual or auditory observations.

#### Ortalis columbiana

Hellmayr, 1906

C1950175-151B-54DB-83E8-96A54E0FB46F

##### Distribution

Alpujarra, Armero-Guayabal, Anzoátegui, Cajamarca, Casabianca, Carmen de Apicalá, Chaparral, Coello, Coyaima, Cunday, Dolores, Espinal, Falan, Fresno, Gualanday, Herveo, Honda, Ibagué, Iconzo, Lerida, Libano, Melgar, Mariquita, Natagaima, Planadas, Prado, Purificación, Rio Blanco, Rovira, Roncesvalles, San Antonio, San Luis, Suárez, Valle de San Juan, Venadillo, Villarrica, Villahermosa

##### Notes

(v; au XC424477; co MLS 574; ph ML123182221). This endemic species was registered in almost all of the lower areas of the Department, occurring in groups of 4 - 18 individuals in dry and premontane forests.

#### Crax alberti

Fraser, 1852

13E5AFF7-C81E-5B01-8713-E2CCD9B59C4D

##### Distribution

Honda

##### Notes

(co MLS 525) CR. Historical record of a specimen collected by Kerr in Honda. This species is probably extinct in the region.

#### Colinus cristatus

(Linnaeus, 1766)

8CBA4AAE-6F1D-5B63-A689-C0B7A13AFE22

##### Distribution

Alpujarra, Armero-Guayabal, Alvarado, Ataco, Carmen de Apicala, Casabianca, Cajamarca, Coyaima, Casabianca, Cunday, Espinal, Guamo, Falan, Flandes, Fresno, Herveo, Honda, Ibagué, Icononzo, Lerida, Mariquita, Melgar, Murillo, Natagaima, Palocabildo, Piedras, Planadas, Prado, Purificación, Rio Blanco, Roncesvalles, San Antonio, San Luis, Suarez, Venadillo, Villarrica, Villahermosa

##### Notes

(v; au; ph ML72669811; co MHN-ICN 4397, MLS 595)

#### Odontophorus hyperythrus

Gould, 1858

CF295817-CF2B-58B7-9ACD-16724452D9D5

##### Distribution

Casabianca, Falan, Ibagué

##### Notes

(v; au XC96070, ML98610951) E NT

#### Phoenicopterus ruber

Linnaeus, 1758

6350D05C-BF60-5FCB-BE8C-3DBEE76D44AC

##### Distribution

Ambalema, Ibagué

##### Notes

(v; ph) INT. New record - in Ibagué, an individual was observed in June 2015 over the lagoon complex of Picaleña. Recently, J. Sanabria photographed a colourful individual in the same locality. These sightings were probably the result of escapes or a consequence of accidental movements (Parra-Hernández et al. 2015, Fig. 4dFig. 4e).

#### Tachybaptus dominicus

(Linnaeus, 1766)

C3A9C5F4-F834-5040-9E4F-85542957389F

##### Distribution

Ibagué, Venadillo

##### Notes

(v; ph ML123896681)

#### Podilymbus podiceps

(Linnaeus, 1758)

1EFA2A97-3963-504D-8CDE-B359C0862876

##### Distribution

Armero-Guayabal, Ibagué, Libano, Prado

##### Notes

(v; co CZUTOR 0286; ph ML123183561)

#### Podiceps occipitalis

Garnot, 1826

7ADC6455-25DE-51D4-9B36-08F8405AAA88

##### Distribution

Roncesvalles, San Antonio

##### Notes

(v; ph) New record - these records correspond to the first observations of this species in the Department of Tolima made in the Municipality of Roncesvalles. Recently, W. Yate photographed three individuals which were observed on 7 April 2005 at Laguna Los Patos at 3200 m and an individual was observed on 9 January 2018 at Laguna Carrizales at 3600 m on the Cucuanita path (Fig. 4e). A photographic record was made of nesting on the border with the Department of Quindio, in the lagoon of Muñeco (ML 106896191).

#### Columba livia

Gmelin, 1789

7B63C9D0-6E43-5DDD-864F-3C898AAB6847

##### Distribution

Alpujarra, Alvarado, Anzoategui, Ambalema, Armero-Guayabal, Coyaima, Casabianca, Honda, Ibagué, Espinal, Falan, Fresno, Libano, Mariquita, Murillo, Piedras, Planadas, Prado, Saldaña, San Antonio, San Luis, Roncesvalles, Valle de San Juan, Venadillo, Villahermosa

##### Notes

(v; ph ML123183941) INT

#### Patagioenas speciosa

(Gmelin, 1789)

168DCB55-C0A9-5BD8-8896-9A2B3EB35655

##### Distribution

Honda, San Luis

##### Notes

(v; co AMHN 94801)

#### Patagioenas fasciata

(Say, 1823)

9F0F5B1A-5EA8-5AA1-BE05-4210C56F06E3

##### Distribution

Anzoátegui, Ataco, Casabianca, Cajamarca, Herveo, Falan, Ibagué, Murillo, Planadas, Roncesvalles, San Antonio, Santa Isabel, Rioblanco, Roncesvalles, Villarrica, Villahermosa

##### Notes

(v; au; ph ML123189671)

#### Patagioenas cayennensis

(Bonnaterre, 1792)

64DFC462-359D-5078-86C5-8C52249A4AA9

##### Distribution

Alvarado, Ambalema, Armero-Guayabal, Ataco, Falan, Honda, Ibagué, Guamo, Mariquita, Natagaima, Planadas, San Antonio, San Luis, Villarrica

##### Notes

(v; au co MHN-ICN 4382; ph ML123915061)

#### Patagioenas plumbea

(Vieillot, 1818)

047FFEC1-C9AE-5AB8-856C-0E767A754626

##### Distribution

Chaparral, Falan, Guamo, Libano, Melgar, Villarrica

##### Notes

(co CZUT-0372). New record - an uncommon species in Tolima, it was observed in Melgar's Victoria Reserve on 6 August 2016, captured in Villarica, in the Galilea Forest, on 20 August 2004 (Parra-Hernández 2006) and photographed on 5 May 2019. There are also records from the Combeima River Canyon by P. Flores on 19 August 2017 and from the Municipality of San Luis (Guamo) on 5 May 2018 by D. Juez.

#### Patagioenas subvinacea

(Lawrence, 1868)

DFB0D40C-3A10-5CED-A193-8AD3EBCBB7B6

##### Distribution

Casabianca, Cunday, Falan, Fresno, Herveo, Ibagué, Libano, Murillo, Planadas, San Antonio, San Luis, Villarrica

##### Notes

(v; au; ph ML83880261) This species is occasionally observed in Tolima. We recorded it in Laguna Venta Quemada - Alvarado on 16 November 2004. Two individuals were observed in Complejo Lagunar Piedras (Gascoña) on 17 January 2017 and in Las Catorce on 24 August 2004.

#### Streptopelia decaocto

(Frivaldszky, 1838)

2409DB27-168D-57A3-BD2F-5559D98107D0

##### Distribution

Ibagué, Mariquita

##### Notes

(v; ph ML 218086841)

#### Geotrygon montana

(Linnaeus, 1758)

4B250C05-9788-51A4-816C-D95813F264C2

##### Distribution

Ibagué, Falan, Fresno, Honda, Lerida, Ortega, Planadas, San Antonio, San Luís, Villarrica

##### Notes

(ca; co MHN-ICN 9462; ph ML123885661)

#### Leptotila verreauxi

(Bonaparte, 1855)

8881FB1F-4557-5437-B941-F9E11241EFE8

##### Distribution

Alpujarra, Alvarado, Ambalema, Armero-Guayabal, Ambalema, Carmen de Apicala, Cunday, Espinal, Falan, Fresno, Guamo, Herveo, Honda, Ibagué, Icononzo, Lerida, Natagaima, Mariquita, Melgar, Ortega, Planadas, Piedras, Prado, Purificación, Rovira, San Luis, Suárez, Venadillo, Villarrica, San Antonio

##### Notes

(v; au XC424488; ca; co MLS 959, CZUTOR 90; ph ML99189531) WD

#### Leptotila conoveri

Bond & Meyer de Schauensee, 1943

49EB7D9F-4345-545E-B9E3-95D5BD3B5214

##### Distribution

Ataco, Casabianca, Falan, Fresno, Herveo, Ibagué, Libano, Planadas, Roncesvalles, San Antonio, Villahermosa, Villarrica

##### Notes

(v; ca; co CZUTOR 524; au XC425868; ph ML79620131) E EN. This pigeon, endemic to Colombia, is mainly recorded in Huila and Tolima, but also in Cauca. This species is rare or moderately common in some coffee zones (Fig. 8a).

#### Leptotila plumbeiceps

(Sclater & Salvin, 1868)

C92C4C52-B8A3-5722-88B8-763BD466E2FA

##### Distribution

Ibagué

##### Notes

(au ML157053441)

#### Zentrygon frenata

(Tschudi, 1843)

0323F296-E748-571A-AEF6-2344576F9D88

##### Distribution

Herveo, Ibagué, Falan, Planadas, San Antonio, Santa Isabel

##### Notes

(v; ca; co ANSP 154883)

#### Zentrygon linearis

(Prévost, 1843)

D86024FD-DF36-5C5C-80D7-DDA091A7D7C6

##### Distribution

Herveo, Ibagué, Falan, Planadas, San Antonio

##### Notes

(v; ph ML75395481) This bird is difficult to observe. A few records were made in Ibagué in the Vereda Chembe on 26 July 2015 and in Juntas (Combeima River Canyon) in January 2015. J. Luna also registered it in Juntas (record from eBird) on 13 November 2017 and N. Mølgaard Thomsen photographed it in the same area.

#### Zenaida auriculata

(Des Murs, 1847)

A27BE606-875B-579E-941F-D714F2FD2A4F

##### Distribution

Alpujarra, Alvarado, Anzoategui, Armero-Guayabal, Ataco, Cajamarca, Casabianca, Chaparral, Coyaima, Coello, Cunday, Dolores, Espinal, Falan, Flandes, Herveo, Honda, Ibagué, Lerida, Libano, Murillo, Mariquita, Palocabildo, Piedras, Planadas Prado, Rio Blanco, Roncesvalles, Saldaña, San Antonio, San Luis, Suárez, Valle de San Juan, Venadillo, Villarricallahermosa, Villarrica

##### Notes

(v; ca; co CZUTOR 0245; ph ML123193911; au XC420996) WD

#### Claravis pretiosa

(Ferrari-Perez, 1886)

C21749E4-9003-5599-B548-A8AEE09570FC

##### Distribution

Armero-Guayabal, Dolores, Ibagué, Icononzo, Falan, Fresno, Libano, Melgar, Natagaima, Planadas, Prado, Purificación, Suárez, San Luis, Valle de San Juan, Venadillo,

##### Notes

(v; au XC386627; co CZUTOR 086; ph ML10922702)

#### Columbina passerina

(Linnaeus, 1758)

2E367802-8829-51B0-8EA7-FE9F9AB68D38

##### Distribution

Armero-Guayabal, Alvarado, Carmen de Apicala, Chaparral, Coyaima, Espinal, Falan, Fresno, Honda, Ibagué, Icononzo, Mariquita, Melgar, Prado, Purificación, San Luis, Villarrica

##### Notes

(v; co MLS 94, CZUTOR 89; ph ML123218521)

#### Columbina minuta

(Linnaeus, 1766)

535246F9-095D-575E-9438-862C5CE282F9

##### Distribution

Armero - Guayabal, Coello, Falan, Fresno, Ibagué, Mariquita, Prado, Purificación, San Luis, Venadillo

##### Notes

(au; ML111645151; co MNH 70814; ph ML111645041 v) This species went unnoticed for some years in Tolima and was initially registered in the Municipality of Prado in August 2004 (Parra-Hernández et al. 2015). It has now been observed in different areas of the Department, especially below 1200 m a.s.l. in the municipalities of Mariquita, Coello, Girardot, Falan, Purificación, San Luis and Ibagué (Fig. 7f).

#### Columbina talpacoti

(Temminck, 1810)

B60D5B11-757F-5F66-B622-3F9A59ED3736

##### Distribution

Alpujarra, Alvarado, Armero-Guayabal, Anzoátegui, Ataco Ambalema, Cajamarca, Casabianca, Chaparral, Carmen de Apicala, Coyaima, Cunday, Dolores, Falan, Fresno, Guamo, Honda, Ibagué, Icononzo, Lerida, Libano, Natagaima, Mariquita, Ortega, Planadas, Prado, Purificación, Rio Blanco, Rovira, Saldaña, San Antonio, San Luis, Valle de San Juan, Venadillo, Villarricallahermosa, Villarrica

##### Notes

(v; au XC420993 ca; co; MLS 931.CZUTOR 92; ph ML72670051) WD

#### Crotophaga major

Gmelin, 1788

FE756AB6-FCA5-5797-8BAA-4B1C0277F9BF

##### Distribution

Armero-Guayabal, Alvarado, Ambalema, Ataco, Carmen de Apicala, Casabianca, Chaparral, Coyaima, Cunday, Espinal, Falan, Fresno, Guamo, Honda, Ibagué, Lerida, Libano, Mariquita, Melgar, Murillo, Natagaima, Ortega, Piedras, Prado, Purificación, Saldaña, San Luis, Venadillo

##### Notes

(v; au XC386464; co MLS 1291, MHN-ICN 12894; ph ML123196651)

#### Crotophaga ani

Linnaeus, 1758

4B149A96-4F35-52F5-9714-92A638706755

##### Distribution

Alpujarra, Alvarado, Armero-Guayabal, Alvarado, Anzoátegui, Ataco Ambalema, Cajamarca, Casabianca, Chaparral, Carmen de Apicala, Coyaima, Cunday, Dolores, Falan, Fresno, Guamo, Honda, Ibagué, Icononzo, Lerida, Libano, Natagaima, Mariquita, Ortega, Planadas, Prado, Purificación, Rio Blanco, Rovira, Saldaña, San Antonio, San Luis, Valle de San Juan, Venadillo, Villarricallahermosa, Villarrica

##### Notes

(v; au XC386463; co CZUTOR 822; ph ML123200521) WD

#### Crotophaga sulcirostris

Swainson, 1827

705C8CC5-7FBD-59EB-9A71-C6E41C676346

##### Distribution

Armero-Guayabal, Chicoral, Coyaima, Cunday, Espinal, Falan, Honda, Ibagué, Mariquita, Melgar, Prado, Purificación, Rio Blanco, Saldaña, San Luis, Venadillo, Villarrica

##### Notes

(v; au; co MLS 1302, CZUTOR 70; ph ML123200911)

#### Tapera naevia

(Linnaeus, 1766)

D26120D9-4717-5D94-A625-50B509CFF655

##### Distribution

Alpujarra, Alvarado, Anzoategui, Casabianca, Cajamarca, Chaparral, Coello, Coyaima, Cunday, Dolores, Espinal, Fresno, Guamo, Herveo, Honda, Ibagué, Falan, Lerida, Libano, Mariquita, Melgar, Murillo, Natagaima, Palocabildo, Planadas, Piedras, Prado, Purificación, Rio Blanco, Saldaña, San Antonio, San Luis, Santa Isabel, Valle San Juan, Venadillo, Villahermosa, Villarrica

##### Notes

(v; au XC157753; co MLS 1306; ph ML123201491)

#### Dromococcyx phasianellus

(Spix, 1824)

EE0E164E-4668-5433-B49A-4ED1B4485094

##### Distribution

Alvarado, Guamo, Ibagué, Piedras, San Luis, Venadillo

##### Notes

(v; au XC20387; co mls 1280). New record - this species was registered for the first time in Tolima by H. Arias and J. Sanabria at Hacienda el Chaco (Piedras) on 27 February 2008 and subsequently registered in several surrounding areas of dry forest. It is trackable in riverine forest and small fragments of tropical dry forest near water sources, as well as near bamboo. Although it is difficult to track, it can be identified by its vocalisation. It has been observed within the limits of Ibagué in Alvarado and Piedras.

#### Coccycua minuta

(Vieillot, 1817)

3D614449-02AA-5A7C-8B9E-C3DD2747254B

##### Distribution

Espinal, Ibagué, Icononzo, Libano, Melgar, Piedras, Planadas, Prado, San Luis

##### Notes

(v; au; co MLS 1281, CZUTOR 0894; ph ML26340791)

#### Coccycua pumila

(Strickland, 1852)

CA4D013B-AD07-5055-97B9-AD17AB45A75C

##### Distribution

Ambalema, Espinal, Falan, Guamo, Ibagué, Libano, Purificación, San Luis, Villarrica

##### Notes

(v; co MLS 3255; ph ML123202821)

#### Piaya cayana

(Linnaeus, 1766)

B2D034B6-2D42-5108-99E9-E1C3464D7DCF

##### Distribution

Armero-Guayabal, Carmen de Apicala, Chaparral, Cunday, Dolores, Espinal, Falan, Fresno, Guamo, Gualanday, Herveo, Honda, Ibagué, Lerida, Libano, Mariquita, Planadas, Rio Blanco, San Antonio, Santa Isabel, San Luis, Venadillo, Villahermosa, Villarrica

##### Notes

(v; au; co MLS 1258; ph ML123204751)

#### Coccyzus melacoryphus

Vieillot, 1817

FEC0A00B-0DBE-5902-8CDF-32C21DEB8D18

##### Distribution

Armero-Guayabal, Espinal, Falan, Guamo, Ibagué, Libano, Purificación, San Antonio, San Luis, Villarrica

##### Notes

(v; co MLS 3263, CZUT OR1067; ph ML101854131)

#### Coccyzus americanus

(Linnaeus, 1758)

30F83E85-BC6C-5D6F-99FD-5B14AC69F999

##### Distribution

Armero-Guayabal, Coyaima, Espinal, Falan, Fresno, Ibagué, Melgar

##### Notes

(v; co CZUTOR 0363; ph ML83847861) M

#### Coccyzus erythropthalmus

(Wilson, 1811)

D76D07A5-FEEA-5E99-844E-6E15B5951C50

##### Distribution

Armero-Guayabal, Espinal, Ibagué, Libano

##### Notes

(v; co ANSP 154810; ph ML99174331)

#### Steatornis caripensis

Humboldt, 1817

74CAB041-5ECC-54F2-B4C5-A485B85DFA3A

##### Distribution

Casabianca, Cajamarca, Chaparral, Coyaima, Cunday, Herveo, Ibagué, Icononzo, Murillo, Roncesvalles

##### Notes

(v; ca; co CZUTOR 211; au ML82127)

#### Nyctibius griseus

(Gmelin, 1789)

276A5143-9276-5F3D-9E98-2A3E65FA1499

##### Distribution

Alvarado, Armero-Guayabal, Ataco, Falan, Fresno, Herveo, Honda, Ibagué, Lerida, Libano, Mariquita, Melgar, Murillo, Planadas, Prado, San Antonio, San Luis, Venadillo, Villarrica

##### Notes

(v; au; co CZUTOR 0212; ph ML105628231)

#### Chordeiles nacunda

(Vieillot, 1817)

1EF85D46-1324-5A2E-8B57-525C5A690D2A

##### Distribution

Coyaima, Ibagué, Guamo, Honda, Natagaima, Mariquita, San Luis, Venadillo

##### Notes

(v; co MVZ 93791; ph)

#### Chordeiles acutipennis

(Hermann, 1783)

9A2C31BA-0E37-5C82-B128-BB5C8E7E4277

##### Distribution

Alvarado, Armero-Guayabal, Coello, Coyaima, Honda, Ibagué, Falan, Fresno, Mariquita, Melgar, Piedras, Prado, Saldaña

##### Notes

(v; co MLS 8272)

#### Chordeiles minor

(Forster, 1771)

EB29CF82-A07E-5D8B-8210-081682F81582

##### Distribution

Armero - Guayabal, Espinal, Fresno, Guamo, Herveo, Ibagué, Mariquita, Piedras, Venadillo

##### Notes

(v; co MLS 8326; ph ML123213631)

#### Lurocalis rufiventris

Taczanowski, 1884

6FD23E10-AC47-5D5E-88CF-61D0E95CA41A

##### Distribution

Cajamarca, Casabianca, Ibagué, Rio Blanco

##### Notes

(v; au XC518033)

#### Nyctidromus albicollis

(Gmelin, 1789)

117A8296-1D03-5E94-8171-F6A3B568B51A

##### Distribution

Alvarado, Armero - Guayabal, Ataco, Cajamarca, Carmen de Apicala, Chaparral, Coello, Espinal, Honda, Icononzo, Ibagué, Libano, Mariquita, Melgar, Prado, Roncesvalles, Rovira, Sladaña, San Antonio, San Luis, Villarrica

##### Notes

(v; ca; au-XC96383; co MLS 8327, CZUTOR 209; ph ML36497411)

#### Uropsalis segmentata

(Cassin, 1849)

8586D60A-E8AB-5FF9-A926-68BFC09A9BF8

##### Distribution

Ibagué, Cajamarca

##### Notes

(v; ph) Some recent records were made in Ibagué, Silencio on 23 July 2013 (W. Figueroa photographed ML 370281651, ML 370281661, ML 370281671) and Toche at Finca La Leona by M. De Freitas on 2 August 2018 and Salento-Toche Road by C. Sanchéz on 30 August 2019.

#### Uropsalis lyra

(Bonaparte, 1850)

3B2A3C36-F602-51A1-B1F5-F6F5F9B7CA2D

##### Distribution

Cajamarca, Ibagué, Roncesvalles

##### Notes

(v; co ANSP 154700; ph ML51940921)

#### Hydropsalis cayennensis

(Gmelin, 1789)

24C2E01C-5907-5F15-9B1E-9FEF00BD5515

##### Distribution

Armero-Guayabal, Honda, Ibagué, Melgar, Piedras, Planadas

##### Notes

(ca; co NHMA 73169; ph)

#### Systellura longirostris

(Bonaparte, 1825)

3C5E54D1-0EB5-5A6E-A1DE-7AA5413D7772

##### Distribution

Anzoátegui, Cajamarca, Falan, Herveo, Ibagué, Libano, Murillo, Rio Blanco, Roncesvalles, San Antonio, Santa Isabel.

##### Notes

(v; au; co MHN-ICN 27016, CZUTOR 0657)

#### Antrostomus carolinensis

(Gmelin, 1789)

724AEC4A-51FC-5EB6-A19B-ED467944A666

##### Distribution

Chaparral, Ibagué, Villahermosa

##### Notes

(co MHN-ICN 4440; ph ML83559451) ICN

#### Cypseloides cherriei

Ridway, 1893

13B9FC3B-DBB1-5961-B3D5-A7DB684A8347

##### Distribution

Ibagué, Libano

##### Notes

(v; co CZUTOR 0436; ph) DD EL. Ibagué, Tolima. coordinates; 4°25'58"N; 75°13'01"W. altitude 1250 m a.s.l.; date 4 August 2006, Collector. RM. Parra. CZUTOR 0436. This unusual species in the Department was observed by chance for the first time on 4 August 2006 in the Municipality of Ibagué (Parra-Hernández et al. 2008) and later in Ibagué on 7 June 2015 (Fig. 8e).

#### Streptoprocne rutila

(Vieillot, 1817)

32FCEEBE-DFA8-5676-99DE-B519E9BF5AA4

##### Distribution

Alpujarra, Anzoátegui, Casabianca, Cajamarca, Chaparral, Falan, Fresno, Herveo, Ibagué, Libano, Mariquita, Murillo, Palocabildo, Piedras, Planadas, Prado, Roncesvalles, Rio Blanco, San Antonio Villarrica

##### Notes

(v; co MLS 10164, CZUTOR 0128)

#### Streptoprocne zonaris

(Shaw, 1796)

305B00EF-6B47-58A1-8CB3-816EC78BBBD7

##### Distribution

Alvarado, Armero-Guayabal, Ambalema, Anzoátegui, Casabianca, Chaparral, Cajamarca, Carmen de Apicala, Coello, Cunday, Dolores, Espinal, Herveo, Icononzo, Ibagué, Falan, Fresno, Guamo, Honda, Lerida, Libano, Mariquita, Melgar, Murillo, Natagaima, Piedras, Planadas, Prado, Rio Blanco, Roncesvalles, San Antonio, Santa Isabel, San Luis, Venadillo, Villahermosa, Villarica

##### Notes

(v; co SIB 126381, CZUT OR1183; ph ML98402401) WD

#### Chaetura cinereiventris

Sclater, 1862

DEA04A70-9BE2-5D98-8232-1018C5754280

##### Distribution

Armero-Guayabal, Cajamarca, Casabianca, Coello, Falan, Fresno, Ibagué, Libano, Melgar, Murillo, Santa Isabel, San Luis, Villarrica

##### Notes

(v)

#### Chaetura spinicaudus

(Temminck, 1839)

24C598AA-56F1-5663-8B1E-A2550C18FFDC

##### Distribution

Ibagué, Falan, Fresno, San Antonio

##### Notes

(v)

#### Chaetura pelagica

(Linnaeus, 1758)

D8EE2D56-D6AD-5499-BFDE-46C4F2BABAD6

##### Distribution

Armero - Guayabal, Cajamarca, Ibagué, Lerida, Libano, Valle de San Juan

##### Notes

(v; ph ML 503924801)

#### Chaetura brachyura

(Jardine, 1846)

BC4E3785-BFB5-5397-916C-62F5C37B4DB0

##### Distribution

Armero-Guayabal, Falan, Fresno, Honda, Ibagué, Lerida, Libano, Mariquita, Planadas, San Luis, Villarrica

##### Notes

(v; ph ML115462101)

#### Aeronautes montivagus

(d'Orbigny & Lafresnaye, 1837)

8C1BDFF3-9F02-58C9-A16D-F14BDF7D2314

##### Distribution

Chaparral, Falan, Ibagué, Libano, Mariquita, Planadas, Roncesvalles, San Antonio

##### Notes

(v; ph ML43402911)

#### Panyptila cayennensis

(Gmelin, 1789)

6673599C-7CC7-5895-9FE1-6D2A35CA1396

##### Distribution

Armero - Guayabal, Cajamarca, Fresno, Ibagué, Libano

##### Notes

(v)

#### Florisuga mellivora

(Linnaeus, 1758)

1D355CC6-81C7-5152-BB76-5657EC6B3A70

##### Distribution

Armero - Guayabal, Alvarado,Carmen de Apicala, Cajamarca, Casabianca, Chaparral, Coyaima, Cunday, Falan Fresno, Honda, Icononzo, Ibagué, Fresno, Libano, Lerida, Mariquita, Melgar, Planadas, Prado, Saldaña, San Antonio, San Luis, Valle de San Juan, Villarrica

##### Notes

(v; co MLS 249, CZUTOR 096; ph ML123215381)

#### Eutoxeres aquila

(Bourcier, 1847)

661046DE-739D-527C-8811-1D445E8C0B84

##### Distribution

Anzoátegui, Cajamarca, Ibagué, Falan, Fresno, Icononzo, Libano, Planadas, Roncesvalles, San Antonio, Santa Isabel, Villarrica

##### Notes

(v; ca; co CZUTOR 0047; ph ML57129811)

#### Glaucis hirsutus

(Gmelin, 1788)

9A4FAD9D-D858-5FCA-B420-8F579CF93789

##### Distribution

Armero-Guayabal, Coyaima, Cunday, Falan, Fresno, Guamo, Honda, Ibagué, Icononzo, Lerida, Libano, Mariquita, Melgar, Piedras, Planadas, Roncesvalles, Rovira, Saldaña, San Antonio, Villarrica

##### Notes

(v; co; CZUTOR 0277; ph ML123219681)

#### Threnetes ruckeri

(Bourcier, 1847)

3B72D460-693D-554E-A2FB-68334718D755

##### Distribution

Falan, Fresno, Honda, Libano, Mariquita

##### Notes

(ph ML2600159). This hummingbird was captured by F. Espinosa in the vereda Coralito (Líbano) on 12 March 2016 (record from eBird) and was observed on one occasion near the Morales River in Falan in March 2017.

#### Phaethornis striigularis

Gould, 1854

A3AB0D1A-285C-50D5-8BDD-7D307FE64315

##### Distribution

Armero - Guayabal, Casabianca, Ibagué, Falan, Fresno, Libano, Honda, San Luis, Villarrica

##### Notes

(v; co MLS 1651; ph ML26340841)

#### Phaethornis anthophilus

(Bourcier, 1843)

97270101-7F4B-5069-94C6-706C9ECADA33

##### Distribution

Alpujarra, Alvarado, Armero - Guayabal, Casabianca, Chaparral, Carmen de Apicala, Coello, Coyaima, Cunday, Dolores, Espinal, Falan, Guamo, Honda, Ibagué, Icononzo, Lerida, Libano, Mariquita, Melgar, Piedras, Planadas, Prado, Purificación, Rio Blanco, Rovira, Saladaña, San Antonio, San Luis, Venadillo

##### Notes

(v; ca; co MLS 1634, CZUTOR 74; ph ML70659211)

#### Phaethornis guy

(Lesson, 1833)

C45EB5C0-3D64-555A-BB7E-FEA521F11023

##### Distribution

Armero - Guayabal, Anzoategui, Casabianca, Chaparral, Cunday, Heerveo, Honda, Ibagué, Icononzo, Falan, Mariquita, Melgar, Natagaima, Palocabildo, Planadas, Prado, Purificación, Rio Blanco, Rovira, San Antonio, Santa Isabel, San Luis, Venadillo, Villahermosa, Villarrica

##### Notes

(v; ca; co MLS 32327, CZUTOR 23; ph ML99294501)

#### Phaethornis syrmatophorus

Gould, 1851

7047C3DF-DF7C-50D7-B817-85ADD17BF07D

##### Distribution

Anzoátegui, Casabianca, Ibagué, Libano, Planadas, San Antonio, San Luis, Villarrica

##### Notes

(v; ca; co CZUTOR 532; ph ML22279521)

#### Phaethornis longirostris

(Delattre, 1843)

504DB6A8-C2EB-515A-BD57-A3E8CC43288E

##### Distribution

Armero - Guayabal, Falan, Fresno, Lerida, Libano, Mariquita

##### Notes

(v)

#### Doryfera ludoviciae

(Bourcier & Mulsant, 1847)

50B4DC23-201E-5B9A-98C5-CEE542523DD2

##### Distribution

Cajamarca, Casabianca, Chaparral, Cunday, Falan, Herveo, Ibagué, Libano, Murillo, Roncesvalles, Villahermosa, Villarrica

##### Notes

(v; co CZUTOR 0283; ph)

#### Schistes albogularis

Gould, 1851

C2D306A4-CA75-58A6-9619-C324F2B4C8F5

##### Distribution

Anzoátegui, Cajamarca, Casabianca, Falan, Libano, Herveo, Ibagué, Planadas, San Antonio, Villahermosa

##### Notes

(v; ca; co CZUTOR 0786; ph ML 376971311, ML 337952211)

#### Colibri delphinae

(Lesson, 1839)

D59B6FB6-E476-52DA-AEA9-4622D1952249

##### Distribution

Falan, Ibagué, Icononzo, San Antonio, Villarrica

##### Notes

(v; au-XC424028; ca; co MHN-ICN 32372; ph)

#### Colibri cyanotus

(Bourcier, 1843)

BBFD3A3D-B1D4-5B7F-B229-41248EFA2773

##### Distribution

Anzoátegui, Falan, Cajamarca, Ibagué, Libano, San Antonio, Santa Isabel

##### Notes

(v; au; ca; co CZUTOR 0633; ph ML123222211)

#### Colibri coruscans

(Gould, 1846)

E78AB98F-BAB5-5B28-B68A-5BB069BB76DF

##### Distribution

Anzoátegui, Casabianca, Cajamarca, Falan, Fresno, Herveo, Ibagué, Icononzo, Murillo,

##### Notes

(v; au XC336002 ca; co CZUTOR 020; ph ML58016731)

#### Heliothryx barroti

(Bourcier, 1843)

C859E7CD-5AA2-56E2-9E34-E73921CBDB9D

##### Distribution

Falan, Honda

##### Notes

(v) Registered by W. Figueroa. Some records with photo at the border with Caldas (ML 198143691).

#### Chrysolampis mosquitus

(Linnaeus, 1758)

C6EECA43-7ED3-55E8-9858-286CA2809195

##### Distribution

Espinal, Ibagué, Natagaima, Mariquita, Melgar, Prado, San Luis

##### Notes

(v; ca; co MLS 7896, CZUTOR 0325; ph)

#### Anthracothorax nigricollis

(Vieillot, 1817)

8E39C845-E842-5B51-A522-777F2A2978A2

##### Distribution

Armero-Guayabal, Carmen de Apicala, Dolores, Espinal, Falan, Gualanday, Guamo, Honda, Ibagué, Melgar, Murillo, Prado, San Luis, Villarrica

##### Notes

(v; ca; co MLS 174, CZUTOR 0781; ph ML123222801)

#### Heliangelus exortis

(Fraser, 1840)

BD82F5EA-4461-5447-A795-9311B2CBA704

##### Distribution

Anzoátegui, Cajamarca, Falan, Fresno, Ibagué, Murillo, Planadas, Santa Isabel

##### Notes

(v; ca; co CZUTOR 1272; ph ML99048711)

#### Discosura conversii

(Bourcier & Mulsan, 1846)

2BCAEAAE-1956-5873-B966-67017F70BF54

##### Distribution

Falan

##### Notes

(v; ph ML99183441). New record - this is a rare hummingbird in the Department. Individuals have been observed since June 2006 in the path Piedecuesta (Municipality Falan) in the canopy, usually solitary and rarely in small groups of three individuals (Fig. 9b).

#### Lophornis delattrei

(Lesson, 1839)

327A6AAD-70FD-5908-A59C-C01A8926F093

##### Distribution

Honda, Ibagué, Icononzo, Prado

##### Notes

(v; co MLS 1969, ph ML197470921)

#### Lophornis stictolophus

Salvin & Elliot, 1873

7D7BEC0B-1CE7-5113-A5DF-E0647C787FF5

##### Distribution

Icononzo, Prado

##### Notes

(v, co)

#### Adelomyia melanogenys

(Fraser, 1840)

A9A84FC1-D32A-50E9-80E4-4650401DCF92

##### Distribution

Anzoátegui, Casabianca, Cajamarca, Ibagué, Icononzo, Libano, Planadas, Santa Isabel, San Antonio, Villarrica

##### Notes

(v; ca; co CZUTOR 1422; ph ML120532001)

#### Aglaiocercus kingii

(Lesson, 1832)

A3896841-B87A-51C2-9AF7-C99C31389684

##### Distribution

Cajamarca, Falan, Herveo, Ibagué, Libano, Murillo, Planadas, San Antonio, Villarricallahermosa, Villarrica

##### Notes

(v; co MLS 245, CZUT OR0981; ph ML115213621)

#### Opisthoprora euryptera

(Loddiges, 1832)

DE9FA33E-88B7-5EDB-BE49-7D282B25C812

##### Distribution

Cajamarca, Ibagué

##### Notes

(ca; co SIB 6540)

#### Lesbia nuna

(Lesson, 1832)

5B787FE5-272C-58EB-B8F6-332380F2CAF4

##### Distribution

Ibagué, Murillo, Planadas

##### Notes

(v; co MHN-ICN 26170; ph ML115213241)

#### Ramphomicron microrhynchum

(Boissonneau, 1840)

57DEEFE4-D77E-59E4-94F6-0ED0678BBB15

##### Distribution

Anzoátegui, Cajamarca, Ibagué, Murillo, Santa Isabel

##### Notes

(v; co SIB 126768, CZUTOR 745; ph)

#### Oxypogon stubelii

Meyer, 1884

77951766-BBD7-5A0B-A445-DD82BF1DC89E

##### Distribution

Ibagué, Murillo, Santa Isabel

##### Notes

(v; co CZUTOR 0670, ph ML118814161) CE

#### Chalcostigma herrani

(De lattre & Bourcieri, 1846)

526F4864-E979-55CD-A4F2-004BB08EC25F

##### Distribution

Cajamarca, Ibagué, Murillo, Roncesvalles

##### Notes

(v; co CZUT OR1274; ph ML123901711)

#### Metallura tyrianthina

(Loddiges, 1832)

06F24EF1-BA62-5267-8814-D4CD18A54AC8

##### Distribution

Anzoátegui, Cajamarca, Herveo, Ibagué, Murillo, Prado, Santa Isabel, Villarrica

##### Notes

(v; ca; co ISB 166025, CZUTOR 101; ph ML123902171)

#### Metallura williami

(De Lattre & Bourcieri, 1846)

F8AED4F5-495D-55CB-83B3-8C3CEA01175A

##### Distribution

Cajamarca, Ibagué, Murillo, Planadas, Prado, Roncesvalles, Santa Isabel, Villarrica

##### Notes

(v; ca; co CM 70714, CZUTOR 238; ph ML32218831)

#### Haplophaedia aureliae

(Bourcier & Mulsant, 1846)

E8E486FC-F70A-5353-9FF9-B5AA3D9FA51D

##### Distribution

Falan, Ibagué, Planadas, San Antonio, Santa Isabel

##### Notes

(v; ca; co CZUTOR 0365)

#### Eriocnemis vestita

(Lesson, 1839)

31AF6E28-D4E2-5DE0-A4F8-DC4CF738AF7D

##### Distribution

Cajamarca, Murillo, Planadas

##### Notes

(v; co MHN-ICN 26157) A reg. H. Arias.

#### Eriocnemis derbyi

(De lattre & Bourcier, 1846)

1E00334A-18F9-5000-9EBF-6575A0A72B07

##### Distribution

Anzoátegui, Cajamarca, Herveo, Ibagué, Murillo, Roncesvalles, Santa Isabel

##### Notes

(v; co; CZUTOR 1242; ph ML22278111) CE NT

#### Eriocnemis mosquera

(De lattre & Bourcier, 1846)

32F1ED43-09A7-5120-BDE0-FCE85460E22B

##### Distribution

Anzoátegui, Cajamarca, Herveo, Ibagué, Murillo, Planadas, Roncesvalles, Santa Isabel

##### Notes

(v; ca; co SIB 126815, CZUTOR 141; ph ML68138951) CE

#### Eriocnemis aline

(Bourcier, 1843)

558647B9-7B58-562F-A841-682BD8433BAD

##### Distribution

Planadas, (Nacimiento río Saldaña)

##### Notes

(co MLS 2324)

#### Aglaeactis cupripennis

(Bourcier, 1843)

6E0BD681-DD99-5511-A641-0EBBC0FA52BC

##### Distribution

Cajamarca, Ibagué, Herveo, Murillo, Santa Isabel

##### Notes

(v; ph ML58243691)

#### Coeligena coeligena

(Lesson, 1833)

968D47AF-227F-51D9-8594-13F7F9886508

##### Distribution

Anzoátegui, Casabianca, Cajamarca, Herveo, Ibagué, Libano, Murillo, Planadas, Santa Isabel, Villarrica

##### Notes

(v; ca; co CZUTOR 0218; ph ML108866751)

#### Coeligena torquata

(Boissonneau, 1840)

5BFCF9E4-7301-52FC-BE6D-42C70B9D697A

##### Distribution

Anzoátegui, Cajamarca, Fresno, Ibagué, Murillo, Planadas, Santa Isabel, Villarrica

##### Notes

(v; ca; co MHN-ICN 26871, CZUTOR 124; ph ML23431181)

#### Coeligena lutetiae

(De lattre & Bourcier, 1846)

47016F9C-2B50-5F46-AD9D-2C62DDB5F3F1

##### Distribution

Anzoátegui, Cajamarca, Roncesvallesa, Ibagué, Herveo, Murillo, Roncesvalles

##### Notes

(v; ca; co CZUTOR 0366; ph ML105812131)

#### Coeligena helianthea

(Lesson, 1839)

3582B40A-57C9-5679-B1D8-2ACA3155D7DD

##### Distribution

Icononzo

##### Notes

(co CUMV 5009)

#### Lafresnaya lafresnayi

(Boussonneau, 1840)

7091E231-9458-5918-BE8D-299E21322635

##### Distribution

Anzoátegui, Cajamarca, Falan, Ibagué, Santa Isabel, Murillo

##### Notes

(v; ca; co SIB 126814, CZUTOR 298; ph ML114453591)

#### Ensifera ensifera

(Boissonneau, 1840)

1E923E58-AB9B-581A-B5B3-CABD25E9D3D1

##### Distribution

Anzoátegui, Ibagué, Planadas, Santa Isabel, Roncesvalles

##### Notes

(v; ca; co CZUTOR 0165; ph ML115212971)

#### Pterophanes cyanopterus

(Fraser, 1840)

FEDDDA58-BAA5-5E2B-AC93-00A3320AF0BC

##### Distribution

Cajamarca, Honda, Ibagué, Icononzo, Murillo, Santa Isabel

##### Notes

(v; ca; co SIB -A4738; ph ML32219061)

#### Boissonneaua flavescens

(Loddiges, 1832)

4B48EE5B-5E7B-5847-86CF-9CEB4F819760

##### Distribution

Cajamarca, Ibagué, Libano, San Antonio

##### Notes

(v; ca; co MHN-ICN 26873, CZUTOR 488; ph ML123227351)

#### Ocreatus underwoodii

(Lesson, 1832)

8D944338-D768-55CB-859D-61631CEE7B87

##### Distribution

Anzoátegui, Falan, Fresno, Ibagué, Libano, Planadas, San Antonio, Villahermosa

##### Notes

(v; ca; co CZUTOR 0642; ph ML79620671)

#### Heliodoxa rubinoides

(Bourcier & Mulsant, 1846)

A244CE40-DD7F-534B-A9CA-4D4DE05BAE40

##### Distribution

Cunday, Fresno, Ibagué, Murillo

##### Notes

(v; ca; co CZUTOR 0317; ph ML123229231)

#### Heliodoxa leadbeateri

(Bourcier, 1843)

2E3434DA-6AB6-5F1C-8054-F9E96BC4254A

##### Distribution

Anzoátegui, Chaparral, Falan, Planadas, Rio Blanco

##### Notes

(v; co CZUTOR 1041, 1105)

#### Heliomaster longirostris

(Audebert & Vieillot, 1801)

D6B7DCF4-5811-5A3F-B559-AA6AE7A7EB55

##### Distribution

Chaparral, Cunday, Honda, Ibagué, Icononzo

##### Notes

(v; co MLS 2462, CZUT OR1410; ph ML73918541)

#### Chaetocercus mulsant

(Bourcier, 1843)

2A263789-DD2B-55E9-980B-F297CF57A00B

##### Distribution

Casabianca, Falan, Herveo, Ibagué, Libano, Murillo, San Antonio, Villahermosa

##### Notes

(v; co CZUTOR 0318; ph ML93615631)

#### Chaetocercus heliodor

(Bourcier, 1840)

EEC24601-437E-59F0-B97C-801D7FB19B4E

##### Distribution

Ibagué

##### Notes

(co AMHN 76505; ph ML70658091) Recorded for Ibagué as Acestrura harterti (ornis II P202 1901).

#### Philodice mitchellii

(Bourcier, 1847)

E12F7585-1FCD-50DC-A7D2-2D88AB45D4E7

##### Distribution

Ibagué, Purificación

##### Notes

(v, ph ML123230551) This species was registered specifically in the Municipality of Ibagué in the Combeima River Canyon and north-western hills. Individuals were perched at about 1600 to 1800 m a.s.l. (Fig. 9c).

#### Chlorostilbon melanorhynchus

Gould, 1860

C8203343-57DA-5442-B3E6-943DCA0A0891

##### Distribution

Coyaima, Cunday, Dolores, Honda, Ibagué, Icononzo, Falan, Mariquita, Planadas, Prado, Rio Blanco, San Antonio, San Luis, Venadillo, Villarrica

##### Notes

(v; ca; co CZUTOR 0273; ph ML26002221)

#### Chlorostilbon gibsoni

(Fraser, 1840)

4EC390C2-72BC-5314-B162-9C55901B6383

##### Distribution

Armero-Guayabal, Chaparral, Espinal, Gualanday, Honda, Ibagué, Melgar, Prado, Rio Blanco, San Antonio, San Luis

##### Notes

(v; au XC424725; ca; co MLS 1993, CZUTOR 0179; ph ML102230501) CE

#### Chlorostilbon poortmani

(Bourcier, 1843)

89624FCD-5A3A-5018-8997-5DBA9804F451

##### Distribution

Prado (Río Saldaña)

##### Notes

(co CZUT-OR0383, 0273)

#### Klais guimeti

(Bourcier, 1843)

C48C474E-D315-5757-9A9C-7AD24B01963B

##### Distribution

Honda

##### Notes

(co AMHN 94841)

#### Anthocephala berlepschi

Salvin, 1893

52F8D711-4F8C-5668-9B90-3F7788B55D27

##### Distribution

Falan, Ibagué, Libano, Planadas, Roncesvalles, Villahermosa

##### Notes

(v; ca; co CZUT OR1442; ph ML75640231) E VU El. This endemic species has been recorded at elevations of 1600 to 2500 m a.s.l.. It has been found along forest edges, secondary scrubland and wooded areas bordering ravines in an errant way and is rarely seen perched at low altitudes. It has also been observed in Falan, Villahermosa, Planadas, Líbano, Ibagué and Roncesvalles (Fig. 8f).

#### Campylopterus falcatus

(Swainson, 1821)

D05A68E8-5E87-5464-903A-FC3E7933F94D

##### Distribution

Cunday, Ibagué, Planadas, Rio Blanco

##### Notes

(v; co; AMHN 94823, MHN ICN 26261; ph ML116424351). New record - this species went unnoticed for years in the Department. In recent years, it has been regularly recorded from 1000 to 1600 m a.s.l. near the Combeima River Canyon and Anaime. Previous records of the species date from 2002 in Ibagué and Bocas de Anamichu in Rio Blanco in 2008 (Parra-Hernández et al. 2007, Molina-Martínez et al. 2015).

#### Chalybura buffonii

(Lesson, 1832)

BBF7AB8A-70AA-5DFC-958D-ADB149226A88

##### Distribution

Armero-Guayabal, Cunday, Dolores, Falan, Fresno, Honda, Ibagué, Icononzo, Lerida, Libano, Planadas, Rio Blanco, San Antonio, San Luis, Venadillo, Villarrica

##### Notes

(v; ca; co MLS 8296, CZUTOR 45; ph ML70659201) WD

#### Chalybura urochrysia

(Gould, 1861)

5E62A52C-2601-5452-AE81-0F4C8CAB5B45

##### Distribution

Armero-Guayabal, Ambalema, Honda, Venadillo

##### Notes

(v; co CZUTOR)

#### Thalurania colombica

(Bourcier, 1843)

81C1DDFA-BE70-5B2E-9B63-190B30132D1C

##### Distribution

Armero-Guayabal, Cajamarca, Falan, Herveo, Honda, Ibagué, Icononzo, Lerida, Libano, Mariquita, Melgar, Ortega, Planadas, Prado, San Antonio, Rio Blanco, San Luis, Villarrica

##### Notes

(v; ca; co MLS 219, CZUTOR 310; ph ML104936211) WD

#### Saucerottia saucerottei

(Delattre & Bourcier, 1846)

24C46D24-5895-547E-848C-1BAE1CE543E4

##### Distribution

Espinal, Ibagué, Libano, San Antonio

##### Notes

(co MLS 8967; ph ML102953231)

#### Saucerottia cyanifrons

(Bourcier, 1843)

B0773D3E-235B-53A4-88A6-06AAF99BC11D

##### Distribution

Cunday, Espinal, Falan, Guamo, Gualanday, Ibagué, Icononzo, Libano, Melgar, San Antonio, Villarrica

##### Notes

(v; ca; co MLS 2128, CZUTOR 0024; ph ML70658991) CE. This hummingbird is regularly observed at elevations of 1200 to 2100 m a.s.l. (Fig. 9a). Fig. 9a

#### Amazilia tzacatl

(de la Llave, 1833)

FF77A8C8-E08D-53E4-B9F2-5955703B454C

##### Distribution

Armero-Guayabal, Alvarado,Coyaima, Cunday, Dolores, Falan, Fresno, Honda, Ibagué, Icononzo, Lerida, Libano, Mariquita, Melgar, Prado, Purificación, Rio Blanco, San Antonio, San Luis, Venadillo, Villarrica

##### Notes

(v; au XC424725; co MLS 7897; CZUTOR 0076; ph ML72670111) WD

#### Uranomitra franciae

(Bourcier & Mulsant, 1846)

EA9C54F7-2D13-5F7C-8C4A-60CB041FB850

##### Distribution

Cajamarca, Casabianca, Chaparral, Cunday, Falan, Fresno, Herveo, Honda, Ibagué, Libano, Murillo Planadas, San Antonio, Rio Blanco, Roncesvalles, Villarrica

##### Notes

(v; ca; co CZUTOR 0019; ph ML123232881, ML 533974481)

#### Chrysuronia goudoti

(Bourcier, 1843)

00189CA4-3E6C-5644-9384-3BFD8FCB5A13

##### Distribution

Armero-Guayabal, Carmen de Apicala, Chaparral, Espinal, Guamo, Gualanday, Honda, Ibagué, Melgar, Natagaima, Mariquita, Melgar, Prado, Saldaña, San Antonio, San Luis, Venadillo

##### Notes

(v; ca; co MLS 2495, CZUTOR 328; ph ML123886341)

#### Polyerata amabilis

(Gould, 1853)

02B80778-D943-5631-9BA2-CF5A71692FA4

##### Distribution

Armero-Guayabal, Honda, Ibagué, Mariquita, Melgar, Prado, San Luis, Venadillo

##### Notes

(v; ca; co CZUT 0431; ph)

#### Chlorestes julie

(Bourcier, 1842)

6E748946-786D-5678-9376-DEA578C45738

##### Distribution

Armero-Guayabal, Carmen de Apicala, Espinal, Falan, Honda, Ibagué, Libano, Mariquita, Melgar, Venadillo

##### Notes

(v; ca; co MLS 237)

#### Aramus guarauna

(Linnaeus, 1766)

6D1C79D9-8CF1-588A-82D1-E6C702A27366

##### Distribution

Alvarado, Ambalema, Doima, Ibagué, Espinal, Lerida

##### Notes

(v; au; co MLS 3351; ph ML108051151). New record - the first record of this species was made on 27 December 2011 when an individual was observed at the El Carmen Fish Farm (Ibagué) consuming snails and oysters (Fig. 6d). Nick Bayly (SELVA) and W. Figueroa registered this species in the Prado Reservoir on 13 January 2013 and on 23 March 2016, respectively. Subsequently, we registered it in Espinal, Venadillo, Lérida, Ambalema and Piedras alone or in groups of no more than six individuals.

#### Porphyrio martinica

(Linnaeus, 1766)

1A9277BE-6BC1-53D6-A5D5-D919B7E69CBB

##### Distribution

Armero-Guayabal, Carmen de Apicala, Coyaima, Falan, Ibagué, Mariquita, Prado, Venadillo

##### Notes

(v; co MHN-ICN 4386, CZUTOR 430; ph ML118594861)

#### Anurolimnas viridis

(Statius Muller, 1776)

6EBCF42D-4676-52BB-909B-4EA19C9A68E1

##### Distribution

Alvarado, Ibagué, Falan, Fresno, Libano

##### Notes

(v; au ML82120; ca; co CZUTOR 0198; ph)

#### Laterallus albigularis

(Lawrence, 1861)

83B049DF-B718-5244-8244-BF4241B9C4F3

##### Distribution

Guamo, Ibagué, Piedras, San Luis

##### Notes

(v; co CZUTOR 0831; ph ML23232771)

#### Coturnicops notatus

(Gould,1841)

E6472D81-D921-5A00-A74A-916B9C85299A

##### Distribution

Espinal

##### Notes

(co MLS 8819) An individual was deposited in the collection La Salle, which was found in the Espinal.

#### Mustelirallus erythrops

(Sclater, 1867)

C9A4A871-3FC8-5240-86E0-5D2E2BA73913

##### Distribution

Mariquita

##### Notes

(ca, ph) This unusual species in the Department was observed for the first time on 24 October 2019 near the Guali River (Parra-Hernández et al. 2019).

#### Pardirallus maculatus

(Boddaert, 1783)

65622525-C0C1-53F7-BEDA-9C01C784605E

##### Distribution

Ibagué, Espinal, Guamo, Purificación

##### Notes

(v; au; co MLS 4756; ph ML123235741) ICN. This is an uncommon species of the rice fields of Tolima. An individual was observed on 26 October 2010 by D. Carantón in the Lagoon La Esmeralda (Ibagué). We registered this species in 2017. A tired individual was observed in the courtyard of a house where there had previously been an urban wetland in the City of Ibagué (Molina-Martínez 2014). Some individuals were observed near irrigation channels in Espinal in 2013 and two individuals were observed on 28 September 2015 in the Totumo (Fig. 6f).

#### Aramides cajaneus

(Statius Muller, 1776)

CA852C98-FF36-5D39-A2E6-A2AB5230FD00

##### Distribution

Armero-Guayabal, Alvarado,Coyaima, Cunday, Falan, Guamo, Honda, Ibagué, Mariquita, Prado, Purificación, Rio Blanco, San Antonio, San Luis, Venadillo, Villarrica

##### Notes

(v; au; co MHN-ICN 21836; ph ML118594601)

#### Porzana carolina

(Linnaeus, 1758)

8E9C0155-C8D3-53B9-9ABA-DB97074E4238

##### Distribution

Ibagué, Espinal, Piedras

##### Notes

(co MLS 8509; ph) There were a few records of this species in Tolima on 13 March 2015 on the route to Piedras. P.G. Nell recorded two individuals in April 2018 (Fig. 6e).

#### Gallinula galeata

(Lichtenstein, 1818)

2C62B74F-B13C-5593-A9CE-B7A45FB85603

##### Distribution

Armero-Guayabal, Alvarado, Espinal, Ibagué, Mariquita, Honda, Piedras, Prado

##### Notes

(v; ph ML123236601)

#### Fulica americana

Gmelin, 1789

C282F37A-589D-5166-BFED-208FA3DE340B

##### Distribution

Anzoátegui, Honda

##### Notes

(v)

#### Pluvialis dominica

(Statius Muller, 1776)

390BB293-9964-564F-92D7-3BD9067B4D6B

##### Distribution

Ibagué

##### Notes

(v)

#### Vanellus chilensis

(Molina, 1782)

04079A10-EEA4-5F8A-BF63-AF4E8DAEAC56

##### Distribution

Armero-Guayabal, Alvarado,Anzoátegui, Ambalema, Chaparral, Coyaima, Guamo, Espinal, Honda, Ibagué, Falan, Libano, Mariquita, Murillo, Prado, Purificación, Rio Blanco, San Antonio, San Luis, Venadillo, Villarrica

##### Notes

(v, au XC384415, ML97062611; co AMHN 35233; ph ML123242991) WD

#### Vanellus resplendens

(Tschudi, 1843)

75F5D9F8-460F-517A-9E88-3599F21DEB57

##### Distribution

Anzoátegui, Cajamarca, Herveo, Ibagué

##### Notes

(v; ph ML122237191) Alcaraván registered this species in the páramo zone in specific areas near the Tolima and Ruiz peaks. A photographic record was made by C. Guevara in 2018 (Fig. 7c).

#### Anarhynchus collaris

Vieillot, 1818

E54681B8-89E0-5907-AD9E-6C2C7EBEAED2

##### Distribution

Ibagué, Ortega, Piedras, Venadillo

##### Notes

(V; au ML111647501; ca; co MLS 8322; ph ML111647251)

#### Himantopus mexicanus

(Statius Muller, 1776)

1CE1C288-53CE-54F3-BD0B-1FBD2A4E2EEB

##### Distribution

Ibagué, Purificación

##### Notes

(v; ph ML24395601). New record - an uncommon species in the Department, it has only been registered in the lagoon complex El Escobal. The first record of this species was made on 2 March 2008 by the Tolima Bird Observer Group (GOAT). It has been subsequently observed in the same place on a regular basis alone or in groups of eight individuals (October 2018) (Fig. 7a).

#### Hesperoburhinus bistriatus

(Wagler, 1829)

3FDBB6B8-F4E8-5259-BEE0-CB27B22959F9

##### Distribution

Alvarado, Doima, Ibagué, Natagaima, San Luis

##### Notes

(v; ph ML123242151). This species is difficult to see and has always been observed in a solitary way. For the first time, it was observed in the desert of Natagaima bordering Aipe (Huila) on 20 March 2004. Later, on 22 June 2006, it was observed in Piedras in an arid area adjacent to the rice fields. Between 2017 and 2018, we regularly registered several individuals in Ventaquemada (Alvarado).

#### Bartramia longicauda

(Bechstein, 1812)

2FB90D6B-4C15-5D93-B32D-8937536599DC

##### Distribution

Espinal

##### Notes

(co AMHN 111327)

#### Calidris bairdii

(Coues, 1861)

4A3A29BA-7392-5490-92D8-EA00928A9126

##### Distribution

Ibagué, Roncesvalles

##### Notes

(v; ph https://www.flickr.com/photos/proaves/3577733780)

#### Calidris minutilla

(Vieillot, 1819)

D7E9F365-8F36-5F16-AC30-303E51B32268

##### Distribution

Alvarado, Doima, Ibagué, Piedras

##### Notes

(v; ph ML124781241) M

#### Calidris melanotos

(Vieillot, 1819)

0C6C8577-4DFD-5414-B26F-1CCEB2BB2311

##### Distribution

Ibagué

##### Notes

Migrant species with sporadic records in the Department of Tolima.

#### Calidris mauri

(Cabanis, 1857)

532D846A-121B-53AB-82DC-7CA588ADBA65

##### Distribution

Ibagué

##### Notes

(v; ca; ph) M. This sporadic species was registered on one occasion at El Toro Lagoon Complex and photographed on 5 January 2005 (Parra-Hernández et al. 2007).

#### Gallinago jamesoni

(Jardine & Bonaparte, 1855)

3CFC0055-413E-5F72-9589-3B31831E225D

##### Distribution

Ibagué, Santa Isabel

##### Notes

(co ANSP 154802)

#### Gallinago nobilis

Sclater, 1856

7BD7FF2D-E5A1-5608-B1C2-EEBA2AE9E9FE

##### Distribution

Cajamarca, Ibagué, Planadas, Santa Isabel

##### Notes

(V; co ANSP 154803; ph ML62591231)

#### Gallinago delicata

(Ord, 1825)

8573A605-D611-524E-B209-58E595751464

##### Distribution

Ibagué, Murillo

##### Notes

(v Three collection records at the border of the Municipality of Natagaima with the Department of Huila; co MVZ 93749).

#### Actitis macularius

(Linnaeus, 1766)

5ED1A352-CB31-5540-8047-651D60723C5C

##### Distribution

Alvarado, Cajamarca, Chaparral, Carmen de Apicala, Doima, Falan, Honda, Ibagué, Melgar, Piedras, Prado, Purificación

##### Notes

(v; ph ML111644551; ca; co CZUTOR 0201) M

#### Tringa solitaria

Wilson, 1813

97E37D33-4220-53FB-96EE-52659FE1FC2B

##### Distribution

Armero-Guayabal, Alvarado,Cajamarca, Guamo, Falan, Ibagué, Honda, Purificación

##### Notes

(v; co MHN, ICN 1187; ph ML123934521) M

#### Tringa melanoleuca

(Gmelin, 1789)

02ECB043-96B3-5C08-A80F-37E071CD4D64

##### Distribution

Alvarado, Doima, Ibagué, San Luis

##### Notes

(v; au ML 222194951; ph ML 183616821) M. This is a migratory species that is sporadically observed in some areas of the Department. We found a record of an individual on 13 October 2004 in Villacafe sector (Ibagué), the rice fields of Saldaña, Piedras and Roncesvalles. It was recently (October 2018) observed by J. Poveda in Murillo (Fig. 7b).

#### Tringa semipalmatus

(Gmelin, 1789)

AB136F12-6E6D-5C7D-8568-F2B33DEE8651

##### Distribution

Melgar

##### Notes

(co MCZ 347551)

#### Tringa flavipes

(Gmelin, 1789)

1BF0E906-A116-5CB6-B775-E810115428CC

##### Distribution

Falan, Ibagué, Piedras

##### Notes

(v; ph ML 182836571) M

#### Jacana jacana

(Linnaeus, 1766)

7B11A094-E6DD-5CE5-BEF3-8D4F041725CB

##### Distribution

Armero-Guayabal, Ambalema, Carmen de Apicala, Coyaima, Espinal, Falan, Honda, Guamo, Ibagué, Libano, Mariquita, Prado, San Luis, Venadillo

##### Notes

(v; au; ca; co MLS 2918, CZUTOR 440; ph ML123244611) WD

#### Rynchops niger

Linnaeus, 1758

2BA99EF3-C3EE-577D-A1C3-1558CD319812

##### Distribution

Ambalema, Ibagué, Honda

##### Notes

(v; ca; co; ph ML123250951). This species has been observed in the Magdalena River Basins (Honda, Ambalema and Girardot) and large lagoons in the Department. It usually forms groups of up to 12 individuals. At Laguna El Escobal (Picaleña, Ibagué), a small group was initially seen in 2008, which spread to other municipal water bodies and Piedras (Fig. 7e).

#### Leucophaeus atricilla

(Linnaeus, 1758)

3629203A-C638-5BEC-ACDC-5A40A0B1EDC3

##### Distribution

Ibagué, Libano, Alvarado

##### Notes

(v; ph) M. Photographic record in the black lagoon (Caldas), less than 200 m from the limits with the Tolima (ML 206785451).

#### Sternula superciliaris

(Vieillot, 1819)

5B660B9E-281F-5F79-A324-146A00E8C414

##### Distribution

Venadillo

##### Notes

(v) Several records near Honda

#### Phaetusa simplex

(Gmelin, 1789)

414ED18D-216E-51E0-B021-E1EFCBD10B28

##### Distribution

Ambalema, Armero-Guayabal, Honda, Suarez

##### Notes

(v; ph ML123250851). This species has been observed in the Magdalena River Basins (Honda, Ambalema and Girardot) and large lagoons in the Department. It usually forms groups of up to 12 individuals. At Laguna El Escobal (Picaleña, Ibagué), a small group was initiallyseen in 2008, which spread to other municipal water bodies and Piedras (Fig. 7e).

#### Eurypyga helias

(Pallas, 1781)

62828CB3-DB9C-58F8-8754-848777B04601

##### Distribution

Chaparral

##### Notes

(ph ML99634321) An individual photographed by D. Ceballos

and an individual was photographed.

#### Mycteria americana

Linnaeus, 1758

F287D612-932B-557F-8788-A0F9AE0AB218

##### Distribution

Armero-Guayabal, Ibagué, Lerida

##### Notes

(v; ph ML118594791). New record - this species is occasionally observed in Tolima. This species has been observed below 1,000 m a.s.l. in a casual way since 2005 (Fig. 5f) in the Municipalities of Piedras, Ibagué, Alvarado and Venadillo.

#### Anhinga anhinga

(Linnaeus, 1766)

CEF3B892-4386-5B23-BBBD-976CA6973E1F

##### Distribution

Armero-Guayabal, Ibagué

##### Notes

(v; ph ML68428501)

#### Phalacrocorax brasilianus

(Gmelin, 1789)

02FCD71A-284C-5FEC-A6D5-F5BE86854EF9

##### Distribution

Armero-Guayabal, Ambalema, Chaparral, Ibagué, Honda, Libano, Mariquita, Roncesvalles, Venadillo

##### Notes

(v; ph ML123251401)

#### Pelecanus occidentalis

(Linnaeus, 1766)

414CC410-022B-5B5E-AFF2-2D6D9787A063

##### Distribution

Ibagué, Prado

##### Notes

(v; ph **ML350019381**)

#### Tigrisoma lineatum

(Boddaert, 1783)

57C6BEA8-E0D5-5E79-B150-FD45909F0902

##### Distribution

Ibagué, Falan

##### Notes

(v; ph). New record - an individual was observed on 5 January 2005 at the edge of the Lagoon El Toro. On 21 February 2015, D. Quintero registered an individual in the Sports Park. On 7 November 2010, a record was made by T. Rasmussen in Laguna del Hato. An observation was made in Vereda Chenche Aguafría on 7 December 2015 by C. Martínez. In 2015, an individual was photographed at the Prado Reservoir by W. Figueroa (Fig. 5a).

#### Cochlearius cochlearius

(Linnaeus, 1766)

693BA07D-0FB9-506D-B8F0-163A1529FA7B

##### Distribution

Armero-Guayabal, Ibagué, Prado

##### Notes

(v; ph ML123261191) EL. New record - an individual was photographed in Laguna El Toro (Municipality of Ibagué) on 5 December 2012 (Fig. 5b). On 14 May 2016, two individuals were spotted in Llanitos of Combeima by A. Buitrago (annotated record in Ebird).

#### Agamia agami

(Gmelin, 1789)

31578FDC-0E4B-5D1E-AAFF-9C32EF00C3B9

##### Distribution

Ambalema, Piedras, Venadillo

##### Notes

(ph ML55849831). On 9 June 2014, an individual was photographed by J. Drucker in the Jabirú Reserve (Guayabal). On 2 September 2012, O. Marín registered an individual in the Village of Tavera (Venadillo Municipality). On 23 November 2015, K. Certuche observed an individual in Venadillo and F. Espinosa photographed an individual in the same locality (pers. commun., F. Espinosa, Fig. 5d).

#### Ixobrychus exilis

(Gmelin, 1789)

21CE5926-FC1A-5B5E-9F95-DFE356EF4C12

##### Distribution

Piedras

##### Notes

(ph ML118752031). New record - an individual was photographed at Finca Gascoña (Piedras) on the high pastures bordering a lagoon in the morning hours on 13 October 2018 (Fig. 5c). This record is the first of this species in the Upper Magdalena Valley.

#### Pilherodius pileatus

(Boddaert, 1783)

3FD967F2-8C7D-5B9E-9E0B-F46983C7D28E

##### Distribution

Armero-Guayabal, Ambalema, Ataco, Chaparral, Falan, Honda, Ibagué, Mariquita, Pi Planadas, Prado, Purificación, Venadillo

##### Notes

(v; ph ML63999381)

#### Syrigma sibilatrix

(Temminck, 1824)

DEC7339D-C579-51F7-9B4E-8BA3EB4576F4

##### Distribution

Ambalema, Armero-Guayabal, Ibagué, Venadillo, Honda

##### Notes

(v; ph ML27837881). The first record of this species was made on 28 February 2005 at the lagoon complex of Salado (Ibagué) (Parra-Hernández et al. 2007). Later, different records were made towards the north of Tolima. In Pajonales (Ambalema), an individual was registered on 12 May 2016 by Y. Tolosa. Another record was made in Venadillo on 18 January 2015 (pers. commun., D. Garzón).

#### Egretta caerulea

(Linnaeus, 1758)

2FDB7972-329A-5A9B-8ABD-CF4A2469B584

##### Distribution

Doima, Ibagué, Piedras

##### Notes

(v; ph ML123255131) Mb

#### Egretta tricolor

(Statius Muller, 1776)

6240CE11-89B0-524A-A688-92260AAF99DF

##### Distribution

Armero-Guayabal

##### Notes

(v)

#### Egretta thula

(Molina, 1782)

27FB1197-8B16-5465-8F02-CCC68A929E48

##### Distribution

Armero-Guayabal, Ambalema, Alvarado, Coyaima, Espinal, Falan, Ibagué, Piedras, Prado, Purificación, San Luis

##### Notes

(v; ph ML118593561)

#### Nyctanassa violacea

(Linnaeus, 1758)

3359A322-C856-5EAD-805A-DB9F48872E87

##### Distribution

Ambalema, Honda, Ibagué, Natagaima

##### Notes

(ph ML124779931)

#### Nycticorax nycticorax

(Linnaeus, 1758)

C5627A3F-DA40-5BAF-AEB3-4FC59D731439

##### Distribution

Piedras, Purificación, Ibagué, San Luis, Saldaña, Espinal, Flandes, Carmen de Apicala, Ambalema, Armero-Guayabal, Honda

##### Notes

(co USNM 444273; ph ML123259071)

#### Butorides striata

(Linnaeus, 1758)

11BA500F-831C-5AA1-B397-DBE50B88B160

##### Distribution

Armero-Guayabal, Carmen de Apicala, Chaparral, Cajamarca, Coyaima, Cunday, Coyaima, Espinal, Guamo, Honda, Ibagué, Falan, Piedras, Prado, Purificación, San Luis, Venadillo

##### Notes

(v; au; co MLS 2947, MHN ICN 4372; ph ML123241141) WD

#### Butorides virescens

(Linnaeus, 1758)

6F13E43D-04DF-59F3-A98D-5ECF42505020

##### Distribution

Armero-Guayabal, Ambalema, Ibagué, Honda, San Luis

##### Notes

(v; co AMHN 94799; ph ML123240481). This is a casual species in the Department of Tolima. On 5 May 2017, an individual was observed in the rice fields of Ambalema; another individual was sighted on 10 January 2013 in the lagoon complex of Picaleña (Ibagué). Nick Bayly de Selva recorded an individual on 24 November 2012 in the paddy fields of Prado.

#### Bubulcus ibis

(Linnaeus, 1758)

F1ED18B4-4200-5134-8728-C270559661CD

##### Distribution

Armero-Guayabal, Alvarado,Ambalema, Casabianca, Chaparral, Cajamarca, Coyaima, Cunday, Espinal, Falan, Guamo, Honda, Ibagué, Lerida, Libano, Mariquita, Piedras, Planadas, Purificación, Rio Blanco, San Luis, Venadillo, Villarrica

##### Notes

(v; co MLS 78, CZUT OR1150; ph ML123255851) WD

#### Ardea alba

Linnnaeus, 1758

16450E0A-5D85-570F-B325-F6982E6E95DB

##### Distribution

Armero-Guayabal, Alvarado,Ambalema, Chaparral, Coyaima, Espinal, Ibagué, Falan, Mariquita, Libano, Piedras, Prado, Purificación, San Luis, Venadillo

##### Notes

(v; ph ML63999471)

#### Ardea herodias

Linnaeus, 1758

FEFE9BB9-F997-589B-977C-E8521A893994

##### Distribution

Ibagué, Melgar, Mariquita

##### Notes

(v; ph ML 246484781) Migrant species with sporadic records in the Tolima.

#### Ardea cocoi

Linnaeus, 1766

5A8F1F93-0E02-51E6-8D8D-BED732D320FA

##### Distribution

Armero-Guayabal, Alvarado, Ambalema, Coyaima, Coyaima, Espinal, Ibagué, Piedras, Prado, Purificación

##### Notes

(v; co ICN 163; ph ML118593081)

#### Plegadis falcinellus

(Linnaeus, 1766)

D4E7BE71-7ED4-5CA8-8976-196984E33F14

##### Distribution

Ambalema, Armero-Guayabal, Ibagué

##### Notes

(v; ph ML24371311). New record - an individual was spotted during the morning hours of 13 February 2016 in the sector of Picaleña, Laguna El Escobal, over 900 m a.s.l., foraging alone, but then together with other aquatic bird species. These sightings correspond to occasional sightings of the tropical dry forest (Fig. 5e) and are the first records of this species in the Upper Magdalena Valley.

#### Mesembrinibis cayennensis

(Gmelin, 1789)

7A826706-680A-5B75-AA71-F12E13ED0BCB

##### Distribution

Armero-Guayabal, Libano, Mariquita, Venadillo

##### Notes

(v)

#### Phimosus infuscatus

(Lichtenstein, 1823)

8996F499-F67B-5BF2-BF51-66F2FA87EC2F

##### Distribution

Armero-Guayabal, Ambalema, Casabianca, Coyaima, Coyaima, Ibagué, Falan, Honda, Mariquita, San Luis, Piedras, Prado, Purificación, Venadillo

##### Notes

(v; ca; co ICN 181787; ph ML123263751)

#### Theristicus caudatus

(Boddaert, 1783)

A3CABC54-ABFA-5B49-96EF-296577405A7E

##### Distribution

Ataco, Melgar, Ibagué

##### Notes

(v; ph ML 221533741). Common in beaches of the Magdalena River. New record - on 13 April 2016, an individual was observed and photographed by H. Cruz in the Municipality of Melgar. This person also observed two individuals in Las Villas (Ataco) on 4 December 2017 (pers. commun., H. Cruz). S. García registered an individual in the Rio Viejo Wetland on 18 January 2018.

#### Platalea ajaja

(Linnaeus, 1758)

508D8A47-E7C2-5A4F-B566-40BEA94386D5

##### Distribution

Natagaima, Ambalema, Venadillo .

##### Notes

(v; ph ML645648414)

#### Sarcoramphus papa

(Linnaeus, 1758)

602601B4-AED2-5018-8519-A3404A1653FA

##### Distribution

Armero - Guayabal, Alvarado,Chaparral, Coello, Coyaima, Espinal, Falan, Ibagué, Lerida, Libano, Mariquita, Honda, Natagaima, Piedras, Purificación, San Antonio, San Luis, Venadillo

##### Notes

(v; co MLS 220; ph ML123266381)

#### Vultur gryphus

Linnaeus, 1758

35EAD4AD-35C3-5723-B431-60944658662E

##### Distribution

Anzoátegui, Ibagué, Murillo, Roncesvalles, Villahermosa

##### Notes

(v; ph ML118451711) NT

#### Coragyps atratus

(Bechstein, 1793)

985B7409-50CC-5408-A8F7-7F9E37B3A0AF

##### Distribution

Armero-Guayabal, Alvarado, Ambalema, Anzoátegui, Casabianca, Cajamarca, Chaparral, Coyaima, Falan, Ibagué, Lerida, Libano, Prado, Santa Isabel, Rio Blanco, San Antonio, San Luis, Venadillo

##### Notes

(v; ph ML123267051)

#### Cathartes aura

(Linnaeus, 1758)

7D6316B6-4CA1-5185-BF5A-CBDD43C30F3F

##### Distribution

Armero-Guayabal, Ambalema, Casabianca, Cajamarca, Chaparral, Coyaima, Cunday, Dolores, Falan, Herveo, Honda, Ibagué, Lerida, Libano, Mariquita, Melgar, Prado, Purificación, Rio Blanco, San Antonio, San Luis, Venadillo, Villarrica

##### Notes

(v; ph ML123881471) M WD

#### Cathartes burrovianus

Cassin, 1845

390E30DF-2CE6-5300-8B24-87BE0DE74A79

##### Distribution

Armero-Guayabal, Alvarado, Coyaima, Doima, Ibagué, Mariquita, Piedras, San Luis

##### Notes

(v; ph ML118594551) EL

#### Pandion haliaetus

(Linnaeus, 1758)

7AD569CE-8085-538E-83FA-173B85E7DEDE

##### Distribution

Armero-Guayabal, Alvarado,Coyaima, Ibagué, Falan, Melgar, Piedras, Prado, Purificación, Venadillo

##### Notes

(v; co CZUTOR 0465; ph ML123286261) M

#### Gampsonyx swainsonii

Vigor, 1825

1E2832A5-80C5-56FE-87E9-9A59E36ECF2F

##### Distribution

Armero-Guayabal, Alvarado, Falan, Honda, Ibagué, Lerida, Mariquita, Melgar, San Luis, Venadillo

##### Notes

(v; co MHN-ICN 30736 v ph ML123287271)

#### Elanus leucurus

(Vieillot, 1818)

961EA6AB-B4F4-560B-89C9-43279DB65B3A

##### Distribution

Ataco, Armero-Guayabal, Alvarado,Chaparral, Coyaima, Cunday, Espinal, Falan, Honda, Ibagué, Lerida, Mariquita, Melgar, Purificación, Santa Isabel, San Luis, Venadillo

##### Notes

(v; co MLS 234, MHN-ICN 8723; ph ML123917361)

#### Chondrohierax uncinatus

(Temminck, 1822)

FB82A495-0A82-5F6F-853E-E91A8D9F7705

##### Distribution

Cajamarca, Falan, Libano, Purificación

##### Notes

(v; ph ML38703021). New record - in November 2015, a male individual was observed in the Municipality of Ibagué in a park near Jardín Botánico San Jorge (Botanical Garden); on 26 May 2017, May 2016 and 29 October 2018, an individual was observed in Piedecuesta (Falan). D. Johnston made a photographic record of an individual on 27 November 2012 (record in eBird). Photographic records are also known in Toche (Ibagué) and in Libano from the Santa Librada Reserve (pers. commun., M. Vallejo).

#### Leptodon cayanensis

(Latham, 1790)

50BD1CCB-C9F2-542A-99B9-F977EEF1ADA5

##### Distribution

Natagaima

##### Notes

(co MLS 244)

#### Elanoides forficatus

(Linnaeus, 1758)

A8AD161B-2680-5625-ACA4-2DB660633F06

##### Distribution

Ataco, Fresno, Ibagué, Natagaima, Libano, Planadas, San Antonio

##### Notes

(v, co; ph ML25998311) M. On 17 October 2008, a group of 10 individuals was observed and recorded migrating over the mountains of the north-western hills of Ibagué (Clarita Botero). On 5 May 2018, an individual was photographed at Finca Villa Laura (Ibagué) by J. Vargas (Reg. eBird). There are also records of this species in Lérida, Falan and Las Hermosas.

#### Harpia harpyja

(Linnaeus, 1758)

F1FAF60E-D21C-5399-A7E3-D57691BD6888

##### Distribution

Purificación

##### Notes

NT. Unspecified records in south of Tolima. Probably extinct in the region.

#### Spizaetus tyrannus

(Wied, 1820)

FAF059D7-BCC7-5666-B39D-05510C7B6C48

##### Distribution

Ibagué, Falan, Libano

##### Notes

(v; ph ML72669871). New record - there have been a high number of records of this species in the last three years in Tolima. This species is easily identified by its peculiar song and silhouette. Records were made in the municipalities of Ibagué (towards the north-western hills), Líbano, Falan, Casabianca and Palocabildo (Fig. 6c).

#### Spizaetus isidori

(Des Murs, 1845)

A7522C0D-F072-508D-A029-94B28FC42D31

##### Distribution

Ibagué, Chaparral, Libano, Villarrica

##### Notes

(v) NT

#### Busarellus nigricollis

(Latham, 1790)

05B86F9E-8C6E-55D1-BF58-74879C6B0F3C

##### Distribution

Armero-Guayabal, Ibagué, Venadillo

##### Notes

(v) One rec. A. Quevedo in Ibagué.

#### Rostrhamus sociabilis

(Vieillot, 1817)

7E440DA7-2BBD-55FF-A7BF-4183AC54D2BC

##### Distribution

Armero-Guayabal, Ambalema, Ibagué

##### Notes

(v; co; ph ML108050991) A few visual and photographic records were made in the Municipalities of Ibagué, Alvarado, Prado, Ambalema and Venadillo in 2010 with sightings below 1,000 m (photograph Figueroa W., Fig. 6a).

#### Harpagus bidentatus

(Latham, 1790)

FB5AAA84-B712-57E2-8DB9-5FC64592DE96

##### Distribution

Santa Isabel, Libano

##### Notes

(v)

#### Ictinia mississippiensis

(Wilson, 1811)

310D6915-D7B3-57DA-BC97-5565F8AC1321

##### Distribution

Armero-Guayabal, Ibagué, Libano, Venadillo, San Antonio

##### Notes

(v; ph ML118601361) M. A group of approximately 30 individuals was observed in migration on 17 October 2008, flying over the mountains of the north-western hills of Ibagué (Clarita Botero) with *Elanoides forficatus*. JF León observed and photographed five individuals in Venadillo on 8 April 2017. J. Castaño made records of this species in Girardot on 12 October 2016. Recently, on 20 October 2018, a group of 15 individuals was observed flying over the Municipality of Falan.

#### Ictinia plumbea

(Gmelin, 1788)

455CF79C-B415-52C3-8EDA-5860EEEF10E8

##### Distribution

Armero-Guayabal, Chaparral, Ibagué, Piedras

##### Notes

(v; ph)

#### Accipiter striatus

Vieillot, 1808

21D58B04-1B04-5F95-9327-3354B085DCC7

##### Distribution

Ibagué, Prado, Villarrica, San Antonio

##### Notes

(v; co ANSP 154990; ph ML123912051)

#### Astur bicolor

(Viellot, 1817)

88B56B20-DFFF-5CFE-A5E5-80050E823E20

##### Distribution

Alvarado, Ibagué, Piedras, Purificación, San Luis, San Antonio

##### Notes

(v; co AMNH 132205). An uncommon species, it was observed in Totumo (900 m) in riparian forest surrounding a lagoon on 9 July 2017. Another record was made on 30 June 2017 in the Lagoon La vieja San Luis, in riparian forest and near the river. Another record was made by M. Cardona in Herveo on 23 April 2018 (registered in eBird).

#### Circus hudsonius

(Linnaeus, 1766)

4711BC32-3876-5BAE-8221-B7C1B1A716B5

##### Distribution

Villahermosa

##### Notes

(co MHN-ICN 8699)

#### Circus buffoni

(Gmelin, 1788)

B3617605-39C5-5B77-B034-611FE8E04AB7

##### Distribution

Armero-Guayabal, Ibagué

##### Notes

(v; ph ML63189841) EL. New record - in recent years, there have been several records of this species in Tolima. The first record corresponds to an individual observed perched on a fence in a rice field in Guayabal on 4 May 2009. On 8 August 2015, a female was recorded flying over the rice fields of Picaleña. On 21 July 2017, an individual was also photographed (Fig. 6b).

#### Microspizias collaris

Sclater, 1860

71618EFB-CF30-5441-A5DF-85A284A1C32A

##### Distribution

Ataco, Honda, Planadas, San Luis

##### Notes

(v; co MHN-ICN 685, NMNH 81887)

#### Geranospiza caerulescens

(Vieillot, 1817)

DF28A8F0-AEF2-54C6-9E40-E0B4D38111D9

##### Distribution

Armero-Guayabal, Ambalema, Espinal, Ibagué, Piedras, Falan

##### Notes

(v; co MLS 429; ph ML123293041)

#### Buteogallus anthracinus

(Deppe, 1830)

0C8BA97F-1461-5197-A7AE-1B97945B86B8

##### Distribution

Honda, Ibagué

##### Notes

(v) Several visual records in low areas.

#### Buteogallus meridionalis

(Latham, 1790)

2F9FB625-8792-59D0-84B7-7C4345AB7DBC

##### Distribution

Armero-Guayabal, Alvarado, Ambalema, Guamo, Espinal, Falan, Ibagué, Piedras, Purificación, Saldaña

##### Notes

(v; co MLS 31, WFVZ SS-20405; ph ML111644761)

#### Buteogallus urubitinga

(Gmelin, 1788)

1922C542-96DD-5CEC-8055-7C053DDBE9E5

##### Distribution

Falan

##### Notes

(v)

#### Buteogallus solitarius

(Tschudi, 1844)

62E9EE8D-DFD2-521E-BD2A-D373EE422005

##### Distribution

Ibagué, Murillo

##### Notes

(v; co CZUTOR 1423). New record - two visual records are known for this species in Tolima. One corresponds to a perched individual observed in October 2017 by D. Carantón in the path Juntas (pers. commun., D. Carantón). The second observation is a pair observed flying over Villa Restrepo on 13 October 2016 by J. Castaño. Another individual was collected in Murillo and deposited into the ornithology collection of the University of Tolima with catalogue OR 1423 on 18 November 2015 (J. Gil and N. Vega).

#### Morphnarchus princeps

(Sclater, 1865)

FAC1F580-7E07-5F39-BE68-6BCD845FC4BD

##### Distribution

Ibagué, Villarrica

##### Notes

(ph; v). This uncommon species was observed on 16 November 2004 in the Laguna Venta Quemada (Alvarado) and in the Vereda Agua de Dios, Casabianca on 9 July 2019. H. Arias registered it in the urban area of Ibagué and J. Poveda registered it in the Agroecological Reserve Santa Librada on 22 October 2014 (Fig. 12b).

#### Rupornis magnirostris

(Gmelin, 1788)

DD7A3560-F75F-5E2C-A68B-8B48E2FEEF8D

##### Distribution

Armero-Guayabal, Alvarado, Ambalema, Anzoátegui, Ataco, Casabianca, Cajamarca, Chaparral, Carmen de Apicala, Coyaima, Cunday, Dolores, Espinal, Falan, Fresno, Guamo, Honda, Ibagué, Lerida, Libano, Mariquita, Murillo, Planadas, Prado, Purificación, Rio Blanco, Santa Isabel, San Antonio, San Luis, Venadillo, Villarrica, Villahermosa

##### Notes

(v; au XC424599; co MLS 328, CZUTOR 0622; ph ML115211821) WD

#### Parabuteo unicinctus

(Temminck, 1824)

321DAAC5-C397-5530-8D0F-84AA5F272C6E

##### Distribution

Ibagué

##### Notes

(v; ph ML 194699361). A few records of this species have been registered in the area. An individual was observed perched on a tree on 11 July 2005. On 12 July 2006, an individual was observed in an area near the Perales de Ibagué Airport, in a forest relic near a lagoon.

#### Parabuteo leucorrhous

(Quoy & Gaimard, 1824)

8D3EBD49-809A-54BB-B096-BD9BBA305814

##### Distribution

Falan, Ibagué, Líbano

##### Notes

(v; co, ph ML 247144681). There are various records of this species in the Department. On 19 November 1989, Peter Kaestner recorded it in Toche. This species has also been registered in Juntas (Ibagué), Piedecuesta (Falan) and Polca (Líbano).

#### Gerenoaetus albicaudatus

(Vieillot, 1816)

A96C112A-43D9-5F7B-93DF-45348FF4DF9E

##### Distribution

Armero-Guayabal, Ibagué, Mariquita, Melgar, San Antonio, Santa Isabel, San Luis, Venadillo

##### Notes

(v; co MHN-ICN 728; ph ML115212841)

#### Gerenoaetus polyosoma

(Quoy & Gaimard, 1824)

EFF11B85-B788-5730-99CC-AB6394EC19AF

##### Distribution

Cajamarca, Ibagué, Murillo, Santa Isabel

##### Notes

(v; co AMHN 111403)

#### Geranoaetus melanoleucus

(Vieillot, 1819)

A8614833-47B3-5A5B-B378-55455D4B0663

##### Distribution

Cajamarca, Ibagué, Murillo

##### Notes

(v; ph ML78851871)

#### Buteo nitidus

(Latham, 1790)

051A4E6A-6C2B-547A-AB91-A715178F4C2E

##### Distribution

Armero-Guayabal, Alvarado, Chaparral, Falan, Mariquita, Melgar, Ibagué, Planadas, San Antonio

##### Notes

(V; co ICN 10866; ph ML123829971)

#### Buteo platypterus

(Vieillot, 1823)

5DCF3A09-7083-56F0-A412-3D0822876FEE

##### Distribution

Ataco, Cajamarca, Ibagué, Falan, Fresno, Honda, Lerida, Libano, Murillo, Planadas, San Luis, San Antonio

##### Notes

(V; ca; co MHN-ICN 13329, CZUTOR 0221; ph ML84679171) M

#### Buteo albigula

Philippi, 1899

5CB388B6-A81C-5459-A981-B3EACB317B12

##### Distribution

Ibagué

##### Notes

(v; ph ML115212321)

#### Buteo brachyurus

Viellot, 1816

5F207020-1008-5896-A938-5D4799AB3EB1

##### Distribution

Armero-Guayabal, Cajamarca, Chaparral, Ibagué, San Antonio, San Luis

##### Notes

(v; ph ML107748731)

#### Buteo swainsoni

Bonaparte, 1838

852A8AD2-9487-5AC4-9FB1-F89CF3BCA09E

##### Distribution

Cunday, Espinal, Guamo, Honda, Ibagué, Falan, Lerida, Murillo, San Antoni

##### Notes

(v; co MHN-ICN 8367; ph ML47562641) M

#### Buteo jamaicensis

Gmelin, 1788

44B2B1E6-6E53-5546-8790-D4375AA13D56

##### Distribution

Ibagué

##### Notes

(v) M. New record - this is an unusual species that was observed on 25 January 2010, migrating at a low altitude of 900 m a.s.l. in a dry forest zone of the Municipality of Ibagué near the canyon of the Combeima River. Individuals were flying low and were identified by the red tail and black subterminal on the tail.

#### Tyto furcata

(Temminck, 1827)

F4A015C0-DB95-51F1-A6C1-BDB93F815437

##### Distribution

Anzoátegui, Alvarado,Espinal, Herveo, Honda, Falan, Ibagué, Libano, Murillo, Prado, Purificación, Villahermosa, Villarrica

##### Notes

(v; co MLS 1334; ph)

#### Megascops albogularis

(Cassin, 1849)

54285390-3D98-563C-886E-85AFB07FC655

##### Distribution

Ibagué, Roncesvalles, Murillo, San Antonio

##### Notes

(v; au ML 233621091; co)

#### Megascops choliba

(Vieillot, 1817)

17DDAADC-D2B8-5FF2-97D3-4BEACD6E7D4C

##### Distribution

Armero-Guayabal, Casabianca, Chaparral, Coyaima, Espinal, Falan, Natagaima, Herveo, Honda, Ibagué, Lerida, Libano, Mariquita, Planadas, Prado, Purificación, San Antoni, Villarrica

##### Notes

(v; au; co MPUJ 733, CZUTOR 213, MLS 1360; ph ML27840031)

#### Lophostrix cristata

(Daudin, 1800)

5AE4C4A0-43C1-5D9E-B6F6-F8BE9B73CDED

##### Distribution

Chaparral

##### Notes

(v) M. Moreno

#### Pulsatrix perspicillata

(Latham, 1790)

14B823AC-4333-513F-93DB-8A79975E9474

##### Distribution

Ambalema, Honda, Villahermosa

##### Notes

(au; ca)

#### Bubo virginianus

(Gmelin, 1788)

4FC984EC-1781-5FD7-B4CA-79ECD52BACF2

##### Distribution

Espinal, Ibagué, Purificación

##### Notes

(v; co MLS 7773)

#### Strix virgata

(Cassin, 1849)

B52BDB45-4475-528D-B578-0A14CD9A3965

##### Distribution

Cajamarca, Falan, Ibagué, Icononzo, Libano, San Antonio

##### Notes

(v; au ML81694 co ANSP 155086, CZUTOR 0178; ph ML79778021)

#### Strix nigrolineata

Sclater, 1859

FBF347C5-1C4F-5ACE-82AD-B835F986AE3E

##### Distribution

Coyaima Ibagué, Purificación, Saldaña

##### Notes

(co MLS 1411; ph ML 240304101)

#### Strix albitarsis

(Bonaparte, 1850)

065208C2-E280-5AD0-8623-D0C5164561AE

##### Distribution

Cajamarca, Ibagué

##### Notes

(v; co ANSP 155084)

#### Glaucidium jardinii

(Bonaparte, 1855)

C5B8D98E-DD7D-5DB4-A672-97D2C4AE991F

##### Distribution

Cajamarca, Ibagué

##### Notes

(au ML 198292011; ph ML 37878561)

#### Glaucidium brasilianum

(Gmelin, 1788)

00ACE321-5AA7-5053-A5B5-EAC94E4A1B97

##### Distribution

Honda, Ibagué

##### Notes

(co ANSP 154698)

#### Athene cunicularia

(Molina, 1782)

24490FAC-2D0A-5188-B210-E0B32FD85241

##### Distribution

Armero-Guayabal, Ambalema, Coyaima, Ibagué, Guamo, Honda, Saldaña, San Luis, Saldaña

##### Notes

(v; co MLS 8324; ph ML98822281)

#### Aegolius harrisii

(Cassin, 1849)

463F17B9-D081-5539-868D-0341A8FF0974

##### Distribution

Ibagué

##### Notes

(v)

#### Asio clamator

(Vieillot, 1807)

A122F079-6EFA-5F74-95F4-7BAC11B44A28

##### Distribution

Armero-Guayabal, Cajamarca, Espinal, Falan, Honda, Ibagué, Lerida, Mariquita, Planadas, Venadillo

##### Notes

(v; ca; co MLS 1424, CZUT-OR1343)

#### Asio stygius

(Wagler, 1832)

8D1711DD-652D-584F-B1ED-BC8B53F9C598

##### Distribution

Ibagué, Espinal, Guamo, Fresno

##### Notes

(v; ca; co MLS 3169, CZUTOR 251; ph ML24063731)

#### Asio flammeus

(Pontoppidan, 1763)

E0753691-B2E3-5283-B2D8-103D0BF19820

##### Distribution

Ibagué

##### Notes

(v; A photographic record at the border with caldas in Nevado del Ruiz ML 133046581).

#### Pharomachrus auriceps

(Gould, 1842)

E1D5841A-2D60-5586-8F53-83920CA014F4

##### Distribution

Ibagué, Planadas, Roncesvalles, San Antonio

##### Notes

(V; au; co SIB 5959; ph ML78998721)

#### Pharomachrus antisianus

(d'Orbigny, 1837)

C914610D-0ECB-5B6A-9A7C-CF68B2F94FF7

##### Distribution

Ataco, Ibagué, Libano, Murillo, San Antonio

##### Notes

(co MHN-ICN 9128)

#### Trogon melanurus

Swainson, 1838

4A82EAC3-F3A6-5AAF-849B-A6647E029B2A

##### Distribution

Honda, Icononzo

##### Notes

(co)

#### Trogon chionurus

Sclater & Salvin, 1870

2C69C1FC-AF18-523B-A2AF-ED3140B78980

##### Distribution

Honda, Mariquita

##### Notes

(v; co AMNH-SKIN 94848) Several searches at the border with the Department of Caldas between Victoria and Albania.

#### Trogon viridis

Linnaeus, 1766

63BBE242-F854-5C06-8064-168EC1C0730D

##### Distribution

Honda

##### Notes

(co AMHN 94848)

#### Trogon rufus

Gmelin, 1788

A6E72FEC-AEDE-5CB9-812F-D23F31FC9413

##### Distribution

Honda

##### Notes

(co)

#### Trogon collaris

Vieillot, 1817

8589D9AB-F4F8-56D8-A9F1-48FF44B5223A

##### Distribution

Cajamarca, Ibagué, Honda, Planadas, San Antonio, Santa Isabel

##### Notes

(v; au ML 99165581; co ANSP 154666; ph ML99188281)

#### Trogon personatus

Gould, 1842

B290965D-7104-5B83-9DD0-9CD069BA8A0D

##### Distribution

Anzoátegui, Cajamarca, Falan, Ibagué, Icononzo, Murillo, San Antonio, Santa Isabel

##### Notes

(v; au XC421113; co ANSP 35272; ph ML115212921)

#### Baryphthengus martii

(Spix, 1824)

D57ACBD6-AB24-56D1-9A1D-033A82B9F822

##### Distribution

Ambalema, Falan, Honda, Mariquita

##### Notes

(v; co MHNP 1889)

#### Momotus subrufescens

Sclater, 1853

C996D981-BFE0-5305-AA65-17AAD2690499

##### Distribution

Armero-Guayabal, Ambalema, Casabianca, Chaparral, Carmen de Apicala, Coyaima, Espinal, Falan, Guamo, Lerida, Libano, Honda, Ibagué, Mariquita, Santa Isabel, San Luis, Venadillo

##### Notes

(v; au XC384417; ca; co MLS 261, CZUTOR 717; ph ML99192751)

#### Momotus aequatorialis

Gould, 1858

47A7C7F3-99F5-5CB5-AF4F-7F00B4170E75

##### Distribution

Anzoátegui, Ataco, Casabianca, Carmen de Apicala, Espinal, Falan, Guamo, Lerida, Libano, HerVenadilloo, Honda, Ibagué, Rio Blanco, Santa Isabel, San Luis, Venadillo, San Antonio

##### Notes

(v; au XC425859; co MLS 267, CZUTOR 474; ca; ph ML72670341)

#### Megaceryle torquata

(Linnaeus, 1766)

26BB0E09-4A58-5EE1-A034-D1B4093C2E6C

##### Distribution

Armero-Guayabal, Cunday, Espinal, Falan, Honda, Ibagué, Mariquita, Melgar, Prado, Purificación, Roncesvalles, Venadillo

##### Notes

(v; au; co MLS 2543, CZUTOR 812; ph ML120513441)

#### Chloroceryle amazona

(Latha, 1790)

399296A8-7EA7-574E-9EBB-83B935EB35FA

##### Distribution

Armero-Guayabal, Chaparral, Coyaima, Cunday, Guamo, Espinal, Falan, Honda, Ibagué, Mariquita, Prado, Purificación, San Luis

##### Notes

(v; au; co MLS 2553, CZUTOR 202; ph ML110076711)

#### Chloroceryle aenea

(Pallas, 1764)

D51A936F-70CD-5B59-83DB-39DAE171A200

##### Distribution

Armero-Guayabal, Ambalema, Espinal, Honda

##### Notes

(v; ca; co MLS 2587)

#### Chloroceryle americana

(Gmelin, 1788)

2ABE3BD9-CAC4-5C41-A5CE-CB0A214CCAA5

##### Distribution

Armero-Guayabal, Chaparral, Coyaima, Espinal, Falan, Gualanday, Guamo, Honda, Ibagué, Lerida, Mariquita, Ortega, Prado, Purificación, San Luis

##### Notes

(v; au; co MLS 2571, CZUTOR 0105; ph ML98326261)

#### Galbula ruficauda

Cuvier, 1816

B174B231-5C87-52C2-81C6-619CB0EA727F

##### Distribution

Armero-Guayabal, Ambalema, Carmen de Apicala, Chaparral, Coyaima, Cunday, Espinal, Guamo, Gualanday, Honda, Ibagué, Falan, Natagaima, Mariquita, Prado, Purificación, Saldaña, Venadillo, Villarrica

##### Notes

(v; au XC420247; co MLS 2701, CZUTOR 262; ph ML123187971; ca;)

#### Notharchus hyperhynchus

(Sclater, 1856)

64C81BF3-14E9-542A-847B-4FB545646D88

##### Distribution

Ambalema, Coyaima, Espinal, Honda, Prado, San Luis

##### Notes

(co MLS 2732)

#### Notharchus tectus

(Boddaert, 1783)

E425F051-344F-5A40-91D4-BC9F0388C4C8

##### Distribution

Espinal, Mariquita, Honda

##### Notes

(co NHMA 73190)

#### Nystalus radiatus

(Sclater, 1854)

946BDB28-CA42-552F-9025-FB55BC01B093

##### Distribution

Ambalema, Carmen de Apicala, Coyaima, Espinal, Falan, Honda, Ibagué, Mariquita, Melgar, Purificación, Saldaña, Venadillo

##### Notes

(v; co MLS 8274; ph ML123875481)

#### Hypnelus ruficollis

(Wagner, 1829)

7C471DFD-9F32-55B0-9651-514C68A9D622

##### Distribution

Armero-Guayabal, Falan, Libano, Mariquita, Natagaima, Honda

##### Notes

(v; co CZUTOR 0944; ph ML123914791)

#### Malacoptila mysticalis

(Lafresnayi, 1850)

96EA43DB-6F88-585B-970B-B9A20CF4E318

##### Distribution

Falan, Fresno, Honda, Ibagué, Prado, San Antonio

##### Notes

(v; ca; co AMHN 94885; ph ML123915971)

#### Monasa morphoeus

(Hahn & Kuster, 1823)

D940E783-55FA-57A6-9826-1D0017B7478C

##### Distribution

Honda

##### Notes

(co MCZ 124974)

#### Capito hypoleucus

Salvin, 1897

737A003B-6DB7-5285-9EF7-303BF21BAFFB

##### Distribution

Falan, Fresno, Honda, Libano, Mariquita

##### Notes

(v; co AMHN 94855; ph ML99183031) EN E. This endemic species is permanently registered in the north of Tolima in Líbano, Falan, Fresno and Mariquita. It is frequently identified by its song and is commonly observed in pairs, especially in established secondary forest and with the presence of yarumos (*Cecropia* sp.) (Fig. 9d).

#### Eubucco bourcierii

(Lafresnaye, 1845)

38D40A33-75CB-5F5B-ABBC-FDD0ABE2C6E7

##### Distribution

Ataco, Cunday, Dolores, Falan, Icononzo, Planadas, Villarrica

##### Notes

(v; ca; co MHN-ICN 24913, CZUT OR0887; ph)

#### Ramphastos ambiguus

(Swinson, 1823)

D7AF94B2-D40E-5C29-A4BF-6A288BD3EF4B

##### Distribution

Ataco

##### Notes

(co MHN – ICN; ph ML 99154131)

#### Ramphastos vitellinus

Litchtenstein, 1823

98B1661A-7606-54B2-ACBC-62738B045F6A

##### Distribution

Honda, Mariquita

##### Notes

(co AMNH 94859)

#### Aulacorhynchus albivitta

(Gold, 1833)

358B1768-7445-5366-956B-EEEBC78DAB44

##### Distribution

Anzoátegui, Ataco, Casabianca, Cajamarca, Dolores, Falan, Herveo, Ibagué, Libano, Murillo, Planadas, San Antonio, Santa Isabel, Villahermosa, Villarrica

##### Notes

(v; au-XC95833; co MHN-ICN 11286, CZUTOR 688; ph ML123892881)

#### Aulancorhynchus haematopygus

(Gould, 1835)

F4C367FB-618D-5BD9-ABCF-69A6A48505E6

##### Distribution

Ataco, Falan, Ibagué, Honda, San Antonio, Villarrica

##### Notes

(v; co CZUTOR 919; ph ML 158776161)

#### Andigena hypoglauca

(Gould, 1833)

C68D029E-16BC-5B9D-9820-20AE87CA9196

##### Distribution

Anzoátegui, Cajamarca, Ibagué, Murillo, Planadas, Roncesvalles, Santa Isabel

##### Notes

(v; au; co ML 126300; ph ML85221791) NT

#### Andigena nigrirostris

(Waterhouse, 1839)

7AF70DE5-B251-598B-8C86-04EC505F0480

##### Distribution

Cajamarca, Ibagué, Murillo, Roncesvalles, San Antonio

##### Notes

(v; au XC426296; co ML 126303; ph ML115212081)

#### Pteroglossus torquatus

(Gmelin, 1788)

47681848-6DA5-5CC1-9E6B-3469BF237F74

##### Distribution

Armero-Guayabal, Honda

##### Notes

(v; co AMHN 94862; ph ML28756191)

#### Picumnus olivaceus

Lafresnaye, 1845

1FE1F45A-0A87-5707-9DD6-14046CC86B22

##### Distribution

Armero-Guayabal, Chaparral, Cajamarca, Carmen de Apicala, Dolores, Espinal, Guamo, Falan, Honda, Ibagué, Lerida, Mariquita, Melgar, Planadas, San Antonio, San Luis, Venadillo, Villarrica

##### Notes

(v; au XC424740ca; co MLS 3249, CZUTOR 142; ph ML123909111) WD

#### Melanerpes formicivorus

(Swainson, 1827)

C5CBCAEA-A12F-5CA4-B76E-C973D767ADD3

##### Distribution

Anzoátegui, Ataco, Chaparral, Dolores, Herveo, Ibagué, Libano, Murillo, Planadas, Roncesvalles, San Antonio

##### Notes

(v; au XC96320; co MHN-ICN 1568; ph ML123912431)

#### Melanerpes pulcher

Sclater, 1870

4AA139CB-D083-5CAA-BD2D-6E949EFC61A6

##### Distribution

Honda, Falan, Libano

##### Notes

(v; co AMHN 94899)

#### Melanerpes rubricapillus

(Cabanis, 1862)

7F0CA1A1-99F8-5985-BA77-4528627CBC33

##### Distribution

Armero-Guayabal, Alvarado, Ambalema, Ataco, Cajamarca, Chaparral, Coyaima, Cunday, Dolores, Espinal, Honda, Ibagué, Falan, Lerida, Libano, Mariquita, Melgar, Planadas Purificación, Rio Blanco, San Antonio, San Luis, Venadillo

##### Notes

(v; au; ca; co MLS 315, CZUTOR 129; ph ML123938441) WD

#### Dryobates fumigatus

(d'Orbigny, 1840)

741E3765-8947-5E3B-9535-71C2B2B89B6E

##### Distribution

Ataco, Falan, Ibagué, Icononzo, Planadas, San Antonio

##### Notes

(v; co MHN-ICN 1665; ph ML85278961)

#### Dryobates kirkii

(Malherbe, 1845)

FFEB30B6-B298-5A21-A1AF-1594F552DAD8

##### Distribution

Armero-Guayabal, Chaparral, Coyaima, Dolores, Espinal, Ibagué, Gualanday, Guamo, Falan, Honda, Lerida, Mariquita, Planadas, Prado, Purificación, San Antonio, San Luis, Venadillo Villarrica

##### Notes

(v; ca; co MLS 3198, CZUTOR 98; ph ML98795211)

#### Dryobates dignus

(Sclater & Salvin, 1877)

1B8DC898-4212-506A-B4EA-26D8D4B6F97F

##### Distribution

Espinal, Herveo, Ibagué, Planadas, San Antonio, Santa Isabel

##### Notes

(v; ph ML99043281)

#### Dryobates nigriceps

(d'Orbigny, 1840)

FF3EA450-081D-5DC0-A17B-B85E60F1FC9E

##### Distribution

Cajamarca, Ibagué, San Antonio, Santa Isabel

##### Notes

(v; co ANSP 155073; ph ML98890091)

#### Campephilus pollens

(Bonaparte, 1845)

6D3B06DD-1F00-5D4E-9DF5-E3D367216A11

##### Distribution

Casabianca, Falan, Ibagué, Libano, Murillo, Planadas, San Antonio, Santa Isabel, Villahermosa, Villarrica

##### Notes

(v; ph ML25981251)

#### Campephilus melanoleucos

(Gmelin, 1788)

1F9C5737-6DBE-5670-B40A-4AA268B711AC

##### Distribution

Armero-Guayabal, Anzoátegui, Falan, Honda, Ibagué, Santa Isabel, Villarrica

##### Notes

(v; co AMHN 94901; ph)

#### Dryocopus lineatus

(Linnaeus, 1766)

03816495-694C-5CAA-B637-F9C2443545CB

##### Distribution

Armero-Guayabal, Ataco, Cunday, Falan, Honda, Ibagué, Piedras, Prado, Rio Blanco, San Antonio, Villarrica

##### Notes

(v; au XC424647; co AMHN 94902 ph ML47582601)

#### Colaptes rubiginosus

(Swainson, 1820)

B4FC510C-DA3A-5879-819A-4CC9A385CCF6

##### Distribution

Anzoátegui, Casabianca, Falan, Herveo, Honda, Ibagué, Libano, Murillo, Planadas, San Antonio, Villarrica

##### Notes

(v; au ML98598481; co; ph ML123911261)

#### Colaptes rivolii

(Boissonneau, 1840)

3C5B8AE8-7AC0-537E-9D9D-31326FF919DD

##### Distribution

Anzoátegui, Cajamarca, Ibagué, Murillo, Planadas, San Antonio, Santa Isabel

##### Notes

(v; au-XC96279; ca; co CZUTOR 058; ph ML123901241)

#### Colaptes punctigula

(Boddaert, 1783)

6A07BEE5-2EF6-5407-99A4-F4BDF9BFB4FE

##### Distribution

Armero-Guayabal, Alvarado, Ataco, Coyaima, Cunday, Espinal, Falan, Fresno, Guamo, Honda, Ibagué, Lerida, Libano, Mariquita, Melgar, Murillo, Venadillo, Villarrica

##### Notes

(v; au XC420375; ca; co CZUTOR 490, MLS 397; ph ML123907681)

#### Herpetotheres cachinnans

(Linnaeus, 1758)

0C66695E-21EB-5318-9794-897829000232

##### Distribution

Armero-Guayabal, Chaparral, Carmen de Apicala, Coyaima, Espinal, Honda, Ibagué, Falan, Mariquita, San Luis, Venadillo, San Antonio

##### Notes

(v; au-XC335012 co MLS 436, MHN-ICN 13236; ph ML123896221)

#### Micrastur ruficollis

(Vieillot, 1817)

D14AD522-99ED-5D2C-AE8B-E3EE25A30493

##### Distribution

Anzoátegui, Espinal, Falan, Ibagué, Libano, San Antonio

##### Notes

(v; au; ca; co CZUTOR 600; ph). There are a few records of this species in the Department. An adult was collected (CZUT-OR - 600) on 18 February 2007 in the mature forest on the Ambala, in the north-western hills (Molina-Martínez 2014). On 19 February 2017, an individual was identified by vocalisation in La Mariposa (Ibagué). Other records from other authors were made in Ibagué and Líbano (registered in eBird).

#### Micrastur semitorquatus

(Vieillot, 1817)

32971CC4-C9CF-58D8-B332-34B1FEDDBAC4

##### Distribution

Armero-Guayabal, Ibagué

##### Notes

(au ML58233971) EL. Probably all over Tolima below 1000 m a.s.l. A few records have been made in Tolima. On 10 February 2007, an individual was observed by the Tolima Bird Observer Group (GOAT) in Piedras. D. Carantón observed an individual in the Tabares Hacienda, Vereda Tavera on 13 April 2016. We observed an individual in Totumo and Salado on 9 July 2017 and 30 September 2017, respectively.

#### Caracara plancus

(Jacquin, 1784)

E80A1381-32CB-52CA-8568-EC8A8DA80096

##### Distribution

Alvarado, Armero-Guayabal, Anzoátegui, Chaparral, Cajamarca, Coyaima, Dolores, Guamo, Purificación, Honda, Ibagué, Falan, Libano, Mariquita, Murillo, Prado, Roncesvalles, San Antonio, San Luis, Venadillo

##### Notes

(v; au; ph ML111644711) WD

#### Phalcobaenus carunculatus

Des Murs, 1853

3F945F22-B24D-574D-8397-F78D881196C4

##### Distribution

Cajamarca, Ibagué, Roncesvalles

##### Notes

(v; ph ML30790131) CE

#### Milvago chimachima

(Viellot, 1816)

2378A727-4F17-5DFC-9FA6-8C12EEB9ED8D

##### Distribution

Armero-Guayabal, Alvarado,Ambalema, Carmen de Apicala, Cajamarca, Chaparral, Cunday, Coyaima, Dolores, Espinal, Falan, Guamo, Honda, Ibagué, Mariquita, Murillo, Prado, Purificación, Rio Blanco, San Antonio, San Luis, Venadillo, Villarrica

##### Notes

(v; au; co MLS 467, MHN-ICN 13225; ph ML110073261) WD

#### Falco sparverius

Linnaeus, 1758

F99B6443-7820-58EF-98BA-D6107E67616A

##### Distribution

Armero-Guayabal, Alvarado, Ataco, Cajamarca, Carmen de Apicala, Coyaima, Cunday, Espinal, Guamo, Honda, Ibagué, Libano, Murillo, Natagaima, Planadas, Purificación, Santa Isabel, San Luis, Roncesvalles, San Antonio, Venadillo, Villahermosa, Villarrica

##### Notes

(v; au; co MLS 500, WFVZ SS-20731; ph ML109291181) WD

#### Falco columbarius

Linnaeus, 1758

19763A42-D872-5D3E-AFEF-69CC7B2FDCEF

##### Distribution

Cajamarca, Coyaima, Cunday, Ibagué, Libano, Melgar, Villarrica

##### Notes

(v; ph ML 149038251) M

#### Falco rufigularis

Daudin, 1800

0151179D-5A1D-5276-98A6-AF3B1897A7BE

##### Distribution

Armero-Guayabal, Guamo, Ibagué, Honda, Libano, Mariquita, San Luis

##### Notes

(v; au XC423694; co MLS 472)

#### Falco deiroleucus

Temminck, 1825

E11A8DF0-7E4C-57D7-9AC8-2D24D0BA01C3

##### Distribution

Purificación

##### Notes

(co MLS 471)

#### Falco femoralis

Temminck, 1822

A7A844A2-46FE-553D-B007-2734454BA841

##### Distribution

Armero-Guayabal, Guamo, Ibagué, Mariquita, Purificación, San Antonio, San Luis

##### Notes

(v; co MLS 483; ph ML35419481)

#### Falco peregrinus

Tunstall, 1771

1635E64F-4436-5647-B593-056A7900528C

##### Distribution

Ibagué, San Luis

##### Notes

(v; ph ML75626811) M

#### Touit stictopterus

P. L. Sclater, 1862

D9FFF9B5-59B6-53DC-BCFD-4CACEC2212E0

##### Distribution

San Antonio

##### Notes

(v; ph ML 251432391) New record - This unusual species in the Department was observed for the first time on 20 July 2020 in Reserva Natural Finca El Manantial (Parra-Hernández et al. 2022).

#### Bolborhynchus lineola

(Cassin, 1853)

4605D71F-92B1-5DAC-B738-C056537DD40D

##### Distribution

Ibagué, Cajamarca, Libano

##### Notes

(v; au). New record - this parrot usually goes unnoticed in Tolima. We observed it in Cajamarca (Anaime) flying over 2400 m a.s.l. on 23 September 2016. In the Municipality of Líbano, in the locality called La Trigrera, we observed several groups of 5 - 10 individuals in January 2011 and December 2017.

#### Bolborhynchus ferrugineifrons

(Lawrence, 1880)

EA63CF28-6FA0-5E3A-9889-30F143DAAC24

##### Distribution

Anzoátegui, Casabianca, Ibagué, Libano, Santa Isabel

##### Notes

(v; au ML90032081; co SIB A6800; ph ML58185821) E VU. This endemic parrot was observed in the páramo zone, in the Municipalities of Murillo and permanently in the Cabaña (pers. commun., Yuli Rincón) in groups of 8 - 25 individuals. It has also been registered in Anzoátegui, Ibagué and other páramo zones of the Department (Fig. 8d).

#### Brotogeris jugularis

(Statius Muller, 1776)

51E4E27B-D1B7-573A-8540-18A82F9E8F39

##### Distribution

Armero-Guayabal, Alvarado, Ataco, Casabianca, Chaparral, Carmen de Apicala, Cunday, Dolores, Espinal, Falan, Honda, Ibagué, Falan, Lerida, Libano, Mariquita, Melgar, Planadas, Prado, Purificación, Rio Blanco, San Antonio, San Luis, Venadillo

##### Notes

(v; au XC384416; ca; co MLS 1171; ph ML83887931) WD

#### Hapalopsittaca amazonina

(Des Murs, 1845)

8125E128-4E70-562B-A3BC-B3E0C78591A1

##### Distribution

Anzoátegui, Planadas, Roncesvalles

##### Notes

(v) E VU. This parrot can be seen in relics of forest and forests of Roncesvalles, Anzoátegui and Murillo at 2700 to 3000 m a.s.l..

#### Hapalopsittaca fuertesi

(Chapman, 1912)

F179F8EF-3533-5F89-B06A-7A678AD4FAD4

##### Distribution

Cajamarca, Ibagué, Roncesvalles, Santa Isabel

##### Notes

(v; ph https://www.inaturalist.org/photos/29051444) E CR. There are several recent records for this endemic parrot. These records include new locations for the species in Colombia. Since 2015, it has been observed in Fuertes's Parrot Bird Reserve (Cajamarca), with a photographic record from Stephan Lorens in eBird (ML 148379031). In June 2015, it was also observed in the Reserva Semillas de Agua (Cajamarca). J. Sanabria registered it in the Municipality of Ibagué near the Combeima River Canyon on 3 August 2017 and in Tochecito on 5 May 2018.

#### Pyrilia pyrilia

(Bonaparte, 1853)

DF9B7243-D2C3-5F3A-9605-42B89B4E298F

##### Distribution

Casabianca, Villarricallahermosa, Villarrica

##### Notes

(v, au)

#### Pionus tumultuosus

(Tschudi, 1844)

62B22E07-DC16-5A81-BD6E-39AFFA0C4C13

##### Distribution

Falan, Ibagué, Planadas, Prado, Villarrica

##### Notes

(v; au ML97307511; co ANSP 155096; ph ML23771431)

#### Pionus menstruus

(Linnaeus, 1766)

AC247712-D8F8-50B1-8B15-40EE0BA7012E

##### Distribution

Armero-Guayabal, Ambalema, Alvarado, Chaparral, Honda, Ibagué, Falan, Mariquita, San Antonio, Villarrica

##### Notes

(v; au; co; ph ML35418741)

#### Pionus chalcopterus

(Fraser, 1841)

0D82D2C0-8981-59E0-9B5F-F1C6AF08910C

##### Distribution

Ataco, Cajamarca, Ibagué, Libano, Planadas, Rio Blanco, San Antonio

##### Notes

(v; au; co MHN-ICN 10832; ph ML49801261)

#### Amazona ochrocephala

(Gmelin, 1788)

826BDD86-ED74-50FD-862F-7F9C560FC84B

##### Distribution

Armero-Guayabal, Chaparral, Cunday, Falan, Honda, Ibagué, Mariquita, Prado, San Antonio, Venadillo, Villarrica

##### Notes

(v; au XC425945; co AMHN 121484; ph ML119357191)

#### Amazona amazonica

(Linnaeus, 1766)

381ACC76-626C-5D5C-B78F-11EE0C5D0039

##### Distribution

Falan, Ibagué, Santa Isabel, Venadillo, Piedras

##### Notes

(v; ph ML 167041761) INT

#### Amazona mercenarius

(Tschudi, 1844)

CC9BEBBA-A162-5898-ABE5-621036FBDDA6

##### Distribution

Anzoátegui, Ataco, Cajamarca, Ibagué, Santa Isabel

##### Notes

(v; au; co MHN-ICN 10696; ph ML22279591)

#### Forpus conspicillatus

(Lafresnaye, 1848)

110F048F-717F-5643-AEA8-504A91904EB1

##### Distribution

Armero-Guayabal, Ambalema, Alvarado, Casabianca, Chaparral, Carmen de Apicala, Coyaima, Cunday, Dolores, Espinal, Falan, Honda, Ibagué, Icononzo, Fresno, Lerida, Libano, Mariquita, Planadas, Prado, Purificación, Rio Blanco, San Antonio, San Luis, Venadillo, Villarrica

##### Notes

(v; au XC386426; ca; co MLS 1117, CZUTOR 009; ph ML117706921) WD

#### Pyrrhura melanura

(Spix, 1824)

B1C2C58D-94AD-504D-830A-5F9C7593D5A0

##### Distribution

Ataco, Planadas, Villarrica

##### Notes

(v; au; co MHN-ICN 10640; ph ML98752671)

#### Eupsittula pertinax

(Linnaeus, 1758)

75FC8886-8069-5EE0-9F7B-326C838D4EA4

##### Distribution

Espinal, Ibagué, Mariquita

##### Notes

(v; au; co; AMHN 111439; ph ML123937751). This wandering species has been observed in some areas of Tolima in some seasons, specifically in the Municipalities of Ibagué, Mariquita, Venadillo and Piedras and mainly below 1300 m a.s.l. For the first time in Tolima, we registered two individuals on 16 February 2007 (Bird Observers Group of Tolima, GOAT).

#### Ara ararauna

(Linnaeus, 1758)

55E9AA11-EE41-53AA-8D3B-17207CDC023E

##### Distribution

Mariquita, Honda

##### Notes

Visual record of E. Herrera. New record - this species was sporadically observed north of Tolima in Honda and Mariquita in June 2016. On 18 March 2018, E. Herrera registered two individuals in Honda. In the Magdalena River area, some individuals have been permanently recorded flying over Ricaurte (Cundinamarca) within the limits of Tolima. A. Armando registered an individual on 5 May 2017 in the same location.

#### Ara severus

(Linnaeus, 1758)

6E0D64DB-B156-5496-A49C-994B18C0496A

##### Distribution

Alvarado, Ibagué, Honda, Mariquita, Melgar

##### Notes

(v; ph)

#### Ara militaris

(Linnaeus, 1766)

AD4EC516-3009-592C-A077-510A60B6CC4E

##### Distribution

Prado, Melgar, Villahermosa

##### Notes

(v; ph ML83421601). New record - there are a few records of this species in Tolima. N. Bayly observed 25 individuals in the eastern area of the Department in Prado on 30 December 2010 (pers. commun., N. Bayly). Two individuals were observed by C. Guevara on 19 February 2018 in Carmen de Apicala and another individual was recorded by M. Quimbayo on 5 May 2018 in Villarrica (Fig. 8b).

#### Ara macao

(Linnaeus, 1758)

40970998-04C5-5943-A5BE-0430F93753ED

##### Distribution

Melgar

##### Notes

Close to Girardot, Ricaurte and Melgar (5 minutes from Tolima), a record in Melgar (5 individuals).

#### Leptosittaca branickii

Berlepsch & Stolzmann, 1894

AC48E11E-9B3B-5180-B48D-09AAB98E8073

##### Distribution

Anzoátegui, Cajamarca, Ibagué, Santa Isabel

##### Notes

(v; au; co ICN 33030; ph ML22278521) VU. This species was observed in high areas of Tolima on 19 November 1989 by P. Kaestner, who registered it in Toche, occurring in groups of more than 50 individuals. We have been recording groups between 20 and 30 individuals since 6 March 2006 in the Combeima River Canyon (Ibagué) above 2600 m a.s.l. in the locality of El Silencio. Individuals have also been recorded in Roncesvalles, Murillo and Santa Isabel.

#### Ognorhynchus icterotis

(Massena & Souance, 1854)

D2058C33-6EB7-5249-899B-EA736B24AF55

##### Distribution

Cajamarca, Ibagué, Murillo, Roncesvalles, San Antonio

##### Notes

(au XC420539; co CZUTOR 1073; ph ML74640611) CE CR. This species is critically endangered and even considered extinct in some areas. Its numbers have expanded, being registered in Páramo Anaime (Cajamarca) on 23 September 2006, reported in numbers greater than 80 individuals in September 2018 in Murillo (pers. commun., Y. Rincón) and registered in Roncesvalles and Toche (Ibagué) (Fig. 8c).

#### Psittacara wagleri

(Gray, 1845)

E3E32503-B07A-5C80-BE80-78917BE1691C

##### Distribution

Armero-Guayabal, Chaparral, Cunday, Espinal, Falan, Honda, Ibagué, Natagaima, Mariquita, Prado, Purificación, Rio Blanco, San Antonio, San Luis, Villarrica

##### Notes

(v; au XC386650; co MLS 1059; ph ML83420811)

#### Euchrepomis callinota

(Sclater, 1855)

CE397D06-0B3F-5E28-8A16-40BC004CDBFD

##### Distribution

Villahermosa

##### Notes

(v; au)

#### Taraba major

(Vieillot, 1816)

ECB32745-E91D-555A-AD18-F3F644EE9F2B

##### Distribution

Armero-Guayabal, Falan, Santa Isabel, Libano, Mariquita, Honda

##### Notes

(v; co MLS 3573)

#### Thamnophilus doliatus

(Linnaeus, 1764)

9B0EC5F8-62FC-5F1C-8E37-AE21BB4C6D4B

##### Distribution

Armero-Guayabal, Casabianca, Chaparral, Carmen de Apicala, Cunday, Coyaima, Dolores, Espinal, Falan, Honda, Ibagué, Lerida, Mariquita, Melgar, Libano, Ortega, Planadas, Prado, Purificación, San Luis, Rio Blanco, Venadillo, Villarrica

##### Notes

(v; au ML81693; co MLS 3634, CZUTOR 867; ph ML123186171)WD

#### Thamnophilus multistriatus

Lafresnaye, 1844

8985721E-4578-56BE-A493-F786292D53E8

##### Distribution

Anzoátegui, Casabianca, Falan, Fresno, Ibagué, Lerida, Planadas, Rio Blanco, San Antonio, Villarrica

##### Notes

(v; au XC421089, ML120515771; co AMHN 94914, CZUTOR 552) CE. The antbird, which is relatively common in the Department, usually displaces *T. doliatus* at altitudes above 1100 or 1200 m a.s.l. It is typically located between 1300 and 2100 m a.s.l. (Fig. 9e).

#### Thamnophilus atrinucha

Salvin & Godman, 1892

A5F385F0-275F-55BC-8431-A797D1DC41B6

##### Distribution

Ambalema, Chaparral, Coyaima, Espinal, Falan, Fresno, Guamo, Honda, Ibagué, Lerida, Mariquita, Prado, San Luis, Venadillo

##### Notes

(v; au XC424615, CZUT-OR1414; co MLS 3684; ph ML24068471)

#### Thamnophilus nigriceps

Sclater, 1869

DF4BC046-255C-5B32-A320-88B56D40E7F7

##### Distribution

Espinal, Falan, Fresno, Honda, Mariquita, San Luis

##### Notes

(v; co AMHN 94923)

#### Thamnophilus unicolor

(Sclater, 1859)

0A5397B8-F0D6-5DD5-AC96-361ECCBEF706

##### Distribution

Cunday, Ibagué, Libano

##### Notes

(au ML66936501; co CZUTOR 0561; ph ML44331801)

#### Dysithamnus mentalis

(Temminck, 1823)

1384E48A-36C2-5817-8903-635945C8B3D2

##### Distribution

Anzoátegui, Ibagué, Honda, Libano, Piedras, Rio Blanco, San Antonio, Villahermosa

##### Notes

(au-XC421232; ca; co CZUTOR 1443)

#### Epinecrophylla fulviventris

(Lawrence, 1862)

2BC89401-2DE9-5441-A950-A57EC388BD5D

##### Distribution

Ibagué

##### Notes

(v; au, co ML 141938061)

#### Myrmotherula pacifica

Hellmayr, 1911

ACCB9725-9506-57FD-88E5-444AFE698296

##### Distribution

San Luis, Mariquita

##### Notes

(v) Some records in eBird on the border with Caldas in the Guarino River Basin.

#### Myrmotherula axillaris

(Vieillot, 1817)

F44094D8-48D6-5BCF-9255-0C0351350C77

##### Distribution

Armero-Guayabal, Mariquita, Prado

##### Notes

(v)

#### Myrmotherula schisticolor

(Lawrence, 1865)

9EA7D4AE-3966-582C-BB31-FE95943E15DD

##### Distribution

Falan, Villahermosa, Ibagué

##### Notes

(v; ca)

#### Formicivora grisea

(Boddaert, 1783)

97E0B841-AE1E-565D-9087-56F2391CED07

##### Distribution

Armero-Guayabal, Alvarado, Ambalema, Cunday, Espinal, Honda, Ibagué, Icononzo, Falan, Lerida, Mariquita, Melgar, Planadas, Prado, Purificación, Rio Blanco, San Luis, Venadillo, Villarrica

##### Notes

(v; au-XC386651; co MLS 3776, CZUTOR 278; ph ML123886091)

#### Drymophila striaticeps

Chapman, 1912

17C62D19-B7AD-56A8-B8FD-34010F25D98A

##### Distribution

Cajamarca, Ibagué, Libano

##### Notes

(v; au ML96502701; co; ph)

#### Cercomacroides parkeri

Graves, 1997

93E415C3-3894-5C5B-947E-9869BDE80359

##### Distribution

Falan, Fresno, Libano

##### Notes

(v; au XC198035)

#### Cercomacroides tyrannina

(Sclater, 1855)

D2D406FC-54AF-5968-9F4D-81013CDACE0D

##### Distribution

Chaparral, Cunday, Falan, Honda, Ibagué, Lerida, Prado, Rio Blanco, Venadillo

##### Notes

(v; au-XC157409; co CZUTOR 0420)

#### Cercomacra nigricans

Sclater, 1858

EE5A8817-85EE-5AA5-B77B-4D93F4262EE6

##### Distribution

Armero-Guayabal, Carmen de Apicala, Coyaima, Espinal, Melgar, San Luis, Mariquita

##### Notes

(v; au; co MHN-ICN 12503, WFVZ SS-25947; ph)

#### Pyriglena maura

(Ménétriés, 1835)

35AFBEA4-FC22-54F3-AB89-C41CB75962DF

##### Distribution

Planadas

##### Notes

(au)

#### Myrmeciza longipes

(Swainson, 1825)

13A093C3-67BD-5C68-B12B-5212D466BE0F

##### Distribution

Armero-Guayabal, Ambalema, Carmen de Apicala, Coyaima, Cunday, Dolores, Espinal, Falan, Honda, Ibagué, Libano, Mariquita, Melgar, Prado, Purificación, Rio Blanco, San Luis, Venadillo, Villarrica

##### Notes

(v; au XC424466; ca; CZUTOR 0108; ph ML65475771)

#### Poliocrania exsul

(Sclater, 1859)

6825946F-AA6F-5903-9879-96024626661B

##### Distribution

Armero-Guayabal, Falan

##### Notes

(v; au)

#### Sipia palliata

(Todd, 1917)

FE58B2F3-A3E4-5BE1-A747-5989159E23F7

##### Distribution

Falan, Fresno, Ibagué, Libano

##### Notes

(v; au; ph ML26341731, ML 151083641). New record - individual observed by H. Arias in Ibagué. Like many species in its group, it is difficult to see and identify. There are some records from Tolima, Falan, Líbano and Ibagué at about 1200 to 1400 m a.s.l. On 24 March 2017, F. Espinosa photographed this species in Líbano. In Falan, it was observed by A. Pinto on 24 July 2016. In Ibagué, the first record was made by H. Arias and W. Figueroa in the Village of Chembe at 1350 m a.s.l. (pers. commun., W. Figueroa).

#### Hafferia immaculata

(Lafresnaye, 1845)

464491A0-B30C-5AA0-BEEC-AD5BC83CFC5A

##### Distribution

Ibagué, Fresno, Honda, Santa Isabel

##### Notes

(v; au; ca; co CUMV 7147; ph ML25604801)

#### Grallaria squamigera

Prevost & Des Murs, 1842

04E6336E-084F-5D83-BD95-4CAACD26E475

##### Distribution

Anzoátegui, Cajamarca, Ibagué, Roncesvalles, Santa Isabel

##### Notes

(v; co CZUTOR 1310; au ML97306261)

#### Grallaria alleni

Chapman, 1912

39A45AF6-35AB-51F4-8AAE-8F5EAF99F240

##### Distribution

Ibagué

##### Notes

au ML 176339131

#### Grallaria guatimalensis

(Prevost & Des Murs, 1842)

C7895C54-F6D9-5B4B-9B64-7EEF0FF08EE2

##### Distribution

Planadas, San Antonio

##### Notes

(au ML 513615651, ML 513615661; ph ML 261346921)

#### Grallaria ruficapilla

Lafresnaye, 1842

DA48E250-5ED5-5F5A-A441-56C5905148D1

##### Distribution

Anzoátegui, Cajamarca, Falan, Ibagué, Libano, Murillo, Planadas, San Antonio, Santa Isabel

##### Notes

(v; au XC424625, co; ph ML78998811)

#### Grallaria nuchalis

Sclater, 1860

C4D7F7A1-D6C2-55EB-9456-FE2F1A207371

##### Distribution

Anzoátegui, Cajamarca, Ibagué, Murillo, Roncesvalles

##### Notes

(v; au XC420919, ML97303881; co SIB)

#### Grallaria rufocinerea

Sclater & Salvin, 1879

CFCABF6B-8DC3-5A9E-80A3-48A8ABE2F88E

##### Distribution

Ibagué, Roncesvalles

##### Notes

(au) CE VU

#### Grallaria hypoleuca

Sclater, 1855

BD77E684-7767-5617-8C55-8F54C3084C05

##### Distribution

Casabianca, Villahermosa

##### Notes

(au ML 246568861) New record - this species was registered only in one place (Casabianca) in the Department on 26 December 2016. Its presence was strengthened by auditory identification on 5 May 2018 by F. del Castillo.

#### Grallaria saturata

Domaniewski & Stolzmann, 1918

78F9C096-C38A-5EBD-8A6F-B24D802F1343

##### Distribution

Cajamarca, Ibagué, Santa Isabel

##### Notes

(v; au-ML116201201; co SIB; ph ML119865521)

#### Grallaria quitensis

Lesson, 1844

9E0D6CE6-279C-597E-A1E7-AFF5F4D9E8AC

##### Distribution

Anzoátegui, Cajamarca, Ibagué, Murillo, Santa Isabel

##### Notes

(v; au XC96374; co SIB; ph ML45289051)

#### Grallaria milleri

(Chapman, 1912)

25CA7BC0-DEAB-5696-B9F1-99B1683F9F7B

##### Distribution

Cajamarca, Herveo, Ibagué

##### Notes

(v; au XC424621; ph ML78998821) E EN

#### Grallaricula flavirostris

(Chapman, 1924)

40CD96B8-B5A7-5456-A8E3-A9E2C17013C3

##### Distribution

Villahermosa

##### Notes

(au ML 158190801; co; ph)

#### Grallaricula lineifrons

(Chapman, 1924)

087A5DE0-6373-58E6-A0F4-5DB6D6575656

##### Distribution

Cajamarca, Ibagué, Roncesvalles

##### Notes

(ca; co CZUTOR 1255; ph ML28900731) New report - the first photographic record of the species was made by J. Sanabria on 8 August 2013 at Laguna Las Mellizas, Roncesvalles (Moreno and Losada 2016) at over 3100 m a.s.l. More recently, it has been registered in Toche (Ibagué) and photographed by J. Sanabria and H. Arias. The species is usually heard in the area that divides Tolima and Quindío in areas with bamboo shrubs and secondary forest in low strata.

#### Grallaricula cucullata

(Sclater, 1856)

E6378ACD-62A4-5C1D-B831-122562F72FCC

##### Distribution

Libano

##### Notes

(v; au; co CZUTOR 0560; ph)

#### Grallaricula nana

(Lafrenaye, 1842)

BE3BBD30-9F3E-51EE-98CE-0A4CCFFDEE92

##### Distribution

Anzoátegui, Falan, Ibagué, Murillo

##### Notes

(v; au; co SIB; ph ML78998841, ML97311561)

#### Acropternis orthonix

(Lafresnaye, 1843)

B0F5BDAB-3ECF-5A9E-B075-05C269CB131D

##### Distribution

Anzoátegui, Cajamarca, Ibagué, Roncesvalles

##### Notes

(v; au XC96366; co SIB 126283)

#### Myornis senilis

(Lafresnaye, 1940)

0C244C29-241B-5F8E-A3B4-DFC7470BB441

##### Distribution

Anzoátegui, Cajamarca, Fresno, Ibagué, Murillo, Planadas

##### Notes

(v; au XC96508, ML 96567561; co SIB 126726)

#### Scytalopus opacus

Zimmer, 1941

6A68DBDC-C3C4-5C62-BDB3-39E8EC0577D8

##### Distribution

Anzoátegui, Cajamarca, Ibagué, Murillo, Santa Isabel

##### Notes

(v; ph ML115352241)

#### Scytalopus atratus

Hellmayr, 1922

8971F4CA-EE18-590F-A7B1-4379A69682D5

##### Distribution

Ibagué, Roncesvalles, Cajamarca, Fresno, Libano, Murillo, Herveo, Santa Isabel

##### Notes

(v; au XC424490; co)

#### Scytalopus latrans

Hellmayr, 1924

5CDA4AB7-B184-54D0-819A-BB35DF518635

##### Distribution

Falan, Ibagué, Cajamarca, Roncesvalles, Anzoategui, Libano, Murillo, Casabianca, Herveo

##### Notes

(au XC424624; co SIB 126324)

#### Scytalopus rodriguezi

Krabbe et al., 2005

28F0EC4D-6AC7-545C-A3C3-147BD602CBA2

##### Distribution

Planadas

##### Notes

(au ML 156858411)

#### Scytalopus spillmani

Stresemann, 1937

A7271442-1F86-5B0A-81C8-871164100FD2

##### Distribution

Anzoátegui, Cajamarca, Ibagué

##### Notes

(au XC424619; co SIB 126327)

#### Chamaeza turdina

Cabanis & Heine, 1859

89D2F53F-4BB9-5FBE-9B7E-62D39C6CB02C

##### Distribution

Dolores, Villarrica

##### Notes

(v)

#### Chamaeza mollissima

Sclater, 1855

E963358F-7CBA-5424-A2AE-622A1E4E73A9

##### Distribution

Ibagué, Roncesvalles

##### Notes

(au; ca)

#### Sclerurus obscurior

(Hartert, 1901)

211B4AE4-ED12-56A3-AC0A-E929954E43FF

##### Distribution

Villahermosa

##### Notes

(v; ph ML 158144061)

#### Sittasomus griseicapillus

(Vieillot, 1818)

D2BDACDA-707E-550F-A7B0-1147147DC703

##### Distribution

Falan, Libano

##### Notes

(v)

#### Dendrocincla tyrannina

(Lafrenaye, 1851)

7A28B658-64DA-57E9-AC40-602858E13C30

##### Distribution

Anzoátegui, Cajamarca, Ibagué, Libano, Murillo, San Antonio, Villarrica

##### Notes

(v; au; co CZUTOR 0527; ph)

#### Dendrocincla fuliginosa

(Vieillot, 1818)

81935DDB-FDC4-5304-B68C-77BD3AF9D891

##### Distribution

Falan, Fresno, Honda, Ibagué, Icononzo, Lerida, Libano, Mariquita, Prado, San Antonio

##### Notes

(v; ca; co MPUJ 764, CZUTOR 0263; ph ML26003081)

#### Glyphorynchus spirurus

(Vieillot, 1819)

D1A35DE7-5626-59B5-8F89-604F891EA994

##### Distribution

Villahermosa

##### Notes

(v; au)

#### Dendrocolaptes picumnus

Linchtenstein, 1820

66048C17-56B4-5D27-A6B3-22BAC956CCF5

##### Distribution

Anzoátegui, Falan, Ibagué, Libano, Murillo

##### Notes

(v; co CZUTOR 0521; ph)

#### Xiphocolaptes promeropirhynchus

(Lesson, 1840)

0193FBF1-459D-5FDC-9A0A-F32EC47B87F0

##### Distribution

Anzoátegui, Ibagué, Libano, Murillo, Planadas, Villarrica

##### Notes

(v; ca; co CZUTOR 0584; ph ML104274931)

#### Xiphorhynchus susurrans

(Jardine, 1847)

15C778F9-308A-5239-A5EE-C07E1D3FE3F1

##### Distribution

Espinal, Falan, Ibagué, Honda, Lerida, Purificación

##### Notes

(au XC424400, ML82108, CZUT OR0386; co MLS 341)

#### Xiphorhynchus triangularis

(Lafresnaye, 1842)

785477B5-BD1A-569A-9CEF-ED3CD9D452DA

##### Distribution

Cajamarca, Cunday, Ibagué

##### Notes

(v; co MLS 3353)

#### Dendroplex picus

(Gmelin, 1788)

CB568389-18F9-563B-895E-5D63B110A311

##### Distribution

Armero-Guayabal, Ambalema, Carmen de Apicala, Cunday, Espinal, Honda, Ibagué, Falan, Mariquita, Melgar, Prado, Purificación, Saldaña, Venadillo, Villarrica

##### Notes

(v; au-XC420237; co MLS 3289, CZUTOR 664; ph ML104428621)

#### Campylorhamphus trochilirostris

(Lichtenstein, 1820)

7441995A-4ABD-569C-850F-63C3F67F8596

##### Distribution

Armero-Guayabal, Cunday, Espinal, Falan, Guamo, Honda, Ibagué, Icononzo, Lerida, Mariquita, Natagaima, Rio Blanco

##### Notes

(v; au XC424480; co MLS 3375, CZUTOR 0778; ph ML123826951)

#### Campylorhamphus pusillus

(Sclater, 1860)

B4470E85-3757-5DAF-80D3-E2FE48E71FDE

##### Distribution

Falan, Ibagué

##### Notes

(ca, au XC16569; ph)

#### Lepidocolaptes souleyetii

(DesMurs, 1849)

E3870B0B-395D-5740-BE3F-5DA62D756E0D

##### Distribution

Chaparral, Falan, Honda, Ibagué, San Antonio, San Luis

##### Notes

(v; ca; au-XC420378; co MLS 3369, CZUTOR; ph ML105911441)

#### Lepidocolaptes lacrymiger

(Des Murs, 1849)

6FA5DC92-8A1C-574D-90C9-27EE790A8BF1

##### Distribution

Anzoátegui, Cunday, Honda, Ibagué, LIbagué, Murillo, Planadas, Prado, San Antonio, Santa Isabel, Villarrica

##### Notes

(v; co CZUTOR 0654; ph ML123925381)

#### Xenops mexicanus

(Sclater, 1857)

F58365E5-DF12-519C-9A4D-53EC5755673D

##### Distribution

Casabianca, Chaparral, Falan, Ibagué, Icononzo, San Antonio

##### Notes

(v; ca; co CUMV 6984; ph ML102226081)

#### Xenops rutilans

(Teminck 1821)

AA60C118-F0DC-5B3E-9BCD-07C6FDB21AF4

##### Distribution

Anzoátegui, Falan, Ibagué, Libano, Murillo, San Antonio, Santa Isabel

##### Notes

(v; ca; co CZUTOR 0149; ph ML99043111)

#### Pseudocolaptes boissonneautii

(Lafresnaye, 1840)

3098B79F-DF39-5F3A-BC7D-7C9BE3DF98E8

##### Distribution

Anzoátegui, Cajamarca, Falan, Ibagué, Murillo, Santa Isabel, Villarrica

##### Notes

(v; ca; co ISB 126315; ph ML22276301)

#### Premnornis guttuliger

(Sclater, 1864)

846C55F3-AC69-5CB0-AD2A-892386929505

##### Distribution

Cunday, Ibagué, Libano, Murillo

##### Notes

(v; co CZUTOR 0582)

#### Lochmias nematura

(Lichtenstein, 1823)

391CE97F-EECB-5965-B90D-4D06BB4BF7CA

##### Distribution

Cajamarca, Ibagué, Planadas, Rio Blanco

##### Notes

(v; co MHN-ICN 26880, CZUTOR 516; ph)

#### Cinclodes excelsior

Sclater, 1860

0BC32DA1-C4DF-5EBC-A9DD-E3EC23ABBB0E

##### Distribution

Anzoátegui, Cajamarca, Herveo, Ibagué, Murillo, Santa Isabel

##### Notes

(v; co; ph ML58185941)

#### Cinclodes albidiventris

Sclater, 1860

B0861B2F-4E26-5DB0-A4E1-5162C7FCA880

##### Distribution

Herveo, Ibagué

##### Notes

(v)

#### Anabacerthia striaticollis

Lafresnaye, 1841

F21484DC-C175-5EE4-97E5-79E2950A61DF

##### Distribution

Anzoátegui, Casabianca, Cunday, Falan, Ibagué, Libano, Planadas, San Antonio, Santa Isabel, Villahermosa, Villarrica

##### Notes

(v; ca; co CZUTOR 0054; ph ML99052401)

#### Syndactyla subalaris

(Sclater, 1859)

2C6E89C6-970F-54FC-95DA-859530C56576

##### Distribution

Falan, Ibagué, Roncesvalles

##### Notes

(v; co CZUT OR0994)

#### Dendroma rufa

(Vieillot, 1818)

E2ED5F07-380A-5AD0-8E9D-6D51AF3CB7C4

##### Distribution

Villahermosa

##### Notes

(v; au)

#### Clibanornis rubiginosus

(Sclater, 1857)

C6B1B323-E661-51FB-9492-23B79B79BA15

##### Distribution

Ibagué, Libano

##### Notes

(v; au XC376596, ML61689491). New report - this bird is difficult to observe, but recognisable by its song. It was observed in a tangled area over 900 m a.s.l. in the Convention (Líbano) on 11 June 2017 (record from eBird). In the Municipality of Ibagué, it was registered for the first time by O. Humberto on 15 November 2008 in the north-western hills. We recorded the song of an individual in this municipality in the Gaia Nature Reserve in April 2017 (XC404392).

#### Thripadectes flammulatus

(Eyto, 1849)

A76AE48A-6F66-5DE3-9B9F-491552D8720C

##### Distribution

Ibagué, Rio Blanco

##### Notes

(v; au ML78998871)

#### Thripadectes holostictus

(Sclater & Salvin, 1876)

036F81F8-1518-5608-9F98-E8F8F6DCC07F

##### Distribution

Ibagué

##### Notes

(v; au; co CZUTOR 084)

#### Thripadectes virgaticeps

Lawrence, 1874

DC64ECF5-A5E9-5FDC-B67D-18ED5488952D

##### Distribution

Falan, Ibagué, Libano

##### Notes

(v; ca; ph ML76072981)

#### Automolus ochrolaemus

(Tschudi, 1844)

137A55B5-B7EB-5684-9DFC-3A612B9DCE10

##### Distribution

Falan, Honda, Ibagué, Libano, Icononzo, Rio Blanco, Venadillo

##### Notes

(v; au XC24100; ca co CZUT 0590; ph ML109878071)

#### Premnoplex brunnescens

(Sclater, 1856)

1D89F26C-8FB2-5E0A-ABF0-8896763A08FA

##### Distribution

Chaparral, Falan, Ibagué, Planadas, Santa Isabel

##### Notes

(v; ca; co CZUTOR 437; ph ML234221281)

#### Margarornis squamiger

(d'Orbigny & Lafrenayi, 1838)

E6608112-4933-5685-B1E1-6EA244352ACA

##### Distribution

Anzoátegui, Natagaima, Cajamarca, Cunday, Falan, Ibagué, Icononzo, Murillo, Prado, Santa Isabel, Villarrica

##### Notes

(v; ca; co AMHN 132229, CZUTOR 506; ph ML51937281)

#### Leptasthenura andicola

Sclater, 1870

1550563D-13ED-53E7-A4AC-DD77FF997AF9

##### Distribution

Cajamarca, Ibagué, Murillo, Santa Isabel

##### Notes

(v; co; ph ML32219251)

#### Hellmayrea gularis

(Lafresnaye, 1843)

7A681B3C-C000-5274-8D56-B13DAE7E1332

##### Distribution

Anzoátegui, Cajamarca, Ibagué, Roncesvalles, Santa Isabel

##### Notes

(v; co SIB 126813, MHN-ICN 26223)

#### Asthenes flammulata

(Jardine, 1850)

9311D222-6755-50F7-9985-3810502288F0

##### Distribution

Cajamarca, Ibagué, Murillo, Santa Isabel

##### Notes

(v; co SIB 6743; ph ML115351491)

#### Asthenes fuliginosa

(Lafresnaye, 1843)

B51BB533-A8A6-54A6-BD4C-7583959FC3FC

##### Distribution

Anzoátegui, Ibagué, Murillo, Villahermosa

##### Notes

(co MHN-ICN 5916; ca; ph ML107110871)

#### Siptornis striaticollis

(Lafresnaye, 1843)

72D1819E-F86C-5F44-9C44-B8785D785FC5

##### Distribution

Ibagué, San Antonio

##### Notes

(v; ph ML 174982751)

#### Cranioleuca curtata

(Sclater, 1870)

F1E1477A-5B08-5E05-B136-C9B4A636C97A

##### Distribution

Villahermosa, Icononzo

##### Notes

(v; au ML 156435591, ph ML 221890931)

#### Certhiaxis cinnamomeus

(Gmelin, 1788)

BEB20147-3D71-5468-A8F0-3C8CF15D5567

##### Distribution

Armero-Guayabal, Falan, Guamo, Ibagué, Prado

##### Notes

(v; au; co CZUTOR 0816; ph ML123879031)

#### Synallaxis brachyura

Lafresnaye, 1843

A3C8B82B-50B4-5D0C-A763-87D443047E89

##### Distribution

Armero-Guayabal, Ataco, Casabianca, Cunday, Falan, Honda, Ibagué, Icononzo, Libano, Mariquita, Planadas, Rio Blanco, San Antonio, Venadillo, Villarrica

##### Notes

(v; au XC420556; co MLS 3469, CZUTOR 735; ph ML72671011)

#### Synallaxis albescens

Temminck, 1823

B991120F-37ED-5AA0-84F7-C528A483CACE

##### Distribution

Armero-Guayabal, Ambalema, Casabianca, Chaparral, Cunday, Dolores, Falan, Ibagué, Icononzo, Lerida, Planadas, Prado, Rio Blanco, San Luis, Villarrica

##### Notes

(v; au XC376475; ca; co CZUTOR 0348; ph ML26003001)

#### Synallaxis azarae

d'Orbigny, 1835

29F9D658-562E-57E6-AEA6-4A4F9CF3BC50

##### Distribution

Anzoátegui, Casabianca, Cajamarca, Cunday, Ibagué, Falan, Honda, Libano, Murillo, Planadas, Prado, San Antonio Santa Isabel, Villarricallahermosa, Villarrica

##### Notes

(v; au XC423758; ca; co WFVZ 20731; ph ML115221491)

#### Synallaxis unirufa

Lafresnaye, 1843

2B17AD54-E20D-53EF-9F3D-74B460EC82FA

##### Distribution

Anzoátegui, Cajamarca, Ibagué

##### Notes

(v; au ML97307601; ca; co SIB 126382)

#### Synallaxis cinnamomea

Lafresnaye, 1843

204534B7-681A-5977-B48A-8FB68241410E

##### Distribution

Icononzo, Prado

##### Notes

(co)

#### Chloropipo flavicapilla

(Sclater, 1852)

B48BEFC2-36B4-52B8-8750-F08E62C537B6

##### Distribution

Chaparral, Cunday, Falan, Ibagué, Icononzo, Libano, Planadas, Santa Isabel

##### Notes

(v; ca; co CZUTOR 0568; ph) E NT

#### Chiroxiphia lanceolata

(Wagler, 1830)

D4448ED3-0596-5D4E-958E-D400A44488CB

##### Distribution

Armero-Guayabal, Alvarado, Ambalema, Ibagué, Espinal, San Luis, Venadillo

##### Notes

(v; au; ca; co CZUTOR 0825; ph)

#### Masius chrysopterus

(Lafresnaye, 1843)

9C868FD1-38A9-558D-8FB4-99756D5E5075

##### Distribution

Cunday, Dolores, Ibagué, Falan, Melgar, San Antonio, Villarrica

##### Notes

(v; ca; co CZUTOR 416, MLS 4230)

#### Corapipo leucorrhoa

(Sclater, 1863)

0D337069-55C5-5343-AD10-8C25B0C6B1D7

##### Distribution

Falan, Honda, Libano, Mariquita, Rio Blanco

##### Notes

(v; ca; co CZUTOR 930, MPUJ 758)

#### Lepidothrix coronata

(Spix, 1825)

B8294104-0A44-5ABD-A8B4-EE9A1426CCE6

##### Distribution

Falan

##### Notes

(v)

#### Manacus manacus

(Linnaeus, 1766)

C059F469-3F91-53C3-A247-B4EDDC7BB133

##### Distribution

Armero-Guayabal, Ambalema, Carmen de Apicala, Cunday, Dolores, Espinal, Honda, Ibagué, Icononzo, Guamo, Falan, Lerida, Mariquita, San Luis, Ortega, Planadas, Prado, Purificación, Rio Blanco, San Antonio, Venadillo, Villarrica

##### Notes

(v; au XC386465; ca; co MLS 425, CZUTOR 30; ph ML99188661)

#### Macheropterus striolatus

(von Pelzeln, 1856)

70DCD4B2-802B-5026-A8DB-A149A286C5FB

##### Distribution

Espinal, Falan, Honda, Libano

##### Notes

(v; co AMHN 94973; ph L105060731)

#### Pseudopipra pipra

(Linnaeus, 1758)

47D717A5-7A6D-54F3-82B9-C864795DE7F3

##### Distribution

Falan, Libano, Rio Blanco

##### Notes

(au ML970632411; co CZUT557; ph ML123832711). We initially observed and collected (CZUT–Or 557) an individual in Quebradon (Rio Blanco) in May 2018 (Molina-Martínez et al. 2015). Additionally, this species was registered in Líbano and Falan in 2017 by J. Sanabria and recently registered in Planadas at a height of about 1300 m a.s.l. in well-conserved forest vocalising in middle strata (Fig. 10c).

#### Ceratopipra erythrocephala

(Linnaeus, 1758)

530E7976-AE04-50BD-B920-40E1786D98B2

##### Distribution

Honda, Ibagué, Falan, Mariquita

##### Notes

(v; ca; co AMHN 94968; au ML81684; ph ML123832151) This manakin was registered in humid areas of the north of the Department: in Falan in July 2006 and in Mariquita at low and medium strata.

#### Pipreola riefferii

(Boissoneau, 1840)

342D2372-99CD-5CC5-AEA9-04C05FB1C247

##### Distribution

Anzoátegui, Cajamarca, Falan, Ibagué, Honda, Murillo, San Antonio, Santa Isabel

##### Notes

(v; ca; co CZUTOR 0083; ph ML99050311)

#### Pipreola arcuata

(Lafresnaye, 1843)

01C906B5-E529-537A-AF28-3164CF397DFE

##### Distribution

Anzoátegui, Ibagué

##### Notes

(v; co SIB A6778; ph ML115212811)

#### Pipreola aureopectus

(Lafresnay, 1843)

54A8B6CB-1CC3-567A-B116-F8280D2DDB1C

##### Distribution

Planadas

##### Notes

(au ML97063491; ph ML97066671). New record - this species was registered in Tolima on 22 April 2018 in the Municipality of Planadas by H. Arias, R. Parra, K. Certuche and M. Olaya in mature forest (Fig. 10b). This record corresponds to the first record made in the Upper Magdalena Valley.

#### Doliornis remseni

Robbins, Rosenberg & Molina, 1994

9880104E-D7F0-52D5-9735-A3E6D5D644DA

##### Distribution

Rio Blanco, Planadas, Roncesvalles, Cajamarca

##### Notes

(v) In areas of páramo vegetation in Tolima

#### Ampelion rubrocristatus

(d'Orbigny & Lafresnaye, 1837)

A629288B-94A9-5C27-8D77-A8A5C307C23A

##### Distribution

Anzoátegui, Cajamarca, Ibagué, Lerida, Libano, Murillo, Santa Isabel

##### Notes

(v; co MHN-ICN 27018, CZUTOR 0069; ph ML114073411)

#### Ampelion rufaxilla

(Tschudi, 1844)

BA689ADF-B370-5946-ACB4-0BA176B6E421

##### Distribution

Murillo, Rio Blanco, Roncesvalles

##### Notes

(v) Several photographic records on the border with the Department of Huila in the PNN nevado del Huila.

#### Rupicola peruviana

(Latham, 1790)

91937428-51A0-561D-8B6A-9262D2F8F357

##### Distribution

Ataco, Coyaima, Espinal, Ibagué, Falan, Planadas, Rio Blanco, San Antonio, Villarrica

##### Notes

(v; au; co CZUTOR 1444; ph ML98753151)

#### Pyroderus scutatus

(Shaw, 1792)

0888EBCC-58AA-5061-92AE-F01301DDBFC8

##### Distribution

Roncesvalles, Villarrica

##### Notes

(MNHNP CG1928.1067) In addition to a few records in Tolima, an individual was deposited in the Museum National d'Histoire Naturelle Paris (CG1928.1067). Two observations were made in a primary forest in Galilea, Villahermosa: the first observation was on 25 Jan 2019 with a photographic record (pers. commun., L. Arevalo) and another record was made on 4 May 2019 in primary forest (Fig. 13a).

#### Lipaugus fuscocinereus

(Lafresnaye, 1843)

0377EF83-B308-5A72-BB85-374D684D1C8C

##### Distribution

Cajamarca, Ibagué, Villarrica

##### Notes

(v; au; co ANSP 154658; ph ML22278721)

#### Tityra inquisitor

(Lichtenstein, 1823)

3AF89651-413F-5B21-9E8B-3A6649A88FF4

##### Distribution

Armero-Guayabal, Coyaima, Espinal, Guamo, Falan, Ibagué, Honda, Libano, Mariquita, Ortega

##### Notes

(v; co MHN-ICN 9065, CZUT OR1500; ph ML99193431)

#### Tityra semifasciata

(Spix, 1825)

8D211309-F244-5BDB-B478-435A4921A6DB

##### Distribution

Armero-Guayabal, Coyaima, Espinal, Falan, Planadas, San Luis, Roncesvalles, Rovira

##### Notes

(v; co MLS 4112)

#### Pachyramphus versicolor

(Hartlaub, 1843)

800F4026-31B7-54CC-BEF1-2B49D12BE164

##### Distribution

Cajamarca, Falan, Ibagué, Planadas

##### Notes

(v; co AMHN 112414; ph ML97743731)

#### Pachyramphus rufus

(Boddaert, 1783)

0AC51EC1-5E16-591E-9977-B9D15538B6C7

##### Distribution

Coyaima, Honda, Falan, Ibagué, Gualanday, Libano, Melgar, Purificación, San Antonio, San Luis

##### Notes

(ca; co MLS 4053; ph ML123899261)

#### Pachyramphus cinnamomeus

Lawrence, 1861

6FEB2F9B-18E6-59E0-BCC1-A8F1DA33DCFB

##### Distribution

Armero-Guayabal, Falan, Honda, Ibagué, Mariquita, Venadillo

##### Notes

(v; co AMHN 94936, CZUTOR 865)

#### Pachyramphus polychopterus

(Vieillot, 1818)

1CDBFD31-9C06-597B-A8C7-54ACADA04F57

##### Distribution

Chaparral, Coyaima, Honda, Ibagué, Fresno, San Antonio

##### Notes

(v, co AMHN 71743; ph ML123914521)

#### Platyrinchus mystaceus

Vieillot, 1818

AD67FDE5-8218-5C08-B737-0A063D14938E

##### Distribution

Ibagué, Icononzo, Rio Blanco

##### Notes

(ca; co CZUT 558; ph)

#### Pseudotriccus ruficeps

(Lafresnaye, 1843)

8B9E42E5-6BC8-56C8-BAB5-F27660AC859A

##### Distribution

Cajamarca, Ibagué, Roncesvalles, Santa Isabel

##### Notes

(ca; co CZUTOR 1250)

#### Pogonotriccus ophthalmicus

(Taczanowsky, 1874)

E0527B23-4548-5D40-9433-7319E2098E83

##### Distribution

Falan, Ibagué, Herveo, Murillo, San Antonio

##### Notes

(v; ca; co AMHN 112285; ph ML123897501)

#### Pogonotriccus poecilotis

(Sclater, 1862)

7066D356-13BD-5A4C-911A-D7E6B8264C11

##### Distribution

Falan, Ibagué, Libano, Planadas, San Antonio, Villarrica

##### Notes

(v; ca; co AMHN 112242)

#### Phylloscartes superciliaris

(Sclater & Salvi, 1868)

B15241C5-4C95-5B6E-8C06-DD4BD02240FA

##### Distribution

Villahermosa

##### Notes

(v)

#### Mionectes striaticollis

(Orbingy & Lafresnaye, 1837)

3A077BD7-4999-5FE7-B848-70E138F0EBB2

##### Distribution

Anzoátegui, Herveo, Falan, Ibagué, Libano, Planadas, Prado, Roncesvalles, Santa Isabel, Villarricallahermosa, Villarrica

##### Notes

(v; ca; co CZUTOR 040; ph ML79638951)

#### Mionectes olivaceus

Lawrence, 1868

EEEBEB48-3BF6-5917-999E-ADDF64805346

##### Distribution

Armero-Guayabal, Dolores, Falan, Honda, Icononzo, Mariquita, Libano, Venadillo

##### Notes

(v; ca; co CZUTOR 0414)

#### Mionectes oleagineus

(Lichtenstein, 1823)

4F3FC3E0-7D57-54DC-8A70-93BF420A0465

##### Distribution

Armero-Guayabal, Alvarado, Chaparral, Falan, Guamo, Honda, Ibagué, Icononzo, Lerida, Prado, San Antonio, Venadillo, Villarrica

##### Notes

(v; ca; co MPUJ 855, CZUTOR 258; ph ML123920351)

#### Leptopogon amaurocephalus

Tschudi, 1846

29C55E9D-A91C-5357-9B53-A44147CD5068

##### Distribution

Armero-Guayabal, Chaparral, Cunday, Espinal, Falan, Ibagué, Mariquita, Prado, Venadillo, Villarrica

##### Notes

(v; ca; co; MLS 5003, CZUTOR 0319; ph)

#### Leptopogon superciliaris

Tschudi, 1844

5A896B5C-4A23-5264-929D-C84C633159CB

##### Distribution

Armero-Guayabal, Honda, Ibagué, Libano, Mariquita, Venadillo, Villarrica

##### Notes

(v; au; co CZUTOR 1006; ph ML123905261)

#### Leptopogon rufipectus

(Lafresnaye, 1846)

C9BBC120-4028-57C5-BE62-63F70BD7E46E

##### Distribution

Falan, Ibagué, Rio Blanco

##### Notes

(ca; co CZUTOR 0765; ph ML78849961) CE

#### Tolmomyias sulphurescens

(Spix, 1825)

4DBE1CBA-FFA0-52D0-A2D4-177C87666D2D

##### Distribution

Armero-Guayabal, Chaparral, Carmen de Apicala, Falan, Gualanday, Guamo, Honda, Ibagué, Lerida, Mariquita, Melgar, Natagaima, Saldaña, San Luis, Venadillo

##### Notes

(v; au XC335916ca; co MLS 4658 CZUTOR 417; ph)

#### Lophotriccus pileatus

(Tschudi, 1844)

ADE31761-2BD0-5641-B9B2-059880483CD4

##### Distribution

Anzoátegui, Ibagué, Herveo, Fresno, Icononzo, Libano, Murillo, San Antonio, Villarrica

##### Notes

(v; au XC421235; ca; co MLS 4732, CZUTOR 882; ph ML23232981)

#### Atalotriccus pilaris

(Cabanis, 1847)

18F588F4-0268-5892-AB66-3745B6B74BAE

##### Distribution

Alvarado, Ambalema, Armero-Guayabal, Chaparral, Coello, Cunday, Coyaima, Doima, Espinal, Falan, Honda, Ibagué, Gualanday, Lerida, Mariquita, Melgar, Natagaima, Prado, Purificación, San Antonio, San luis, Villarrica

##### Notes

(v; ca; co MLS 4734, CZUTOR 294; ph ML65473221)

#### Hemitriccus margaritaceiventer

(d'Orbigny & Lafresnaye, 1837)

F6C791D5-3A15-55BC-981C-00FE62912026

##### Distribution

Armero-Guayabal, Alvarado, Chaparral, Coello, Coyaima, Doima, Falan, Ibagué, Honda, Lerida, Mariquita, Melgar, Natagaima, Purificación, Venadillo

##### Notes

(v; co CZUT OR1359; ph ML75626401)

#### Hemitriccus granadensis

(Hartlaub, 1843)

BBE03978-F14A-5FBB-A17A-114E8AA75589

##### Distribution

Cajamarca, Herveo, Ibagué, Falan, Libano, Murillo

##### Notes

(v; co ANSP 153871)

#### Poecilotriccus ruficeps

(Kaup, 1852)

9CD6D4C1-BEFD-5B0E-83EA-BACE780899FC

##### Distribution

Anzoátegui, Cajamarca, Chaparral, Falan, Herveo, Ibagué, Murillo, Planadas, Santa Isabel, San Antonio

##### Notes

(v; co AMHN 112218; ph ML115221431)

#### Poecilotriccus sylvia

(Desmarest, 1806)

B5ACECAF-ADE7-542C-8C01-9EA6B11DF3ED

##### Distribution

Ambalema, Alvarado, Armero-Guayabal, Ambalema, Chaparral, Coyaima, Falan, Ibagué, Honda, Falan, Guamo, Lerida, Mariquita, Melgar, Purificación, San Luis, Suarez, Valle de San Juan, Venadillo, Villarrica

##### Notes

(v; co MLS 8288, CZUTOR 727; ph)

#### Todirostrum cinereum

(Linnaeus, 1766)

0615DB5A-F9F1-5360-ACBF-769CE6202724

##### Distribution

Alpujarra, Alvarado, Ambalema, Armero-Guayabal, Ataco, Casabianca, Chaparral, Coyaima, Cunday, Dloes, Espinal, Falan, Fresno, Guamo, Herveo, Honda, Ibagué, Icononzo, Lerida, Libano, Natagaima, Mariquita, Melgar, Ortega, Palocabildo, Planadas, Prado, Rio Blanco, Roncesvalles, Rovira, San Antonio, San Luis, Saldaña, Valle de Sa Juan, Venadillo, Villarrica

##### Notes

(v; au XC424491; ca; co MLS 4715, CZUTOR 350; ph ML72671281)

#### Todirostrum nigriceps

(Sclater, 1855)

0DE6D936-930F-5AD8-94E6-7F1C33CF50F7

##### Distribution

Armero-Guayabal, Purificación, Falan, Honda, Mariquita, Melgar

##### Notes

(v; ph)

#### Myiotriccus ornatus

(Lafresnaye, 1853)

D8002AA4-3755-5368-8DE0-2C34205FFBF8

##### Distribution

Anzoategui, Honda, Villarrica

##### Notes

(v; co; ph ML 156434011)

#### Nephelomyias pulcher

(Sclater, 1861)

E2BE417D-2435-548B-8FE8-ED53ED7C48C9

##### Distribution

Ibagué, Libano

##### Notes

(v, ph ML 79689091)

#### Hirundinea ferruginea

(Gmelin, 1788)

EDC53BDB-6D43-57BA-ACF9-9357E217FDC0

##### Distribution

Chaparral, Ibagué, Icononzo, Melgar, Natagaima

##### Notes

(v; co SIB A348; ph). New record - there are a few recent records of this species in Tolima. On 19 March 2015, Y. Tolosa and companions registered it in the canyon of the Combeima River (Ibagué). It was also registered in Chaparral (Vega Chiquita) by M. Moreno on 1 December 2013 and in Toche (Ibagué) by P. Kaestner and F. Espinosa in October 2018.

#### Pyrrhomyias cinnamomeus

(d'Orbigny & Lafresnaye, 1837)

F3F6E07A-B33A-5862-83CA-99F4FC2A7A0B

##### Distribution

Anzoátegui, Cajamarca, Chaparral, Falan, Herveo, Ibagué, Libano, Murillo, Planadas, Roncesvalles, San Antonio, Santa Isabel, Villahermosa, Villarrica

##### Notes

(v; au XC428187; co SIB 126321; ph ML115221451)

#### Zimmerius chrysops

(Sclater, 1859)

D74B8404-8A5A-5A9D-B907-42E4AFB57F51

##### Distribution

Anzoátegui, Casabianca, Cunday, Dolores, Falan, Fresno, Herveo, Honda, Ibagué, Icononzo, Lerida, Mariquita, Melgar, Murillo, Palocabildo, Planadas, Prado, Rio Blanco, San Antonio, Villahermosa, Villarrica

##### Notes

(v; au XC157385; ca; co MPUJ 751, 908; ph ML123928791)

#### Euscarthmus meloryphus

Wied-Neuwied, 1831

639CB8CD-6F99-5E9D-A710-AD498CB2AA6C

##### Distribution

Ambalema, Alvarado, Armero-Guayabal, Cajamarca, Chaparral, Carmen de Apicala, Coello, Doima, Ibagué, Falan, Honda, Mariquita, Natagaima, San Luis, Venadillo

##### Notes

(v; ca; co CZUTOR 0389; ph)

#### Elaenia flavogaster

(Thunberg, 1822)

E11EE5C3-9C4D-5DB1-AB75-3A0D74E3A2B7

##### Distribution

Alvarado, Anzoategui, Ambalema, Armero-Guayabal, Ataco, Cajamarca, Casabianca, Carmen de Apicala, Chaparral, Chicoral, Coello, Coyaima, Cunday, Doima, Dolores, Espinal, Guamo, Honda, Ibagué, Icononzo, Falan, Fresno, Lerida, Libano, Mariquita, Planadas, Prado, Rio Blanco, San Luis, Venadillo, Villarrica

##### Notes

(v; au XC424613; ca; co MLS 4916, CZUTOR 337; ph ML123883711) WD

#### Elaenia parvirostris

Pelzeln, 1868

C39FC273-9099-50D1-9763-09404487EB8E

##### Distribution

Cunday, Falan, Ibagué, Icononzo, Prado, Rovira, San Luis, Venadillo, Villarrica

##### Notes

(v; ph ML 123919531) M

#### Elaenia chiriquensis

Lawrence, 1865

6CA12781-D493-579C-938A-6AB39FC84D6F

##### Distribution

Alpujarra, Alvarado, Armero-Guayabal, Doima, Dolores, Honda, Ibagué, Icononzo, Falan, Fresno, Libano, Mariquita, Prado, Rovira, Valle de San Juan

##### Notes

(v; ca; co CZUTOR 0252; ph ML98988291)

#### Elaenia frantzii

Lawrence, 1865

97DDBF4E-F827-55B7-9025-0DF10EFF3EF9

##### Distribution

Anzoátegui, Cajamarca, Casabianca, Dolores, Falan, Ibagué, Icononzo, Herveo, Libano, Melgar, Murillo, Palocabildo, Planadas, Prado, Roncesvalles, San Antonio, Santa Isabel, Villahermosa, Villarrica

##### Notes

(v; au-XC424585; co CZUTOR 0039; ph ML97745681)

#### Elaenia pallantagae

Sclater, 1862

C66A94FF-692A-5167-A30B-B9619826E045

##### Distribution

Alvarado, Ibagué, Honda, Rovira, Planadas

##### Notes

(v; ca; co CZUTOR 0136; ph ML123919531)

#### Tyrannulus elatus

(Latham, 1790)

B30305B5-7EC1-525F-B8F3-0198B7546FB7

##### Distribution

Alvarado, Ambalema, Armero-Guayabal, Ambalema, Carmen de Apicala, Chaparral, Cunday, Doima, Falan, Fresno, Honda, Ibagué, Lerida, Mariquita, Melgar, Natagaima, Prado, Rio Blanco, Rovira, San Luis, Valle de San Juan, Saldaña, Venadillo

##### Notes

(v; au XC424041; co MLS 4984, MHN-ICN 8628; ph ML123883491)

#### Myiopagis gaimardii

(d'Orbigny, 1840)

93ADB85E-431B-5E07-821C-C9859B99E89D

##### Distribution

Alvarado, Armero-Guayabal, Chaparral, Coyaima, Doima, Libano, Mariquita, Melgar, Planadas, Venadillo

##### Notes

(co MLS 8004)

#### Myiopagis viridicata

(Vieillot, 1817)

CC8D5CD1-3FC4-52B8-BC2A-C2B6498F43D7

##### Distribution

Armero-Guayabal, Chaparral, Carmen de Apicala, Coyaima, Cunday, Espinal, Falan, Fresno, Honda, Ibagué, Icononzo, Honda, Libano, Mariquita, Melgar

##### Notes

(v; ca; co MLS 4964, CZUTOR 368; ph ML123886491)

#### Capsiempis flaveola

(Lichtenstein, 1823)

3225E653-3171-5F55-9461-40AF25E96369

##### Distribution

Armero-Guayabal, Alvarado, Ataco, Carmen de Apicala, Chaparral, Doima, Icononzo, Gualanday, Espinal, Falan, Herrera, Honda, Mariquita, Melgar, San Luis, Venadillo

##### Notes

(v; au XC365770; ca; co MLS 5027, CZUT OR1426)

#### Mecocerculus poecilocercus

(Sclater & Salvin, 1873)

3B59FB83-53FE-5F5C-BCE2-D390420A8467

##### Distribution

Anzoategui, Cajamarca, Herveo, Ibagué, Murillo, Roncesvalles

##### Notes

(au ML25990061; co CZUTOR 0742; ph)

#### Mecocerculus stictopterus

(Sclater, 1859)

12D05C9F-3ABE-558E-9010-022F01AA227B

##### Distribution

Anzoátegui, Cajamarca, Herveo, Ibagué, Libano, Murillo, Roncesvalles, Saldaña, Santa Isabel, Vilahermosa

##### Notes

(v; co SIB 6702; ph ML99049501)

#### Mecocerculus leucophrys

(d'Orbigny & Lafresnaye, 1837)

54A980A2-A33C-59B7-8D0A-CD11962D0F93

##### Distribution

Anzoátegui, Cajamarca, Chaparral, Falan, Herveo, Ibagué, Libano, Murillo, Roncesvalles, Santa Isabel

##### Notes

(v; au XC96385; co CZUTOR 1265; ph ML32219331)

#### Mecocerculus minor

(Taczanowsky, 1879)

63C439F6-B312-5560-9785-510B1BF109DD

##### Distribution

Anzoátegui, Cajamarca, Chaparral, Ibagué, Libano, Murillo

##### Notes

(v; co CZUTOR 0233; ph ML96881941)

#### Phyllomyias griseiceps

(Sclater & Salvin, 1871)

AFE12CBD-E507-5881-9B02-947650EF9340

##### Distribution

Armero-Guayabal, Cajamarca, Casabianca, Cunday, Doima, Falan, Fresno, Honda, Ibagué, Lerida, Mariquita, Melgar, Palocabildo, Planadas, Prado, Rio Blanco, Venadillo

##### Notes

(v; au XC424614; co AMHN 132239; ph ML123883071)

#### Phyllomyias plumbeiceps

(Lawrence, 1869)

7EEA3133-47C2-5334-A233-D5D418402A91

##### Distribution

Ibagué, Falan, Villarrica, Planadas

##### Notes

(v; au)

#### Acrochordopus zeledoni

(Lawrence, 1869)

D6C91EEF-282D-58D5-B0CB-40F75CD2ACF2

##### Distribution

Ibagué, Honda

##### Notes

(au ML58939131 ph ML58939041) This unusual species in the Department was observed for the first time on 21 May 2017 in Charcorrico (Parra-Hernández and Arias-Moreno 2019).

#### Tyranniscus cinereiceps

(Sclater, 1860)

8B476477-13F0-53CF-A2AA-A78049B9069F

##### Distribution

Cajamarca, Herveo, Ibagué, Libano, Roncesvalles

##### Notes

(v; co CZUTOR 0041)

#### Tyranniscus nigrocapillus

(Lafresnaye, 1845)

643B1015-AB51-548B-B8F6-7DE47A7A904E

##### Distribution

Cajamarca, Cunday, Falan, Herveo, Ibagué, Libano, Melgar, Murillo, Planadas, Prado, Roncesvalles, Villarrica

##### Notes

(v; co CZUTOR 0595; ph ML 22279311)

#### Tyranniscus uropygialis

(Lawrence, 1869)

D2DC2691-DF7D-58FF-874C-89592664F145

##### Distribution

Herveo, Ibagué, Melgar, Planadas, Roncesvalles

##### Notes

(v; co SIB, MHN- ICN 26179)

#### Camptostoma obsoletum

(Temminck, 1824)

D11AE82A-F6DB-5C0C-B3F5-566AF664E006

##### Distribution

Alpujarra, Alvarado, Ambalema, Armero-Guayabal, Ambalema, Carmen de Apicala, Chaparral, Cunday, Doima, Espinal, Falan, Fresno, Ibagué, Lerida, Planadas, San Luis, Venadillo, Villarrica

##### Notes

(v; au XC335017; ca; co MPUJ 505, CZUTOR 0197; ph ML123884401)

#### Nesotriccus murinus

(Spix, 1825)

8E6520BA-52A7-5F7D-BE07-975772437F40

##### Distribution

Ambalema, Armero-Guayabal, Alvarado, Carmen de Apicala, Chaparral, Chicoral, Coyaima, Doima, Espinal, Falan, Fresno, Honda, Guamo, Ibagué, Icononzo, Guamo, Lerida, Libano, Mariquita, Melgar, Natagaima, Planadas, Prado, San Luis, Venadillo, Villarrica

##### Notes

(v; au XC420171; ca; co MLS 5026, CZUT OR1483; ph ML105911651)

#### Pseudocolopteryx acutipennis

(Sclater & salvin, 1873)

28AA15A5-D5F2-5F92-83CD-7C07FCD8B291

##### Distribution

Ibagué, Roncesvalles

##### Notes

(v; co CZUTOR 1084; ph ML70968771)

#### Serpophaga cinerea

(Tschudi, 1844)

801A5DEA-D2A3-506B-82D3-50974B85F684

##### Distribution

Casabianca, Chaparral, Falan, Fresno, Herveo, Ibagué, Libano, Murillo, San Antonio, Venadillo, Villarrica, Villahermosa

##### Notes

(v; au XC423025; co; ph ML123893531)

#### Uromyias agilis

(Sclater, 1856)

DD764073-E35B-5073-969F-02FD5FE16176

##### Distribution

Cajamarca, Chaparral, Herveo, Ibagué, Roncesvalles

##### Notes

(v; au ML97304141; co MHN-ICN 5754, CZUT OR1270) CE

#### Legatus leucophaius

(Viellot, 1818)

E0529B4A-717C-5596-A8F9-E37912F6D4DC

##### Distribution

Alpujarra, Alvarado, Armero-Guayabal, Ambalema, Ataco, Chaparral, Cunday, Doima, Espinal, Falan, Fresno, Honda, Ibagué, Lerida, Libano, Mariquita, Melgar, Natagaima, Prado, Rovira, Santa Isabel, Venadillo, Villarrica, San Antonio

##### Notes

(v; au XC424493; ca; co MLS 447; ph ML24062881)

#### Pitangus sulphuratus

(Linnaeus, 1766)

7541603E-7062-5568-A5DE-528A527562ED

##### Distribution

Alvarado, Armero-Guayabal, Anzoategui, Ambalema, Ataco, Cajamarca, Casabianca, Carmen de Apicala, Chaparral, Coello, Coyaima, Cunday, Dolores, Espinal, Guamo, Falan, Fresno, Guamo, Honda, Ibagué, Icononzo, Lerida, Libano, Natagaima, Mariquita, Melgar, Murillo, Palocabildo, Planadas, Prado, Purificación, Rio Blanco, Saldaña, San Luis, Santa Isabel, Suarez, Venadillo, Villahermosa, Villarrica, San Antonio

##### Notes

(v; au XC424597; ca; co MLS 454, CZUTOR 447; ph ML123874091) WD

#### Philohydor lictor

(Lichtenstein, 1823)

572C52BD-BA76-575D-BB0D-17CFAE8FE456

##### Distribution

Armero-Guayabal, Alvarado, Doima, Espinal, Guamo, Ibagué, Mariquita, Piedras, Prado, San Luis, Venadillo

##### Notes

(v; au; ph ML123874341). New record - this flycatcher is observed in riparian forest and adjacent semi-open areas, lagoon complexes and streams below 1000 m a.s.l. in different areas of the Department (Fig. 10a).

#### Machetornis rixosa

(Vieillot, 1819)

3AA8873E-D196-5CC9-ABF7-D7978F988D56

##### Distribution

Alvarado, Ambalema, Armero-Guayabal, Alvarado, Coyaima, Coello, Doima, Espial, Falan, Fresno, Guamo, Herveo, Honda, Ibagué, Lerida, Murillo, Palocabildo, Planadas, Prado, San Luis, Suarez, Valle de San Juan, Venadillo, Villahermosa, Villarrica

##### Notes

(v; co CZUTOR 0789; ph ML ML123874571)

#### Megarhynchus pitangua

(Linnaeus, 1766)

A7FFEED0-D2C3-5074-B10F-827E7448A44B

##### Distribution

Ambalema, Alvarado, Armero-Guayabal, Ataco, Chicoral, Cunday, Doima, Espinal, Falan, Fresno, Guamo, Honda, Ibagué, Lerida, Mariquita, Melgar, Natagaima, Planadas, Purificación, Rio Blanco, Rovira, San Antonio, San Luis, Suarez, Valle de San Juan, Venadillo

##### Notes

(v; au XC424865; ca; co MLS 8147, CZUTOR 33; ph ML123872011)

#### Myiodynastes hemichrysus

(Cabanis, 1861)

80AA8379-854C-57D1-9FA1-1275121B30A1

##### Distribution

Cajamarca, Casabianca, Chaparral, Falan, Herveo, Ibagué, Libano, Prado, Planadas, Roncesvalles, Santa Isabel, Villahermosa

##### Notes

(v; au; co ANSP 153606; ph ML123878341)

#### Myiodynastes luteiventris

Sclater, 1859

40EE9B47-412A-5A2B-A117-82344A2B57FE

##### Distribution

Alvarado, Anzoátegui, Armero-Guayabal, Chaparral, Falan, Fresno, Honda, Ibagué, Libano, Mariquita, Planadas,Venadillo

##### Notes

(v; co AMHN 112316) M

#### Myiodynastes maculatus

(Statius Muller, 1776)

D602925A-68D6-538F-9784-58ACCC766DA2

##### Distribution

Alvarado, Ambalema, Anzoategui, Armero-Guayabal, Ataco, Chaparral, Chicoral, Coyaima, Doima, Espinal, Guamo, Herveo, Honda, Ibagué, Falan, Fresno, Lerida, Libano, Mariquita, Melgar, Planadas, Prado, Rio Blanco, Saldaña, San Luis, San Antonio, San Luis, Santa Isabel, Suarez, Venadillo, Villarica

##### Notes

(v; au XC424489; ca; co MLS 4427; ph ML123878031) M

#### Myiozetetes cayanensis

(Linnaeus, 1766)

3BABB138-2AA1-56CD-AF59-E613AD88853E

##### Distribution

Alvarado, Armero-Guayabal, Ambalema, Casabianca, Carmen de Apicala, Coyaima, Cunday, Dolores, Espinal, Guamo, Falan, Fresno, Honda, Ibagué, Libano, Mariquita, Melgar, Palocabildo, Prado, Planadas, Venadillo, Villarrica, San Antonio

##### Notes

(v; au XC439770; co MLS 4451, CZUTOR 290; ph ML123873881)

#### Myiozetetes similis

(Spix, 1825)

2B8360E5-C8A3-57D7-91C4-62F4C47B5289

##### Distribution

Armero-Guayabal, Alvarado, Casabianca, Chaparral, Coyaima, Cunday, Doima, Dolores, Espinal, Falan, Guamo, Gualanday, Honda, Ibagué, Lerida, Natagaima, Mariquita, Planadas, Prado, Purificación, Rio Blanco, Rovira, San Luis, Venadillo, Villarrica

##### Notes

(v; au; co MLS 4479, CZUTOR 272; ph ML72671421) WD

#### Myiozetetes granadensis

Lawrence, 1862

A30057BE-8420-5764-BB24-BAB22BBA528B

##### Distribution

Coyaima

##### Notes

(co AMHN 71870)

#### Conopias cinchoneti

(Tschudi, 1844)

142314F2-D882-5C8E-ADCF-76C8CF4A7BD6

##### Distribution

Chaparral, Ibagué, Planadas, Rio Blanco

##### Notes

(v) an individual Photographed by J. Sanabría in Juntas (Ibagué) on 27 Aug 2016 ML 343821781.

#### Empidonomus varius

(Vieillot, 1818)

95FA5A93-3DAB-53BE-83AE-47B6512CDF65

##### Distribution

Armero-Guayabal. Ibagué, Lerida, San Luis

##### Notes

(v) M

#### Tyrannus melancholicus

Vieillot, 1819

77F8149E-488F-58C3-9AA3-5ACE36899E39

##### Distribution

Armero-Guayabal, Anzoátegui, Ambalema, Ataco, Casabianca, Chaparral, Carmen de Apicala, Chicoral, Coyaima, Cunday, Doima, Espinal, Ibagué, Icononzo, Guamo, Herveo, Honda, Falan, Lerida, Libano, Mariquita, Melgar, Murillo, Planadas, Purificación, Rio Blanco, San Antonio, Santa Isabel, San Luis, Venadillo, Villarrica, Villahermosa

##### Notes

(v; au XC423704; ca co CZUTOR 0648; ph ML72671521)

#### Tyrannus savana

Vieillot, 1808

3F8AF955-9D78-5495-A4EB-483F74D79004

##### Distribution

Ambalema, Armero-Guayabal, Alvarado, Ataco, Cajamarca, Carmen de Apicala, Chaparal, Chicoral, Coyaima, Cunday, Doima, Dolores, Espinal, Herveo, Ibagué, Falan, Fresno, Guamo, Honda, Lerida, Libano, Natagaima, Mariquita, Melgar, Murillo, Planadas, Prado, Purificación, Rio Blanco, Roncesvalles, San Luis, Valle de San Juan, Villahermosa, Villarrica

##### Notes

(v; co MLS 4361; ph ML111646811) M

#### Tyrannus tyrannus

(Linnaeus, 1758)

87B493A0-F570-5503-AB26-E336748D2B97

##### Distribution

ALvarado, Ambalema, Armero-Guayabal, Casabianca, Chaparral, Carmen de Apicala, Coello, Doima, Dolores, Espinal, Ibagué, Falan, Fresno, Herveo, Honda, Lerida, Libano, Mariquita, Palocabildo, Planadas, Prado, Saldaña, Suarez, Valle de an Juan, Venadillo

##### Notes

(v; co MHN-ICN 5735, MLS 1765; ph ML42732701) M

#### Tyrannus dominicensis

(Gmelin, 1788)

9F7CFC27-DE5B-5A78-952B-121F2B3F038C

##### Distribution

Alvarado, Espinal, Falan, Ibagué, Honda, Saldaña

##### Notes

(v; co AMHN 94960) M

#### Myiarchus tuberculifer

(d'Orbigny & Lafresnaye, 1837)

233A1503-C8CF-572C-AD05-88AA38752876

##### Distribution

Alvarado, Armero-Guayabal, Cunday, Fresno, Honda, Ibagué, Libano, Mariquita, Prado, Santa Isable, Venadillo, Villarrica

##### Notes

(v; au XC424030; co CZUTOR 333 ca; ph ML79639071)

#### Myiarchus panamensis

Lawrence, 1860

3540D484-093C-55D2-B15F-E934C0F4D3A6

##### Distribution

Alvarado, Armero-Guayabal, Casabianca, Chaparral, Carmen de Apicala, Coyaima, Espinal, Guamo, Falan, Fresno, Honda, Ibagué, Libano, Mariquita, Melgar, San Luis, Saldaña, Venadillo

##### Notes

(ca; co MLS 4533, CZUT OR1236; ph ML123876001)

#### Myiarchus apicalis

Sclater & Salvi, 1881

07269EA3-60FF-5160-A36C-92672E5F21FC

##### Distribution

Armero-Guayabal, Ataco, Chaparral, Coyaima, Cunday, Doima, Dolores, Espinal, Guamo, Gualanday, Falan, Honda, Ibagué, Libano, Mariquita, Melgar, Planadas, Prado, Rio Blanco, Rovira, San Luis, Venadillo

##### Notes

(v; au; ca; co MLS 4561, CZUTOR 182; ph ML123880191) E. This flycatcher is usually seen at heights up to 1400 m a.s.l. in open areas and areas with shrubs and occasionally in areas with crops. It has been observed throughout the lower areas of Tolima (Fig. 9f).

#### Myiarchus cephalotes

Taczanowski, 1880

2B690995-D630-51F3-B8BC-D405FF6230AD

##### Distribution

Casabianca, Cajamarca, Chaparral, Herveo, Ibagué, Icononzo,Falan, Lerida, Libano, Murillo, Palocabildo, Santa Isabel, Roncesvalles

##### Notes

(v; au XC423691; ca; co CZUTOR 0846; ph ML120521251)

#### Myiarchus crinitus

(Linnaeus, 1758)

8FD34206-F346-5757-9B62-6F06D815CA87

##### Distribution

Alvarado, Armero- Guayabal, Chaparral, Falan, Fresno, Ibagué, Lerida, Libano, Prado, San Luis, Venadillo, Villarrica

##### Notes

(v; au ML86923331; co DNHM 56699; ph ML24063581)

#### Myiarchus tyrannulus

(Statius Muller, 1776)

84518073-2D0A-5445-8474-3A8D6AF5DFBD

##### Distribution

Armero-Guayabal, Espinal, Falan, Ibagué, Honda,Melgar, Prado, Venadillo

##### Notes

(v; co MLS 7787)

#### Colonia colonus

(Vieillot, 1818)

4FCEBBD3-9A7A-5594-A44C-6C461D55C8D1

##### Distribution

Ambalema, Armero-Guayabal, Carmen de Apicala, Chaparral, Cunday, Falan, Fresno, Honda, Mariquita, Melgar, Prado, Venadillo

##### Notes

(v; co MHN-ICN 4430; ph ML123923421)

#### Myiophobus flavicans

(Sclater, 1861)

703C4DC3-D216-5EEA-B7A1-2455ED7F643B

##### Distribution

Cajamarca, Chaparral, Cunday, Ibagué, Falan, Libano, Murillo, Planadas, Rio Blanco, Roncesvalles, San Antonio, Villarrica

##### Notes

(v; ca; co CZUTOR 0309; ph)

#### Myiophobus fasciatus

(Statius Muller, 1776)

8592F8AD-C081-580D-A370-DEB63043DE3B

##### Distribution

Alvarado, Armero-Guayabal, Casabianca, Chaparral, Doima, Falan, Honda, Ibagué, Icononzo, Libano, Planadas, Prado, Rio Blanco, Villarrica, Villahermosa, Villarrica

##### Notes

(v; au ML82121; co CZUTOR 0392; ph ML105235981)

#### Silvicultrix frontalis

(Lafresnaye, 1847)

496C402A-A71A-58E7-9FE7-D3D64CF7E271

##### Distribution

Cajamarca, Ibagué, Murillo, Santa Isabel

##### Notes

(v; ca; co SIB 126763; ph)

#### Silvicultrix diadema

(Hartlaub, 1843)

B2C2CC62-489E-5F34-A29A-849033B34E7F

##### Distribution

Cajamarca, Herveo, Ibagué, Murillo, Santa Isabel, Villarrica

##### Notes

(v; ph ML115221471)

#### Ochthoeca cinnamomeiventris

(Lafresnaye, 1843)

45060271-0172-523D-8C28-A9856F80D232

##### Distribution

Anzoátegui, Cajamarca, Chaparral, Herveo, Ibagué, Falan, Libano, Murillo, Planadas, Santa Isabel, Roncesvalles, Villahermosa, Villarrica

##### Notes

(v; au; co CZUTOR 189; ph ML123893141)

#### Ochthoeca rufipectoralis

(d'Orbigny & Lafresnaye, 1837)

669B5EEA-5CC0-546D-8213-B6D0DD61B860

##### Distribution

Anzoátegui, Cajamarca, Fresno, Herveo, Ibagué, Murillo, Roncesvalles

##### Notes

(v; co ISB 126290; ph ML114075331)

#### Ochthoeca fumicolor

Sclater, 1856

CC4781CA-C3A5-5F2D-B75E-52AF4FD5396A

##### Distribution

Anzoátegui, Cajamarca, Chaparral, Herveo, Ibagué, Murillo, Rio Blanco, Roncesvalles, San Antonio, Santa Isabel

##### Notes

(v; ca; co CZUTOR 0291; ph ML115212671)

#### Pyrocephalus rubinus

(Boddaert, 1783)

8FD37D50-3A19-5C48-8503-8C1496955C50

##### Distribution

Alvarado, Armero-Guayabal, Ambalema, Ataco, Carmen de Apicala, Casabianca, Chaparral, Chicoral, Coyaima, Cunday, Doima, Espinal, Guamo, Honda, Ibagué, Icononzo, Falan, Flandes, Fresno, Lerida, Libano, Mariquita, Melgar, Murillo, Planadas, Purificación, Rovira, Saldaña, San Anotonio, San Luis, Santa Isabel, Suarez, Valle de San Juan, Venadillo, Villahermosa, Villarrica

##### Notes

(v; au XC425867 co MLS 4338; ph ML123877541) WD

#### Fluvicola pica

(Boddaert, 1783)

922E6F5C-CA9D-581B-9FED-3A78CDCF49C5

##### Distribution

Ambalema, Armero-Guayabal, Ataco, Coello, Coyaima, Doima, Espinal, Guamo, Falan, Flandes, Honda, Ibagué, Natagaima, Mariquita, Melgar, Libano, Prado, Purificación, Roncesvalles, Saldaña, San Luis, Venadillo, Villarrica

##### Notes

(v; ca; co MLS 4318, CZUTOR 759; ph ML123874881)

#### Arundinicola leucocephala

(Linnaeus, 1764)

933068D3-01BF-5D4B-A890-669D62E4CCC5

##### Distribution

Ambalema, Alvarado, Armero-Guayabal, Ataco, Coyaima, Doima, Lerida, Melgar, Natagaima, Honda, Ibagué, Prado, Purificación, Saldaña

##### Notes

(v; co MHN-ICN 7038, CZUTOR 815; ph ML123881071)

#### Knipolegus poecilurus

(Sclater, 1862)

D27A83EE-3A68-5C06-BF07-C43B47E8C901

##### Distribution

Casabianca, Ibagué, Libano, Roncesvalles, Villarrica

##### Notes

(v ca co CSJ 133781ph)

#### Muscisaxicola alpinus

(Jardine, 1849)

D01A32AD-2544-5010-96AB-D05ECFD21C19

##### Distribution

Anzoátegui, Herveo, Ibagué, Murillo, Roncesvalles, Santa Isabel, Villahermosa

##### Notes

(co MHN-ICN 4506, 20450; ph ML78852391)

#### Myiotheretes striaticollis

(Sclater, 1853)

44DAE5B4-EE9B-59BE-86D9-D089542F8F9B

##### Distribution

Anzoátegui, Cajamarca, Ibagué, Murillo, Planadas, Roncesvalles, Santa Isabel

##### Notes

(v; au ML28978891; co ANSP 153601; ph ML115212851)

#### Myiotheretes fumigatus

(Boissoneau, 1840)

E113A92C-0ABF-5B76-9ED8-C68877FA4BA8

##### Distribution

Anzoátegui, Cajamarca, Herveo, Ibagué, Murillo, Roncesvalles, Santa Isabel

##### Notes

(v; co SIB 126778, CZUTOR 700; ph)

#### Cnemotriccus fuscatus

(Wied-Neuwied, 1831)

CF19F3EA-45C7-51D0-A5EA-0FC8C0814CAF

##### Distribution

Armero-Guayabal, Carmen de Apicala, Chaparral, Espinal, Falan, Fresno, Ibagué, Libano, Mariquita, Palocabildo, Planadas, San Luis, Venadillo

##### Notes

(v; ca; co MLS 4624, CZUTOR 483; ph ML123884681)

#### Sayornis nigricans

(Swainson, 1827)

488F3BD9-FF60-5273-ACDD-CF08C40821B9

##### Distribution

Alvarado, Armero-Guayabal, Cajamarca, Chaparral, Cunday, Doima, Espinal, Falan, Fresno, Herveo, Honda, Ibagué, Icononzo, Lerida, Libano, Natagaima, Mariquita, Melgar, Murillo, Planadas, Prado, Purificación, Rio Blanco, Roncesvalles, San Luis, Saldaña, Venadillo, Villarrica,

##### Notes

(v; co MLS 8003, CZUTOR 303; ph ML123879601)

#### Empidonax virescens

(Vieillot, 1818)

5E8475A4-33EC-5785-A4E3-ED03502EE17F

##### Distribution

Chaparral, Doima, Falan, Ibagué, Lerida, Piedras, Planadas, San Luis, Villarrica

##### Notes

(v; co CZUTOR 0818) M

#### Empidonax traillii

(Audubon, 1828)

987F146A-6DB7-5887-97F2-7DF8044CAB0A

##### Distribution

Alvarado, Carmen de Apicala, Doima, Falan, Fresno, Honda, Ibagué, Libano, Mariquita

##### Notes

(v; co MHN-ICN 26167; ph) M

#### Empidonax alnorum

Brewster, 1895

7431F02C-CE69-5482-85E9-1A6E08236440

##### Distribution

Carmen de Apicala, Espinal, Ibagué, Honda, Libano, Villarrica

##### Notes

(ca; co MHN-ICN 4423) M

#### Contopus cooperi

(Nutall, 1831)

BCCA2DC9-BFE9-5CCE-A816-9FE81B61586A

##### Distribution

Cajamarca, Honda, Ibagué, Falan, Fresno, Libano, Planadas, San Antonio, San Luis, Villarrica

##### Notes

(v; ph ML99189971) NT M

#### Contopus fumigatus

(d'Orbigny & Lafresnaye, 1837)

6C6EBB8C-0598-5EB1-95F5-821E3307ACA5

##### Distribution

Anzoátegui, Cajamarca, Casabianca, Cunday, Falan, Ibagué, Herveo, Libano, Murillo, Santa Isabel, Villahermosa, Villarrica, Planadas

##### Notes

(v; au XC335997; co CZUTOR 0645; ph ML43403191)

#### Contopus sordidulus

Sclater, 1859

821F016A-FA51-586A-A02C-7143474EEBEC

##### Distribution

Armero- Guyabal, Ibagué, Melgar, Murillo, San Luis, Venadillo, Villarrica

##### Notes

(v; ca; co MHN-ICN 4425) NT M

#### Contopus virens

(Linnaeus, 1766)

381D2BCE-9DD7-53E8-B9F0-ACCB65BD9CBB

##### Distribution

Armero-Guayabal, Chaparral, Espinal, Falan, Fresno, Honda, Ibagué, Libano, Mariquita, Murillo, Planadas, Rio Blanco, Villahermosa

##### Notes

(v; au XC364398; ca; co MHN-ICN 4427, CZUTOR 0702) M

#### Contopus cinereus

(Spix, 1825)

00B2BD03-FD7E-531D-AB27-390D2DCEBA2B

##### Distribution

Alvarado, Armero-Guayabal, Ataco, Chaparral, Doima, Falan, Fresno, Honda, Ibagué, Icononzo, Lerida, Libano, Mariquita, Melgar, Planadas, Venadillo, Villahermosa

##### Notes

(v; ca; co MHN-ICN 31684)

#### Cyclarhis gujanensis

(Gmelin, 1789)

7F96327C-7865-509D-8E7F-3A43CB10F613

##### Distribution

Armero-Guayabal, Cajamarca, Casabianca, Carmen de Apicala, Chaparral, Cunday, Dolores, Espinal, Falan, Fresno, Herveo, Venadilloo, Honda, Icononzo, Ibagué, Mariquita, Melgar, Natagaima, Planadas, Prado, Purificación, Saldaña, San Luis, Santa Isabel, Suarez, Venadillo, Villarrica

##### Notes

(v; au XC424453; co MLS 7964, CZUTOR 897; ph ML123928241)

#### Cyclarhis nigrirostris

Lafresnaye, 1842

07ED271F-C73C-5C26-9B15-598288BEE60A

##### Distribution

Anzoátegui, Falan, Herveo, Ibagué, Murillo, Planadas, Roncesvalles, Villahermosa

##### Notes

(v; au XC421237; ML96502071; ca co CZUTOR 0057; ph)

#### Hylophilus flavipes

Lafresnaye, 1845

2A0D77DA-7B0A-5354-AD7C-FF1C4DF5DDD4

##### Distribution

Anzoategui, Ambalema, Alvarado, Armero-Guayabal, Doima, Carmen de Apicala, Chicoral, Coyaima, Chaparral, Dolores, Espinal, Falan, Fresno, Gualanday, Guamo, Honda, Ibagué, Icononzo, Lerida, Libano, Mariquita, Melgar, Natagaima, Planadas, Prado, Purificación, Saldaña, Santa Isabel, Rio Blanco, San Luis, Venadillo, Villarrica

##### Notes

(au XC424394; ca; co MLS 5795, CZUTOR; ph ML123831561)

#### Vireolanius eximius

Baird, 1866

F9085249-8815-5DC3-BEF8-F5C5B2F01AB2

##### Distribution

Casabianca, Falan, Mariquita, Palocabildo

##### Notes

(v; au)

#### Pachysylvia decurtata

(Bonaparte, 1838)

9F31A3F5-BF83-5778-8C75-435F12A4D728

##### Distribution

Ibagué, Mariquita, Melgar, Falan

##### Notes

(v; co WFVZ SS-42967)

#### Pachysylvia semibrunnea

Lafresnaye, 1845

FD6A2988-67D4-5ED9-B002-451B96D51587

##### Distribution

Ataco, Chaparral, Cunday, Falan, Fresno, Herveo, Ibagué, Icononzo, Libano, Mariquita, Planadas, Rio Blanco, San Luis, Santa Isabel, Villahermosa

##### Notes

(v; au-XC424034; co CZUTOR 282; ca; ph)

#### Vireo flavifrons

Vieillot, 1808

088DA4B1-9E0D-5B2B-9907-1334B11FDFE1

##### Distribution

Fresno, Ibagué, Libano, Mariquita, San Luis

##### Notes

(v; co) M

#### Vireo philadelphicus

(Cassin, 1851)

DAD47EAC-FA5F-5FB9-B6F4-D12603135075

##### Distribution

Ibagué, Falan, San Luis

##### Notes

(v; ca; co; ph) M

#### Vireo leucophrys

(Lafresnaye, 1844)

2671499D-37AE-5C33-BF66-F0F5FBB9332C

##### Distribution

Cajamarca, Chaparral, Cunday, Falan, Herveo, Ibagué, Melgar, Villahermosa

##### Notes

(v; au XC424496; ca; co CZUTOR 055; ph ML79639131)

#### Vireo olivaceus

(Linnaeus, 1766)

65B5A51D-D0E9-56B4-8D94-FC695E383FDA

##### Distribution

Armero- Guayabal, Chaparral, Doima, Espinal, Falan, Fresno, Herveo, Honda, Ibagué, Icononzo, Casabianca, Chaparral, Lerida, Mariquita, Melgar, Piedras, Planadas, San Luis

##### Notes

(v; ca; co MPUJ 740, CZUTOR 611; ph ML117807641) M

#### Vireo flavoviridis

(Cassi, 1851)

EDF657BA-C5A3-5E56-8EB4-34932A1CD58F

##### Distribution

Alvarado, Armero-Guayabal, Chaparral, Coyaima, Doima, Falan, Fresno, Ibagué, Lérida, Libano, Mariquita, Melgar, San Luis, Valle de San Juan, Venadillo

##### Notes

(v; co MHN-ICN 5437; ph ML123890351) M. There are a few records for this species in the Upper Magdalena Valley. We captured an individual on 6 September 2015 in the Municipality of San Luis (record from eBird). It is regularly observed in Falan between October and January.

#### Vireo altiloquus

(Vieillot, 1808)

E3EA6F33-8DE9-5BDF-9C51-DD7EA275E30B

##### Distribution

Falan, Prado

##### Notes

(v) M

#### Cyanolyca armillata

(Gray, 1845)

4DAB4E72-66FB-5C25-8571-93193A6097D2

##### Distribution

Anzoátegui, Cajamarca, Herveo, Ibagué, Murillo, Planadas, Roncesvalles, San Antonio, Santa Isabel, Villahermosa

##### Notes

(v; au; co SIB A6826; ph ML78850691)

#### Cyanocorax affinis

Pelzeln, 1856

14A0F6F2-5DE4-5E48-B66C-115ECAA60200

##### Distribution

Alpujarra, Alvarado, Ambalema, Armero-Guayabal, Ataco, Carmen Apicala, Chaparral, Coyaima, Cunday, Doima, Espinal, Gualanday, Falan, Fresno, Honda, Ibagué, Lerida, Libano, Mariquita, Melgar, Natagaima, Prado, Purificación, San Luis, Suarez, Venadillo, Villarrica

##### Notes

(v; au XC424495; co MLS 5222; ph ML123879711)

#### Cyanocorax yncas

(Boddaert, 1783)

9C4F736A-5DDE-5E51-BDB3-806BB4758179

##### Distribution

Anzoátegui, Ataco, Cajamarca, Falan, Icononzo, Herveo, Ibagué, Libano, Murillo, Planadas, Rio Blanco, Roncesvalles, San Antonio, Santa Isabel, Villarrica

##### Notes

(v; au XC421112; co ANSP 154818; ph ML115213281)

#### Pygochelidon cyanoleuca

(Vieillot, 1817)

A9A9C79B-5ABF-5732-8227-9396DD7F6D38

##### Distribution

Alpujarra, Ambalema, Anzoátegui, Armero-Guayabal, Ataco, Carmen de Apicala, Chaparral, Cunday, Dolores, Falan, Fresno, Herveo, Ibagué, Icononzo, Lerida, Libano, Mariquita, Murillo, Natagaima, San Luis, Planadas, Prado, Rio Blanco, San Antonio, Santa Isabel, Saldaña, Villarrica, Villahermosa

##### Notes

(v; co ISB 126317, CZUTOR 385; ph ML123830491) WD

#### Oreochelidon murina

(Cassin, 1853)

BACF81A0-F689-50F9-8763-59B1C92F7387

##### Distribution

Anzoátegui, Cajamarca, Casabianca, Cunday, Herrera, Herveo, Ibagué, Libano, Murillo, Planadas, Prado, San Antonio, Roncesvalles, Villarrica

##### Notes

(v; ca; co SIB 126292, CZUTOR 002; ph ML123830221)

#### Oreochelidon flavipes

(Chapman, 1922)

377B8BEC-3F27-53A9-8BE0-0591B2D2B953

##### Distribution

Cajamarca, Ibagué, Murillo

##### Notes

(v; co ANSP 154234)

#### Atticora tibialis

(Cassin, 1853)

FA93DE0D-9CEB-5AF1-BE75-3776127B6902

##### Distribution

Casabianca, Herveo, Ibagué, Libano, Falan, Fresno

##### Notes

(v; ph ML123829391)

#### Stelgidopteryx ruficollis

(Vieillot, 1817)

FF67B247-A9E4-590C-9D69-9DD763E7A8ED

##### Distribution

Alvarado, Ambalema, Armero-Guayabal, Ataco, Cajamarca, Casabianca, Chaparral, Cunday, Doima, Espinal, Herveo, Honda, Ibagué, Icononzo, Guamo, Falan, Fresno,, Lerida, Libano, Mariquita, Palocabildo, Planadas, Prado, Purificación, Rio Blanco, San Antonio, San Luis, Valle de San Juan, Suarez, Venadillo, Villahermosa, Villarrica

##### Notes

(v; ca; co CZUTOR 008; ph ML123827941) WD

#### Progne tapera

(linnaeus, 1766)

CF1A330D-C961-5611-910C-3819AC3F973E

##### Distribution

Alvarado, Ambalema, Armero-Guayabal, Chaparral, Espinal, Honda, Ibagué, Libano, Mariquita, Melgar, Natagaima, Prado, Purificación, San Luis, Valle de San Juan, Venadillo, Villarrica

##### Notes

(v; ph ML 197917961) M

#### Progne subis

(Linnaeus, 1758)

E384577A-5FD6-5E75-B978-4078026ABC5A

##### Distribution

Ambalema, Ibagué

##### Notes

(ph ML718291)

#### Progne chalybea

(Gmelin, 1789)

F932F7CA-BB3A-561E-8B10-4BC66CEFAA59

##### Distribution

Ambalema, Alvarado, Armero-Guayabal, Chaparral, Doima, Espinal, Guamo, Honda, Ibagué, Falan, Fresno, Lerida, Mariquita, Melgar, Natagaima, Ortega, Prado, Rovira, Saldaña, San Luis, Venadillo

##### Notes

(v; CZUT OR1452; ph ML123827721) M

#### Tachycineta albiventer

(Boddaert, 1783)

3746D2A6-2192-592F-A284-070997FA5BF4

##### Distribution

Ambalema, Armero-Guayabal, Cajamarca, Chaparral, Doima, Coyaima, Cunday, Guamo, Ibagué, Honda, Libano, Mariquita, Natagaima, Planadas, Prado, Purificación, Saldaña, San Luis, Venadillo

##### Notes

(co; ph ML104428641 v)

#### Riparia riparia

(Linnaeus, 1758)

731D1375-9FDB-585A-B93F-E5DF492F3796

##### Distribution

Alvarado, Falan, Guamo, Ibagué, Lerida, San Luis, Villarrica

##### Notes

(v; co; ph) M

#### Hirundo rustica

(Linnaeus, 1758)

05DBF741-1754-5952-A005-F06DE3320DC8

##### Distribution

Armero-Guayabal, Alvarado, Cajamarca, Doima, Falan, Guamo, Honda, Ibagué, Lerida, Libano, Mariquita, Honda, Prado, Purificación, Saldaña, Villarrica

##### Notes

(v; co; ph ML123827241) M

#### Petrochelidon pyrrhonota

(Vieillot, 1817)

3A29B9FB-283F-537A-BAFF-CF94A309BCA0

##### Distribution

Armero-Guayabal, Doima, Ibagué, Libano, Mariquita, San Luis

##### Notes

(v) M

#### Microcerculus marginatus

(Sclater, 1855)

0DC3A349-1578-54F4-A1F7-0B21E628D598

##### Distribution

Casabianca, Cunday, Dolores, Icononzo, Falan, Fresno, Libano, Mariquita, Melgar, Prado, Venadillo, Villarrica

##### Notes

(v; au; ca; co CZUTOR 0356; ph ML123824371)

#### Troglodytes musculus

Vieillot, 1809

02E4C1B1-0671-5365-A65C-1402552B2FB4

##### Distribution

Alvarado, Armero-Guayabal, Anzoategui, Ambalema, Cajamarca, Casabianca, Chaparral, Chicoral, Carmen de Apicala, Coyaima, Doima, Dolores, Espinal, Falan, Fresno, Guamo, Herveo, Honda, Ibagué, Icononzo, Lerida, Libano, Mariquita, Murillo, Ortega, Planadas, Prado, Rio Blanco, Roncesvalles, Rovira, San Antonio, San Luis, Saldaña, Valle de San Juan, Venadillo, Villahermosa, Villarrica

##### Notes

(v; au XC424026; co MLS 5449, CZUTOR 331; ph ML123824591) WD

#### Troglodytes solstitialis

Sclater, 1859

D3792274-4AC3-50B0-B955-63C7966EF8F1

##### Distribution

Anzoátegui, Cajamarca, Chaparral, Ibagué, Murillo, Roncesvalles, San Antonio, Santa Isabel

##### Notes

(v; au ML28984741; co SIB 126390; ph ML114073211)

#### Cistothorus platensis

(Latham, 1790)

A34F9CE9-50F0-5567-AF20-0D83543BDA4C

##### Distribution

Anzoátegui, Casabianca, Cajamarca, Herveo, Ibagué, Murillo, Roncesvalles, Santa Isabel

##### Notes

(v; au-XC96379; co CZUTOR 1268; ph ML123901131)

#### Campylorhynchus zonatus

(Lesson, 1832)

FD470B94-1048-592E-A531-DA9213B80D62

##### Distribution

Armero-Guayabal, Casabianca, Falan, Fresno, Honda, Libano, Santa Isabel

##### Notes

(v; ph ML99192301) This is a moderately common bird in the north of Tolima (Falan, Líbano and Fresno) that has been recorded in open areas, bushes and near houses, frequently vocalising (Fig. 10e).

#### Campylorhynchus griseus

(Swainson, 1838)

E7E80EF0-394D-5DCD-8BA1-2C937999FAB1

##### Distribution

Alvarado, Ambalema, Armero- Guayabal, Coyaima, Doima, Dolores, Falan, Honda, Ibagué, Guamo, Libano, Planadas, Prado, Purificación, San Luis, Suarez, Villarrica

##### Notes

(v; au; co MHN-ICN 11931; ph ML123873491)

#### Pheugopedius spadix

Bangs, 1910

BFDF855C-AD18-59CE-A85A-B5CE0836CD6E

##### Distribution

Ibagué, Falan, Fresno, Libano

##### Notes

(au ML119598531)

#### Pheugopedius fasciatoventris

(Lafresnaye, 1845)

B8B3B262-275E-5414-9E08-98BB507C7CDA

##### Distribution

Ambalema, Alpujarra, Alvarado, Armero-Guayabal, Casabianca, Chaparral, Chicoral, Cunday, Doima, Dolores, Espinal, Guamo, Gualanday, Falan, Fresno, Honda, Ibagué, Lerida, Mariquita, Melgar, Natagaima, Ortega, Planadas, Prado, Purificación, Rio Blanco, Rovira, San Antonio, San Luis, Saldaña, Venadillo, Villarrica

##### Notes

(v; au XC424398; ca; co MLS 5365, CZUTOR 269; ph ML123888071)

#### Pheugopedius mystacalis

(Sclater, 1860)

9A32C503-99E7-550B-8DF5-B529385CD48D

##### Distribution

Anzoátegui, Casabianca, Cajamarca, Chaparral, Espinal, Falan, Fresno, Herveo, Ibagué, Libano, Murillo, Palocabildo, Planadas, Rio Blanco, Roncesvalles, San Antonio, Santa Isabel

##### Notes

(v; au XC424587, ML58032291; co 511; MLS 8314 ph)

#### Pheugopedius sclateri

(Taczanowski, 1879)

C83B4B8F-F7F8-54DA-A65F-22FF2D1F8974

##### Distribution

Cajamarca, Casabianca, Ibagué, Icononzo, Libano, Prado, Villarrica

##### Notes

(v; au; co AMHN 154524, CZUT OR1005; ph ML107748361)

#### Cantorchilus nigrocapillus

(Sclater, 1860)

293191A0-D6ED-517D-8EBF-5B62350D859D

##### Distribution

Casabianca, Ibagué, Falan, Fresno, Mariquita, Santa Isabel

##### Notes

(au; ph ML72672001) New record - this uncommon species of the Department is mainly observed and identified by its song. It is distributed from the north of the Department to the Municipality of Ibagué and seen at heights of about 800 to 1300 m a.s.l. (Fig. 10d).

#### Cantorchilus leucotis

(Lafresnaye, 1845)

17777594-1D4B-5D7D-9FD6-18808275BB9F

##### Distribution

Alvarado, Ambalema, Armero-Guayabal, Chaparral, Carmen de Apicala, Chicoral, Cunday, Doima, Espinal, Lerida, Libano, Gualanday, Guamo, Falan, Fresno, Honda, Ibagué, Lerida, Mariquita, Melgar, Prado, San Luis, Rovira, Venadillo, Villarrica

##### Notes

(v; au XC424616; ca; co MLS 5316, CZUTOR 0332; ph)

#### Cinnycerthia unirufa

(Lafresnaye, 1840)

586BC5B4-0D13-5FB1-BBE0-4C7896D30846

##### Distribution

Anzoátegui, Cajamarca, Herveo, Ibagué, Libano, Murillo, Santa Isabel, Roncesvalles, Villahermosa

##### Notes

(v; au ML103814321; co CZUTOR 1249)

#### Cinnycerthia olivascens

Sharpe, 1882

29596226-703C-50A9-A203-4FE7FA6636A2

##### Distribution

Cajamarca, Ibagué, Murillo

##### Notes

(v; au; co MHN-ICN 37880, CZUTOR 192; ph ML23813041)

#### Henicorhina leucosticta

(Cabanis, 1847)

4C5139B8-16AE-5653-A677-3D353C290947

##### Distribution

Armero-Guayabal, Ambalema, Alvarado, Doima, Ibagué, Libano, Mariquita, Honda, San Luis, Rio Blanco, San Antonio, Venadillo

##### Notes

(v; au; ca; co CZUTOR 0973; ph)

#### Henicorhina leucophrys

(Tschudi, 1844)

F6BB234C-16DB-5101-8717-45EFA1BCA78B

##### Distribution

Anzoátegui, Casabianca, Cajamarca, Chaparral, Dolores, Falan, Herveo, Honda, Ibagué, Icononzo, Libano, Murillo, Palocabildo, Planadas, Roncesvalles, San Antonio, San Luis, Villarrica, Villahermosa, Villarrica

##### Notes

(v; au XC424623, ML98610431; ca co CZUTOR 0186; ph ML115221401)

#### Cyphorhinus thoracicus

Tschudi, 1844

3E82A7B5-F445-5750-AC73-6B75E0B0C3A0

##### Distribution

Cajamarca, Casabianca, Falan, Ibagué, Icononzo, Santa Isabel

##### Notes

(v; au ML94669671; co AMHN 112564)

#### Ramphocaenus melanurus

Vieillot, 1819

DE0A5B4D-0A3A-5F77-9A65-EB24C89138EF

##### Distribution

Armero-Guayabal, Espinal, Falan, Fresno, Libano, Mariquita, Melgar, Venadillo, Villarrica

##### Notes

(v; au ML81658; co CZUTOR 1232; ph ML105060121)

#### Polioptila plumbea

(Gmelin, 1788)

3C11AAAD-50BB-55D2-B224-358CB4C19140

##### Distribution

Alvarado, Ambalema, Armero-Guayabal, Alvarado, Casabianca, Chaparral, Carmen de Apicala, Coyaima, Doima, Espinal, Guamo, Gualanday, Honda, Ibagué, Falan, Lerida, Libano, Mariquita, Melgar, Planadas, Prado, Purificación, Saldaña, San Luis, Venadillo

##### Notes

(v; au-XC96416; ca; co MLS 5730; ph ML123888531)

#### Donacobius atricapilla

(Linnaeus, 1766)

90516F43-1240-5634-B6F9-787D9BC07EF0

##### Distribution

Armero-Guayabal, Ambalema, Doima, Espinal, Fresno, Honda, Ibagué, Prado, Mariquita, Purificación, Venadillo

##### Notes

(v; co AMHN 122541; ph ML109227211)

#### Cinclus leucocephalus

Tschudi, 1844

8897D687-EDD9-54C3-8290-9C1DE1DB54A2

##### Distribution

Anzoátegui, Cajamarca, Chaparral, Fresno, Herrera, Herveo, Ibagué, Libano, Planadas, Roncesvalles, Santa Isabel, Villarrica

##### Notes

(v; au; co CZUTOR 1421; ph ML79639151)

#### Myadestes ralloides

(d'Orbigny, 1840)

2CE34457-CF12-5E24-84A1-1AFDF6768675

##### Distribution

Anzoátegui, Cajamarca, Casabianca, Cajamarca, Falan, Herveo, Ibagué, Libano, Murillo, Planadas, Prado, Roncesvalles, San Antonio, Santa Isabel, Vllahermosa, Villarrica

##### Notes

(v; au XC421234, ML98601161; ca; co CZUTOR 080; ph)

#### Catharus aurantiirostris

(Hartlaub, 1850)

75CFE1BE-39E2-5777-92A5-4B3E4E567147

##### Distribution

Cajamarca, Casabianca, Fresno, Ibagué, Icononzo, Libano, San Antonio

##### Notes

(v; au XC423639; ca; ph ML96501551)

#### Catharus fuscater

(Lafresnaye, 1845)

D1921308-AF7B-53D0-8EC7-B7292D2D37A6

##### Distribution

Cajamarca, Falan, Fresno, Ibagué, Libano, Santa Isabel

##### Notes

(v; ca; co CZUTOR 0468)

#### Catharus fuscescens

(Stephens, 1817)

44179E90-4AF0-57D4-9937-7E47A535CB3C

##### Distribution

Ibagué

##### Notes

(v; co) M

#### Catharus minimus

(Lafresnaye, 1848)

B36CCCAD-EFD5-57E9-9709-11B0F764D352

##### Distribution

Anzoategui, Falan, Ibagué, Planadas

##### Notes

(v; ca; ph ML82022581) M

#### Catharus ustulatus

(Nuttall, 1840)

27D3798B-FA0F-59AD-AF24-3FC420B9DE28

##### Distribution

Anzoátegui, Alvarado, Armero-Guayabal, Ataco, Casabianca, Chaparral, Cajamarca, Carmen de Apicala, Coyaima, Cunday, Dolores, Falan, Herveo, Honda, Ibagué, Lerida, Libano, Mariquita, Melgar, Planadas, Prado, Rio Blanco, San Antonio, San Luis, Venadillo, Villarrica

##### Notes

(v; au XC376479; co CZUT OR 0314; ph ML72672881) M WD

#### Turdus leucops

(Taczanowski, 1877)

89808F3E-71BD-58C6-8A1E-E9688C39B675

##### Distribution

Herrera, Ibagué, Libano

##### Notes

(v; ca; co CZUT-Or 523; ph)

#### Turdus leucomelas

Vieillot, 1818

39AFC99D-2C7C-5DA5-B809-FD930B7D2CDF

##### Distribution

Armero-Guayabal, Alvarado, Cajamarca, Chaparral, Chicoral, Cunday, Guamo, Espinal, Falan, Fresno, Honda, Ibagué, Icononzo, Lerida, Mariquita, Melgar, Planadas, Prado, Purificación, Rio Blanco, San Antonio, San Luis, Valle de San Juan, Venadillo, Villarrica

##### Notes

(v; au XC424618; ca; co MLS 5585, CZUTOR 231; ph ML123824851)

#### Turdus ignobilis

Sclater, 1858

6683FCEA-6A0E-55E5-8291-C5E42567B994

##### Distribution

Armero-Guayabal, Alvarado, Anzoátegui, Ataco, Cajamarca, Casabianca, Carmen de Apicala, Chaparral, Coyaima, Cunday, Doima, Dolores, Herveo, Honda, Ibagué, Icononzo, Falan, Fresno, Lerida, Libano, Natagaima, Mariquita, Melgar, Natagaima, Ortega, Palocabildo, Planadas, Prado, Purificación, Rio Blanco, Rovira, San Antonio, San Luis, Saldaña, Valle de San Juan, Suarez, Venadillo, Villarrica

##### Notes

(v; au XC420238, ML82139; ca co MLS 7988, CZUTOR 67; ph ML72672871)

#### Turdus fuscater

d'Orbigny & Lafresnaye, 1837

914157EE-22B7-5B86-AD1A-B6DFDDAC515B

##### Distribution

Anzoátegui, Casabianca, Cajamarca, Chaparral, Falan, Fresno, Herveo, Ibagué, Libano, Murillo, Planadas, Roncesvalles, San Antonio, Santa Isabel, Villarrica, Villahermosa

##### Notes

(v; au; ca; co SIB 126400, CZUTOR 95; ph ML58185661)

#### Turdus serranus

Tschudi, 1844

B5B7CAE2-9D0F-5B9E-9377-AB9637992D58

##### Distribution

Anzoátegui, Cajamarca, Ibagué, Murillo, Planadas, Roncesvalles, San Antonio, Santa Isabel, Villahermosa, Villarrica

##### Notes

(v; co MLS 559, CZUTOR 43; ph)

#### Mimus gilvus

(Vieillot, 1808)

81B562E6-907A-5FA0-AE3A-9C5A041F57CF

##### Distribution

Ambalema, Ataco, Casabianca, Carmen de Apicala, Cajamarca, Chaparral, Cunday, Coyaima, Espinal, Falan, Fresno, Guamo, Herveo, Honda, Ibagué, Lerida, Libano, Mariquita, Melgar, Murillo, Palocabildo, Planadas, Prado, Rio Blanco, San Antonio, San Luis, Saldaña, Suárez, Venadillo, Villarrica

##### Notes

(v; au; co MLS 556, MHN-ICN 11186; ph ML72672141)

#### Lonchura malacca

(Linnaeus, 1766)

D23211C9-10D6-529B-B171-EE195EF0DCDC

##### Distribution

Alvarado, Ambalema, Armero-Guayabal, Chaparral, Coello, Doima, Espinal, Guamo, Falan, Fresno, Ibagué, Ortega, Purificación, San Luis

##### Notes

(v; au; CZUTOR 0457; ph ML63999191) INT. El Salado, Ibagué, Tolima coordinates 4°27'22"N; 75°8'4"W. Date 22 Feb 2007, at 950 m a.s.l. Collector: R. Parra y Moreno (ICN 36229). This species was registered for the first time in Tolima in May 2004 and has expanded its distribution throughout the Department, especially in dry forest (below 1000 m a.s.l.), covering the lower area of the Department from Natagaima to the south to the Municipality of Guayabal to the north. It is usually observed as groups of up to hundreds of individuals flying over areas of rice crops and rarely areas of fruit trees, above 1400 m a.s.l. (Fig. 13b).

#### Lonchura atricapilla

(Vieillot, 1807)

4A9188AE-A80A-55E1-BC41-DB357D5AACF0

##### Distribution

Ibagué

##### Notes

(ph ML63695581) INT. Apparently non-established population. New record - this species was registered for the first time in February 2015 in the Municipality of Ibagué in the lagoon of the airport. In 2013, it was photographed in the Lagoon of Llanitos del Combeima and again on 20 November 2019 in the Jardín Botánico San Jorge, towards the dry forest (below 1300 m a.s.l.) (Fig. 13c).

#### Spinus spinescens

(Bonaparte, 1850)

EE9C830C-A4C9-5308-B6A8-559A31C22360

##### Distribution

Anzoátegui, Cajamarca, Falan, Herveo, Ibagué, Libano, Murillo, Roncesvalles, Santa Isabel

##### Notes

(v; co ISB 126380; ph ML32219391)

#### Spinus magellanicus

(Vieillot, 1805)

CAFEDFD4-32FC-559E-8CF2-71A924449D1D

##### Distribution

Cajamarca, Herveo, Ibagué, Murillo, Roncesvalles, Villarrica

##### Notes

(v; co SIB A6786)

#### Spinus xanthogastrus

(Du Bus, 1855)

8150CEEC-B02A-52E4-9518-4B33B72226A3

##### Distribution

Anzoategui, Casabianca, Herrera, Herveo, Fresno, Ibagué, Palocabildo, Libano, Murillo, Roncesvalles, San Antonio, Santa Isabel

##### Notes

(v; au XC421110; ph ML72672421)

#### Spinus psaltria

(Say, 1822)

692A68F8-44CA-5A41-951E-5850061F46F4

##### Distribution

Alpujarra, Alvarado, Ambalema, Anzoategui, Armero-Guayabal, Ataco, Casabianca, Chaparral, Carmen de Apicala, Chaparral, Coyaima, Cunday, Doima, Espinal, Falan, Flandes, Fresno, Guamo, Gualanday, Honda, Ibagué, Lerida, Libano, Mariquita, Palocabildo, Planadas, Purificación, Rio Blanco, Roncesvalles, San Antonio, San Luis, Suárez, Venadillo, Villarrica

##### Notes

(v; au; ca; co MLS 7449, CZUTOR 316; ph ML123872231)

#### Chlorophonia cyanocephala

(Vieillot, 1819)

8E2CCBEE-8686-5B7A-9687-B582B2F7EB70

##### Distribution

Alpujarra, Anzoátegui, Cajamarca, Casabianca, Chaparral, Falan, Fresno, Herveo, Ibagué, Libano, Murillo, Planadas, Roncesvalles, San Antonio, Villahermosa

##### Notes

(v; au; ca; co MHN-ICN 8592; ph ML82939221)

#### Chlorophonia cyanea

(Tunberg, 1822)

1B09D9F6-FB9B-56B9-B0B2-0CAE08988EBF

##### Distribution

Anzoategui, Cajamarca, Herveo, Falan, Ibagué, Libano, San Antonio, Villahermosa

##### Notes

(v; ca; co ANSP 154475; ph ML98663231)

#### Chlorophonia pyrrhophrys

(Scalter, 1851)

98D13630-EA7E-53C3-BE8F-6EA8B230055C

##### Distribution

Ibagué, Libano, Santa Isabel

##### Notes

(v; au-XC424643; co ANSP154477)

#### Euphonia concinna

Sclater, 1855

E09E5EAB-DAA8-59DC-A770-F52D13DFE0D2

##### Distribution

Alpujarra, Alvarado, Ambalema, Armero-Guayabal, Ataco, Carmen de Apicala, Coyaima, Cunday, Doima, Espinal, Falan, Flandes, Fresno, Guamo, Honda, Ibagué, Lérida, Mariquita, Melgar, Palocabildo, Planadas, Prado, Purificación, Rio Blanco, San Antonio, San Luis, Prado, Purificación, Suárez, Venadillo, Villarrica

##### Notes

(v; au-XC424397; ca; co MLS 6517¸ CZUTOR 190; ph ML111645851) E. This endemic species is found in the lower areas of the Department below 1400 m a.s.l. It has been observed since 2003 in forest relics and in areas with scattered trees, especially in tropical dry forest (Fig. 12d).

#### Euphonia laniirostris

d'Orbigny y Lafresnaye, 1837

228708CE-00E7-5743-AA21-364A13C6B470

##### Distribution

Alpujarra, Alvarado, Armero-Guayabal, Ambalema, Ataco, Carmen de Apicala, Cajamarca, Casabianca, Carmen de Apicala, Chaparral, Chicoral, Coyaima, Cunday, Dolores, Espinal, Falan, Fresno, Herveo, Honda, Ibagué, Icononzo, Lerida, Natagaima, Mariquita, Melgar, Murillo, Ortega, Palocabildo, Planadas, Prado, Rio Blanco, Roncesvalles, San Antonio, San Luis, Suárez, Saldaña, Río Blanco, Venadillo, Villahermosa, Villarrica

##### Notes

(v; au ML81691; ca co MLS 8281, CZUTOR 13 ph ML123871861 WD

#### Euphonia fulvicrissa

Sclater, 1857

3C86B9D3-75AB-5630-8CDF-8ACAFC980B1E

##### Distribution

Castilla (Purificación). Ibagué, One reg. in Armero

##### Notes

(co FMNH 36058)

#### Euphonia xanthogaster

Sundevall, 1834

EC53D8C8-6BE3-50F3-A194-9B795EB64AF2

##### Distribution

Casabianca, Chaparral, Herveo, Ibagué, Falan, Fresno, Guamo, Herveo, Libano, Melgar, Planadas, San Antonio, Villahermosa, Villarrica

##### Notes

(v; au; ca; co CZUTOR 713, MLS 2795)

#### Rhodinocichla rosea

(Lesson, 1832)

B07C3C1D-9C44-5A7B-BFD3-9F8B7FDBB81E

##### Distribution

Alpujarra, Anzoategui, Casabianca, Chaparral, Ibagué, Icononzo, Falan, Fresno, Libano, Melgar, Palocabildo, Planadas, Santa Isabel, Villahermosa, Villarrica

##### Notes

(v; au ML90030241; ca; co CZUTOR 0901; ph ML115255921)

#### Chlorospingus flavigularis

(Sclater, 1852)

BA345300-2FE4-5188-8575-49D5D7EC7ED5

##### Distribution

Ibagué, San Antonio

##### Notes

(v)

#### Chlorospingus parvirostris

Chapman, 1901

0376EDB1-1F6E-576E-B833-E4F08B8723BC

##### Distribution

Chaparral, Rio Blanco

##### Notes

(co CZUTOR 0976)

#### Chlorospingus canigularis

(Lafresnaye, 1848)

A15D6681-9E62-5968-BA15-AE6DD9227298

##### Distribution

Ambalema, Cajamarca, Falan, Ibagué, Libano, Santa Isabel

##### Notes

(v)

#### Chlorospingus flavopectus

(Lafresnaye, 1840)

D487D774-2F37-59DC-8CED-E5853A5E6A4A

##### Distribution

Casabianca, Cajamarca, Falan, Herveo, Ibagué, Libano, Murillo, Planadas, Roncesvalles, Santa Isabel, Villarrica

##### Notes

(v; ca; co CZUT 0077; ph ML123911991)

#### Ammodramus humeralis

(Bosc, 1792)

66B8FBEC-2A1F-510D-9C42-7A5F5FB8D712

##### Distribution

Armero-Guayabal, Alvarado, Chaparral, Chicoral, Coello, Doima, Espinal, Falan, Honda, Ibagué, Mariquita, Melgar, San Antonio, San Luis, Venadillo

##### Notes

(v; au ML97062071; ca co AMHN 112760; ph ML58318971)

#### Arremonops conirostris

(Bonaparte, 1850)

AFAB57EA-11D5-5BFE-915D-A136B3218BEE

##### Distribution

Armero-Guayabal, Alvarado, Ambalema, Ataco, Carmen de Apicala, Chaparral, Chicoral, Carmen de Apicala, Cunday, Doima, Dolores, Espinal, Gualanday, Falan, Fresno, Honda, Ibagué, Icononzo, Lerida, Mariquita, Melgar, Ortega, Palocabildo, Rio Blanco, Suárez, Venadillo, Villarrica

##### Notes

(v; au XC424612; CZUTOR co 0755; ph ML123885151)

#### Arremon atricapillus

(Lawrence, 1874)

40505EA8-DB30-5D57-8DB2-BD7EDA79DE5B

##### Distribution

Venadillo, Ibagué, Fresno, Libano, Murillo

##### Notes

(v; au ML58324001; ph ML123812121)

#### Arremon assimilis

(Boissonneau, 1840)

1900E3A4-CC74-5732-B509-A443B7AFF609

##### Distribution

Anzoátegui, Casabianca, Cunday, Herveo, Honda, Ibagué, Libano, Planadas, Roncesvalles, Santa Isabel, Villarrica

##### Notes

(v; au; ca; co SIB 179560, CZUTOR 529; ph)

#### Arremon aurantiirostris

Lafresnaye, 1847

8A3BB950-7FB7-532B-AA7E-F8EE2C1091A6

##### Distribution

Anzoategui, Alvarado, Armero-Guayabal, Casabianca, Chaparral, Cunday, Doima, Dolores, Espinal, Guamo, Falan, Fresno, Honda, Ibagué, Icononzo, Lerida, Libano, Mariquita, Melgar, Palocabildo, Purificación, Prado, Rio Blanco, Aan Antonio, San Luis, Valle de San Juan, Venadillo, Villarrica

##### Notes

(v; au XC424478; ca; co MLS 7622, CZUTOR 271; ph ML123885181)

#### Arremon brunneinucha

(Lafresnaye, 1839)

36B087B7-D973-58B3-BB2A-EA71CDAE4933

##### Distribution

Anzoátegui, Casabianca, Cajamarca, Ibagué, Falan, Fresno, Libano, Melgar, Murillo, Palocabildo, Planadas, Roncesvalles, San Antonio, Santa Isabel, Villahermosa, Villarrica

##### Notes

(v; au ML97316171; co CZUTOR 0466; ph ML97065891)

#### Zonotrichia capensis

(Statius Muller, 1776)

E8145510-9210-5052-9291-C7037579CE5A

##### Distribution

Anzoátegui, Ataco, Casabianca, Cajamarca, Chaparral, Cunday, Dolores, Falan, Fresno, Herveo, Ibagué, Icononzo, Libano, Melgar, Murillo, Planadas, Prado, Santa Isabel, Rio Blanco, Roncesvalles, San Antonio, Villarrica, Villahermosa, Villarrica

##### Notes

(v; au XC424588; ca; co SIB 126285, CZUTOR 34; ph ML123902591) WD

#### Atlapetes albinucha

(Lafresnaye & d’Orbigny, 1838)

7FEE1894-ABFC-52FF-AFB4-C46CCD3F4C1B

##### Distribution

Anzoátegui, Casabianca, Cajamarca, Falan, Fresno, Herveo, Ibagué, Icononzo, Libano, Murillo, Planadas, Roncesvalle, San Antonio, Santa Isabel, Venadillo, Villahermosa, Villarrica

##### Notes

(v; au XC376474; co CZUTOR 0562; ph ML71012331)

#### Atlapetes flaviceps

Chapman, 1912

75886A0C-E0D5-560C-9732-3F6C8EDC90FE

##### Distribution

Anzoátegui, Cajamarca, Casabianca, Falan, Fresno, Herveo, Ibagué, Libano, Murillo, Planadas, Roncesvalles, San Antonio, Santa Isabel

##### Notes

(v; au XC424583; ca; co CZUTOR 053; ph ML79639381) E EN. This endemic bird is usually registered in pairs or groups of four individuals, rarely forming mixed flocks. It has been registered in Tolima from 1500 to 2450 m a.s.l. in most municipalities with mountains (Fig. 11e).

#### Atlapetes fuscoolivaceus

Chapman, 1914

54255693-846D-5A65-AB69-67888B47EAAD

##### Distribution

Alpujarra, Planadas, Roncesvalles, San Antonio

##### Notes

(v; au ML99154431; ph ML97066601) New record - this endemic bird was registered only in the southern area of the Department, in the Municipalities of Alpujarra (pers. commun., J. Sanabria in 2016) and Planadas. It was recently photographed in the latter region on 22 April 2018 (Fig. 11f).

#### Atlapetes schistaceus

(Boissonneau, 1840)

23EC8F8E-B7A2-5D0F-9AC1-BE486E1B07E3

##### Distribution

Anzoátegui, Cajamarca, Chaparral, Dolores, Falan, Herveo, Ibagué, Icononzo, Murillo, Planadas, Roncesvalles, Santa Isabel, Villarrica

##### Notes

(v; au; co SIB 126540, CZUTOR 494; ph ML115212561)

#### Atlapetes pallidinucha

(Boissonneau, 1840)

B166B25B-35F5-5960-A01B-3D4B7BF0745F

##### Distribution

Anzoátegui, Cajamarca, Dolores, Herveo, Ibagué, Murillo, Planadas, Roncesvalles, Santa Isabel, Villahermosa

##### Notes

(v; co SIB 126694, CZUTOR 743; ph ML87226421)

#### Atlapetes latinuchus

(Du Bus, 1855)

A46763A5-0769-58C2-BA13-55AD9AB40C73

##### Distribution

Ibagué, Murillo

##### Notes

(v)

#### Sturnella magna

(Linnaeus, 1758)

DD5ED484-AD53-5505-90E9-1F0B90B7A61C

##### Distribution

Anzoátegui, Casabianca, Herveo, Murillo, Santa Isabel, Villahermosa, Villarrica

##### Notes

(v, au; ph ML83539091) This bird is commonly observed in the high areas of northern Tolima at elevations above 1800 m and up to 3700 m a.s.l. It has been observed in the Municipalities of Anzoátegui, Santa Isabel, Líbano, Herveo and Murillo where it is commonly seen vocalising on fences in deforested areas, such as pastures and open areas. It was registered for the first time in the Department by Quevedo and Lopéz (2002).

#### Leistes militaris

(Linnaeus, 1771)

408219A1-DBCB-5C48-8EB0-607849919BB0

##### Distribution

Alvarado, Ambalema, Armero-Guayabal, Carmen de Apicala, Chaparral, Cunday, Espinal, Falan, Honda, Ibagué, Lerida, Mariquita, Melgar, Natagaima, Planadas, Prado, Saldaña, Suárez, Valle de San Juan, Villarrica

##### Notes

(v; au; co MLS 8730, MHN-ICN 8214; ph ML111645961)

#### Amblycercus holosericeus

(Deppe, 1830)

7013EE40-069E-53CF-A065-1D1EBDAC3BC5

##### Distribution

Cajamarca, Ibagué, Herveo

##### Notes

(v; co ANSP 154725)

#### Psarocolius angustifrons

(Spix, 1824)

0E78CF63-1CB4-518C-85B7-FA5761721BE8

##### Distribution

Ataco, Ibagué, Honda, Planadas, San Antonio, Villarrica

##### Notes

(v; au; co MHN-ICN 10558; ph-ML97064061)

#### Psarocolius wagleri

(Gray, 1845)

034F85DD-AA65-5171-9DAF-25207D1E22C7

##### Distribution

Ataco, Rio Blanco, Saldaña

##### Notes

(v)

#### Psarocolius decumanus

(Pallas, 1769)

E7D32163-F479-5F04-8DA2-98C9B10EBAF6

##### Distribution

a, Ataco, Carmen de Apicala, Cunday, Honda, Ibagué, Libano, Melgar, Planadas, Prado, Rio Blanco, San Antonio, Suárez, Villarrica

##### Notes

(v; au XC424590; co ph ML123882261)

#### Cacicus uropygialis

Lafresnaye, 1843

D0A371E4-A28C-5BAB-AAA0-190A5EE911D8

##### Distribution

Dolores, Ibague, Planadas, Roncesvalles, San Antonio

##### Notes

(v; au ML99154311)

#### Cacicus cela

(Linnaeus, 1758)

FBC1E707-1D9C-556F-9C43-0C74FD0B33A0

##### Distribution

Espinal, Ibagué, Prado, San Antonio, Villarrica, Villahermosa

##### Notes

(v; co MLS 3115; ph) EL

#### Cacicus chrysonotus

(Bonaparte, 1838)

5597E1FC-AA08-58C1-B0BC-66A7998715E0

##### Distribution

Cajamarca, Herveo, Ibagué, Libano, Murillo, Prado, Roncesvalles, Santa Isabel, Villahermosa, Villarrica

##### Notes

(v; au ML28985441; AMHN 113190; ph ML78850951 v)

#### Icterus icterus

(Linnaeus, 1766)

9172CFCD-818A-5CA8-A968-DD6AF8A65F50

##### Distribution

Ibagué, Fresno

##### Notes

(v; ph ML20580021) M. New record - this species was introduced to the Department through the release of individuals in the San Jorge Botanical Garden. Some recent photographic records were made by H. Arias (Oyuela et al. 2016). However, the establishment of the species remains unknown.

#### Icterus mesomelas

(Wagler, 1829)

81910458-21FC-5CD7-AF1C-862251EE2388

##### Distribution

Armero-Guayabal, Carmen de Apicala, Chaparral, Falan, Libano, Prado, Purificación, San Antonio, San Luis

##### Notes

(v; ph) A photographic record at the border with caldas in Manzanares (ML 98958131).

#### Icterus auricapillus

Cassin, 1848

44D3422F-06A7-59EC-BC59-7842E0491EB3

##### Distribution

Alpujarra, Alvarado, Ataco, Carmen de Apicala, Chaparral, Cunday, Doima, Espinal, Falan, Fresno, Honda, Ibagué, Lerida, Libano, Mariquita, Melgar, Planadas, Prado, San Antonio, San Luis, Santa Isabel, Rio Blanco, Venadillo

##### Notes

(v; au; ca; co CZUTOR 0243 ph)

#### Icterus chrysater

(Lesson, 1844)

142FD2C5-F19D-523D-AD22-9306DF554D0F

##### Distribution

Armero-Guayabal, Anzoátegui, Ataco, Cajamarca, Casabianca, Chaparral, Cunday, Dolores, Falan, Fresno, Herveo, Ibagué, Libano, Murillo, Planadas, Prado, Rio Blanco, Roncesvalles, San Antonio, Santa isabel, Villarrica

##### Notes

(v; au; ca; co CZUTOR 0646)

#### Icterus galbula

(Linnaeus, 1758)

82EB3578-0A2F-5C62-ACB1-1CC1E1D89786

##### Distribution

Alvarado, Ambalema, Armero-Guayabal, Cajamarca, Doima, Falan, Fresno, Ibagué, Libano, Palocabildo, Piedras, San Antonio, Santa Isabel

##### Notes

(v; ph ML 123872681, ML 183462121) M

#### Icterus nigrogularis

(Hahn, 1819)

A10E8C1E-0E7E-5FA4-9306-7EDFA62E17AA

##### Distribution

Armero-Guayabal, Alvarado, Ambalema, Casabianca, Coyaima, Doima, Espinal, Falan, Fresno, Hinda, Ibagué, Mariquita, Melgar, Natagaima, Lerida, Libano, Palocabildo, Planadas, Prado, Purificación, San Luis, Saldaña, Suárez, Valle de San Juan, Venadillo

##### Notes

(v; au; ca; co CZUTOR 0204; ph ML104430081)

#### Molothrus oryzivorus

(Gmelin, 1788)

6A96363D-FC34-5F63-AC8B-FFCA539B8C21

##### Distribution

Ambalema, Cajamarca, Fresno, Honda, Ibagué, Libano, Murillo, Planadas, Piedras, Rovira, San Antonio, Santa Isabel, Suárez, Venadillo

##### Notes

(v; co ANSP 154724; ph ML108866981) This species has been occasionally observed between 1800 and 2000 m a.s.l. in the north-western hills and in the Juntas sector, where it is usually seen in groups of up to eight individuals moving in the middle and canopy of scattered forests and trees.

#### Molothrus bonariensis

(Gmelin, 1789)

83B30710-69F3-5FA8-B35C-2E1383E5CDD4

##### Distribution

Alpujarra, Alvarado, Anzoategui, Armero-Guayabal, Ataco, Casabianca, Cajamarca, Chaparral, Chicoral, Cunday, Doima, Espinal, Falan, Fresno, Guamo, Herveo, Honda, Libano, Natagaima, Mariquita, Melgar, Murillo, Venadilloo, Ibagué, Palocabildo, Planadas, Prado, Purificación, San Luis, Aanta Isabel, Saldaña, Suárez, Venadillo

##### Notes

(v; co MHN-ICN 10348; ph ML107832711)

#### Quiscalus lugubris

Swainson, 1838

9EAA50E6-A25B-53DF-B4E6-7E7AB8C1C368

##### Distribution

Ambalema, Armero-Guayabal, Casabianca, Chaparral, Coyaima, Doima, Ibagué, Lerida, Libano, Herveo, Honda, Espinal, Mariquita, Melgar, Guamo, Falan, Fresno, San Luis, Fresno, Murillo, Icononzo, Natagaima, Ortega, Palocabildo, Plandas, Santa Isabel, Valle de San Juan, Venadillo, Villarrica

##### Notes

(v; au; ph ML123872441). New record - this species has greatly expanded its distribution throughout the Department over the last 12 years, expanding to 3000 m a.s.l. It usually colonises parks and peri-urban areas where it congregates in groups of up to 80 individuals, forming colonies. It has sometimes been observed to displace native species (Fig. 12c).

#### Quiscalus mexicanus

(Gmelin, 1788)

C149DEEE-C1B9-5791-8E25-EB8089736BDD

##### Distribution

Armero-Guayabal, Doima, Fresno, Ibagué, Guamo, Lerida

##### Notes

(v; au; ph ML54189111) New record - this bird was recently registered in the Department on 11 April 2017 in Espinal, perhaps due to wandering or as a form of extension of its territory (Fig. 12a).

#### Hypopyrrhus pyrohypogaster

(Tarragon, 1847)

AD66BADB-4A14-531E-AA61-4283017C6284

##### Distribution

Ataco, Ibagué, San Antonio

##### Notes

(v; co MHN-ICN 12414, ph ML 231125651) E EN

#### Gymnomystax mexicanus

(Linnaeus, 1766)

1CE6D6ED-5D2A-5364-AD46-0B85217B39D1

##### Distribution

Ibagué, Melgar

##### Notes

(v) F. A group of 3 individuals seen in Girardot. Stiles registered some individuals in Melgar as a result of escapes from captivity.

#### Chrysomus icterocephalus

(Linnaeus, 1766)

28640337-1021-596A-9152-4E3259B35537

##### Distribution

Armero-Guayabal, Ambalema, Alvarado, Carmen de Apicala, Coyaima, Doima, Falan, Espinal, Guamo, Honda, Ibagué, Libano, Natagaima, Mariquita, Melgar, Prado, Purificación, Saldaña, San Luis, Venadillo, Villarrica

##### Notes

(v; au XC386427; ca; co MLS 6393, CZUTOR 790; ph ML104430071)

#### Parkesia noveboracensis

(Gmelin, 1789)

D890E77F-5934-5F72-80EB-239C67D71BBE

##### Distribution

Ambalema, Alvarado, Chaparral, Coello, Doima, Espinal, Fresno, Herrera, Honda, Ibagué, Mariquita, Prado, San Luis, Venadillo

##### Notes

(v; ca; co CZUTOR 0814; ph) M

#### Vermivora chrysoptera

(Linnaeu, 1766)

B45F322F-7C8C-58D3-9FF9-898762B69B8B

##### Distribution

Cajamarca, Ibagué, Libano

##### Notes

(v; ca; ph) NT M

#### Mniotilta varia

(Linnaeus, 1766)

D0201DAC-7DD7-5B26-844E-36D63323300A

##### Distribution

Anzoátegui, Cajamarca, Casabianca, Doima, Falan, Fresno, Honda, Ibagué, Libano, Mariquita, Planadas, Roncesvalles, Santa Isabel, San Luis, Villahermosa, Villarrica

##### Notes

(v; co CZUTOR 869 ph ML123891411) M

#### Protonotaria citrea

(Boddaert, 1783)

D405AE5C-8AF3-5C08-89D5-80E02004EEF1

##### Distribution

Ambalema, Armero-Guayabal, Casabianca, Doima, Falan, Honda, Ibagué, Libano, Melgar

##### Notes

(v; co CZUTOR 0829) M

#### Leiothlypis peregrina

(Wilson, 1811)

25190332-8775-5F39-B006-57DB2B99A4C5

##### Distribution

Anzoátegui, Cajamarca, Doima, Falan, Fresno, Herveo, Ibagué, Melgar, Planadas, San Antonio, San Luis, Santa Isabel

##### Notes

(v; ph) M

#### Oporornis agilis

(Wilson, 1812)

49A0B48E-639E-5A16-AA6B-E13FF268F9AB

##### Distribution

Ambalema, Armero-Guayabal, Ibagué

##### Notes

(v; ca; co CZUTOR 0826; ph) M

#### Geothlypis aequinoctialis

(Gmelin, 1789)

685031FD-9FCA-5B49-A223-E9CCB615F4CF

##### Distribution

Ibagué, Gualanday, Natagaima, Rovira

##### Notes

(ca; ph) Old collection record at the border with the Department of Huila, near the Municipality of Natagaima (MVZ 120643).

#### Geothlypis philadelphia

(Wilson, 1810)

5FBE7C6C-3A9B-56AC-A879-0C8EFD5043E0

##### Distribution

Anzoategui, Coello, Falan, Fresno, Ibagué, Herveo, Icononzo, Honda, Libano, Melgar, Murillo, Planadas, San Luis, Villarrica

##### Notes

(co MLS 7991, CZUTOR 658; ph) M

#### Setophaga ruticilla

(Linnaeus, 1758)

FC77945E-AF50-5737-AC98-427CE307C494

##### Distribution

Casabianca, Doima, Espinal, Falan, Honda, Ibagué, Lerida, Libano, Mariquita, Palocabildo, Planadas

##### Notes

(v; ca; co AMHN 122634; ph ML105057001) M

#### Setophaga cerulea

(Wilson, 1810)

0CFFAA4E-608F-5B7D-94AB-6293769ED4B4

##### Distribution

Anzoategui, Casabianca, Falan, Fresno, Ibagué, Honda, Libano, Mariquita, Planadas, Villahermosa

##### Notes

(v; ca; ph ML78622221) M VU

#### Setophaga pitiayumi

(Vieillot, 1817)

07EBB4EA-330E-5794-A609-658DDB0CF0D7

##### Distribution

Alpujarra, Anzoátegui, Cajamarca, Chaparral, Falan, Fresno, Herveo, Ibagué, Libano, Planadas, Prado, San Antonio, Santa Isabel, Roncesvalles, Villahermosa, Villarrica

##### Notes

(v; au; co CZUTOR 0910; ph ML123402031)

#### Setophaga castanea

(Wilson, 1810)

62E53994-CF3B-5370-A504-6B92D732B044

##### Distribution

Ambalema, Armero-Guayabal, Chaparral, Coello, Doima, Guamo, Falan, Honda, Ibagué, Lérida, Libano, Mariquita, San Luis, Suárez

##### Notes

(v; co ICN 5504; ph ML123876761) M

#### Setophaga fusca

(Statius Muller, 1776)

DE9BDD60-8A32-527D-9B1D-410CF24CCC66

##### Distribution

Alpujarra, Anzoátegui, Casabianca, Cajamarca, Chaparral, Cunday, Dolores, Falan, Fresno, Herveo, Herrear, Ibagué, Icononzo, Libano, Melgar, Murillo, Palocabildo, Planadas, Roncesvalles, San Antonio, Santa Isabel, Villarrica

##### Notes

(v; ca; co CZUT 0176; ph ML75625721) M

#### Setophaga petechia

(Linnaeus, 1766)

4F64987A-927D-500F-8524-9585362926CC

##### Distribution

Alvarado, Alpujarra, Ambalema, Ataco, Cajamarca, Casabianca, Chicoral, Cunday, Doima, Espinal, Gualanday, Guamo, Falan, Fresno, Herveo, Honda, Ibagué, Lerida, Mariquita, Natagaima, Palocabildo, Planadas, Prado, San Antonio, San Luis, Venadillo, Villarrica

##### Notes

(ca; co CZUTOR 0651; ph ML120505951) M

#### Setophaga pensylvanica

(Linnaeus, 1766)

2A5C1BB7-297E-5E6F-A256-A05F1FB87098

##### Distribution

Armero-Guayabal, Falan, Ibagué, Mariquita

##### Notes

(v) M

#### Setophaga striata

(Forster, 1772)

D3737CC8-10C4-5C71-A539-7351EDB6489B

##### Distribution

Armero-guayabal, Falan, Ibagué, Libano, Melgar, San Luis, Santa Isabel

##### Notes

(v; ca; co MLS 8148; ph) M

#### Myiothlypis luteoviridis

(Bonaparte, 1845)

65D8EC30-2901-52F1-97DF-9EFF4C4594EC

##### Distribution

Armero-Guayabal, Cajamarca, Herveo, Ibagué, Murillo, Roncesvalles

##### Notes

(v; ca; co MLS 8148; ph ML28985161)

#### Myiothlypis nigrocristata

(Lafresnaye, 1840)

6B07EF63-60DD-5C44-8F74-803A7477C775

##### Distribution

Cajamarca, Dolores, Herveo, Ibagué, Libano, Murillo, Roncesvalles, Santa Isabel, Villarrica

##### Notes

(v; au ML97306301; co SIB 126932, CZUT OR1292; ph ML30789631)

#### Myiothlypis fulvicauda

(Spix, 1825)

ADAAB98A-583D-55A5-AE8B-B19B63F95DCE

##### Distribution

Ambalema, Anzoategui, Alpujarra, Armero-Guayabal, Ataco, Chaparral, Carmen de Apicala, Casabianca, Coello, Coyaima, Cunday, Doima, Espinal, Guamo, Gualanday, Dolores, Espinal, Falan, Fresno, Herveo, Honda, Ibagué, Icononzo, Lerida, Mariquita, Melgar, Natagaima, Palocabildo, Planadas, Prado, Purificación, Rio Blanco, Rovira, San Antonio, San Luis, Suárez, Valle de San Juan, Venadillo, Villarrica

##### Notes

(v; au XC386649; co MLS 6191, CZUTOR 162; ph ML78852941) WD

#### Myiothlypis cinereicollis

(Sclater, 1864)

9E4F1378-CC1C-57FC-A3A6-F0C78F50BD19

##### Distribution

Icononzo

##### Notes

(ca; co CZUTOR 0889)

#### Myiothlypis coronata

(Tschudi, 1844)

1B33B28E-3BA3-5E37-80A4-26F7723E213B

##### Distribution

Anzoátegui, Cajamarca, Casabianca, Falan, Herveo, Ibagué, Libano, Murillo, Planadas, San Antonio, Santa Isabel, Roncesvalles, Villahermosa, Villarrica

##### Notes

(v; au XC96367; ca; co SIB 6699, CZUTOR 517; ph ML118162511)

#### Basileuterus deltattrii

(Swainson, 1838)

448A9A48-4E65-56A9-BE0C-0E0E27A85A55

##### Distribution

Alvarado, Ambalema, Ataco, Armero-Guayabal, Cajamarca, Carmen de Apicala, Casabianca, Chaparral, Chicoral, Cunday, Doima, Dolores, Espinal, Falan, Fresno, Gualanday, Guamo, Herveo, Honda, Ibagué, Icononzo, Lerida, Melgar, Murillo, Ortega, Palocabildo, Planadas, Prado, Rio Blanco, Rovira, Saldaña, San Antonio, San Luis, Suárez, Venadillo, Villahermosa, Villarrica

##### Notes

(v; au XC424472; ca; co MLS 6167, CZUTOR 066; ph ML123877111) WD

#### Basileuterus tristriatus

(Tschudi, 1844)

9A86B980-A530-5B7D-8334-F9D3ABD57B12

##### Distribution

Cajamarca, Chaparral, Herrera, Herveo, Ibagué, Libano, Murillo, Planadas, San Antonio, Santa Isabel, Rio Blanco, Roncesvalles, Villarrica

##### Notes

(v; au; co CZUTOR 0631; ph)

#### Cardellina canadensis

(Linnaeus, 1766)

3E8C3505-5824-5093-B5CC-F6341F2C42AC

##### Distribution

Anzoategui, Alvarado, Ambalema, Armero-Guayabal, Ataco, Casabianca, Cajamarca, Chaparral, Cunday, Doima, Espinal, Falan, Fresno, Ibagué, Herrera, Herveo, Honda, Icononzo, Libano, Mariquita, Melgar, Palocabildo, Planadas, Rovira, San Antonio, Santa Isabel, San Luis, Venadillo, Villahermosa, Villarica

##### Notes

(v; ca; co MLS 7965, CZUTOR 754; ph ML49802591) M

#### Myioborus miniatus

(Swainson, 1827)

09513024-CE35-5364-A0E4-7FABB9469AA5

##### Distribution

Anzoátegui, Casabianca, Cajamarca, Chaparral, Cunday, Dolores, Falan, Herveo, Ibagué, Icononzo, Libano, Murillo, Planadas, Prado, Rio Blanco, Roncesvalles, San Antonio, Santa Isabel, Villarrica, Villahermosa, Villarrica

##### Notes

(v; au XC423751, ML98601881; ca; co AMHN 112678, CZUTOR 381; ph ML98938771)

#### Myioborus ornatus

(Boissonneau, 1840)

679837B0-B38A-5DF8-80E8-7DCFD87F4633

##### Distribution

Anzoátegui, Cajamarca, Chaparral, Herveo, Ibagué, Libano, Murillo, Planadas, Roncesvalles, San Antonio, Santa Isabel, Villahermosa, Villarrica

##### Notes

(v; au ML25994911; co SIB 126818, CZUTOR 698; ph ML115221341) CE

#### Mitrospingus cassinii

(Lawrence, 1861)

2B0897F1-146B-5581-A739-A3A7F989FC9E

##### Distribution

Casabianca, Falan, Fresno, Mariquita

##### Notes

(v; ph ML 151085161) A photographic record by H. Arias.

#### Piranga flava

(Vieillot, 1822)

4005E7DD-8D43-55EC-9838-E08CFCE99542

##### Distribution

Anzoategui, Alpujarra, Falan, Fresno, Herveo, Ibagué, Libano, Murillo, Planadas, Rio Blanco, Roncesvalles, San Antonio

##### Notes

(v; co CZUTOR 0150; ph ML57899841)

#### Piranga rubra

(Linnaeus, 1758)

D9083D83-4108-5F63-BDFC-6C9457BDE070

##### Distribution

Alpujarrra, Ambalema, Armero-Guayabal, Anzoátegui, Ataco, Cajamarca, Carmen de Apicala, Casabianca, Chaparral, Cunday, Doima, Dolores, Falan, Guamo, Herrera, Herveo, Honda, Ibagué, Icononzo, Lerida, Mariquita, Melgar, Palocabildo, Planadas, Prado, Rio Blanco, Rovira, Saldaña, San Antonio, San Luis, Valle de San Juan, Venadillo, Villarrica

##### Notes

(v; au; ca; co MPUJ 803, CZUTOR 338; ph ML24063011) M

#### Piranga olivacea

(Gmelin, 1789)

AB5C9DF2-D4D5-5407-BF82-700EB9B03A64

##### Distribution

Armero-Guayabal, Alvarado, Cajamarca, Espinal, Falan, Fresno, Herrera, Herveo, Ibagué, Lerida, Planadas, Prado, Rovira, San Antonio, San Luis, Santa Isabel, Venadillo, Piedras

##### Notes

(v; ph ML26004701) M

#### Piranga rubriceps

Gray, 1844

CD0E4766-8E52-5401-9FDB-17F79C78C04F

##### Distribution

Cajamarca, Ibagué, Murillo, Planadas, Roncesvalles, San Antonio, San Luis

##### Notes

(v; co MHN-ICN 26877)

#### Piranga leucoptera

Trudeau, 1840

FDC005D1-8D1A-598C-AD8D-B768688D9381

##### Distribution

Falan, Ibagué, Planadas, Roncesvalles, Villarrica

##### Notes

(co ANSP 154568; ph ML97066551)

#### Driophlox gutturalis

(Sclater, 1854)

B6A29649-A5AF-5610-BA3C-F82505F7EF61

##### Distribution

Falan, Fresno, Honda, Mariquita

##### Notes

(v; co MPUJ 787; au ML123804151) E NT

#### Driophlox cristata

(Lawrence, 1875)

AD9456FD-0D75-5AAA-A423-7E6B80DCA65F

##### Distribution

Cajamarca, Chaparral, Falan, Fresno, Ibagué, Libano, Palocabildo, Planada, San Antonio, Rio Blanco

##### Notes

(v; au XC420538; co CZUTOR 1449; ph ML123927431) E

#### Pheucticus ludovicianus

(Linnaeus, 1766)

4A2E6ECC-738F-524C-B3C8-B8404F237EA6

##### Distribution

Ataco, Cajamarca, Casabianca, Chaparral, Cunday, Falan, Fresno, Herrera, Herveo, Ibagué, Libano, Mariquita, Melgar, Murillo, Palocabildo, Planadas, Santa Isabel, Roncesvalles, Villahermosa, Villarrica

##### Notes

(v; ca; co MHN-ICN 2831, CZUTOR 895; ph ML25603971) M

#### Cyanoloxia cyanoides

(Lafresnaye, 1847)

E7B86C49-ECFB-5E12-B567-DF48ECF0D923

##### Distribution

Anzoátegui, Ibagué, Falan, Fresno, Honda, Lerida, Libano, Mariquita, Prado, Rio Blanco, San Luis, Santa Isabel, Venadillo

##### Notes

(ca; co CZUTOR 858, AMHN 95107, ph)

#### Spiza americana

(Gmelin, 1789)

6BD0370E-01E3-54A5-A253-9548D3BFE316

##### Distribution

Alvarado, Armero-Guayabal, Doima, Espinal, Ibagué, Prado

##### Notes

(v; co MHN-ICN 7928; ph ML93079531) M

#### Sericossypha albocristata

(Lafresnaye, 1843)

28AD8828-1E56-5F3A-921D-2EFF0F77DF4C

##### Distribution

Anzoátegui, Cajamarca, Falan, Herveo, Ibagué, Libano, Murillo, Roncesvalles, San Antonio

##### Notes

(v; au; ph ML115221181)

#### Catamblyrhynchus diadema

Lafresnaye, 1842

44697359-9D10-523E-A795-F8EF71CD2637

##### Distribution

Cajamarca, Herveo, Ibagué, Libano, Murillo, Roncesvalles, Santa Isabel

##### Notes

(v; ca; co ANSP 154329, MHN-ICN 26192; ph ML75396161)

#### Chlorophanes spiza

(Linnaeus, 1758)

09460D10-322E-530C-BDBF-351D5813CDB8

##### Distribution

Alvarado, Armero-Guayabal, Cajamarca, Chaparral, Doima, Ibagué, Falan, Fresno, Herveo, Honda, Libano, Mariquita, Melgar, Palocabildo, Planadas, Rio Blanco, San Antonio, Venadillo, Villarrica

##### Notes

(co CZUTOR 0779; ph ML98660361)

#### Heterospingus xanthopygius

(Sclater, 1855)

589CAA3C-24FA-5115-B782-9F1E3426B096

##### Distribution

Cunday, Mariquita

##### Notes

(co MLS 7059)

#### Hemithraupis guira

(Linnaeus, 1766)

B0C84A8F-D623-5D7D-B220-AB90B6552892

##### Distribution

Cunday, Falan, Fresno, Ibagué, Honda, Lerida, Libano, Mariquita, Venadillo, San Antonio

##### Notes

(v; co MLS 783; ph ML26346071)

#### Hemithraupis flavicollis

(Vieillot, 1818)

20B8BB1D-460E-5F47-A902-3BE3C5FD4AB9

##### Distribution

Honda, Ibagué, Falan, Fresno, Lerida, Libano, Mariquita, Melgar, Prado

##### Notes

(v; ph ML105244441)

#### Conirostrum leucogenys

(Lafresnaye, 1852)

342CE9E0-6FED-50F7-9647-954F569135ED

##### Distribution

Alpujarra, Alvarado, Ambalema, Armero-Guayabal, Carmen de Apicala, Doima, Espinal, Falan, Guamo, Honda, Ibagué, Lerida, Mariquita, Natagaima, Piedras, Prado, San Luis, Venadillo

##### Notes

(v; co MHN-ICN 3280; ph ML123930841)

#### Conirostrum sitticolor

Lafresnay, 1840

D09E8C3E-80AD-5D39-971C-5EA3C382F82F

##### Distribution

Anzoátegui, Cajamarca, Herveo, Ibagué, Libano, Murillo, Roncesvalles, Santa Isabel

##### Notes

(v; ca; co SIB 128819)

#### Conirostrum albifrons

Lafresnaye, 1842

80F1B62E-A4A4-584C-A838-E5C74F064D8A

##### Distribution

Anzoátegui,Cajamarca, Herveo, Ibagué, Lerida, Libano, Murillo, Roncesvalles, Santa Isabel

##### Notes

(v; co CZUTOR 062; ph ML22275721)

#### Sicalis citrina

Pelzeln, 1870

2F693DCB-2B3B-5152-AA0C-20AABBBAB06A

##### Distribution

Murillo

##### Notes

(ph ML58021011)

#### Sicalis flaveola

(Linnaeus, 1766)

D1A44BE2-543D-5947-AE51-4550FC983349

##### Distribution

Alvarado, Armero-Guayabal, Ambalema, Ataco, Casabianca, Cajamarca, Chaparral, Coyaima, Cunday, Dolores, Espinal, Falan, Fresno, Guamo, Herveo, Honda, Ibagué, Lerida, Libano, Natagaima, Mariquita, Melgar, Murillo, Planadas, Prado, Purificación, Rio Blanco, Roncesvalles, Saldaña, San Antonio, San Luis, Suárez, Venadillo, Villarricallahermosa, Villarrica

##### Notes

(v; au XC424729; ca; co MPUJ 502, CZUTOR 007; ph ML107735511) WD

#### Sicalis luteola

(Sparrman, 1789)

CED86BED-E79E-554B-A6D2-4EDC74BBF4E6

##### Distribution

Alvarado. Armero-Guayabal, Doima, Espinal, Guamo, Ibagué, Mariquita, San Luis, Venadillo, Villarrica

##### Notes

(v; au; co MHN-ICN 13879; ph)

#### Geospizopsis unicolor

(d'Orbigny & Lafresnaye, 1837)

68D18E6F-9BD0-5791-A683-02E507842806

##### Distribution

Anzoátegui, Cajamarca, Herveo, Ibagué, Murillo, Rio Blanco, Roncesvalles, Santa Isabel

##### Notes

(v; ca; co; SIB A6723; ph ML123900801)

#### Catamenia analis

(d´Orbigny and Lafresnaye, 1837, 1837)

A4DCD56B-DA30-5852-9C74-FAF0D91096C6

##### Distribution

Anzoategui, Cajamarca, Herveo, Ibagué, Murillo, Santa Isabel

##### Notes

(v; co; ph ML106430791)

#### Catamenia inornata

(Lafresnaye, 1847)

962487FD-1F43-50BC-A140-CEC8E51394ED

##### Distribution

Anzoátegui, Cajamarca, Herveo, Ibagué, Murillo, Santa Isabel, Roncesvalles

##### Notes

(v; au XC96315; co CZUTOR 0227; ph ML123902801)

#### Catamenia homochroa

(Sclater, 1859)

D1774C9C-43B2-558E-887E-222048423C2A

##### Distribution

Anzoátegui, Cajamarca, Herveo, Ibagué, Murillo, Santa Isabel, Roncesvalles

##### Notes

(v; co MHN-ICN 26207; ph ML123903561)

#### Diglossa lafresnayii

(Boissonneau, 1840)

3333DF83-D51A-594F-B69D-C85CF9603BA8

##### Distribution

Anzoategui, Cajamarca, Herveo, Ibagué, Libano, Murillo, Planadas, Roncesvalles, Santa Isabel

##### Notes

(v; ca; co CZUTOR 052; ph ML51941521)

#### Diglossa humeralis

(Fraser, 1840)

1DA85518-A849-52FF-B591-362836D979F3

##### Distribution

Anzoátegui, Cajamarca, Falan, Fresno, Ibagué, Libano, Murillo, Planadas, San Antonio, Santa Isabel, Roncesvalles, Villahermosa

##### Notes

(v; au ML98941201; co ANSP 154161; CZUTOR 502 ph)

#### Diglossa brunneiventris

(Lafresnaye, 1846)

0B4EFD30-E4BA-5446-BAE1-38FABF5D02BB

##### Distribution

Herveo, Murillo

##### Notes

(v; ph ML 64388201). There are a few recent records for this species in Tolima in the páramo zone, including some records uploaded to the eBird platform since July 2017. A photographic record was made by A. Collerton in the subpáramo zone.

#### Diglossa albilatera

(Lafresnaye, 1843)

D2E4F759-CB66-5E8B-B228-CFE07EA2ABC9

##### Distribution

Anzoátegui, Cajamarca, Chaparral, Falan, Herveo, Ibagué, Libano, Murillo, Planadas, Roncesvalles, San Antonio, Santa Isabel, Villahermosa, Villarrica

##### Notes

(v; ca; co CZUT 0154; ph ML98497561)

#### Diglossa sittoides

(d'Orbigny & Lafresnaye, 1838)

0DF1CD65-9208-5BF3-9E5A-865B97915E01

##### Distribution

Anzoategui, Cajamarca, Falan, Fresno, Herveo, Ibagué, Libano, Murillo, Planadas, Villahermosa, Santa Isabel, Roncesvalles

##### Notes

(v; ca; co CZUTOR 0997; ph ML103292721)

#### Diglossa caerulescens

(Sclater, 1856)

90A06A04-2A9F-571F-8B5A-E11EDE51BAB7

##### Distribution

Cajamarca, Chaparral, Ibagué, Herveo, Murillo, San Antonio

##### Notes

(v; ca; co CZUTOR 0230; ph ML22279771)

#### Diglossa cyanea

(Lafresnaye, 1840)

951BC387-DD65-517C-B4DD-1E232A624C5F

##### Distribution

Anzoátegui, Cajamarca, Chaparral, Falan, Herveo, Ibagué, Murillo, Roncesvalles, Santa Isabel, San Antonio

##### Notes

(v; au; ML97307011; co CZUTOR 046; ph ML120522391)

#### Haplospiza rustica

(Tschudi, 1844)

9E3E4A79-E060-59DD-9207-C9B0DB440A11

##### Distribution

Casabianca, Falan, Herveo, Ibagué, Palocabildo, Planadas, Roncesvalles, Murillo

##### Notes

(v; ca; ph)

#### Volatinia jacarina

(Linnaeus, 1766)

04BFFFDE-67A1-5E30-8575-4FAB58A04385

##### Distribution

Alvarado, Anzoategui, Ambalema, Armero-Guayabal, Chaparral, Chicoral, Casabianca, Carmen de Apicala, Chicoral, Coyaima, Cunday, Dolores, Espinal, Falan, Fresno, Guamo, Gualanday, Honda, Ibagué, Lerida, Libano, Mariquita, Melgar, Prado, Rio Blanco, Saldaña, San Antonio, San Luis, Suárez, Venadillo, Villarrica

##### Notes

(v; au XC424492; ca; co MLS 8726, CZUTOR 264; ph ML123824971) WD

#### Creurgops verticalis

(Sclater, 1858)

12FA5FE1-C94B-5215-BE56-75D3FE8783B1

##### Distribution

Cajamarca, Chaparral, Falan, Ibagué, Libano, Murillo, Planadas, San Antonio, Santa Isabel, Roncesvalles

##### Notes

(v; ca; co; ph ML 233651461)

#### Loriotus luctuosus

d'Orbigny y Lafresnay, 1837

554BC98B-5D20-5E78-A211-DD69B4E81D8C

##### Distribution

Armero-Guayabal, Cunday, Dolores, Espinal, Falan, Fresno, Guamo, Gualanday, Herveo, Honda, Ibagué, Lerida, Mariquita, Melgar, Palocabildo, Prado, Rio Blanco, Villarrica

##### Notes

(v; ca; co MLS 7050, CZUTOR 127; ph ML24063521)

#### Tachyphonus delatrii

Lafresnaye, 1847

62A1474F-F69D-5A81-9878-F8CC49310890

##### Distribution

Mariquita, Falan, Honda

##### Notes

(v; co AMHN 95050; ph https://www.inaturalist.org/photos/40933554)

#### Tachyphonus rufus

(Boddaert, 1783)

069A45C1-D9AF-55A4-A6E5-B820DD4CE52A

##### Distribution

Amabalema, Alpujarra, Armero-Guayabal, Ataco, Casabianca, Cunday, Doima, Dolores, Falan, Fresno, Herrera, Herveo, Ibagué, Mariquita, Planadas, Prado, Rio Blanco, San Antonio, Santa Isabel, Villarrica

##### Notes

(v; co MHN-ICN 1863, CZUTOR 304; ph ML123932981)

#### Eucometis penicillata

(Spix, 1825)

61DCE128-07AD-5757-AD85-1416C990BE60

##### Distribution

Alvarado, Ambalema, Armero-Guayabal, Chaparral, Coyaima, Cunday, doima, Espinal, Ibagué, Icononzo, Falan, Fresno, Gualanday, Honda, Lerida, Mariquita, Melgar, Palocabildo, Planadas, Prado, San Antonio, San Luis, Suarez, Rio Blanco, Venadillo, Villahermosa, Villarrica

##### Notes

(v; au XC424603, ML82132; ca; co MLS 7066, CZUTOR 244; ph ML123829541)

#### Coryphospingus pileatus

(Wied-Neuwied, 1820)

FAADD8C5-1E85-5F6E-B038-65239F8943F3

##### Distribution

Ambalema, Armero-Guayabal, Alvarado, Coello, Coyaima, Doima, Espinal, Falan, Fresno, Gualanday, Guamo, Ibagué, Icononzo, Lerida, Natagaima, Melgar, San Luis, Venadillo

##### Notes

(v; ca; co MLS 7495, CZUTOR 50; ph ML123187281)

#### Ramphocelus dimidiatus

Lafresnaye, 1837

FD12E85A-17CD-545A-9BD5-E48B904FF579

##### Distribution

Armero-Guayabal, Alvarado,Ambalema, Ataco, Casabianca, Chaparral, Carmen de Apicala, Coello, Cunday, Dolores, Espinal, Falan, Fresno, Herveo, Honda, Guamo, Gualanday, Ibagué, Icononzo, Lerida, Mariquita, Melgar, Murillo, Natagaima, Palocabildo, Prado, Planadas, Purificación, Rio Blanco, Saldaña, San Antonio San Luis, Suárez, Venadillo, Villarrica

##### Notes

(v; au XC424701; co MLS 6956, CZUTOR 25; ca ph ML112143231) WD

#### Ramphocelus flammigerus

(Jardine & Selby, 1833)

31AA186D-E536-5973-969D-4BC201C036C2

##### Distribution

Ataco, Casabianca, Chaparral, Falan, Fresno, Herrera, Herveo, Honda, Ibagué, Lerida, Libano, Mariquita, Palocabildo, Planadas, San Antonio, Villahermosa, Villarrica

##### Notes

(v; ca; ph ML84503201; co ANSP 154506) CE

#### Cyanerpes caeruleus

(Linnaeus, 1758)

3991925E-3523-5949-B505-0B0FA731A298

##### Distribution

Honda

##### Notes

(v, co)

#### Tersina viridis

(Illiger, 1811)

021EF0AF-4B16-5EF6-943F-BD524C6D011C

##### Distribution

Ataco, Casabianca, Chaparral, Ibagué, Falan, Fresno, Honda, Mariquita, Palocabildo, Planadas, Prado, San Antonio, Santa Isabel, Venadillo

##### Notes

(v; co MLS 6436; ph)

#### Dacnis egregia

(Sclater, 1855)

5621C6A7-36F4-51DE-BC35-AE8B2CC107C4

##### Distribution

Alvarado, Anzoategui, Ambalema, Armero-Guayabal, Ataco, Cajamarca, Chaparral, Carmen de Apicala, Cunday, Doima, Dolores, Guamo, Honda, Ibagué, Falan, Fresno, Lerida, Mariquita, Melgar, Natagaima, Palocabildo, Planadas, Prado, Purificación, Rio Blanco, Rovira, San Antonio, San Luis, Valle de San Juan, Venadillo

##### Notes

(v; au XC424403; co MLS 5929, CZUTOR 99; ph ML99185961)

#### Dacnis hartlaubi

Sclater, 1855

01481198-0FC8-5840-BE9A-4DC90749E8BE

##### Distribution

Villahermosa

##### Notes

(v)

#### Dacnis cayana

(Linnaeus, 1766)

075156E1-4D23-5D61-9B92-2AEE0FA7550D

##### Distribution

Alpujarra, Alvarado, Armero-Guayabal, Ataco, Casabianca, Carmen de Apicala, Coyaima, Doima, Dolores, Falan, Fresno, Guamo, Ibagué, Lerida, Libano, Mariquita, Melgar, Palocabildo, Planadas, Prado, Purificación, Rovira, Suarez, Valle de San Juan, San Antonio, San Luis, Rio Blanco, Venadillo

##### Notes

(v; ca; co MLS 597, CZUTOR 0667; ph ML123898291)

#### Sporophila minuta

(Linnaeus, 1758)

C2B6DF49-32D8-5BFC-B96E-1EBA1F5EEEC7

##### Distribution

Alvarado, Armero-Guayabal, Ataco, Cajamarca, Chaparral, Carmen de Apicala, Coello, Cunday, Doima, Dolores, Espinal, Falan, Fresno, Gualanday, Guamo, Herveo, Honda, Ibagué, Lerida, Libano, Mariquita, Natagaima, Planadas, Prado, Rio Blanco, San Luis, Saldaña, Suarez, Venadillo, Villarrica

##### Notes

(v; au; co MLS 7377, CZUTOR 407; ph ML123826651)

#### Sporophila funerea

(Sclater, 1860)

6C993452-9CA1-5D62-820E-870E716A40F0

##### Distribution

Armero-Guayabal, Casabianca, Coyaima, Falan, Fresno, Honda, Ibagué, Icononzo, Lerida, Libano, Mariquita, Plandas, Prado

##### Notes

(v; co CZUT OR 1043; ph ML121462621) ICN. This species is observed most commonly below 1200 m a.s.l. It is seen more often than the congener *S. angolensis*, which was previously classified as the same species. It has been frequently registered in Falan and Mariquita and infrequently registered in Ibagué, Guayabal, Fresno and Líbano ().

#### Sporophila angolensis

(Linnaeus, 1766)

9F5C1416-DD0C-5CF8-96E6-49817B1DEE7A

##### Distribution

Armero-Guayabal, Coyaima, Doima, Ibagué, Mariquita, Melgar, Planadas, Prado, Rovira, Venadillo

##### Notes

(v; au; ca; co CZUTOR 0933; ph ML82117) This uncommon species in Tolima has been registered sporadically in Melgar and Ibagué between 500 and 1200 m a.s.l. (Fig. 11b). In the Municipality of Ibagué, it is found together with *S. funerea*, but not in sympatry.

#### Sporophila crassirostris

(Gmelin, 1789)

3127501F-5C00-5266-9AB4-D92778F67E1B

##### Distribution

Armero-Guayabal, Casabianca, Falan, Fresno, Ibagué, Herveo, Honda, Lerida, Melgar, Mariquita, Venadillo, Villarrica

##### Notes

(v; co MPUJ 509, CZUTOR 708; ph ML55266751). Like other seed finches, it is usually located in the canopy in relics of forest, semi-open areas and areas with crops where it vocalises frequently. It has been frequently observed in the Municipality of Ibagué and in the north of Tolima at about 1200 to 1400 m a.s.l. (Fig. 11d).

#### Sporophila intermedia

Cabanis, 1851

F74682F0-44E1-5525-8936-8152047EA047

##### Distribution

Anzoategui, Armero-Guayabal, Ataco, Alvarado, Chaparral, Coyaima, Doima, Espinal, Falan, Fresno, Honda, Lerida, Mariquita, Melgar, Natagaima, Ibagué, Icononzo, Planadas, Prado, Purificación, Saldaña, San Antonio, Suarez, Venadillo, Villarrica

##### Notes

(v; co MLS 7302; ph ML98989561)

#### Sporophila luctuosa

(Lafresnaye, 1843)

187B49B8-4D72-500C-B84D-D6C3F9DACE96

##### Distribution

Casabianca, Espinal, Falan, Fresno, Ibagué, Libano, Murillo, Palocabildo, Villarrica

##### Notes

(v; co MLS 7327, CZUTOR 639; ph ML99156451)

#### Sporophila nigricollis

(Vieillot, 1823)

23B32BC2-AC12-5EB4-9CFD-3E3341A2A42A

##### Distribution

Anzoategui, Ambalema, Alpujarra, Armero-Guayabal, Ataco, Casabianca, Carmen de Apicala, Chaparral, Coyaima, Cunday, Dolores, Espinal, Falan, Fresno, Guamo, Herveo, Honda, Ibagué, Lerida, Libano, Natagaima, Mariquita, Melgar, Murillo, Planadas, Purificación, Prado, Rio Blanco, San Antonio, Santa Isabel, Saldaña, San Luis, Suárez, Venadillo, Villarrica

##### Notes

(v; au XC424592; ca; co CZUTOR 0153; ph ML120513811)

#### Sporophila schistacea

(Lawrence, 1862)

AE34B4C4-1AD3-5A43-AD11-50B3A5EA2C82

##### Distribution

Cunday, Dolores, Espinal, Falan, Fresno, Honda, Ibagué, Icononzo, Libano, Planadas, Prado, Rio Blanco, San Antonio, Venadillo, Villarrica

##### Notes

(v; au XC424593; ca; co MPUJ 770, CZUTOR 431; ph)

#### Sporophila plumbea

(Wied-Neuwied, 1831)

457798E3-DFEB-5A38-A964-4BD79A8F27B4

##### Distribution

Doima, Ibagué, Melgar

##### Notes

(v; co MHN-ICN 8384)

#### Saltator maximus

(Statius Müller, 1776)

BB251AD6-F6A8-509F-B345-F5E5B12EF914

##### Distribution

Alvarado, Ambalema, Armero-Guayabal, Casabianca, Carmen de Apicala, Chaparral, Coyaima, Cunday, Doima, Dolores, Espinal, Falan, Fresno, Guamo, Honda, Ibagué, Lerida, Libano, Mariquita, Melgar, Palocabildo, Prado, Purificación, Rio Blanco, San Antonio, San Luis, Valle de San Juan, Suarez, Venadillo, Villarrica

##### Notes

(v; au XC423701; ca; co MPUJ 767, CZUTOR 139; ph ML104593371)

#### Saltator atripennis

Sclater, 1857

53C111B6-B901-5C6E-A69F-960B18AD7AF5

##### Distribution

Cjamarca, Casabianca, Chaparral, Cunday, Falan, Fresno, Herveo, Ibagué, Libano, Murillo, Rio Blanco, Roncesvalles, San Antonio, Santa Isabel, Villahermosa, Villarrica

##### Notes

(v; ca; au ML98604791; co CZUTOR 0714; ph ML72672701)

#### Saltator olivascens

Vieillot, 1817

F3200FFE-F624-50F5-B853-589D8601BC98

##### Distribution

Armero-Guayabal, Falan, Honda, Ibagué, Lerida, Libano, Mariquita, Rio Blanco, San Antonio, Suárez, Venadillo

##### Notes

(v; au XC423696; co CZUTOR 0458; ph ML79096071)

#### Saltator striatipectus

Lafresnaye, 1847

55923AC6-3153-50BF-BCD9-2E887C827625

##### Distribution

Armero-Guayabal, Ambalema, Casabianca, Chaparral, Chicoral, Coyaima, Cunday, Dolores, Espinal, Falan, Fresno, Gualanday, Guamo, Honda, Ibagué, Icononzo, Lerida, Libano, Mariquita, Melgar, Natagaima, Planadas, Prado, Rio Blanco, Roncesvalles, Saldaña, San Luis, San Antonio, Suárez, Venadillo, Villarrica

##### Notes

(v; au XC424605; co MLS 7223, CZUTOR 17; ph ML72672601) WD

#### Saltator cinctus

Zimmer, 1943

B8FB3D91-3E94-5C1C-BB65-0444F9678B65

##### Distribution

Anzoátegui, Herveo, Ibagué, Roncesvalles, Murillo

##### Notes

(v; au ML97304231; ph ML112810901) NT

#### Saltator grossus

(Linnaeus, 1766)

3DEAE644-B625-53D5-8AC3-A5525C8C6AB3

##### Distribution

Ibagué, Honda

##### Notes

(co AMHN 95117) *Pitylus* according to Hilty.

#### Emberizoides herbicola

(Vieillot, 1817)

4F06BC3E-AEB7-5598-9EB0-05696B53CFF8

##### Distribution

Falan, Ibagué, Roncesvalles, San Antonio

##### Notes

(v; ph ML36345521)

#### Pseudospingus verticalis

(Lafresnaye, 1840)

D45FE40B-1B4D-5546-9ACF-9ACB1D43486D

##### Distribution

Anzoátegui, Cajamarca, Ibagué, Libano, Murillo, Roncesvalles, Santa Isabel

##### Notes

(v; au; co CZUTOR 0507)

#### Cnemoscopus rubrirostris

(Lafresnaye, 1840)

C3D4CFCF-A132-5591-87E7-CA443A9AC88B

##### Distribution

Anzoátegui, Cajamarca, Herveo, Ibagué, Murillo, Roncesvalles, Santa Isabel

##### Notes

(v; au ML28985231; co ANSP 154499; ph ML35761591)

#### Kleinothraupis atropileus

(Lafresnaye, 1842)

60DF7A6C-8C2A-565E-B5FB-35A46805789E

##### Distribution

Cajamarca, Ibagué, Melgar, Murillo, Roncesvalles

##### Notes

(v; co CZUTOR 0078; ca; ph ML86905801)

#### Sphenopsis frontalis

(Tschudi, 1844)

91ABE946-D1B3-5203-A2AF-1DD846496ABD

##### Distribution

Cajamarca, Falan, Herveo, Ibagué, Libano, Roncesvalles, San Antonio, Santa Isabel, Villahermosa

##### Notes

(v; ca; co CZUTOR 0078; ph ML78851321)

#### Sphenopsis melanotis

(Sclater, 1855)

72221EF7-1487-5896-8FB4-8222D7F05786

##### Distribution

Casabianca, Falan, Herveo, Ibagué, Murillo, Roncesvalles, Santa Isabel

##### Notes

(v; co CZUTOR 1343; ph ML22279441)

#### Thlypopsis superciliaris

(Lafresnaye, 1840)

1862858E-C936-5669-8C16-09D89FC6B074

##### Distribution

Anzoátegui, Casabianca, Cajamarca, Cunday, Herveo, Ibagué, Libano, Murillo, Roncesvalles, Santa Isabel, Villarrica

##### Notes

(v; ca; co CZUTOR 134; ph ML30789701)

#### Urothraupis stolzmanni

Taczanowski & Berlepsh, 1885

08440219-958D-50C6-AC1A-6C760AC1EE4C

##### Distribution

Anzoátegui, Cajamarca, Herveo, Ibagué, Murillo, Roncesvalles, Santa Isabel

##### Notes

(v; ph ML115351201) CE

#### Coereba flaveola

(Linnaeus, 1758)

E424136D-5D06-5865-9141-37CD7C8B5F05

##### Distribution

Anzoategui, Armero-Guayabal, Ambalema, Alvarado, Ataco, Cajamarca, Carmen de Apicala, Casabianca, Chaparral, Coyaima, Cunday, Doima, Espinal, Falan, Fresno, Guamo, Herveo, Honda, Ibagué, Icononzo, Lerida, Libano, Mariquita, Murillo, Natagaima, Palocabildo, Planadas, Prado, Rio Blanco, San Antonio, Santa Isable, Saldaña, Suárez, Venadillo, Villahermosa, Villarrica

##### Notes

(v; au XC420337; ca; co CZUTOR 0007; ph ML99158481) WD

#### Tiaris olivaceus

(Linnaeus, 1766)

C8791CB6-27FD-5F51-8983-9388C2C2972B

##### Distribution

Armero-Guayabal, Anzoátegui, Casabianca, Chaparral, Cunday, Dolores, Falan, Fresno, Herveo, Honda, Ibagué, Lerida, Libano, Planadas, San Antonio, Rio Blanco, Villarrica

##### Notes

(v; au XC424595; ca; co CZUTOR 1080; ph ML72672731)

#### Asemospiza obscura

(d'Orbigny & Lafresnaye, 1837)

1DD3D96B-F1DB-578D-8DA2-8F47FEC13343

##### Distribution

Alvarado, Cajamarca, Doima, Dolores, Guamo, Herveo, Honda, Ibagué, Icononzo, Lerida, Planadas, Prado, Rio Blanco, Roncesvalles, Santa Isabel, Villarrica

##### Notes

(v; ca; co SIB 32349, CZUTOR 376; ph ML123929121)

#### Melanospiza bicolor

(Linnaeus, 1766)

135E31BF-91AB-5F3C-85A0-E493CF96475A

##### Distribution

Alvarado, Armero-Guayabal, Casabianca, Chaparral, Carmen de Apicala, Coyaima, Cunday, Doima, Dolores, Espinal, Guamo, Honda, Ibagué, Falan, Fresno, Lerida, Libano, Mariquita, Natagaima, Planadas, Prado, Rio Blanco, San Antonio, San Luis, Suárez, Valle de San Juan, Venadillo, Villarrica

##### Notes

(v; au XC424872; ca; co MLS 7283, CZUTOR 164; ph ML58280511) WD

#### Chlorochrysa nitidissima

(P. L. Sclater, 1874)

E87605D4-EC8E-54AB-A51C-BD35CB8FDC34

##### Distribution

Ibagué

##### Notes

(v; ph ML 309203161, au ML 278463171) This unusual species in the Department was observed for the first time on 13 and 17 October 2019 in the Reserva Forestal Protectora Regional Bellavista (Loaiza-Hernández et al. 2021). It was subsequently registered by H. Arias in the same area on 8 November 2020.

#### Schistochlamys melanopis

(Latham, 1790)

8B4D37B7-14F9-5772-9E4D-FF8EAC445C15

##### Distribution

Ambalema, Cajamarca, Carmen de Apicala, Casabianca, Chaparral, Coyaima, Espinal, Falan, Ibagué, Mariquita, Melgar, Planadas, San Luis, Rovira, Villarrica

##### Notes

(v; co MLS 7788, MHN-ICN 2256; ph ML72672311)

#### Iridosornis rufivertex

(Lafresnaye, 1842)

A6774AB7-172B-5FCB-A331-A8729DE852D8

##### Distribution

Anzoategui, Cajamarca, Herrera, Herveo, Ibagué, Murillo, Roncesvalles

##### Notes

(v; ca; co CZUTOR 051; ph ML115212771)

#### Pipraeidea melanonota

(Vieillot, 1819)

6654DB54-31A6-521E-B590-4393225495A4

##### Distribution

Anzoategui, Chaparral, Falan, Herrera, Herveo, Ibagué, Libano, Murillo, Planadas, Roncesvalles, San Antonio, Santa Isabel, Villahermosa, Villarrica

##### Notes

(v; au XC425943; ca; co CZUTOR 0012; ph ML98969251)

#### Dubusia taeniata

(Boussonneau, 1840)

09692B58-9368-5D51-9129-F9FC7126F6EF

##### Distribution

Anzoategui, Cajamarca, Herveo, Ibagué, Murillo, Roncesvalles

##### Notes

(v; co ANSP 154566; ph ML78999011)

#### Anisognathus lacrymosus

(Du Bus, 1847)

16DA3FBF-156D-510A-B60D-B45D419B4C1B

##### Distribution

Anzoátegui, Cajamarca, Chaparral, Falan, Herveo, Ibagué, Icononzo, Libano, Murillo, Planadas, Roncesvalles, Santa Isabel, Villahermosa

##### Notes

(v; au; co SIB 126378, CZUTOR 0497; ph ML23812291)

#### Anisognathus igniventris

(d'Orbigny & Lafresnaye, 1837)

64AE5E55-9F0F-59F3-A7CA-680444464091

##### Distribution

Anzoátegui, Cajamarca, Herveo, Ibagué, Murillo, Planadas, Roncesvalles, San Antonio, Santa Isabel

##### Notes

(au XC96370, ML 97306911; co SIB 126312; ph ML58239201)

#### Anisognathus somptuosus

(Lesson, 1831)

A0E1CFA6-B4B7-5D00-9AF2-310955925D5D

##### Distribution

Anzoátegui, Cajamarca, Chaparral, Cunday, Falan, Herveo, Ibagué, Libano, Murillo, Planadas, Roncesvalles, San Antonio, Santa Isabel, Villahermosa, Villarrica

##### Notes

(v; au ML99165821; ca; co CZUTOR 0643; ph ML111847321)

#### Buthraupis montana

(d'Orbigny & Lafresnaye, 1837)

B70DBF31-8846-5FDC-9CE5-138F7792C3E9

##### Distribution

Anzoategui, Casabianca, Cajamarca, Herveo, Ibagué, Murillo, Roncesvalles, San Antonio, Santa Isabel, Villarrica, Villahermosa, Villarrica

##### Notes

(v; au; ca; co CZUT OR1262; ph ML104874401)

#### Tephrophilus wetmorei

(Moore, 1934)

992AECA0-ED9E-5307-BEFD-ED8EB2A152A5

##### Distribution

Cajamarca, Ibagué

##### Notes

(v; ph https://www.inaturalist.org/photos/64883347) VU

#### Sporathraupis cyanocephala

(d'Orbigny & Lafresnaye, 1837)

2132F561-4015-5A6A-89B6-D1EEFB832DBD

##### Distribution

Anzoátegui, Cajamarca, Herrera, Herveo, Ibagué, Falan, Libano, Murillo, Planadas, Roncesvalles, San Antonio, Santa Isabel

##### Notes

(v; co MLS 6867, CZUTOR 82; ca; ph ML86906821)

#### Chlorornis riefferii

(Boissonneau, 1840)

3AD61DAF-2C56-5A34-BF64-E39F63FA442D

##### Distribution

Anzoategui, Casabianca, Falan, Herveo, Ibagué, Murillo, Planadas, Roncesvalles, San Antonio, Villahermosa

##### Notes

(v; au; co ANSP 154571; ph ML22276061)

#### Cnemathraupis eximia

(Boissonneau, 1840)

A776C193-0D48-519F-8570-848A1C6014D0

##### Distribution

Anzoategui, Cajamarca, Herveo, Ibagué, Murillo, Planadas, Roncesvalles, Santa Isabel

##### Notes

(v; co SIB 165714, CZUT OR1277; ph)

#### Chalcothraupis ruficervix

(Prevost & Des Murs, 1846)

5500C9F6-D3DD-5D33-8ECF-BFC64F62C1B3

##### Distribution

Ataco, Cajamarca, Casabianca, Ibagué, Libano, Planadas, Roncesvalles, San Antonio

##### Notes

(v; co CZUTOR 1082; ph ML79639281)

#### Stilpnia heinei

(Cabanis, 1851)

E2520442-FC41-5966-A833-1B1E996EE97A

##### Distribution

Anzoategui, Cajamarca, Casabianca, Falan, Fresno, Herveo, Ibagué, Libano, Palocabildo, Planadas, Roncesvalles, San Antonio, Santa Isabel, Villarrica

##### Notes

(v; ca; co CZUTOR 0563; ph ML86961901)

#### Stilpnia vitriolina

(Cabanis, 1851)

1C6CA4B3-662D-5917-A785-1E122FC549DD

##### Distribution

Ambalema, Armero-Guayabal, Anzoátegui, Ataco, Cajamarca, Casabianca, Chaparral, Carmen de Apicala, Chicoral, Coyaima, Cunday, Dolores, Espinal, Falan, Fresno, Guamo, Herveo, Ibagué, Icononzo, Honda, Lerida, Libano, Mariquita, Melgar, Murillo, Natagaima. Palocabildo, Planadas, Prado, Rio Blanco, Rovira, Saldaña, San Antonio, Valle de San Juan, San Luis, Suárez, Santa Isabel, Venadillo, Villarrica

##### Notes

(v; au; ca; co MLS 6668, CZUTOR 130; ph ML123826151) CE WD. This tanager is relatively common in low to middle areas of the Department (300 to 1600 m a.s.l.) and is usually seen near crops and areas of stubble in almost all of the Department (Fig. 10f).

#### Stilpnia larvata

(Du bus, 1846)

4D7A1C56-13A9-5A50-8903-66EBD75C73F1

##### Distribution

Falan, Fresno, Honda, Lerida, Mariquita

##### Notes

(co; ca; ph ML 176289291)

#### Stilpnia cyanicollis

(d'Orbigny & Lafresnaye, 1837)

5A6B7E7C-EB08-54C4-8B78-72CBBD63D382

##### Distribution

Ambalema, Anzoategui, Alvarado, Armero-Guayabal, Ataco, Chaparral, Coyaima, Cunday, Cajamarca, Dolores, Espinal, Ibagué, Falan, Fresno, Guamo, Herveo, Honda, Lerida, Libano, Mariquita, Melgar, Planadas, Prado, Rio Blanco, San Antonio, San Luis, Valle de San Juan, Villahermosa, Villarrica

##### Notes

(v; au XC424606; ca; co MLS 6612, CZUTOR 236; ph ML123825321) WD

#### Tangara vassorii

(Boissonneau, 1840)

C0926797-F950-57F5-855C-53518FD02A58

##### Distribution

Anzoátegui, Cajamarca, Casabianca, Herveo, Ibagué, Murillo, Planadas, Roncesvalles, San Antonio, Santa Isabel, Villarrica, Villahermosa, Villarrica

##### Notes

(v; au; co CZUT 0048; ca; ph ML123812521)

#### Tangara nigroviridis

(Lafresnaye, 1843)

07E7AAFD-5202-5762-B752-A715FACD7216

##### Distribution

Anzoategui, Cajamarca, Casabianca, Herveo, Ibagué, Falan, Libano, Murillo, Planada, San Antonio

##### Notes

(v; co CZUTOR 110; ph ML98502581)

#### Tangara labradorides

(Biossonneau, 1840)

57F1C5D2-F8CE-5284-8E87-C0DBAF8A7B5F

##### Distribution

Cajamarca, Casabianca, Falan, Herrera, Herveo, Ibagué, Icononzo, Libano, Murillo, Planadas, Roncesvalles, San Antonio, Santa Isabel

##### Notes

(v; co MLS 6583, CZUTOR 109; ph ML91993621)

#### Tangara inornata

(Gould, 1855)

2FB99AF9-38CF-5C36-B04B-F32176F9D0AB

##### Distribution

Alvarado, Ambalema, Anzoategui, Cajamarca, Doima, Falan, Fresno, Honda, Ibagué, Lerida, Libano, Mariquita, San Luis, Santa Isabel, Venadillo,

##### Notes

(ca; co MPUJ 823; ph ML123826071)

#### Tangara gyrola

(Linnaeus, 1758)

B4953FA2-6CD4-5C72-ADF8-61C89F91D81E

##### Distribution

Alvarado, Ambalema, Anzoategui, Armero-Guayabal, Cajamarca, Casabianca, Chaparral, Chicoral, Coyaima, Cunday, Doima, Dolores, Espinal, Falan, Fresno, Herrera, Herveo, Honda, Ibagué, Lerida, Libano, Mariquita, Melgar, Palocabildo, Planadas, Rio Blanco, Roncesvalles, Rovira, San Antonio, San Luis, Venadillo, Villahermosa, Villarrica

##### Notes

(v; au; co CZUTOR 021; ph ML123825391)WD

#### Tangara xanthocephala

(Tschudi, 1844)

0412ED62-929D-51F8-B53D-E87665AD5DB1

##### Distribution

Cajamarca, Chaparral, Dolores, Ibagué, Falan, Fresno, Herveo, Libano, Murillo, Planadas, Roncesvalles, San Antonio, Santa Isabel, Villahermosa,

##### Notes

(v; ca; co CZUTOR 0295; ph ML120521791)

#### Tangara parzudakii

(Lafresnaye, 1843)

A0172621-3E70-5E0F-A866-B444EC3C7950

##### Distribution

Chaparral, Ibagué, Libano, Murillo, Planadas

##### Notes

(v; ph ML35761761)

#### Tangara arthus

Lesson, 1832

92DB5DAB-BA81-52C8-84FA-708751C78C28

##### Distribution

Anzoátegui, Cajamarca, Casabianca, Chaparral, Herrera, Ibagué, Falan, Fresno, Herveo, Libano, Murillo, Planadas, Roncesvalles, San Antonio, Villarrica

##### Notes

(v; ca; co MLS 6572, CZUTOR 574; ph ML120522071)

#### Thraupis episcopus

(Linnaeus, 1766)

5737DB68-EB5D-5AEC-8C41-B176211B1449

##### Distribution

Armero-Guayabal, Alvarado, Anzoátegui, Ambalema, Ataco, Casabianca, Carmen de Apicala, Chaparral, Chicoral, Coyaima, Cunday, Dolores, Espinal, Falan, Fresno, Guamo, Herveo, Honda, Ibagué, Lerida, Libano, Mariquita, Murillo, Planadas, Prado, Rio Blanco, San Antonio, Santa Isabel, San Luis, Suárez, Venadillo, Villarrica

##### Notes

(v; au XC424378; ca; co MPUJ 816, CZUTOR 555; ph ML123826351) WD

#### Thraupis palmarum

Wied-Neunied, 1821

5926320A-B289-529F-8C28-BAC0A5AE9109

##### Distribution

Alvarado, Ambalema, Anzoategui, Armero-Guayabal, Ataco, Chaparral, Carmen de Apicala, Casabianca, Chicoral, Coyaima, Cunday, Dolores, Espinal, Guamo, Herveo, Honda, Ibagué, Falan, Fresno, Lerida, Libano, Mariquita, Melgar, Natagaima, Palocabildo, Planadas, Prado, Rio Blanco, San Antonio, San Luis, Santa Isabel, Suárez, Venadillo, Villarrica

##### Notes

(v; ca; co 761 MPUJ, CZUTOR 284; ph ML123826361, ML26004341) WD

#### Ixothraupis guttata

(Cabanis, 1850)

58E7F3DA-8BDA-5BF5-A415-B71E69AE99A9

##### Distribution

Casabianca, Falan, Fresno, Herveo, Honda, Ibagué, Icononzo, Libano, Planadas, San Antonio, Villahermosa

##### Notes

(co MLS 6564; ph ML123799381) This is a rare tanager in Tolima, with a few records made in the north below 1800 m a.s.l. (pers. commun., Y. Poveda). It was recently photographed in the Municipality of Planadas by R. Parra and in San Antonio by D. Vejarano (Fig. 11a).

## Analysis

**Figure 2.**
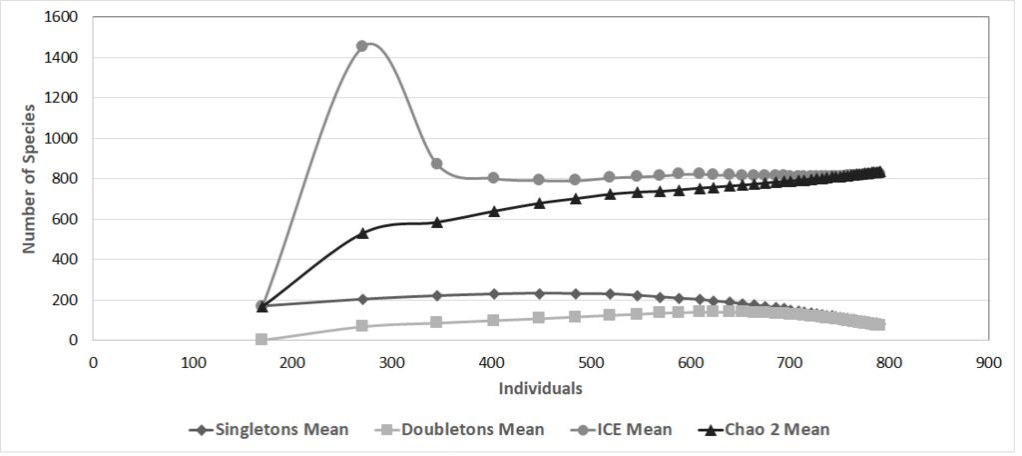
Non-parametric species richness estimators Chao 2 and ICE. Curves were based on bird species recorded at the Tolima Department, Colombia.

This study identified 800 species recorded in Tolima, representing 40% of all birds present in Colombia (Avendaño et al. 2017, Ayerbe 2018, Asociación Colombiana de Ornitología 2020). Seventeen probable species were also noted (Table 4). Bird species richness recorded in this study represents 96.7% (827) of the total number of species expected according to the Chao 2 species richness estimator and 97.2% (823) of total species expected according to the ICE species richness estimator. These results indicate that the survey was representative and that the sampling effort was sufficient to record the majority of species in the study area; however, the accumulation curves show an upward trend (Fig. 2).

The recorded species belong to 71 families and 454 genera, highlighting the presence of 82 migratory species, 24 species endemic to Colombia and 69 near-endemic to Colombia, four species with deficient data, two species considered to be regionally extinct (*Crax alberti* and *Harpia harpyja*), 42 species with some degree of threat (six critically endangered, 21 vulnerable, eight endangered and seven near threatened) and three introduced species that have become established in Colombia (the common pigeon *Columba livia*, the tricolored munia *Lonchura malacca* and the chestnut munia *Lonchura atricapilla*). The families having the greatest number of species were Tyranidae (102 species, 12.8%), Thraupidae (92 species, 11.6%), Trochilidae (68 species, 8.6%) and Furnariidae (42 species, 5.3%).

**Figure 3.**
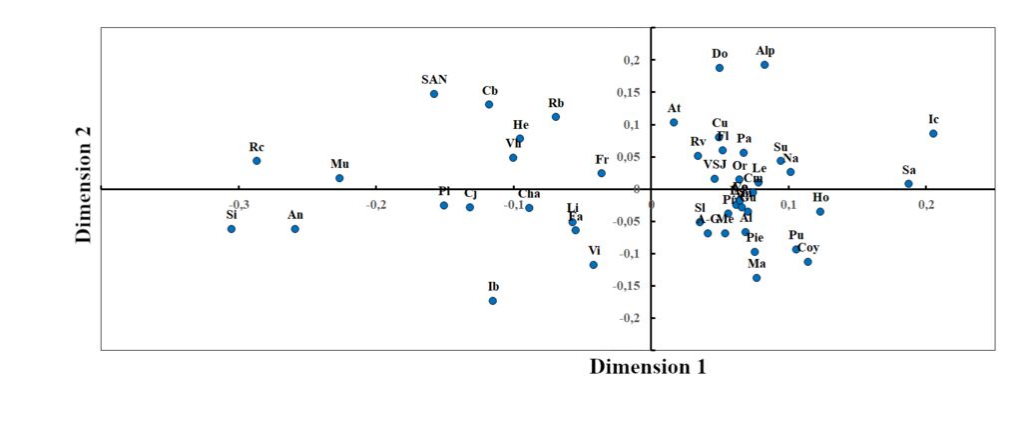
The NMDS analysis joining the municipalities on the basis of composition of bird species in the Tolima Department, Colombia (59.2% of variance explained by these two dimensions).

NDMS showed a similarity in species composition amongst municipalities related mainly to their altitude, forming a scattering of points from lower to higher elevation zones (Fig. 3). On the right side of the scatter plot are grouped the largest number of municipalities, which are principally located in lowlands with a predominant habitat of tropical dry forest. To the left are grouped nearby municipalities (e.g. Casabianca, Herveo, Villahermosa, Líbano, Falan and Fresno) that share similar altitudinal ranges, share some species typical of the middle Magdalena and high levels of species richness. At the extreme end are grouped the highland municipalities including Andean forests and páramos (Roncesvalles, Murillo, Santa Isabel, Anzoátegui, Planadas and Cajamarca).

Around 88% of species records have additional support in the form of audio recordings, photos or museum specimens.

## Discussion

This is the first updated and organised list of bird species in the Tolima Department. It will serve as an essential tool for decision-makers regarding management and conservation plans for birds in the Department. The rigorous review of the different records allows us to conclude that around 96% of species included in the list are represented by two or more records and about 91% of species have been reported in two or more localities.

Some species that are present as single records can be considered as rare or extinct in the area. For example, the Magdalena tinamu or red-legged tinamou (*Crypturellus erythrops saltuarius*), blue-billed curassow (*Crax alberti*) and orange-breasted falcon (*Falco deiroleucus*) represent historical records. Some species were likely observed due to erratic migration, such as *Coturnicops notatus*, *Nyctanassa violacea* and *Syrigma silbilatrix*. For cases such as *Ixobrinchus exilis* and *Pipreola aureopectus*, the species are thought to be very rare.

The northern zones of the Department were identified as those with the greatest species richness, not only because they represent the majority of monitoring or ornithological studies, but also because they registered the most localities sampled — both in area and in time. By contrast, the southern and eastern zones are the least explored, with outdated or non-existent inventories. These gaps in data result in a lower bird richness compared to other Departments in Colombia. Departments like Cauca and Nariño have greater richness of birds, mainly due to biomes or life zones not present in Tolima, such as mangroves and tropical rainforest (i.e. those found in Amazonian forests), as well as coastal and island habitats (Ayerbe et al. 2008, Calderón et al. 2011).

The Department of Cundinamarca is comparable to Tolima because it is located in the Andean Region and has similar life zones. It has a total of 941 species, of which 718 are shared with Tolima. Of the remaining 223 species, 51 are typical of piedmont, 12 are exclusive to the Cundiboyacense plateau, 26 belong to the north of the Magdalena Valley, 108 are restricted to the Eastern mountain range, 17 are sporadic migratory species and 8 are casual wanderers. As the Department of Cundinamarca borders on Tolima, it is expected that monitoring efforts in the bordering areas will increase the number of species in Tolima by more than 15, particularly those distributed in the Middle Magdalena Valley and migratory and erratic species. Similar conditions are expected to occur in areas near the limits of Departments such as Huila and Cauca and in municipalities such as Planadas, Ataco, Natagaima and Alpujarra.

It is worth highlighting that the Municipality of Ibagué contains the highest diversity of birds in Tolima with 675 species (Table 5). The importance of creating protected areas in the tropical dry forest of this region should be addressed. In particular, Important Bird and Biodiversity Areas (IBAs) should be established in the area around Natagaima and Villavieja (Huila Department) due to the presence of species of special interest and its being one of the most disturbed ecosystems in Colombia.

Finally, we encourage the continuation of the process of ornithological characterisation in the Department, mainly in these areas with scarce or no records, where not only the number of regional species will surely increase, but also the new distribution reports and probably even new species for science will be identified.

## Supplementary Material

B717A5E1-07E0-5113-B486-530877A08805 10.3897/BDJ.12.e68286.suppl1 Supplementary material 1 Bird species recorded within Tolima Department, Colombia. Data type Occurrences, ecological data and municipal distribution. Brief description Conventions: With an asterisk (*) the species registered inEbird http://ebird.org/ebird/subnational1/CO-TOL?yr=all (version on-line 01-04-2020). Number of the voucher in: MNHNP = Muséum National d’Histoire Naturelle Paris, DNHM = Delaware NaturalHistory Museum, WFVZ = Western Foundation of Vertebrate Zoology, FMNH = The Field Museum ofNatural History, CUMV = Cornell University Museum of Vertebrates, NHMA = Natural History Museumof Los Angeles County, NMNH = Smithsonian National Museum of Natural History, AMNH = American Museum of Natural History, MCZ = Harvard Museum of Comparative Zoology, MLS = Museo la Salle, SIB = Colección Humboldt IAvH, MPUJ = Museo Pontificie Universidad Javeriana, CZUTOR = Colección Zologica de la Universidad del Tolima - referencia Ornitologia, MHN-ICN = Museo de historia natural, instituto de Ciencias Naturales Universidad Nacional de Colombia, IVI = Insituto vallecaucano de investigación. Locality: Municipalities: the municipalities appearing in the records mentioned in this study are represented by the following acronyms: (A-G) Armero-Guayabal, (Al) Alvarado, (Am) Ambalema, (An) Anzoátegui, (At)Ataco, (Cb) Casabianca, (Cha) Chaparral, (Cj) Cajamarca, (Cm) Carmen de Apicala, (Co) Coyaima, (Cu) Cunday, (Do) Dolores, (Es) Espinal, (Fa) Falan, (Fl) Flandes, (Fr) Fresno, (Gu) Guamo, (Gy) Gualanday, (He) Herveo, (Ho) Honda, (Ib) Ibagué, (Ic) Icononzo, (Le) Lerida, (Li) Libano, (Ma) Mariquita, (Me) Melgar, (Mu) Murillo, (Na) Natagaima, (Or) Ortega, (Pie) Piedras, (Pl) Planadas, (Pr) Prado, (Pu) Purificación, (Rb) Rio Blanco, (Rc) Roncesvalles, (Rv) Rovira, (Sa) Saldaña, (Si) Santa Isabel, (Sl) San Luis, San Antonio (SAN), (Su) Suarez, (Ve) Venadillo, (Vh) Villahermosa and (Vi) Villarrica. Habitat: (alp) aquatic, lakes and ponds, (apf) aquatic, paddy fields, (ars) aquatic, rivers and streams, (as) airspace, (aw) aquatic, wetlands, (cga) coffee growing area, (ch) chusque (thin bamboo thickets), (cv) caves, (dfm) dry forest matorral, (dfv) dry forest vegetation, (fe) forest edge, (g) guadua (extensive thickets of giant bamboo), (gf) gallery forest, (mf) mature forest, (oa) open area, (ocga) other crop-growing area, (pf) primary forest, (p) paddocks and pastureland, (ppt) pine plantation forest, (ppt) paramo/desert vegetation, (rf) riparian forest, (rfa) rice-farming area, (mr) riparian matorral, (sf) secondary forest, (sgm) secondary growth matorral, (sppt) subparamo vegetation, (sscm) stream-side cliffs and mountains, (ua) urban area, (us) understorey, (wf) wet forest. Observations: type of record: (v) visual, (au) auditory, (ca) capture, (co) collection and (ph) photographic. Distribution (Chaparro et al. 2013): (E) endemic, (CE) almost endemic, (M) migratory, (EL) extralimital, (INT) introduced, (RINT) reintroduced and (F) probable translocation and/or flight from an area. Threat category: (CR) critically endangered, (EN) endangered, (VU) vulnerable, (NT) nearly threatened, (EX) considered as being extinct at national or departmental level and (WD) widely distributed throughout the Department. File: oo_1474088.xlsx https://binary.pensoft.net/file/1474088 Ronald M Parra-Hernández, Yair G Molina - Martínez

8826044A-6FAA-57F9-AEAB-C9EE8E5F440E 10.3897/BDJ.12.e68286.suppl2 Supplementary material 2 Checklist of bird species by municipality, used to carry out the NMDS analysis Data type Occurrence Brief description The municipalities appearing in the records mentioned in this study are represented by the following acronyms: (A-G) Armero-Guayabal, (Al) Alvarado, (Alp) Alpujarra, (Am) Ambalema, (An) Anzoátegui, (At) Ataco, (Cb) Casabianca, (Cha) Chaparral, (Cj) Cajamarca, (Cm) Carmen de Apicala, (Co) Coyaima, (Cu) Cunday, (Do) Dolores, (Es) Espinal, (Fa) Falan, (Fl) Flandes, (Fr) Fresno, (Gu) Guamo, (Gy) Gualanday, (He) Herveo, (Ho) Honda, (Ib) Ibagué, (Ic) Icononzo, (Le) Lerida, (Li) Libano, (Ma) Mariquita, (Me) Melgar, (Mu) Murillo, (Na) Natagaima, (Or) Ortega, (Pie) Piedras, (Pl) Planadas, (Pr) Prado, (Pu) Purificación, (Rb) Rio Blanco, (Rc) Roncesvalles, (Rv) Rovira, (Sa) Saldaña, (Si) Santa Isabel, (Sl) San Luis, San Antonio (SAN) (Su) Suarez, (Ve) Venadillo, (Vh) Villahermosa and (Vi) Villarrica. File: oo_1474157.xlsx https://binary.pensoft.net/file/1474157 Ronald M Parra-Hernández & Yair G Molina - Martínez

## Figures and Tables

**Figure 1.**
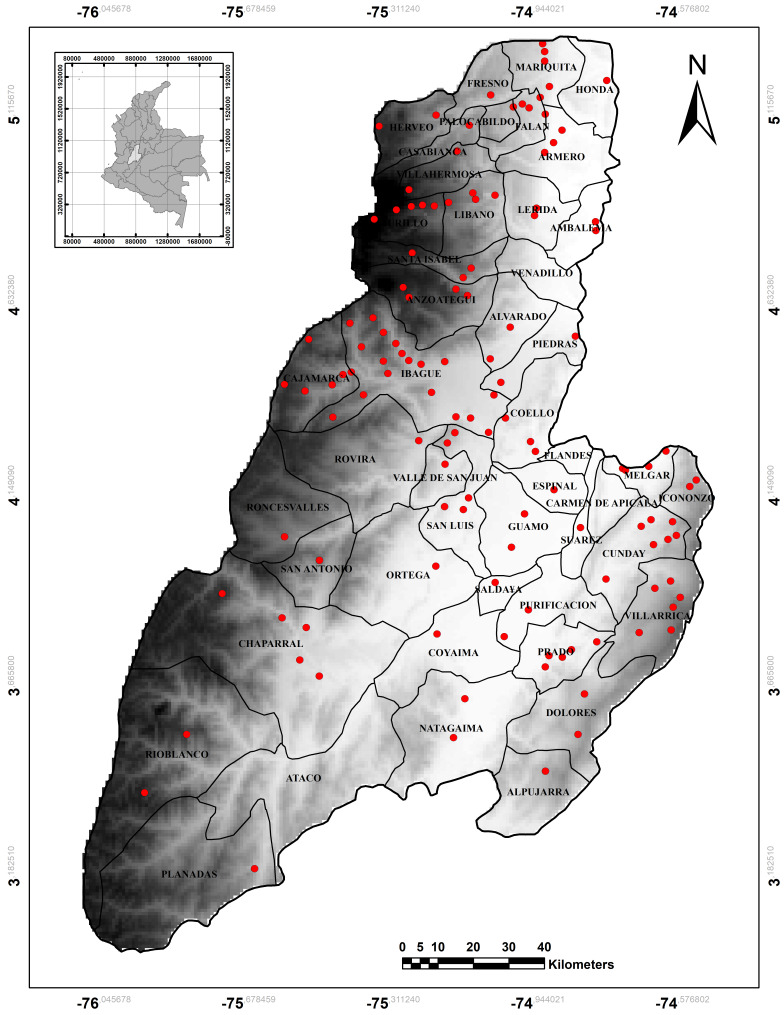
Tolima Department Map, Colombia. Inset shows the location of Tolima in Colombia. The red points indicate localities considered in this study.

**Figure 4a.**
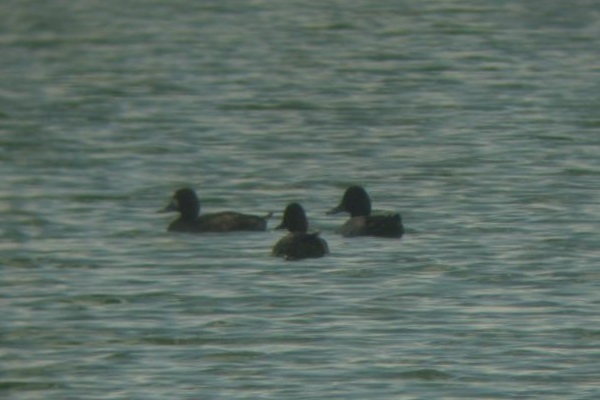
*Aythya affinis*;

**Figure 4b.**
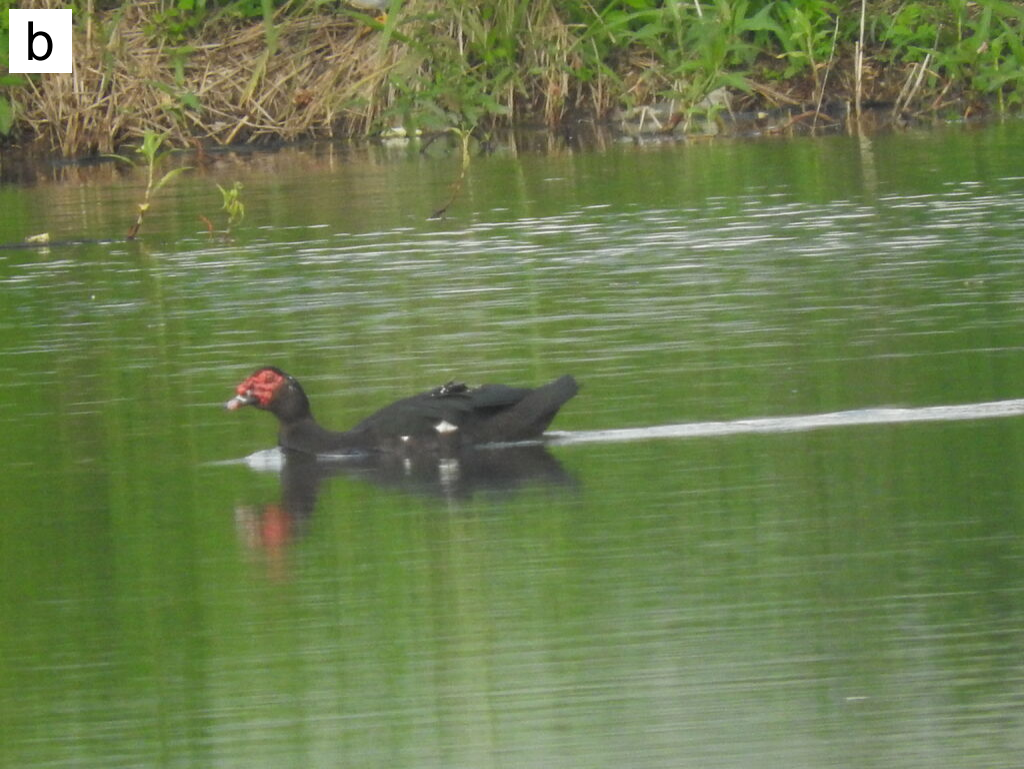
*Cairina moschata*

**Figure 4c.**
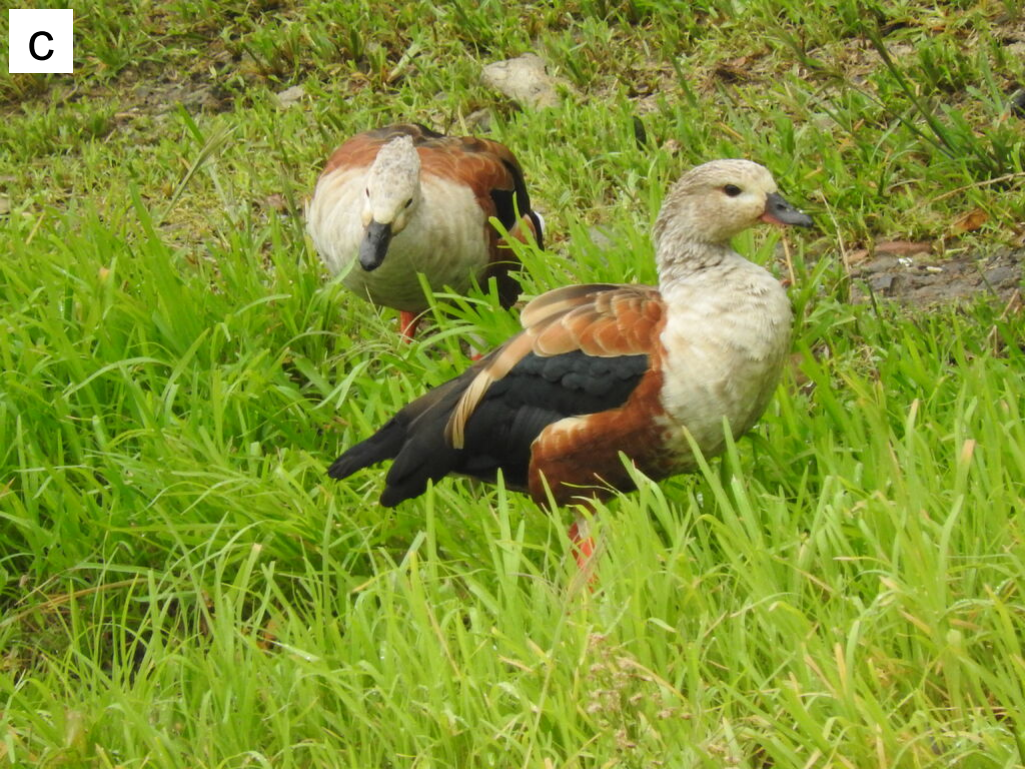
*Oressochen jubatus*

**Figure 4d.**
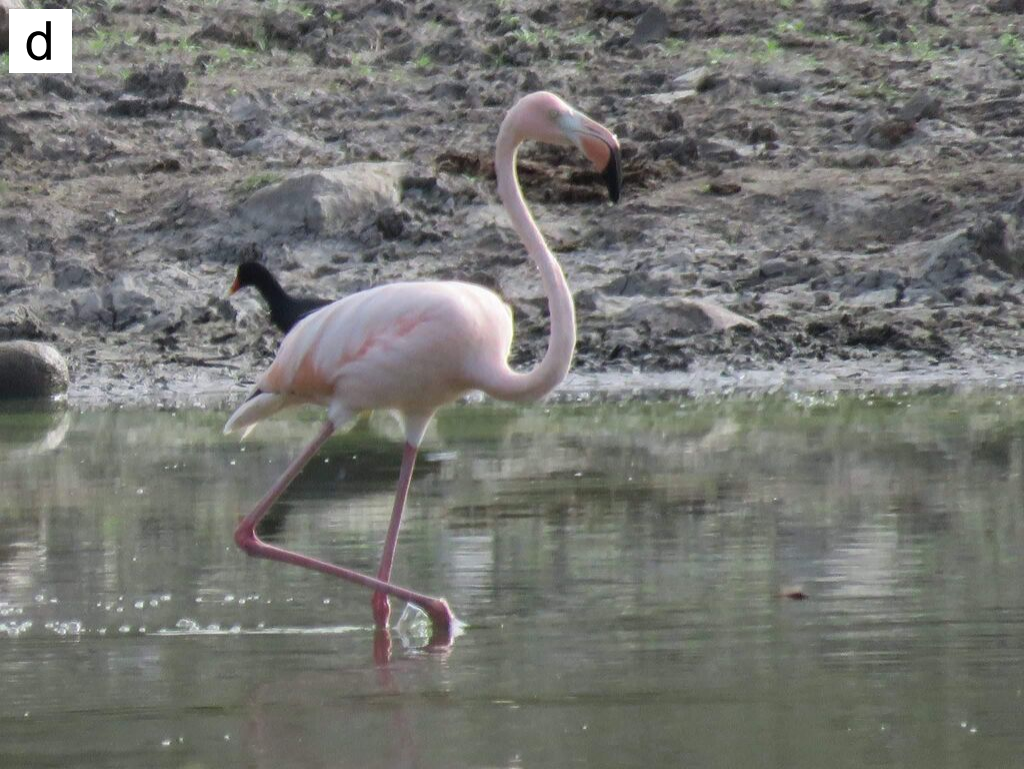
*Phoenicopterus ruber*

**Figure 4e.**
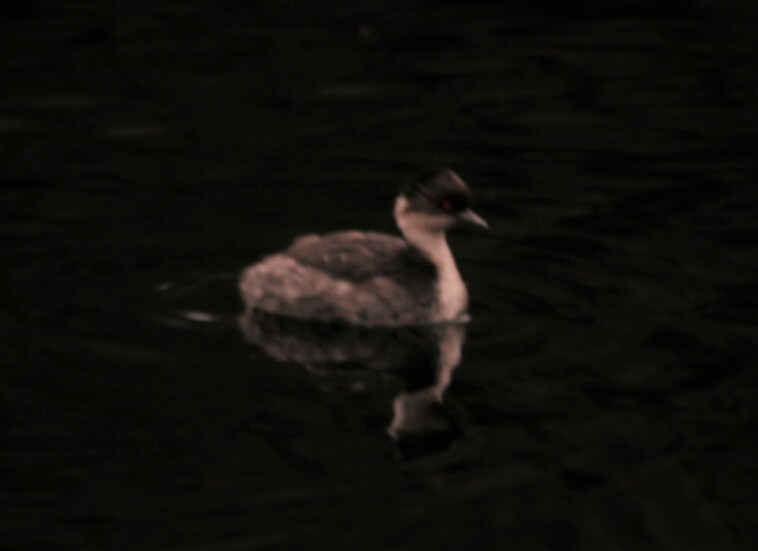
*Podiceps occipitalis*.

**Figure 5a.**
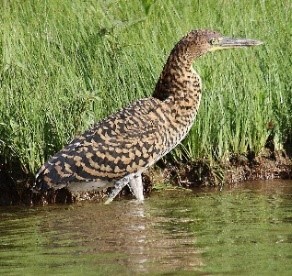
*Tigrisoma lineatum* (photograph of W. Figueroa);

**Figure 5b.**
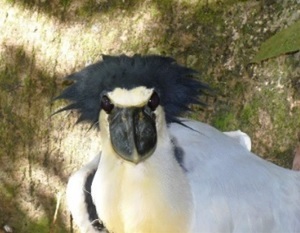
*Cochlearius cochlearius*, a specie unusual;

**Figure 5c.**
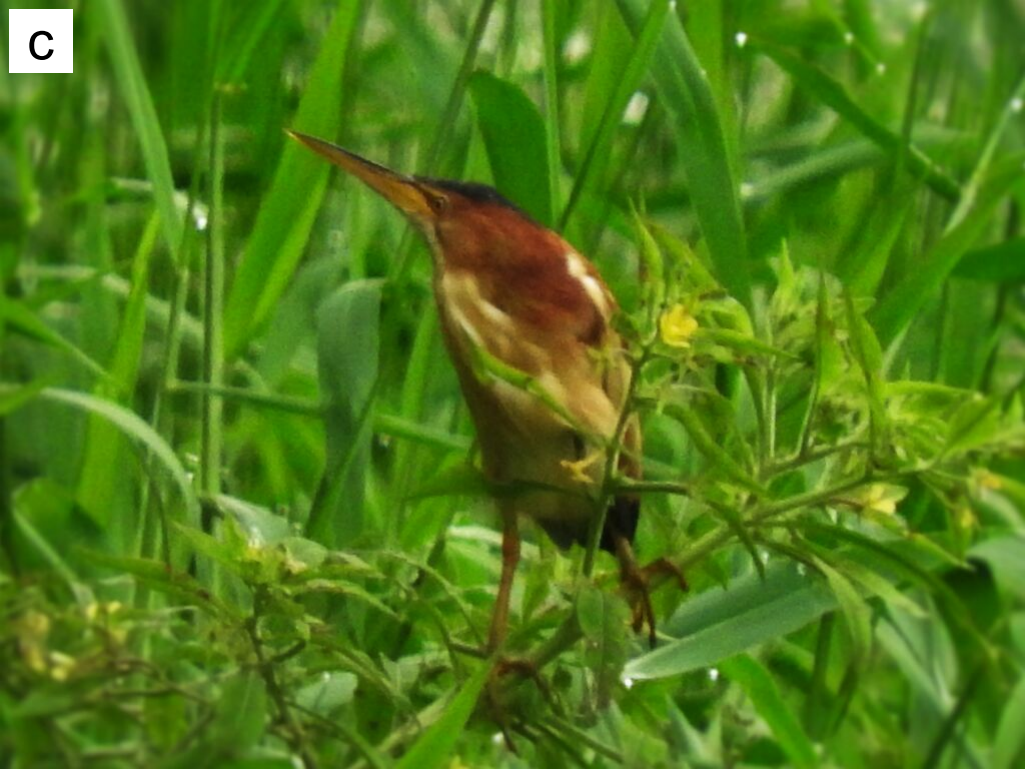
*Ixobrychus exilis*, new registration high valley of Magdalena;

**Figure 5d.**
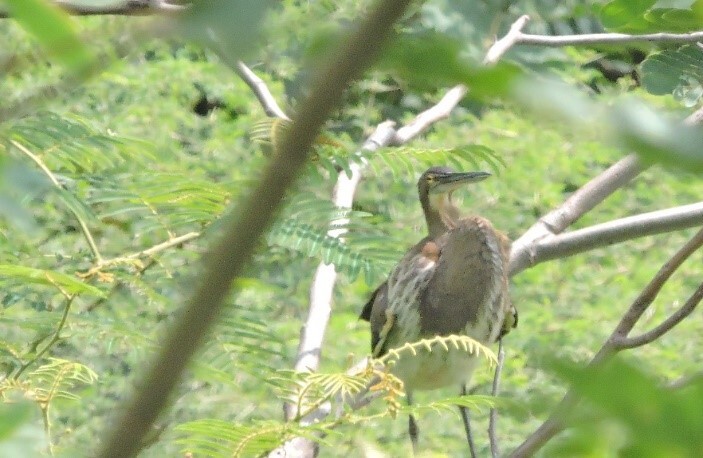
*Agami agami* (photograph of Y. Tolosa - Proyecto Bosque Seco, Cortolima);

**Figure 5e.**
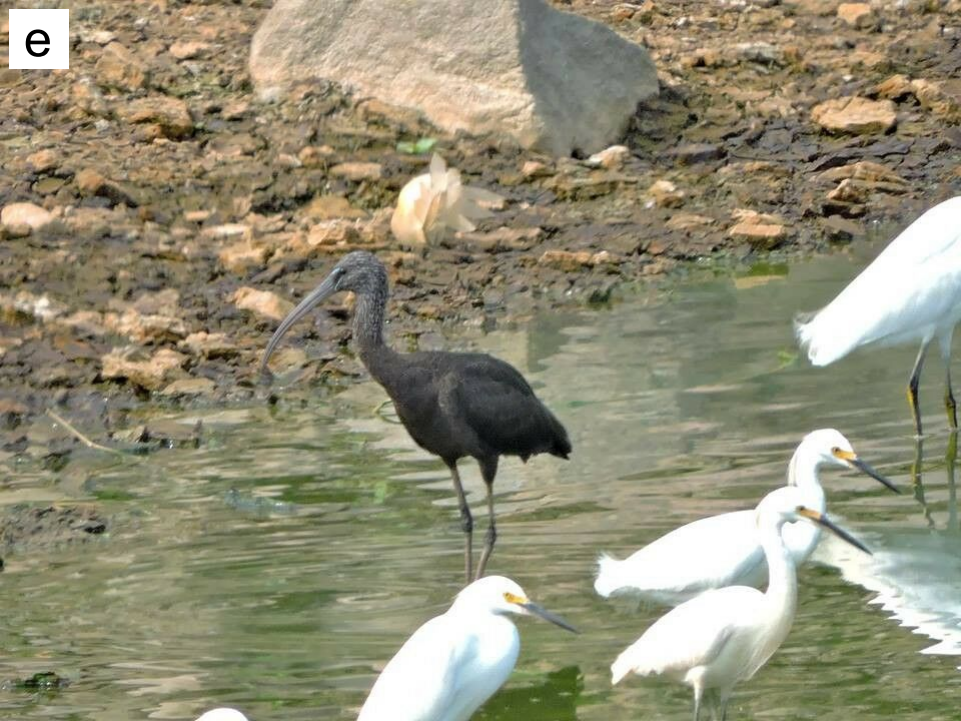
*Plegadis falcinellus*, new registration high valley of Magdalena (photograph of F. Espinosa);

**Figure 5f.**
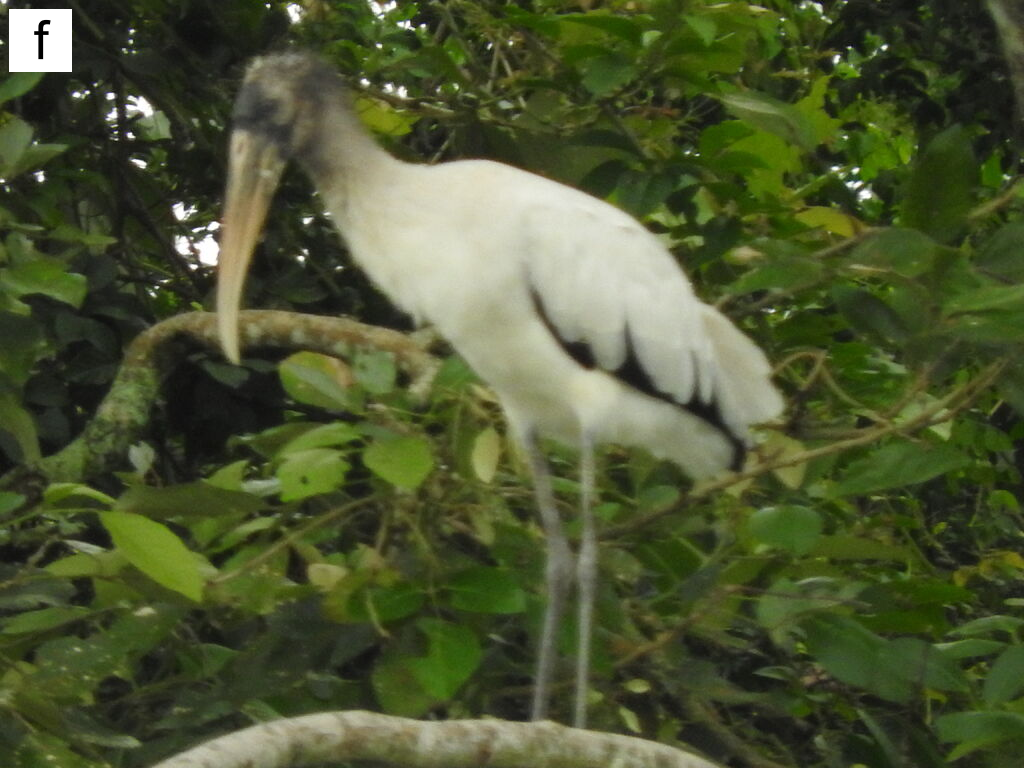
*Mycteria americana*, a specie unusual.

**Figure 6a.**
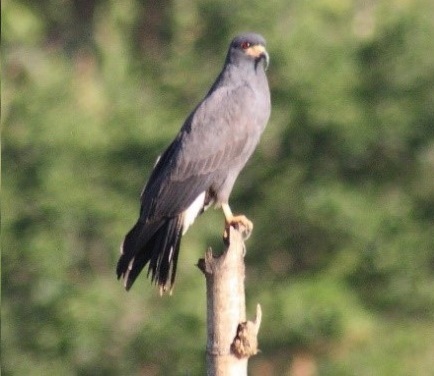
*Rosthraumus sociabilis* (photograph of W. Figueroa);

**Figure 6b.**
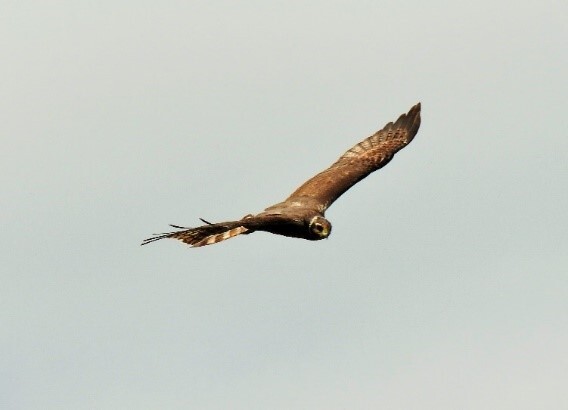
*Circus bufoni*, new registration high valley of Magdalena;

**Figure 6c.**
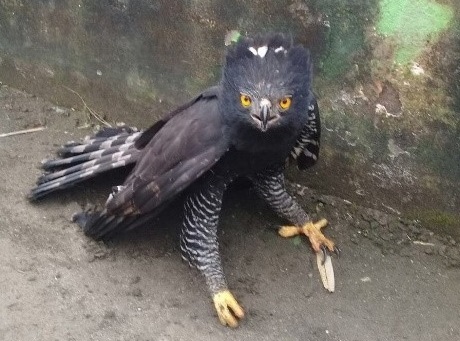
*Spizaetus tyrannus* (photograph of J. W. Molano);

**Figure 6d.**
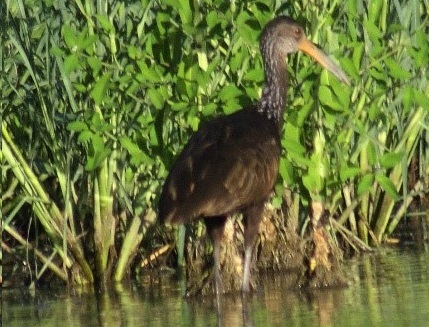
*Aramus guararauna* (photograph of W. Figueroa);

**Figure 6e.**
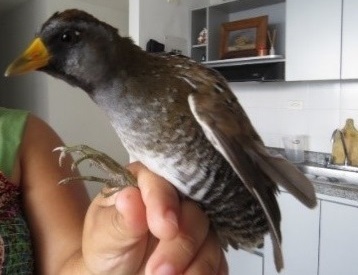
*Porzana carolina*, a migratory specie (photograph of F. Suarez);

**Figure 6f.**
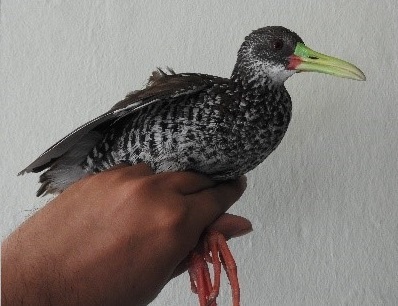
*Pardirallus maculatus*.

**Figure 7a.**
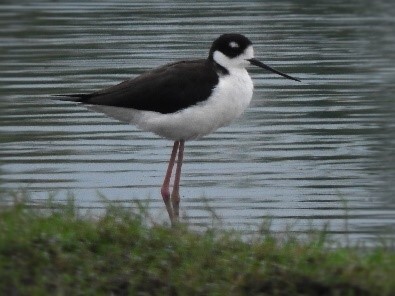
*Himantopus mexicanus*;

**Figure 7b.**
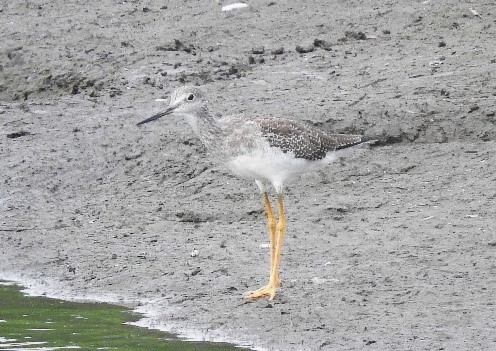
*Tringa melanoleuca*;

**Figure 7c.**
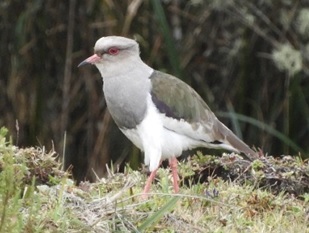
*Vanellus resplandes* (photograph of C. Guevara);

**Figure 7d.**
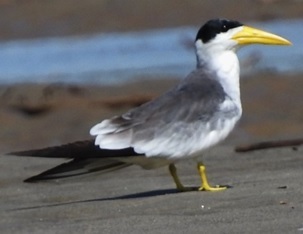
*Phaetusa simplex*;

**Figure 7e.**
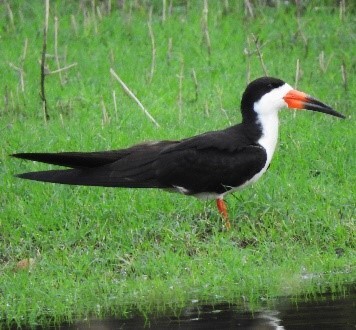
*Rhynchops niger*;

**Figure 7f.**
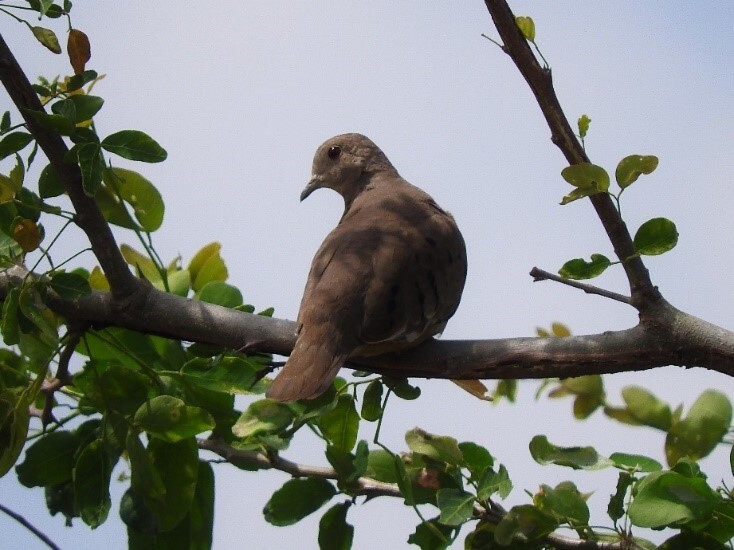
*Columbina minuta*.

**Figure 8a.**
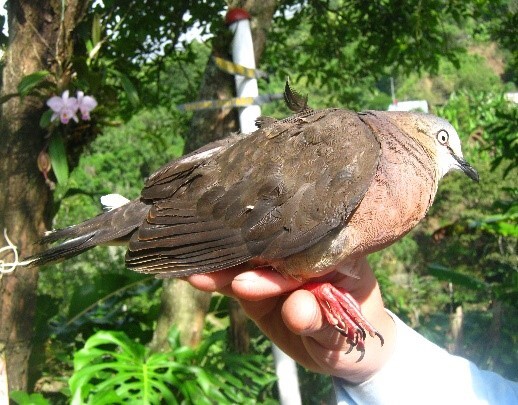
*Leptotila conoveri*, an endangered and endemic (photograph of William E. Figueroa);

**Figure 8b.**
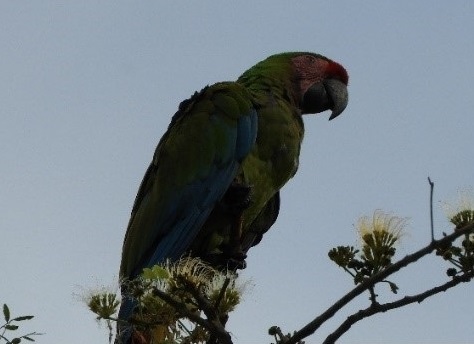
*Ara militaris* (photograph of C. Guevara);

**Figure 8c.**
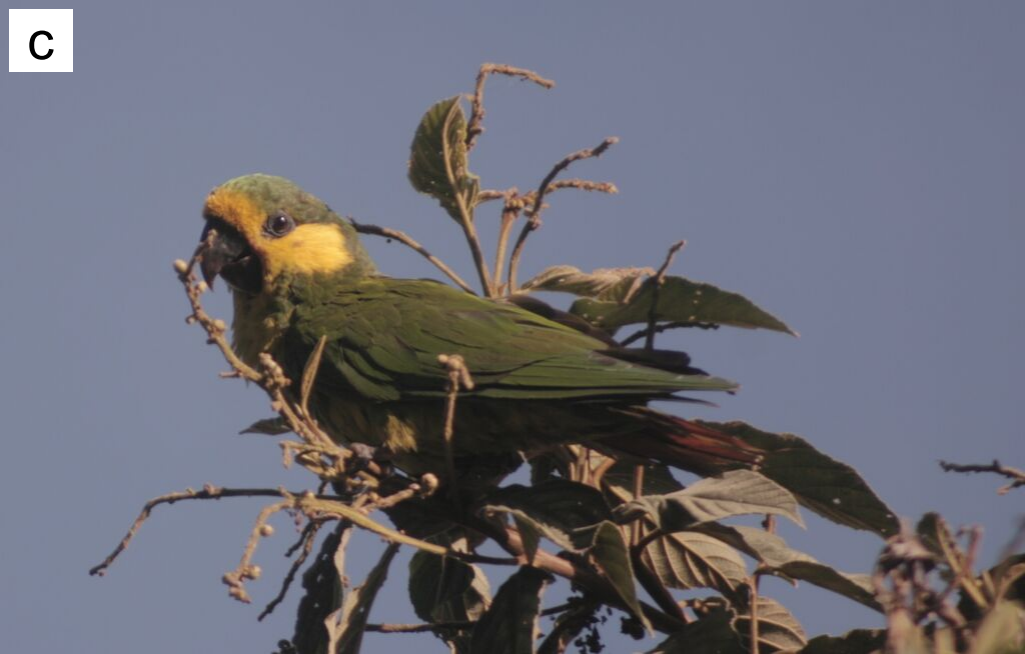
*Ognorhynchus icterotis* (endangered, photograph of Wilbert Yate);

**Figure 8d.**
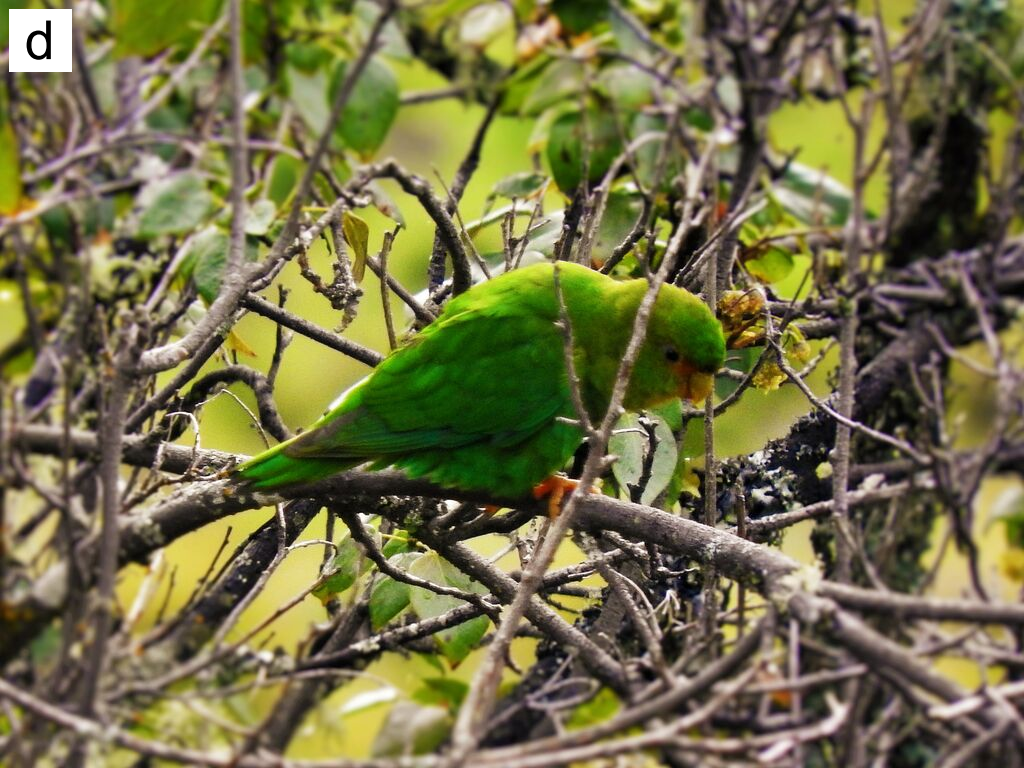
*Bolborhynchus ferrugineifrons* (endangered and endemic);

**Figure 8e.**
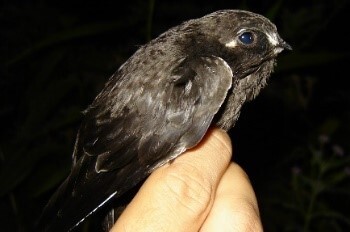
*Cypseloides cherriei*;

**Figure 8f.**
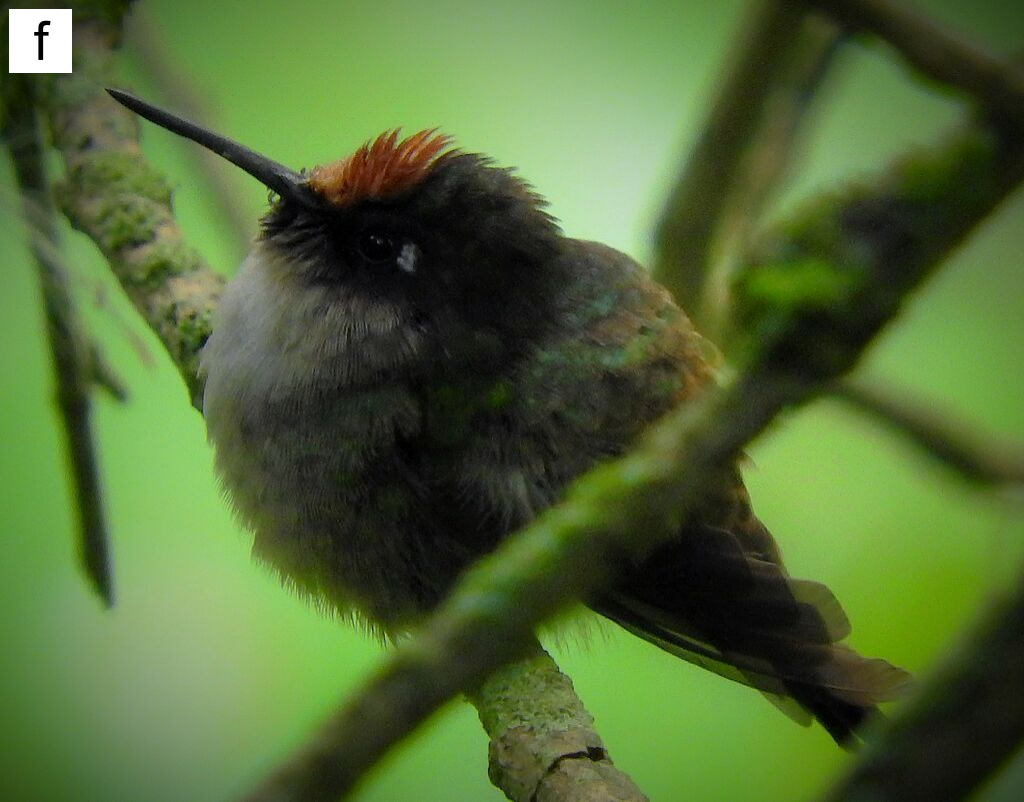
*Anthocephala berlepschi*, an endangered and Colombian Central Andes endemic species.

**Figure 9a.**
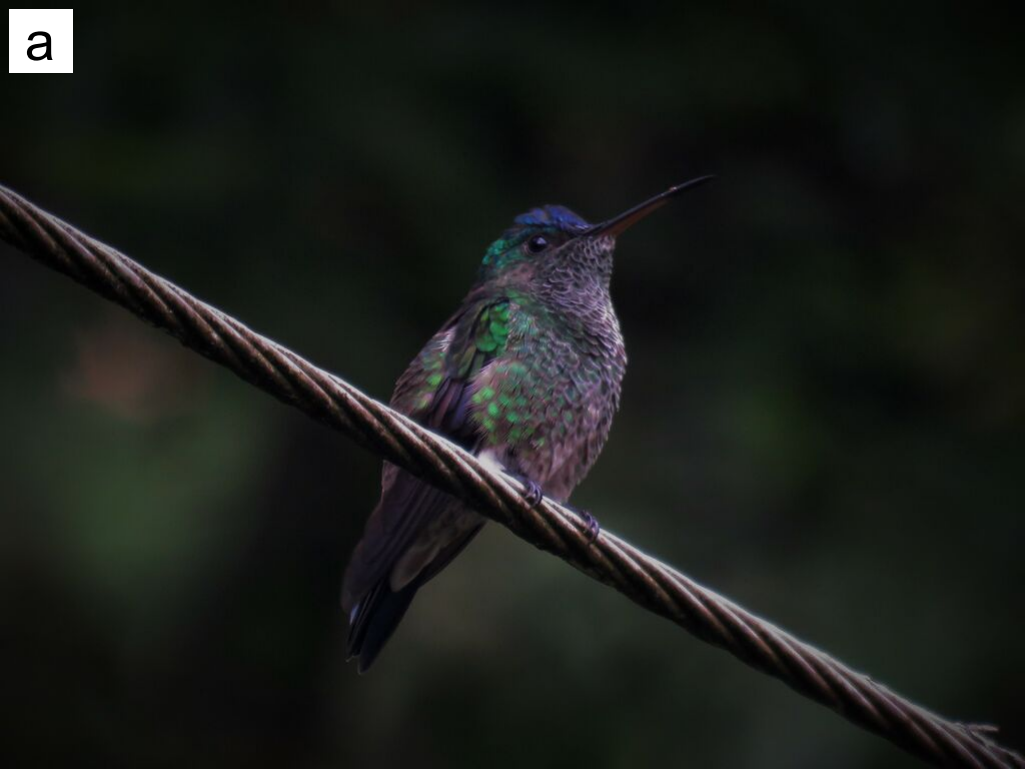
*Saucerottia cyanifrons* (near endemic);

**Figure 9b.**
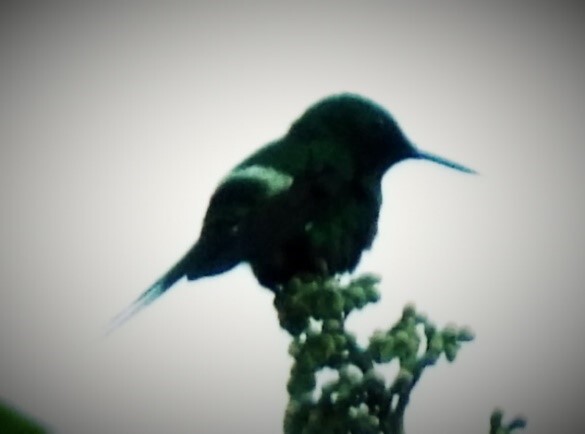
*Discosura conversii*;

**Figure 9c.**
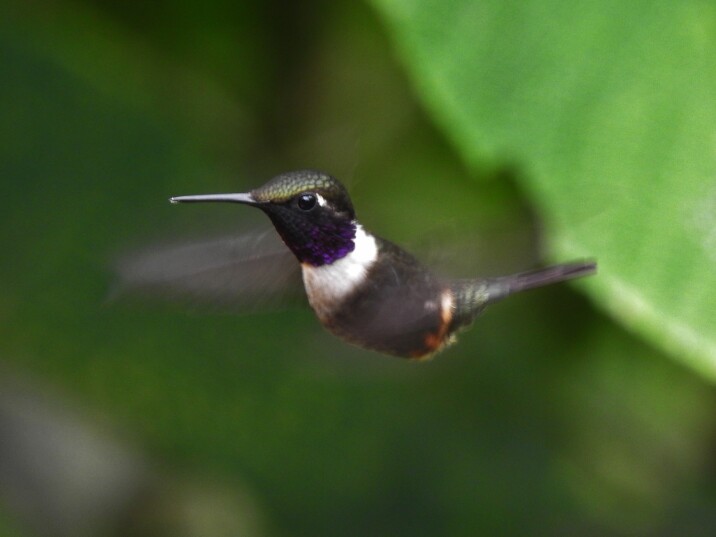
*Calliphlox mitchellii*

**Figure 9d.**
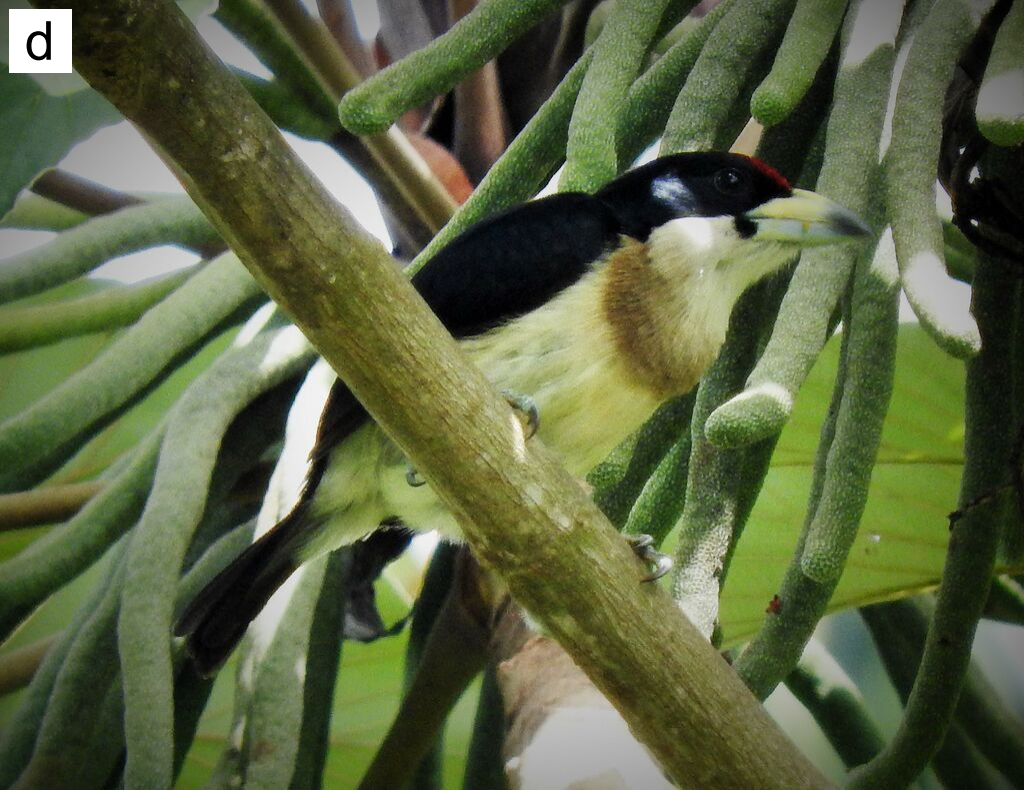
*Capito hypoleucus* (endangered and endemic);

**Figure 9e.**
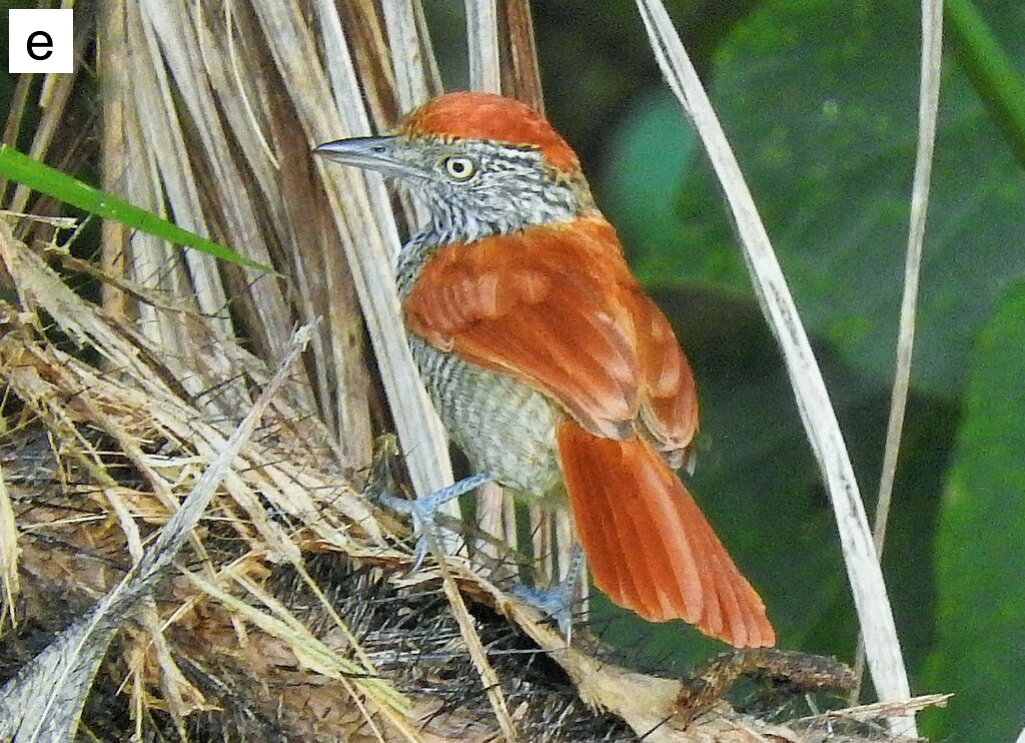
*Thamnophilus multristriatus*;

**Figure 9f.**
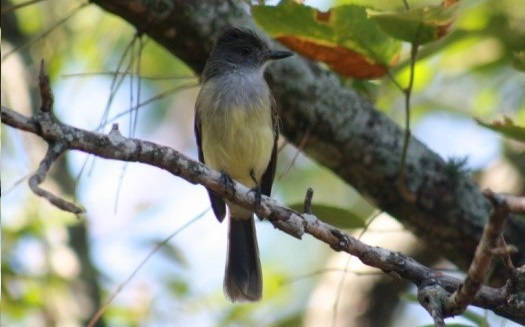
*Myiarchus apicalis*, an endemic species (photograph of William E. Figueroa).

**Figure 10a.**
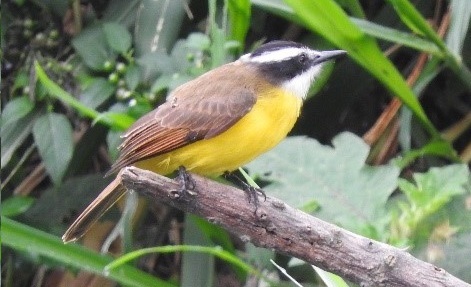
*Pitangus lictor*;

**Figure 10b.**
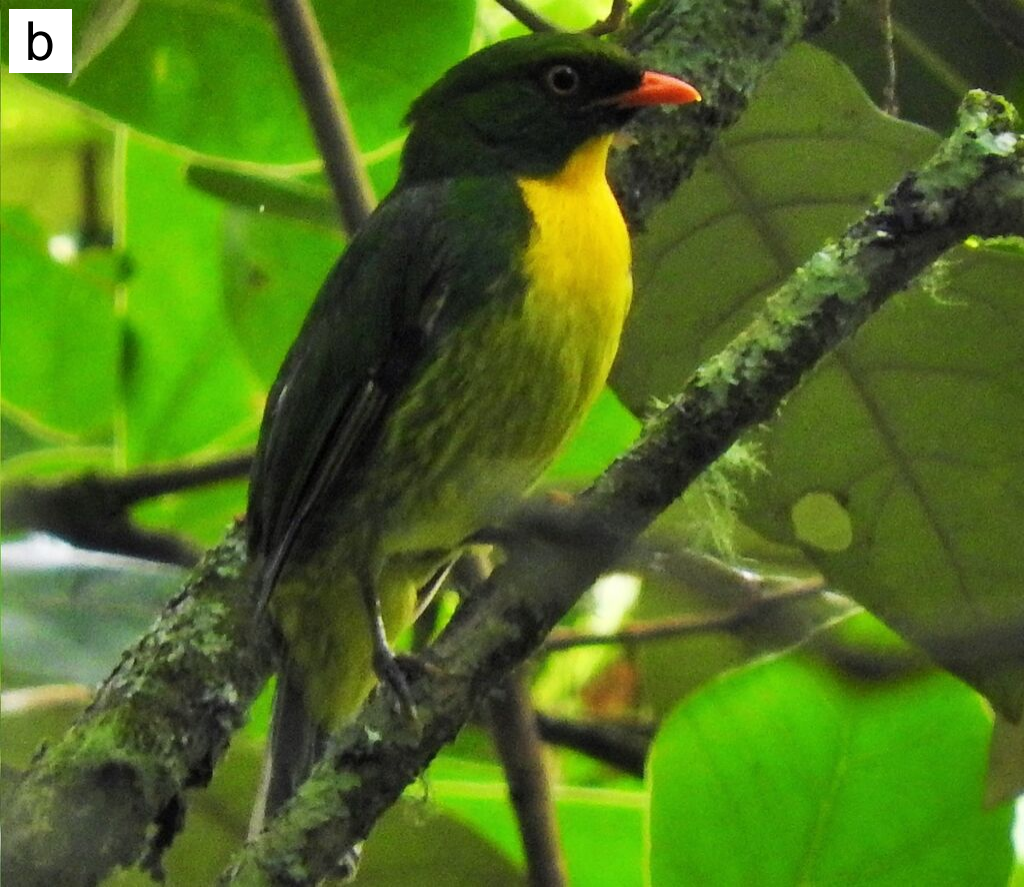
*Pipreola aureopectus*, a new registration in the high valley of Magdalena;

**Figure 10c.**
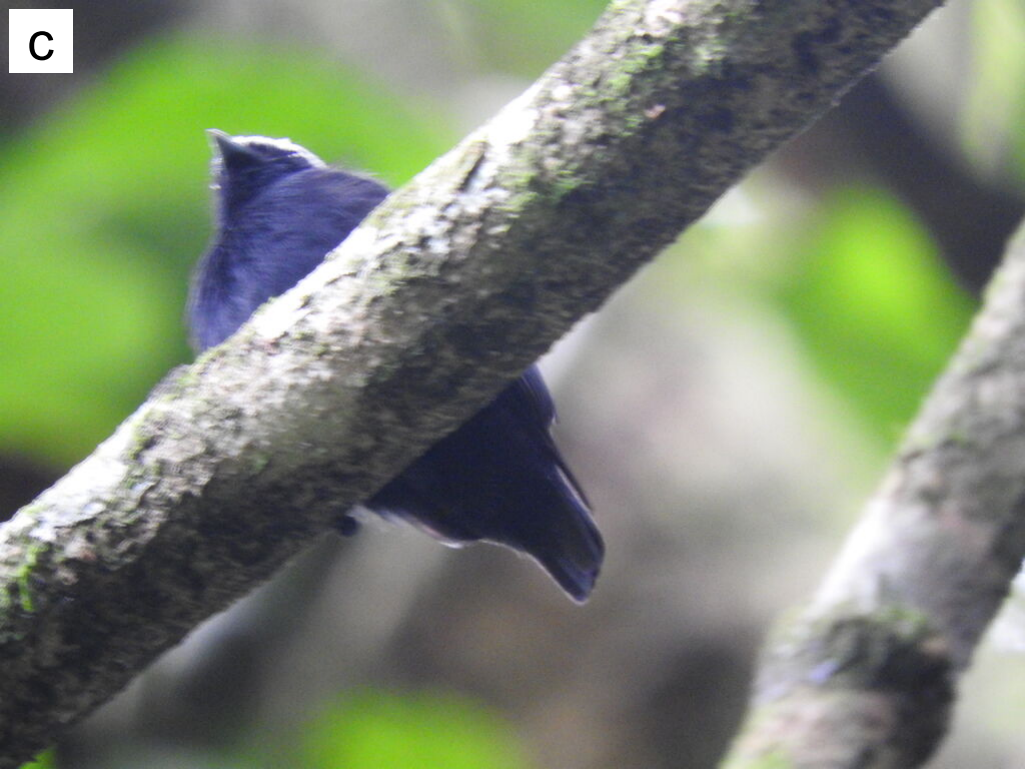
*Pseudopipra pipra*

**Figure 10d.**
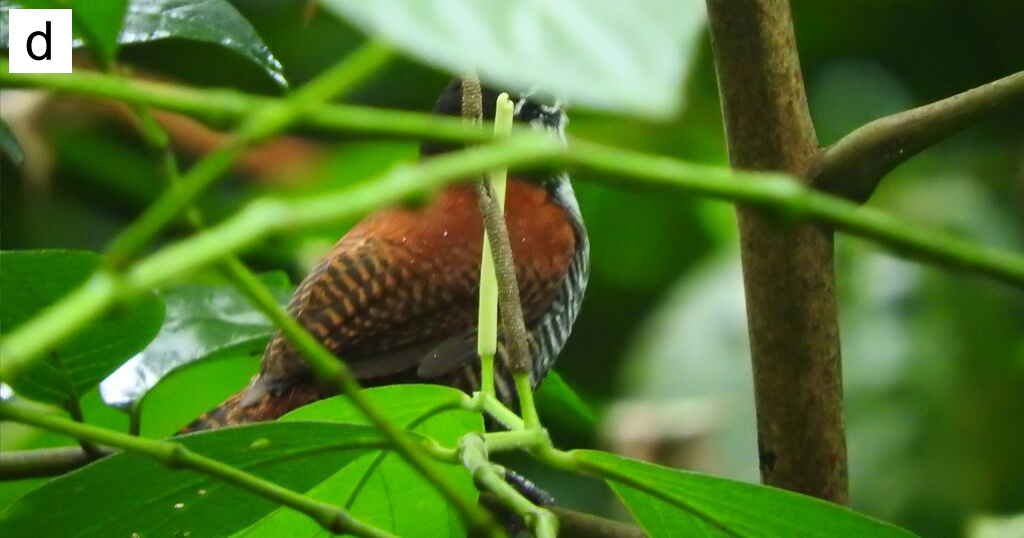
*Cantorchilus nigrocapillus*, a new registration in the high valley of Magdalena;

**Figure 10e.**
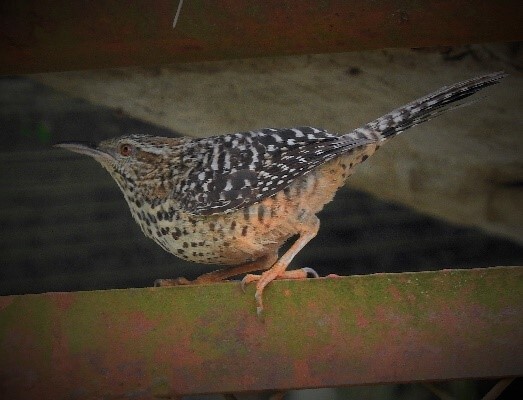
*Campylorhynchus zonatus*

**Figure 10f.**
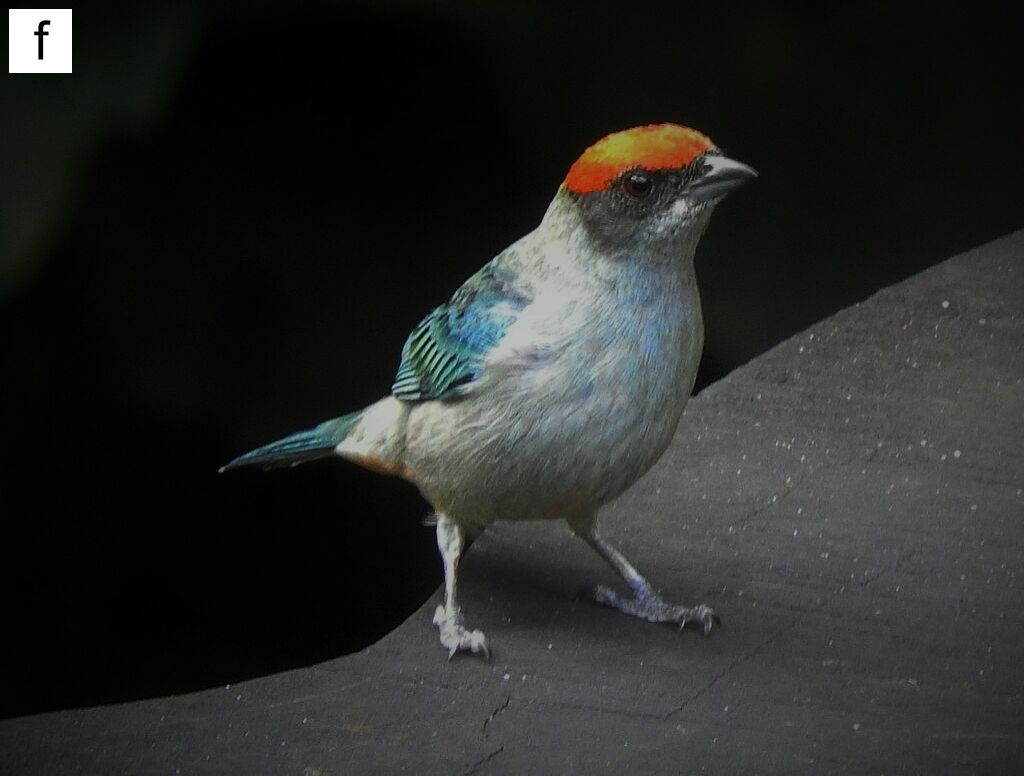
*Stilpnia vitriolina*, a semi-endemic species.

**Figure 11a.**
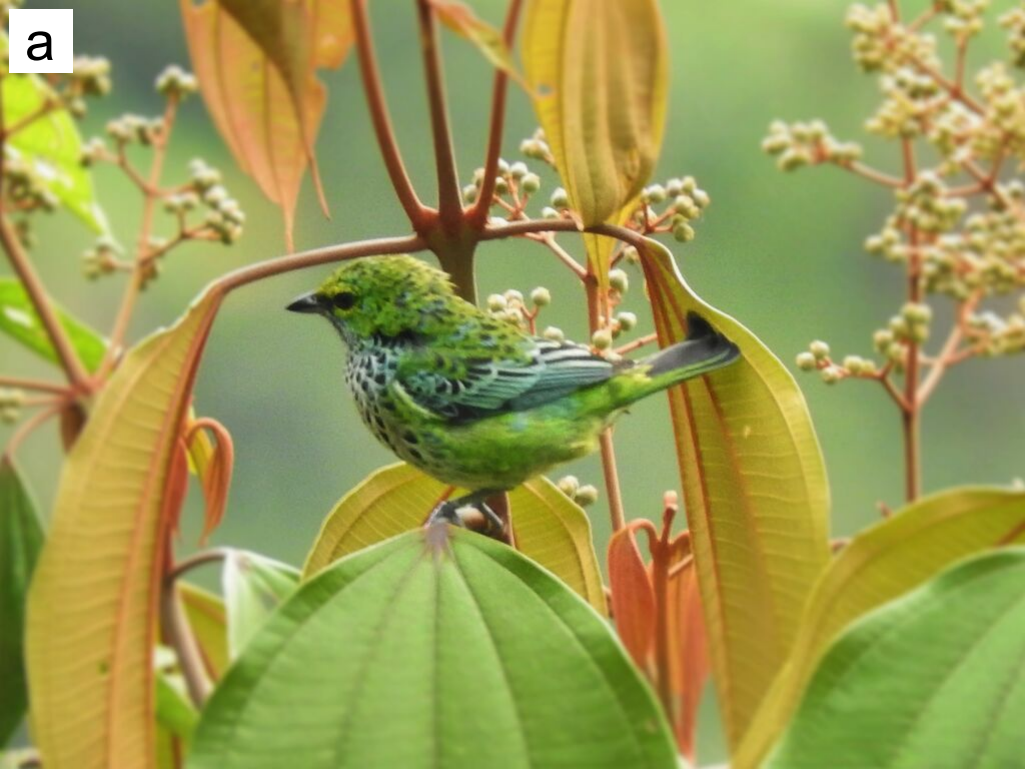
*Ixothraupis guttata*;

**Figure 11b.**
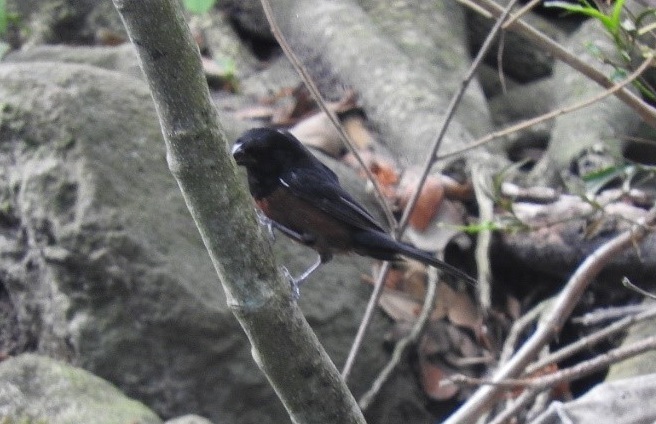
*Sporophila angolensis* (photograph of W. Figueroa);

**Figure 11c.**
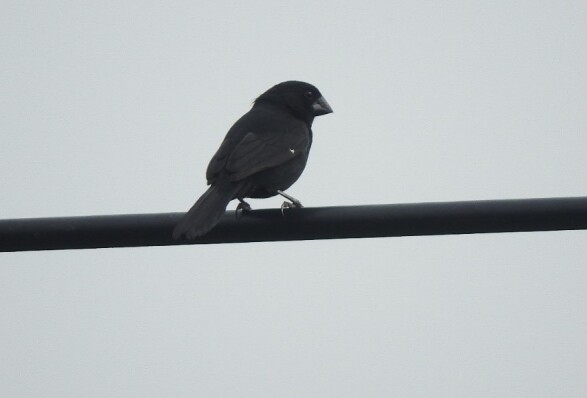
*Sporophila funerea*;

**Figure 11d.**
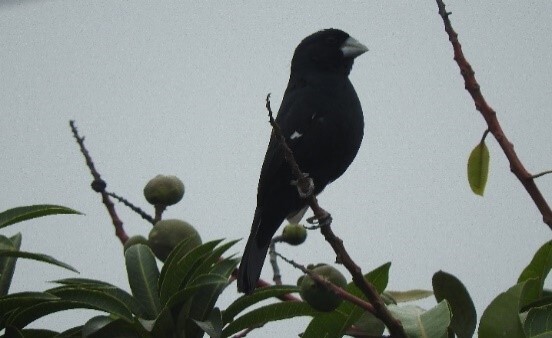
*Sporophila crassirostris*;

**Figure 11e.**
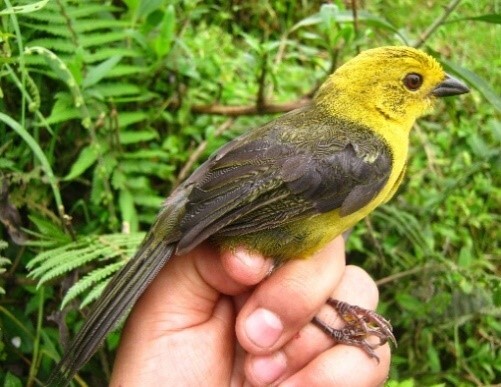
*Atlapetes flaviceps*, an endangered and Colombian Central Andes endemic species;

**Figure 11f.**
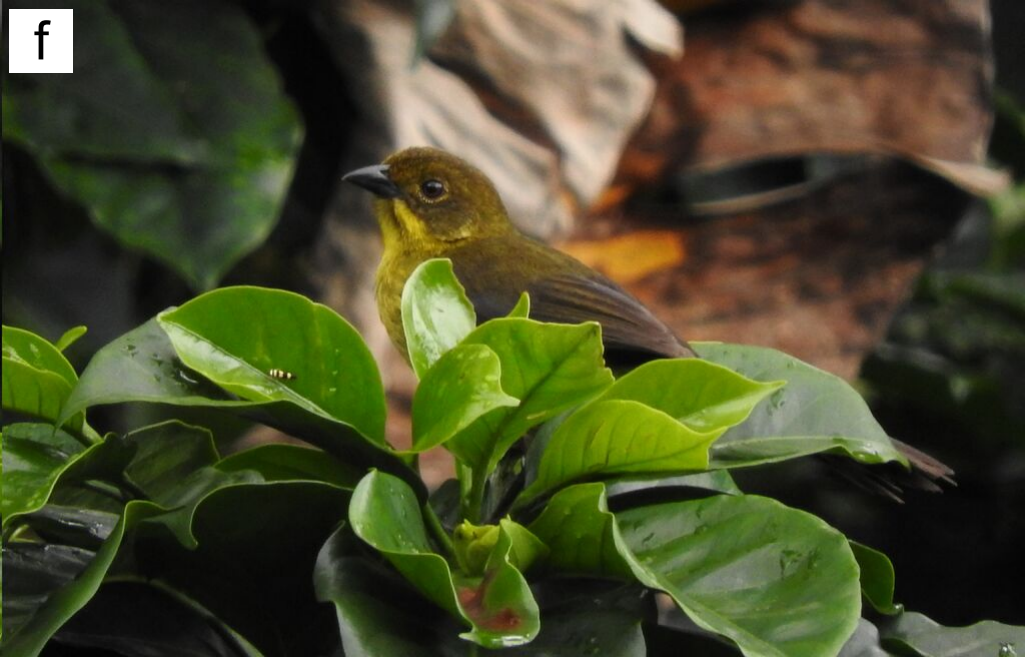
*Atlapetes fuscoolivaceus*, an endangered and Colombian Central Andes endemic species.

**Figure 12a.**
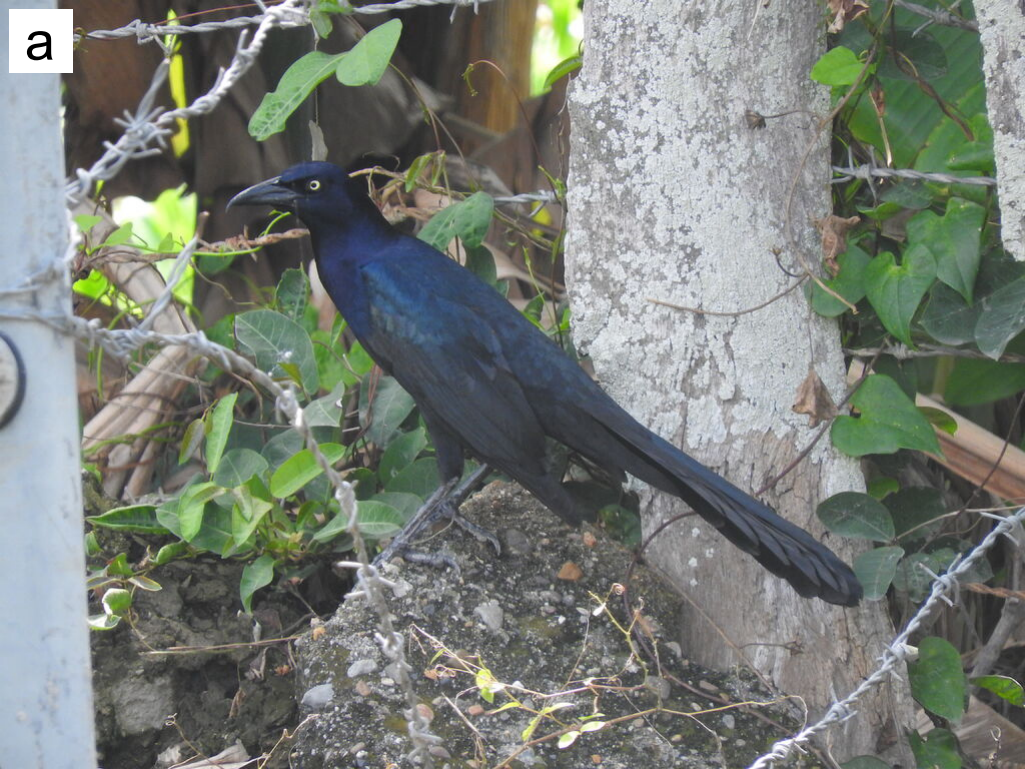
*Quiscalus mexicanus*, a species that has expanded its distribution;

**Figure 12b.**
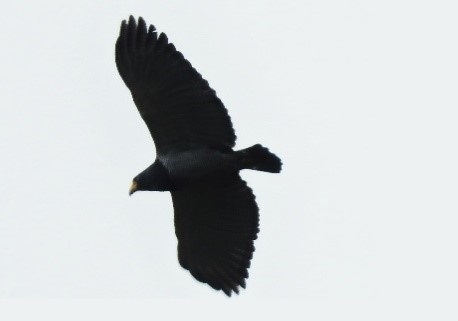
*Morphnarchus princeps*;

**Figure 12c.**
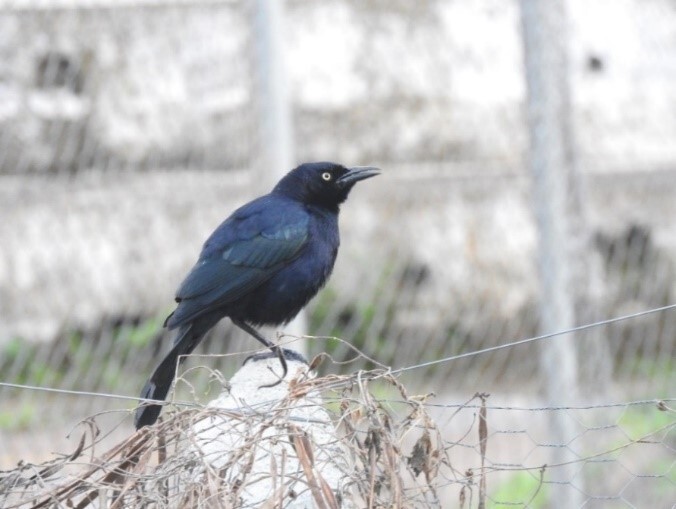
*Quiscalus lugubris*, a species that has expanded its distribution;

**Figure 12d.**
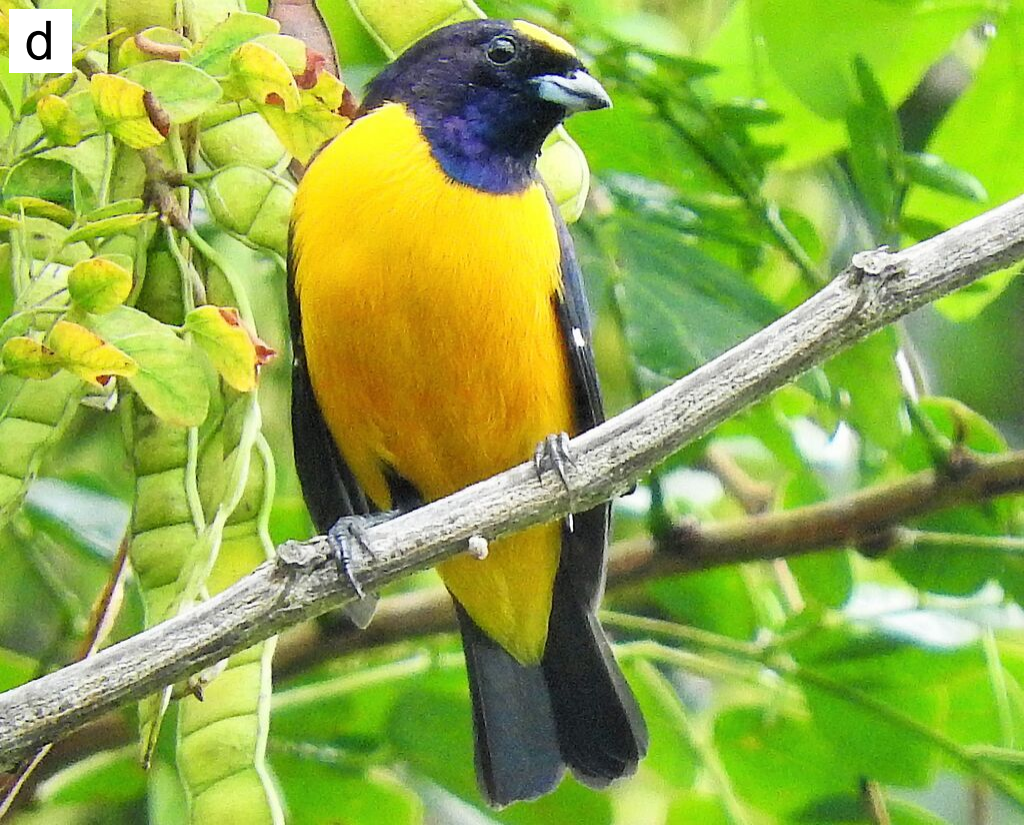
*Euphonia concinna*, an endemic species.

**Figure 13a.**
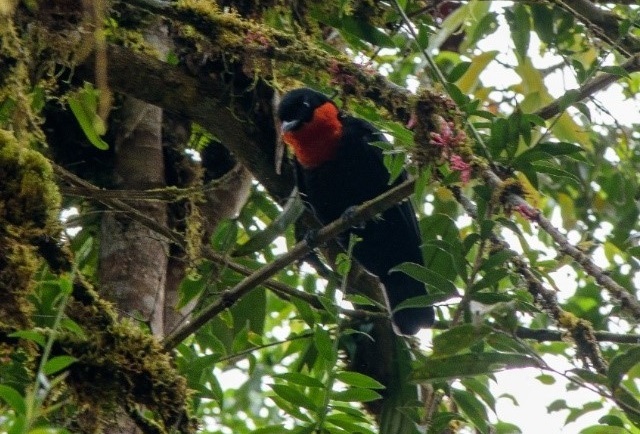
*Pyroderus scutatus* (photograph of. L. Arevalo);

**Figure 13b.**
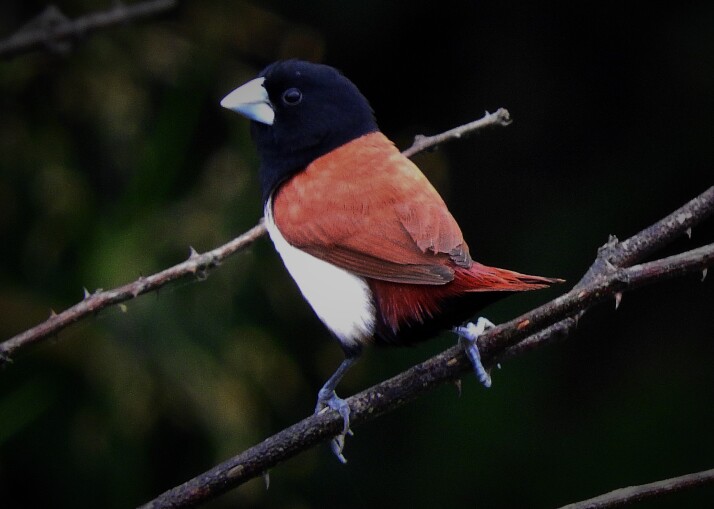
*Lonchura malacca*, an exotic species;

**Figure 13c.**
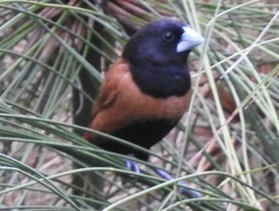
*Lonchura atricapilla*, an exotic species (photograph of D. Rivera).

**Table 1.** Representative localities in the department of Tolima with greater intensity of sampling.

Number	Locality	Municipality	Coordinates	Elevation (m a.s.l.)
1	Anaime	Cajamarca	04°15'3"N; 75°33'35"W	3600
2	Anaime, La Estrella	Cajamarca	04°25'31"N; 75°33'12"W	3400
3	Cristales	Cajamarca	04°27'05"N; 75°31'38"W	2450
4	Carrizales	Cajamarca	04°23'19"N; 75°30'50"W	2050
5	Planadas	Cajamarca	04°25'15"N; 75°22'38"W	1400
6	Potrerillo	Coello	04°14'41"N; 74°58'32"W	433
7	Qda. Martínez	Rovira	04°17'22"N; 75°12'53"W	900
8	Pueblo	Natagaima	03°37'16"N; 75°05'35"W	370
9	Pueblo	Coyaima	03°47'45"N; 75°11'40"W	300
10	Nevado Ruiz	Murillo	04°47'29"N; 75°18'22"W	5320
11	Nevado del Tolima	Ibagué	04°39'06"N; 75°19'32"W	5000
12	La Cueva	Ibagué	04°37'46"N; 75°19'29"W	3600-3800
13	Paso del Quindío	Ibagué	04°33'3"N; 75°26'4.5"W	3480
14	Toche	Ibagué	04°31'10"N; 75°24'32"W	2600
15	Dantas	Ibagué	04°27'05"N; 75°31'18"W	2441
16	Ibanazca	Ibagué	04°34'44"N; 75°19'36"W	2400-2800
17	Silencio	Ibagué	04°36'02"N; 75°19'50"W	2400
18	El Playón	Ibagué	04°35'22"N; 75°18'53"W	2400
19	Rio Chili	Roncesvalles	04°08'77"N; 75°27'54"W	2100
20	Toche	Ibagué	04°32'56"N; 75°24'44"W	2150
21	Clarita Botero	Ibagué	04°28'40"N; 75°13'22"W	1400-1890
22	Piedecuesta	Ibagué	04°26'50"N; 75°15'48"W	1850
23	Juntas	Ibagué	04°32'20"N; 75°13'22"W	1800-1850
24	Mirador	Ibagué	04°28'54"N; 75°12'9"W	1800
25	Laureles	Ibagué	04°33'23"N; 75°22'02"W	1660
26	Cay	Ibagué	04°27'59"N; 75°15'32"W	1600-1700
27	Vda. Chembe	Ibagué	04°28'43"N; 75°09'56"W	1600-1800
28	Ambala	Ibagué	04°28'54"N; 75°12'9"W	1400-2100
29	Santa Teresa	Ibagué	04°26'50"N; 75°15'48"W	1400-1750
30	La Mariposa	Ibagué	04°26'58"N; 75°11'23"W	1300
31	Santa Teresa	Ibagué	04°26'50"N; 75°15'48"W	1345
32	R.N. Gaia	Ibagué	04°27'41"N; 75°12'56"W	1340
33	J.B. San Jorge	Ibagué	04°27'18"N; 75°13'30"W	1286
34	U. del Tolima	Ibagué	04°25'49"N; 75°12'58"W	1070-1170
35	Boquerón	Ibagué	04°24'12"N; 75°17'32"W	1154
36	Aeropuerto-P.Dep.	Ibagué	04°25'49"N; 75°09'52"W	980
37	Lagunas Salado	Ibagué	04°26'52"N; 75°06'51"W	950
38	Salado-Piscícola	Ibagué	04°27'23"N; 75°08'58"W	965
39	Lagunas Picaleña	Ibagué	04°24'19"N; 75°08'27"W	923
40	Totumo	Ibagué	04°22'44"N; 75°11'00"W	900
41	Lagunas Llanitos del Combeima	Ibagué	04°22'43"N; 75°10'45"W	890
42	Chucuni	Ibagué	04°28'5"N; 75°04'25"W	750
43	Laguna del Toro	Ibagué	04°20'36"N; 75°06'27"W	890
44	Ventaquemada	Ibagué	04°30'32"N; 74°59'32"W	740
45	BuenosAires	Ibagué	04°18'30"N; 75°30'36"W	685
46	Rio Chipalo	Alvarado	04°32'23"N; 74°58'39"W	780
47	Lagunas vía Doima	Ibagué	04°23'44"N; 75°04'51"W	540
48	Doima	Piedras	04°23'50"N; 75°02'1.9"W	630
49	M. de los Rodriguez	Piedras	04°35'58"N; 74°49'44"W	270
50	Rio Coello	Gualanday-Ibagué	04°17'02"N; 75°01'09"W	540
51	Corinto	Prado	03°40'19"N; 74°52'35"W	385
52	Restaurante Los Lagos	Guamo	04°04'50"N; 74°55'47"W	380
53	Nataima, SENA	Chicoral	04°11'30"N; 74°57'38"W	390
54	Chicoral	Espinal	04°13'20"N; 74°58'41"W	450
55	Casco Urbano	Espinal	04°09'01"N; 74°53'30"W	323
56	Estación Saldaña	Saldaña	03°56'1,9"N; 75°01'57"W	400
57	Chicuali	San Luis	04°07'4"N; 75°06'6.1"W	437
58	Caso urbano	San Luis	04°8'4.4"N; 75°05'19"W	437
59	Payande	San Luis	04°16'56"N; 75°05'54"W	580
60	Cascadas de Payande	San Luis	04°18'00"N; 75°06'03"W	480
61	Laguna Río Viejo	San Luis	04°07'54"N; 75°05'32"W	365
62	El Puerto	Prado	03°45'30"N; 74°54'29"W	393
63	Embalse-Corinto	Prado	03°47'46"N; 74°53'26"W	390
64	Aco nuevo	Prado	03°43'52"N; 74°49'35"W	430
65	La Virginia	Prado	03°44'13"N; 74°52'2"W	280
66	Anchique	Natagaima	03°34'58"N; 75°05'30"W	320
67	San Pedro	Cunday	04°02'01"N; 74°36'44"W	1000
68	San Antonio	Villahermosa	04°54'42"N; 75°13'44"W	2552
69	Buenos Aires	Prado	03°44'1.6"N; 74°45'2"W	1455
70	La Colonia	Villarrica	03°53'11"N; 74°33'48"W	1803-1972
71	Alto de la Cruz	Villahermosa	05°01'5"N; 75°07'11"W	2188
72	Galilea	Villarrica	03°48'40"N; 74°41'41"W	1750
73	Alto de Bélgica	Villarrica	03°57'16"N; 74°35'33"W	1965
74	Manzanita	Villarrica	03°52'72"N; 74°57'8"W	1600
75	Alto Torres (Galilea)	Villarrica	03°48'46"N; 74°41'57"W	1500
76	Las Catorce	Cunday	04°03'18"N; 74°35'01"W	1703
77	La Arcadia	Villarrica	03°55'11"N; 74°37'23"W	1130
78	San pedro	Cunday	04°03'54"N; 74°38'38"W	1100
79	Varsovia	Cunday	04°04'9"N; 74°35'54"W	900
80	Parroquia Vieja	Cunday	04°01'57"N; 74°34'57"W	400
81	Valencia	Cunday	03°55'46"N; 74°46'10"W	400
82	San José	Dolores	03°31'38"N; 74°49'48"W	1485
83	San Andrés	Dolores	03°31'38.4"N; 74°49"W	1500
84	San Pablo	Dolores	03°38'50"N; 74°48'56"W	860
85	Casco Urbano	Mariquita	05°11'28"N; 74°44'34"W	495-535
86	Casco Urbano	Honda	05°12'17"N; 74°44'26"W	230
87	Honda	Honda	05°14'58"N; 74°50'1.2"W	250
88	Vda. Portachuelo	Icononzo	04°12'29"N; 74°33'55"W	1350
89	Puente Natural	Icononzo	04°11'10"N; 74°30'00"W	850-1000
90	El Chorillo	Ambalema	04°50'32"N; 74°47'15"W	253
91	Tajo	Ambalema	04°48'30'N; 74°47'57"W	255
92	Laguna Coya	Coyaima-Purificación	03°48'43" N; 75°1'40"W	320
93	Purificación	Purificación	03°54'59"N; 75°10'0.4"W	340
94	La Argelia	Venadillo	04°38'22" N; 74°53'3"W	310
95	Potrerito	Venadillo	04°41'49"N; 74°59'22"W	700
96	Alto del Bledo	Lérida	04°41'49.5"N; 74°59'22"W	951
97	Laguna EL Hato	Armero-Guayabal	05°4'26"N; 74°50'0.5"W	290
98	Casco urbano	Guamo	04°1'42"N; 74°58'17"W	330
99	La Siberia	Chaparral	03°53'11"N; 75°41’2"W	1872
100	Desembocadura río Amoya, Guaní	Chaparral	03°40'23"N; 75°23’1"W	540
101	Cuevas Tuluni	Chaparral	03°38'32.3"N; 75°27’26"W	605
102	Caserío Tuluni	Chaparral	03°38'58"N; 75°27’24"W	665
103	Finca Santa Ana, Maito	Chaparral	03°44'21"N; 75°32’42"W	830
104	Pueblo	Venadillo	04°42'45"N; 74°55’32"W	535
105	La Sierra	Venadillo	04°46'40"N; 74°57’47"W	460
106	Uniminuto	Lerida	04°51'18"N; 74°55’01"W	380
107	Piedecuesta	Falan	05°07'27"N; 74°58'16"W	859
108	Puerto Yuca	Falan	05°09'12"N; 74°54'25"W	680
109	Ciudad Perdida	Falan	05°07'00"N; 74°56'31"W	780
110	Vía La Victoria	Mariquita	05°14'05"N; 74°53'19"W	415
111	El Rano	Mariquita	05°17'18"N; 74°51'42"W	365
112	Cataratas de medina	Mariquita	05°13'44"N; 74°53'28"W	560
113	Caimital	Honda	05°12'09"N; 74°45'35"W	463
114	Cruce San Felipe	Armero-Guayabal	05°07'39"N; 74°53'53"W	460
115	Balneario Borbón	Armero-Guayabal	05°04'14"N; 74°50'42"W	460
116	Granja UT	Armero-Guayabal	04°55'23"N; 75°00'34"W	430
117	Vda. La Mejora	Casabianca	05°02'01"N; 75°05'6"W	1700-2000
118	Pueblo	Casabianca	05°04'49"N; 75°07'13"W	2109
119	Cofradia	Venadillo	04°43'35"N; 74°53'57"W	300
120	Limones	Venadillo	04°40'13"N; 74°51'45"W	270
121	Zona rural	Venadillo	04°40'20"N; 74°48'52"W	360
122	Guaimaral	Santa Isabel	04°41'38"N; 75°6'20"W	2100
123	El Resguardo Las Mercedes	Rio Blanco	03°17'31"N; 75°52'02"W	2294
124	Anamichu	Rio Blanco	03°28'6"N; 75°39'48"W	725
125	Alto de Letras	Herveo	05°02'41"N; 75°19'56"W	3600
126	Pueblo	Herveo	05°04'44"N; 75°10'32"W	2250
127	Pueblo	Fresno	05°09'14"N; 75°01'59"W	1260
128	La Cascada	Anzoátegui	04°39'00"N; 75°14'32"W	3640
129	Palomar	Anzoátegui	04°39'4.5"N; 75°13'4"W	2800
130	Puerto Colombia	Anzoátegui	04°35'31"N; 75°09'14"W	2660
131	La Flor	Anzoátegui	04°39'18.4"N; 75°7'3"W	2100
132	La Estrella	Santa Isabel	04°44'36"N; 75°14'22"W	3580
133	La Cabaña	Murillo	04°53'17"N; 75°15'07"W	3200
134	Linea de Paramo	Murillo	04°53'25"N; 75°14'27"W	3600
135	Santa Bárbara	Murillo	04°52'44"N; 75°14'38"W	3300
136	Pueblo	Murillo	04°52'25"N; 75°10'14"W	2950-3000
137	Fifí	Murillo	04°52'12"N; 75°09'15"W	2800
138	El agrado	Libano	04°53'23"N; 75°06'11"W	2190
139	Reserva Isidro Parra	Libano	04°54'42"N; 75°02'51"W	1515
140	Pueblo	Libano	04°55'14"N; 75°03'40"W	1565
141	La Granja	Libano	04°52'34"N; 75°07'17"W	1800-2200
142	Finca El Aguador	Libano	04°55'54"N; 75°01'38"W	1300
143	Chimbi	Melgar	04°12'10"N; 74°43'05"W	319
144	Boquerón-Casco urbano	Melgar	04°12'2.7"N; 74°38'59"W	323-430
145	R.N Victoria	Melgar	04°14'10"N; 74°35'09"W	550-800
146	Pueblo	Melgar	04°12'14"N; 74°38'34"W	320
147	Suarez	Suarez	04°02'12"N; 74°50'09"W	290
148	Gaitania	Planadas	03°10'24"N; 75°38'27"W	1400

**Table 2.** List of the references.

Abbrev.	Reference	Abbrev.	Reference	Abbrev.	Reference
A	Authors	Z3	Molina-Martínez (2009b)	Z32	Lafrancesco et al. (1988)
B	Parra-Hernández et al. (2007)	Z4	Stiles (1995)	Z33	Zimmer (1936), Zimmer (1937), Zimmer (1939), Zimmer (1940)
C	Chapman (1917)	Z5	Harvard University and Morris (2020)(MCZ)	Z34	Olivares (1959)
D	Miller (1952)	Z6	ANSP-Academy of Natural Sciences of Drexel University (2020)	Z35	de-Schauensee (1948), de-Schauensee (1949), de-Schauensee (1950)
E	Losada-Prado and Molina-Martínez (2011)	Z7	FMNH - The Field Museum of Natural History (2020)	Z36	Olivares (1969)
F	Rodríguez (2003)	Z8	MVZ - The Museum of Vertebrate Zoology, Berkeley (2020)	Z37	Chapman (1915)
G	Hilty and Brown (1986)	Z9	CZUT - Colección Zologica de la Universidad del Tolima - referencia Ornitologia (Losada-Prado et al. 2019)	Z38	Apolinar (1950)
H	Marquez et al. (2005)	Z10	Salaman et al. 2008,Salaman et al. (2009)	Z39	Phelps and Phelps (1956)
I	Grupo de Observadores de Aves de Planadas - GOAP (2002)	Z11	IAvH - The Instituto Alexander von Humboldt (Ocampo-Rincón and Borja-Acosta 2019)	Z40	Freeman et al. (2012)
J	Smithsonian National Museum of Natural History - NMNH (2020)	Z12	Nicéforo and Olivares (1965)	Z41	Gutierrez (1995)
K	López et al. (2000), López (2002)	Z13	Xeno-canto (2020)	Z42	Naranjo (2008)
L	Olivares (1960)	Z14	Nicéforo and Olivares (1967)	Z43	Avendaño et al. (2017), Parra-Hernández and Arias-Moreno (2019)
M	Borrero et al. (1962)	Z15	Olson (1981)	Z44	Vargas et al. (2007)
N	Quevedo (2002)	Z16	MSL - Museo de la Salle (Zurc and Bustca 2018)	Z45	Stiles (2009)
Ñ	Quevedo and Gallego (2004)	Z17	Hellmayr and Conover (1942)	Z46	Ayerbe (2015)
O	Miller (1947)	Z18	Lehmann (1957)	Z47	Figueroa et al. (2013)
P	Quevedo and Lopéz (2002)	Z19	Nicéforo (1940)	Z48	Parra-Hernández (2011)
Q	Carvajal and Urueña (2004)	Z20	BioMap Project / Darwin Database (2018)	Z49	Moreno and Losada (2016)
R	Franco and Bravo (2005)	Z21	Dugand (1948)	Z50	Parra-Hernández et al. (2015)
S	Amaya and Rico (2005)	Z22	Nicéforo (1947)	Z51	Molina-Martínez et al. (2015)
T	Parra-Hernández et al. (2008)	Z23	ICN - Instituto de Ciencias Naturales (Raz and Agudelo 2016)	Z52	Molina-Martínez (2014)
U	Losada-Prado et al. (2010)	Z24	Arango (1986)	Z53	Oyuela et al. (2016)
V	Caranton et al. (2008)	Z25	Stone (1899)	Z54	Losada-Prado et al. (2005c)
W	Nicéforo and Olivares (1976a), Nicéforo and Olivares (1976b)	Z26	Renjifo et al. (2002)	Z55	Acevedo and Coral (2017)
X	Quimbayo (2002)	Z27	Lehmann (1957)	Z56	Ayerbe (2018)
Y	Olivares (1973)	Z28	Olivares (1967)	Z57	Parra-Hernández et al. (2019)
Z	Molina-Martínez et al. (2008), Molina-Martinez et al. (2008)	Z29	Nicéforo and Olivares (1978)	Z58	Haffer and Borrero (1965)
Z1	Nicéforo and Olivares (1965)	Z30	Olivares (1957)	Z59	Loaiza-Hernández et al. (2021)
Z2	Molina-Martínez (2009a)	Z31	Niceforo (1923a), Niceforo (1923b)		

**Table 3.** Geographical distribution of species in the Department Tolima.

Geographical distribution	Locality
South-eastern area	Cunday, Villarrica, Purificación, Saldaña, Prado, Coyaima, Natagaima, Alpujarra
Northern area	Mariquita, Honda, Falan, Palocabildo, Fresno, Herveo, Casabianca, Armero, Villahermosa, Líbano, Murillo, Santa Isabel, Anzoátegui, Alvarado, Venadillo, Ambalema, Lérida
South-western area	Roncesvalles, San Antonio, Ortega, Chaparral, Rio Blanco, Ataco, Planadas
Central area	Cajamarca, Ibagué, Piedras, Coello, Flandes, Rovira, San Luis, Guamo, Espinal, Carmen de Apicalá, Melgar, Icononzo, Valle de San Juan

**Table 4.** Species with a probable presence in Tolima.

**Taxon**	**Locality**	**Elevation (m a.s.l.)**	**Habitat**	**Source**	**Observation**
Anatidae					
*Anas georgica* (Gmelin, 1789)	Ib-Pie	500	alp	D	Recorded in census of aquatic birds by K Certuche and C Diaz (GOAT) Subspecie in CR state
*Dendrocygna bicolor* (Vieillot, 1816)	Gi	300	alp	A X	Close to Tolima in Nilo Cundinamarca near Melgar (Piscilagos)
Columbidae					
*Paraclaravis mondetoura* (Bonaparte, 1856)	Li, Vi	1000-3000	fe, g,sf	A	Visually recorded
Cuculidae					
*Coccyzus lansbergi* (Bonaparte, 1850)	Ib	>1000	rf	Ebird	Photographic record of the migratory raptor census by Fello Beltrán (Trópicos Colombia-Conservación de rapaces migratorias) at the counting station located at Hacienda el Escobal on 10 March 2021.
Scolopacidae					
*Calidris subruficollis* (Vieillot, 1819)		< 2600	dfv	Z56	Migrant species
Trochilidae					
*Doryfera johannae* (Bourcier, 1847)	Me	< 1600	gf	Z12	(co USNM 505040)
*Chalcostigma heteropogon* (Boissonneau, 1840)	Tolima snowy	2900-3400	Vi	Z20	(co MNHN CG1941.276)
*Heliangelus amethysticollis* (Longuemare, 1840)	Vi	2200-3000	sf fe gf sppt	Z16 Z20	(co) An ind. from the MLS collection. Probably in areas of the Villarica Municipality.
*Coeligena prunellei* (Bourcier, 1843)	Mu	2600		Z3	A visual record near Nevado of Ruiz (YG Molina). A mislabelled individual from the UCP collection from the south-western slope of the volcán del Tolima (Renjifo et al. 2002).
						Heliornithidae					
*Heliornis fulica* (Boddaert, 1783)	Sl	400	dfv		A reg. M. Moreno in April 2007
Furnariidae					
*Drymotoxeres pucheranii* (Des Murs, 1849)	Fa Ib	1200	sf sgm	B Z10	(v) NT Rec. of Quevedo (2002) which should be confirmed
*Synallaxis subpudica* (Sclater, 1874)	Am	No height defined possibly 1600 m	rf	C Z25	A specimen captured by Stone (1899) in Ambalema. Possibly mislabelled, since Ambalema only refers to heights lower than 600 m a.s.l. and is on the left bank of the Magdalena River.
Passerellidae					
*Atlapetes semirufus* (Sclater, 1840)	Ic	1600-2000		Z16	(co) Some eBirden records on the western slope in the Department of Cundinamarca, near Icononzo
Icteridae					
*Macroagelaius subalaris* (Boissonneau, 1840)	Between Melgar e Icononzo	1800			Old Register of Kaestner, P. In Finding Birds in Colombia. Preliminary report 1991
Fringillidae					
*Euphonia chrysopasta* (Sclater & Salvin, 1869)	Pr		rf	Z20	(co 1898.12.14.284 LNHM)
*Euphonia minuta* (Cabanis, 1849)	Pr		rf	Z20	(co LNHM 1898.12.14.275)
*Euphonia trinitatis* (Strickland, 1851)	Es			Z20	(co MLS 1176)

**Table 5.** .Number of localities (names), richness and endangered species in each area of geographical distribution

**Geographical distribution area**	**Municipality**	**Localities studied**	**Elevation (m a.s.l.)**	**Total species**	**Species: threatened (T), endemic (E), nearly endemic (NE)**
						South-eastern area	Dolores	3	860-1500	105	11 T
							Cunday	5	400-1703	156	5 E
							Villarrica	5	1150-1850	266	5 NE
Suarez	1	290-1000	102			
Purificación	2	300-1400	130			
Saldaña	1	280-1455	117			
Prado	5	300	197			
Coyaima	1	250-300	179			
Alpujarra	1	700-2000	95			
Natagaima	2	250-1850	88			
Total	22	250-1850	398			
Northern area	Mariquita	2	360	252	18 T
	Honda	1	300	285	8 E
	Falan	5	480-1800	408	8 NE
	Palocabildo	2	1100-1700	147	
	Fresno	4	1200-1600	272	
	Herveo	1	2340	164	
	Casabianca	2	1550-2060	185	
	Armero	3	304-350	268	
	Villahermosa	1	2070	173	
	Libano	3	1700-2250	336	
	Murillo	3	2800-3400	227	
	Santa Isabel	1	2100	157	
	Anzoategui	3	2000-3642	197	
	Alvarado	1	350-700	206	
	Venadillo	4	310-700	182	
	Ambalema	3	310-400	174	
	Lerida	3	300-1000	173	
	Total	28	300-3642	681	
South-western area	Roncesvalles	2	2000-3500	176	17 T
	San Antonio	1	900-3000	278	7 E
	Ortega	1	310-2800	105	7 NE
	Chaparral	4	540-830	260	
	Rio Blanco	1	750	100	
	Ataco	1	1600-2100	123	
	Planadas	1	950-4500	279	
	Total	10	540-3500	404	
Central area	Ibagué	31	540-4400	676	27 T
	Cajamarca	4	2500-3800	328	12 E
	Piedras	3	270-600	224	12 NE
	Coello	1	300-1000	179	
	Flandes	1	285	60	
	Rovira	1	800-3600	96	
	San Luis	3	270-350	221	
	Valle de San Juan	1	500-700	93	
	Guamo	1	280-320	180	
	Espinal	1	300-650	236	
	Carmen de Apicala	1	300	129	
	Melgar	1	300-1400	194	
	Icononzo	1	1300-2000	234	
	Total	47	270-4400	687	

## References

[B8005692] Acevedo-Charry O. (2017). Birds of Río Tame of the Andes-Orinoco transitional region: species check-list, biogeographic relationship and conservation. Ornitología Colombiana.

[B6416233] Acevedo O, Coral B (2017). Anotaciones sobre la distribución de *Doliornis
remseni* (Cotingidae) y *Buthraupis
wetmorei* (Thraupidae). Ornitología Colombiana.

[B6416242] Álvarez M (2000). Aves de la isla Malpelo. Biota Colombiana.

[B6309929] Álvarez M, Umaña A, Mejía G, Cajiao J, Hildebrand V, Gast F (2003). Aves del parque nacional serranía de Chiribiquete, Amazonia-Provincia de la Guyana, Colombia.. Biota Colombiana.

[B6309949] Amaya J, Rico G (2005). Caracterización y evaluación del peligro aviario presente en el aeropuerto Perales de Ibagué, Colombia..

[B6309957] University ANSP-Academy of Natural Sciences of Drexel (2020). ANSP Systematics: Ornithology collection search.. http://clade.ansp.org/ornithology/index.php..

[B6416435] Apolinar M (1950). Vocabulario de términos vulgares en historia natural colombiana. Revista de la Academia Colombiana de Ciencias Exactas, Fisicas y Naturales.

[B6416372] Arango G (1986). Distribución del género *Gallinago* Brisson, 1760 (Aves: Scolopacidae) en los Andes Orientales de Colombia.. Caldasia.

[B8787643] Arbeláez-Cortés E, Marín-Gómez OH, Duque-Montoya D, Cardona-Camacho PJ, Renjifo LM, Gómez H Fabio (2011). Birds, Quindío Department, Central Andes of Colombia. Check List.

[B6310003] Ornitología Asociación Colombiana de (2020). Lista de referencia de especies de aves de Colombia.. https://ipt.biodiversidad.co/sib/resource?r=aco_listaavescolombia2017.

[B6310285] Avendaño J, Bohórquez C, Rosselli L, Arzuza D, Estela F, Stiles F, Renjifo L (2017). Lista de chequeo de las aves de Colombia: Una síntesis del estado del conocimiento desde Hilty y Brown (1986). Ornitología Colombiana.

[B7996766] Avendaño J. E., López-O J. P., Laverde-R O. (2018). New bird records from the arid Cúcuta Valley, north-east Colombia. Bulletin of the British Ornithologists’ Club.

[B6815307] Ayerbe F, López J, González M, Estela F, Ramírez M, Sandoval V, Gómez L (2008). Aves del departamento del Cauca-Colombia. Biota Colombiana.

[B6416444] Ayerbe F (2015). Colibríes de Colombia.

[B6310348] Ayerbe F (2018). Guía ilustrada de la avifauna colombiana.

[B6310367] Bejarano D, García J, González A, Machado J, Oyuela G, Yate A (2005). Informe final. Estudio preliminar de fauna y flora de la reserva natural Ibanasca.

[B6310388] Database BioMap Project / Darwin (2018). Distribution database of colombian avifauna. http://biomap.net/.

[B6310957] Borrero J, Olivares A, Camacho J (1962). Notas sobre aves de Colombia. Caldasia.

[B6310967] Calderón J, Flórez A, Cabrera Y (2011). Aves del departamento de Nariño. Biota Colombiana.

[B6310978] Caranton D, Certuche K, Jaramillo C, Parra R, Sanabria J, Moreno M (2008). Aspectos biológicos de una nueva población del capuchino de cabeza negra (*Lonchura
malacca*, Estrildidae) en el alto valle del Magdalena, Tolima. Boletín SAO.

[B6311000] Cárdenas G, Ramírez-Mosquera D, Eusse-González D, Fierro-Calderón E, Vidal-Astudillo V, Estela F (2020). Aves del departamento del Valle del Cauca, Colombia. Biota Colombiana.

[B8787753] Carrillo-Chica E, Jaramillo L F, Portura M Á (2018). La avifauna del departamento de Vaupés, escudo Guayanés, amazonia colombiana. Revista Colombia Amazónica.

[B6311140] Carvajal A, Urueña L (2004). Distribución de la caminera tolimense (*Leptotila conoveri*) según el grado de intervención en dos localidades del departamento del Tolima.

[B6311221] Chaparro S, Echeverry M, Córdoba S, Sua A (2013). Listado actualizado de las aves endémicas y casi-endémicas de Colombia. Biota Colombiana.

[B6311230] Chaparro S, Lopera S, Stiles F (2018). Aves del departamento de Cundinamarca, Colombia conocimiento, nuevos registros y vacíos de información. Biota Colombiana.

[B6311239] Chapman F (1912). Diagnoses of apparently new Colombian birds. Bulletin of the American Museum of Natural History.

[B6311388] Chapman F (1915). The more northern species of the genus *Scytalopus* Gould. The Auk.

[B6311379] Chapman F (1917). The distribution of bird-life in Colombia, a contribution to a biological survey of South America. Bulletin of the American Museum of Natural History.

[B6311397] Cohn-Haft M, Whittaker A, Stouffer P (1997). A new look at the “species - poor” central Amazon: The avifauna north of Manaus, Brazil. Ornithological Monographs.

[B6311406] Colwell R EstimateS: Statistical estimation of species richness and shared species from samples. Version 9. User´s Guide. 9.1 Department of ecology & evolutionary biology, University of Connecticut. http://viceroy.eeb.uconn.edu/estimates.

[B6311414] Córdoba S (2009). Historia de la ornitología colombiana: sus colecciones científicas, investigadores y asociaciones. Boletín SAO.

[B8005652] Cordoba S. Cordoba -, Sierra S. (2018). Nuevos registros y ampliación de distribución de aves en la vertiente occidental, cordillera oriental, Santander, Colombia. Acta Biológica Colombiana.

[B6311423] Society Cornell Lab of Ornithology and National Audubon eBirds: Distribution database for location of avifauna (Tolima). https://ebird.org/region/CO-TOL?yr=all.

[B6311431] (CORTOLIMA) Corporación Autónoma Regional del Tolima (1996). Inventario de fauna silvestre de las cuencas de los ríos Combeima, Toche y Tochecito: Investigación biológica de peces, anfibios, reptiles, aves y mamíferos..

[B6325908] de-Schauensee R M (1948). The birds of the republic of Colombia: Their distribution, and keys for their identification. Caldasia.

[B6325917] de-Schauensee R M (1949). The birds of the Republic of Colombia (Accipitridae - Picidae). Caldasia.

[B6325926] de-Schauensee R M (1950). The birds of the Republic of Colombia (Dendrocolaptidae - Tyrannidae). Caldasia.

[B6325935] de-Schauensee R M (1951). The birds of the Republic of Colombia (Alaudidae - Fringillidae). Caldasia.

[B6311439] Donegan M, Avendaño J, Briceño E, Luna J, Roa C (2010). Aves de la serranía de los Yariguíes y tierras bajas circundantes, Santander, Colombia. Cotinga.

[B6314807] Dugand A (1941). Adiciones a la lista de aves conocidas en Colombia. Caldasia.

[B6314816] Dugand A (1948). Noticias ornitológicas colombianas. Caldasia.

[B6314829] Figueroa W, Parra R, Martínez C, Torres A (2013). Aves del bosque seco tropical, Tolima, Colombia. http://fieldguides.fieldmuseum.org/sites/default/files/rapid-color-guides-pdfs/467.pdf.

[B6314837] History FMNH - The Field Museum of Natural (2020). Catalogue: Zoological collections.. https://collectionszoology.fieldmuseum.org/list.

[B6314845] Franco A., Bravo G., Boyla K., Estrada A. (2005). Áreas importantes para la conservación de las aves en los andes tropicales: sitios prioritarios para la conservación de la biodiversidad.

[B6314858] Freeman B, Hilty S, Calderón D, Ellery T, Urueña L (2012). New and noteworthy bird records from central and northern Colombia. Cotinga.

[B8005662] Gómez C., Cadena C. D., Cuervo A. M., Díaz-Cárdenas J., García-Cardona F., Niño-Rodríguez N., Ocampo-Peñuela N., Ocampo D., Seeholzer G., Sierra-Ricaurte A., Soto-Patiño J. (2022). Reexpedición Colombia: Entender el pasado para empoderar acciones que fortalezcan el conocimiento y conservación de las aves. Biota Colombiana.

[B6314868] GOAP Grupo de Observadores de Aves de Planadas - (2002). Lista de aves observadas en el municipio de Planadas. https://www.proaves.org/.

[B6314876] Gutierrez F (1995). Registros en Girardot. https://www.avesbogota.org/category/clarinero/.

[B6314884] Haffer J, Borrero J (1965). On birds from northern Colombia. Revista de Biología Tropical.

[B6314977] University Harvard, Morris PJ (2020). Museum of Comparative Zoology.

[B6314996] Hellmayr C, Conover B (1942). Catalogue of birds of the Americas and the adjacent islands in Field Museum of Natural History and including all species and subspecies known to occur in North America, Mexico, Central America, South America, the West Indies, and islands of the Caribbean Sea, the Galapagos Archipelago, and other islands which may properly be included on account of their faunal affinities.

[B6315008] Hilty S, Brown S (1986). A guide to the birds of Colombia.

[B7961787] Hochkirch A., Samways M. J., Gerlach J., Böhm M., Williams P., Cardoso P., Cumberlidge N., Stephenson P. J., Seddon M. B., Clausnitzer V., Borges P. A. V., Mueller G. M., Pearce-Kelly P., Raimondo D., Danielczak A., Dijkstra K. (2022). A strategy for the next decade to address data deficiency in neglected biodiversity. Conservation Biology.

[B8005836] Evaluación Instituto de Investigación de Recursos Biológicos Alexander von Humboldt Programa de BioModelos. http://biomodelos.humboldt.org.co/es.

[B6315016] IGAC Instituto Geográfico Agustín Codazzi - (1996). Diccionario geográfico de Colombia.

[B6315028] IGAC Instituto Geográfico Agustín Codazzi - (2004). Estudio general de suelos y zonificación de tierras departamento de Tolima.

[B6315044] IUCN International Union for Conservation of Nature - The IUCN Red list of threatened species. http://www.iucnredlist.org/.

[B6315052] Krabbe N, Flórez P, Suárez G, Castaño J, Arango J, Duque A (2006). The birds of Páramo de Frontino, western andes of Colombia. Ornitologia Colombiana.

[B6315063] Lafrancesco G, Mateus C, Martínez C (1988). Contribución al estudio de los Passeriformes rhinocryptidos y cotingidos de Colombia, entregas 4 y 5, rhinocryptidos y cotingidos del Museo de Ciencias Naturales de la Universidad de La Salle. Boletín Científico de la Universidad de la Salle.

[B6315076] Legendre P, Legendre L (1998). Numerical ecology.

[B6315088] Lehmann F (1957). Contribuciones al estudio de la fauna de Colombia XII. Novedades Colombianas.

[B9045251] Loaiza-Hernández H, Flórez-Delgado VT, Quevedo-Gil A (2021). Ampliación del rango de distribución de *Chlorochrysa
nitidissima* (Thraupidae) hacia el departamento del Tolima. Ornitología Colombiana.

[B6315098] López B, Salaman P, Cowley T, Arango S, Rengifo L (2000). The threatened birds of the río Toche, cordillera central, Colombia. Cotinga.

[B6315108] López B, Renjifo LM, Franco AM, Amaya J, Kattan G, López B (2002). Libro rojo de aves de Colombia. Serie libros rojos de especies amenazadas de Colombia.

[B6321721] Losada-Prado S, Molina-Martínez YG, González AM, Carvajal AM, Franco LM, Villa F, Reinoso G, Bernal M, Losada S (2003). Biodiversidad faunística de la cuenca del río Coello. Biodiversidad regional fase I..

[B6321735] Losada-Prado S, Carvajal A, Molina-Martínez YG (2005). Listado de especies de aves de la cuenca del río Coello (Tolima, Colombia). Biota Colombiana.

[B6321744] Losada-Prado S, González A, Carvajal A, Molina-Martínez Y G (2005). Especies endémicas y amenazadas registradas en la cuenca del río Coello (Tolima) durante estudios rápidos en 2003. Ornitología Colombiana.

[B6321753] Losada-Prado S, Murillo J, Carvajal A, Parra R, Losada S, Villa-Navarro F, Reinoso G, Esquivel H, Garcia-Melo J, Vejarano M, Guevara G (2005). Biodiversidad faunística y florística de la cuenca del río Prado. Biodiversidad regional fase II.

[B6321706] Losada-Prado S, Molina-Martínez YG, Moreno-Palacios M, Reinoso-Florez G, Villa-Navarro FA, Losada-Prado S, Garcia-Melo JE, Vejarano M, Molina-Martínez YG (2010). Biodiversidad faunística y florística de la cuenca mayor del Río Gualí. Biodiversidad regional fase VI.

[B6321769] Losada-Prado S, Molina-Martínez Y G (2011). Avifauna del bosque seco tropical en el departamento del Tolima (Colombia): análisis de la comunidad. Caldasia.

[B6321779] Losada-Prado S, Sánchez J N, Gaitán C D (2019). Colección zoológica de la Universidad del Tolima (CZUT) - Ornitología.

[B6321787] Lozano B, Gómez A, Valderrama C (2011). Estado de fragmentación de los bosques naturales en el norte del departamento del Tolima-Colombia. Revista Tumbaga.

[B6321805] Ludwing J, Reynolds J (1998). A primer on methods and computing statistical ecology.

[B8005722] Marinho L. C., Beech E. (2020). How phantom databases could contribute to conservation assessments. The Science of Nature.

[B6321813] Marquez C, Bechard M, Gast F, Vanegas V (2005). Aves rapaces diurnas de Colombia..

[B6321821] Miller A (1947). The tropical avifauna of the upper Magdalena valley, Colombia. Auk.

[B6321830] Miller A (1952). Supplementary data on the tropical avifauna of the arid Upper Magdalena valley of Colombia. Auk.

[B6321839] Molina-Martinez Y G, Rodríguez Q, Reinoso G, Villa F, Esquivel H, Garcia J, Vejarano M (2007). Biodiversidad faunística y florística de la cuenca del Río Totare. Biodiversidad regional fase III.

[B6321855] Molina-Martinez Y G, Díaz H, Gomez C, Reinoso G, Villa F, Esquivel H, Garcia J, Vejarano V (2008). Biodiversidad faunística y florística de la cuenca mayor del rio Saldaña (subcuenca Anamichú). Biodiversidad regional fase IV.

[B6321871] Molina-Martínez Y G, Díaz H, Gómez C, Reinoso G, Villa F, Esquivel H, Garcia J, Vejarano M (2008). Biodiversidad faunística y florística de la cuenca del río Lagunillas. Biodiversidad regional fase IV.

[B6321903] Molina-Martínez Y G, Reinoso-Florez G, Villa-Navarro F A, Garcia-Melo J E, Vejarano-Delgado M A (2009). Biodiversidad faunística y florística de la cuenca mayor del río Saldaña (Subcuencas Mendarco y Guanábanos). Biodiversidad regional fase V.

[B6321916] Molina-Martínez Y G, Reinoso-Florez G, Villa-Navarro F A, García-Melo J E, Vejarano-Delgado M A (2009). Caracterización de fauna y flora de los páramos del Tolima.

[B6321885] Molina-Martínez Y G (2014). Birds of the Totare river basin, Colombia. Check List.

[B6321894] Molina-Martínez Y G, García J, Losada S (2015). Evaluación rápida de las aves de la parte baja de la cuenca del río Anamichú, municipio de Rio Blanco–Tolima. Revista Tumbaga.

[B6321929] Moreno M, Losada S (2016). Avifauna del complejo de páramos Chilí-Barragán (Tolima, Colombia). Biota Colombiana.

[B6321938] MVZ - The Museum of Vertebrate Zoology Berkeley (2020). Collection database, Museum of Vertebrate Zoology. http://arctos.database.museum/SpecimenSearch.cfm?guid_prefix=MVZ%3ABird.

[B6321946] Naranjo L (2008). Lista de aves observadas en las salidas diarias durante el encuentro. Boletín SAO.

[B6321955] Naranjo L, Amaya J, Eusse-González D, Cifuentes-Sarmiento Y (2012). Guía de las especies migratorias de la biodiversidad en Colombia: aves.

[B6321963] Niceforo M (1923). Troquílidos del Museo del Instituto de La Salle, I. Boletín de la Sociedad de La Salle.

[B6321972] Niceforo M (1923). Troquílidos del Museo del Instituto de La Salle, II. Boletín de la Sociedad de La Salle.

[B6321981] Nicéforo M (1940). Troquilidos del Museo del Instituto La Salle. Revista de la Academia Colombiana de Ciencias Exactas, Físicas y Naturales.

[B6321990] Nicéforo M (1947). Notas sobre aves de Colombia, II. Caldasia.

[B6321999] Nicéforo M (1964). Adiciones a la avifauna colombiana, I (Tinamidae-Falconidae). Boletin del Instituto La Salle.

[B6322008] Nicéforo M, Olivares A (1965). Adiciones a la avifauna colombiana, II (Cracidae, Rynchopidae). Boletin de la Sociedad Venezolana de Ciencias Naturales.

[B6322017] Nicéforo M, Olivares A (1967). Adiciones a la avifauna colombiana, IV (Apodidae-Picidae). El Hornero.

[B6322026] Nicéforo M, Olivares A (1975). Adiciones a la avifauna colombiana, VI (Tyrannidae-Bombicillidae). Lozania.

[B6322035] Nicéforo M, Olivares A (1976). Adiciones a la avifauna colombiana, VI (Tyrannidae-Bombicillidae). Lozania.

[B6322044] Nicéforo M, Olivares A (1976). Adiciones a la avifauna colombiana, VI (Tyrannidae-Bombicillidae). Lozania.

[B6322053] Nicéforo M, Olivares A (1978). Adiciones a la avifauna colombiana VII (Vireonidae-Fringillidae). Revista de la Universidad Social Católica de La Salle.

[B6322062] Ocampo-Rincón D, Borja-Acosta K (2019). Colección de Aves del instituto de investigación de recursos biológicos Alexander von Humboldt (IAvH-A).

[B6322070] Olivares A (1957). Aves de la Costa del Pacífico, Municipio de Guapi, Cauca, Colombia, II. Caldasia.

[B6322079] Olivares A (1959). Aves migratorias en Colombia. Revista de la Academia Colombiana de Ciencias Exactas, Físicas y Naturales.

[B6322088] Olivares A (1960). Algunas aves de Gaitania (Municipio de Ataco, Tolima, Colombia). Caldasia.

[B6322097] Olivares A (1967). Avifaunae Columbiensis (Notulae 2). Seis nuevas aves para Colombia y apuntamiento sobre 60 especies y subespecies registradas anteriormente. Caldasia.

[B6322115] Olivares A (1969). Aves de Cundinamarca.

[B6322145] Olivares A (1973). Las ciconiiformes colombianas (garzas, coclearios, ibis, cigueñas, espatulas, flamencos).

[B6322352] Olson S (1981). Interaction between the two subspecies groups of the seed-finch *Sporophila
angolensis* in the Magdalena Valley, Colombia. The Auk.

[B6322361] Oyuela G, Goméz R, Duarte L, Urueña L, Arias H (2016). Aves del jardin botánico San Jorge.

[B8005703] Palacio R. D., Kattan G. H., Pimm S. L. (2019). Bird extirpations and community dynamics in an Andean cloud forest over 100 years of land-use change. Conservation Biology.

[B9045770] Parra-Hernández RM, Vargas-Oviedo J, Certuche-Cubillos K (2022). Ampliación de la distribución de Touit
stictopterus (Psittacidae) para la cordillera Central, Colombia. Ornitología Colombiana.

[B6325267] Parra-Hernández R M (2006). Caracterización de la avifauna de la cuenca del río Prado (Tolima).

[B6325366] Parra-Hernández R M, Carantón D, Sanabria J, Barrera L, Sierra A, Moreno M, Yate W, Figueroa W, Díaz C, Florez V, Certuche K, Loaiza H, Florido B (2007). Aves del municipio de Ibagué - Tolima, Colombia. Biota Colombiana.

[B6322475] Parra-Hernández R M, Carantón D, Moreno M, Sanabria J (2008). Primer registro del vencejo cuatro ojos (*Cypseloides
cherriei*) para la cordillera central de los andes (Colombia). Ornitología Colombiana.

[B6325404] Parra-Hernández R M (2011). Aves del Clarita Botero, Ibagué, Tolima, Colombia. http://fieldguides.fieldmuseum.org/sites/default/files/rapid-color-guides-pdfs/311_Aves_Clarita_Botero_02_1.pdf.

[B6325429] Parra-Hernández R M, Tolosa Y, Figueroa W (2015). Nuevos registros y estado actual de las especies introducidas en el municipio de Ibagué. Revista Tumbaga.

[B6322420] Parra-Hernández R M, Arias-Moreno H D (2019). Primer registro de *Phyllomyias
burmeisteri* para la cordillera central de los andes colombianos, con comentarios en su variación acústica. Ornitología Colombiana.

[B6322518] Parra-Hernández R M, Poveda J, Juez D (2019). Nuevo registro de distribución de *Mustelirallus
erythrops* (Rallidae) para el Valle del Alto Magdalena, Colombia. Boletín SAO.

[B6325420] Parra R M, Rojas C (2011). Aporte a la distribución del pato careto (*Dendrocygna
viduata*) en el departamento del Tolima.. Revista Tumbaga.

[B6325438] Phelps W, Phelps W (1956). Five new birds from río Chiquito, Táchira, Venezuela and two extensions of ranges from Colombia. Proceedings of the Biological Society of Washington.

[B6325766] Pomar J, Vargas G (1985). Estudio preliminar para determinar las diferentes zonas de vida en el departamento del Tolima según el sistema de Holdrige.

[B6325774] Quevedo A (2002). Primer aporte sobre la composición taxonómica de la avifauna del municipio de Ibagué. www.proaves.org/.

[B6325782] Quevedo A, Lopéz B (2002). Registro extralimital de *Sturnella
magna* en Colombia: Tolima, Cordillera central. Boletin SAO.

[B6325791] Quevedo A, Gallego O (2004). Estudio fauna municipio de Fresno (Tolima). En: Plan básico de ordenamiento territorial (POT) de Fresno. Componente fauna.

[B6325799] Quimbayo M (2002). Efectos del paisaje sobre la distribución de la avifauna en fragmentos de bosque seco tropical en el área del parque recreacional - Piscilago (Nilo-Cundinamarca).

[B6325807] Raz L, Agudelo H (2016). ICN - Universidad Nacional de Colombia.

[B6325815] Reinoso G, Garcia J, Vejarano M, Villa F, Guevara G, Molina-Martínez Y G, Garcia L J, Galindo Y, Yara D C, Carranza X, Peña J M, Gutierrez C, López E O, Parra Y T, Gutierrez K A, Yara C L, Vasquez J M (2009). El Tolima: diversidad en el corazón de los andes colombianos.

[B6325836] Remsen J VJ, Areta J, Bonaccorso E, Claramunt S, Jaramillo A, Pacheco J, Robbins M, Stiles F, Stotz D, Zimmer K A classification of the bird species of South America, American Ornithological Society. http://www.museum.lsu.edu/~Remsen/SACCBaseline.htm.

[B6325850] Renjifo L, Franco A, Amaya J, Kattan G, López B (2002). Libro rojo de las aves de Colombia. Serie libros rojos de especies amenazadas de Colombia.

[B6325860] Restall R, Rodner C, Lentino M (2006). Birds of northern South America: An identification guide.

[B6325868] Rodríguez Q (2003). Estudio de la comunidad aviaria en la reserva natural Semillas de Agua, páramo de los Valles Cajamarca Tolima. www.proaves.org.

[B6325876] Salaman P, Donegan T, Caro D (2008). Listado de avifauna colombiana. Conservación Colombiana.

[B6325885] Salaman P, Donegan T, Caro D (2009). Listado de aves de Colombia. Conservación Colombiana.

[B6325944] NMNH Smithsonian National Museum of Natural History - (2020). Smithsonian National Museum of Natural History, Search the Division of Birds Collections. https://collections.nmnh.si.edu/search/birds/.

[B6326011] Stiles F (1995). Distribución y variación en el ermitaño carinegro (*Phaetornis
anthophilus*) en Colombia. Caldasia.

[B6325998] Stiles F, Chaves M E, Arango N (1998). Informe nacional del estado de la biodiversidad..

[B6326020] Stiles F (2009). A review of the genus *Momotus* (Coraciiformes: Momotidae) in Northern South America and adjacent areas. Ornitología Colombiana.

[B6326033] Stone W (1899). On a collection of birds from the vicinity of Bogotá, with a review of the South American species of *Speotyto* and *Troglodytes*. Proceedings of the Academy of Natural Sciences of Philadelphia.

[B6326042] Vargas R, Rojas L, Lozano A, Pérez U, Quimbayo M, Fernández D, Trujillo M, Murillo M, Bonilla J, Dávila Y, Trujillo J, Valderrama S, Gómez F, Aguirre A, Frausin M, Loaiza H, Mayorca H, Yaya M, Barrios G, Forero J (2007). Plan genereal de ordenación forestal para el departamento del Tolima. https://www.cortolima.gov.co/sites/default/files/images/stories/centro_documentos/estudios/tomo_01.pdf.

[B6326070] Verhelst J, Rodriguez J, Orrego O, Botero J, López J, Franco V, Pfeifer A (2011). Aves del municipio de Manizales - Caldas, Colombia. Biota Colombiana.

[B6326094] Wirt R (1895). A flying trip to the tropics, a record of an ornithological visit to the United states of Colombia, South America and to the Island of Curacao West Indies in the year 1892.

[B6326114] Xeno-canto Xeno-canto: Sharing bird sounds from around the world. https://www.xeno-canto.org/.

[B6326122] Zimmer J (1936). Studies of Peruvian birds XXIV. Notes on *Pachyramphus, Platypsaris, Tityra*, and *Pyroderus*. American Museum Novitates.

[B6326131] Zimmer J (1937). Studies of Peruvian Birds Nº XXVII - Notes on the genera *Muscivora, Tyrannus, Empidonomus* and *Knipolegus*. American Museum Novitates.

[B6326140] Zimmer J (1939). Studies of Peruvian birds Nº XXXIII - The genera *Tolmomyias* and *Rhynchocyclus* with further notes on Ramphotrigon. American Museum Novitates.

[B6326149] Zimmer J (1940). Studies of Peruvian Birds Nº XXXIV - The genera *Todirostrum*, *Euscarthmornis*, *Snethlagea, Poecilotriccus, Lophotriccus, Myiornis, Pseudotriccus*, and *Hemitriccus*. American Museum Novitates.

[B6326158] Zurc D, Bustca A (2018). Colección de ornitología - Museo de Ciencias Naturales de La Salle.

